# Reactivity of Pnictaalumenes Towards 1,3‐Dipole Molecules

**DOI:** 10.1002/anie.202506356

**Published:** 2025-06-17

**Authors:** Tim Wellnitz, Edgar Zander, Leonie Wüst, Jakob Boardman, Max Neubauer, Samuel Nees, Holger Braunschweig, Christian Hering‐Junghans

**Affiliations:** ^1^ Institute for Inorganic Chemistry Julius‐Maximilians‐Universität Würzburg Am Hubland 97074 Würzburg Germany; ^2^ Institute for Sustainable Chemistry & Catalysis with Boron Julius‐Maximilians‐Universität Würzburg Am Hubland 97074 Würzburg Germany; ^3^ Leibniz Institute for Catalysis e.V. (LIKAT) A.‐Einstein‐Str. 29a 18059 Rostock Germany

**Keywords:** 1,3‐Dipoles, Azides, Inorganic rings, Phosphorus, Pnictaalumenes

## Abstract

Alkynes undergo 1,3‐dipolar cyclization reactions with organic azides giving 1,2,3‐triazoles. Pnictaalumenes RPn═AlRˈ are the isoelectronic congeners of alkynes, hence a similar reactivity toward 1,3‐dipole molecules is expected. Herein, we report the reactions of ^Dipp^TerPn═AlCp* (^Dipp^Ter  =  2,6‐(2,6‐*i*Pr_2_C_6_H_3_)‐C_6_H_3_, Cp*  =  [Me_5_C_5_], Pn  =  P, As) toward aryl azides. Instead of the anticipated phosphaaluminatriazole products from a [3+2] cyclization, the formation of four‐membered AlN_2_Pn ring systems of the general form ^Dipp^TerPn(*μ*‐NR)_2_AlCp* was observed. Depending on the steric profile of the azide, different outcomes were observed: a) N*_α_* insertion into the Pn═Al bond accompanied by twofold N_2_ elimination, b) N*_γ_* insertion under preservation of the N_3_ motif, or c) mixed N*_a_*/N*_γ_* products. The bonding situation is complex and best described as Al(III) complexes with NPnN‐amino‐amide‐type ligands. A twofold insertion also occurs with the isovalent electronic diazoalkane Me_3_Si(H)C═N_2_, yielding imine‐substituted AlN_2_Pn derivatives. In the case of its P‐congener, two thermal rearrangements were observed, which first result in a AlN_3_P ring species with a terminal Al─CN unit, followed by a second slower rearrangement to an aluminate cyanido complex with an unusual NNN pincer‐type ligand framework. The current study contrasts well‐established [3+2] cyclization reactions between alkynes and 1,3‐dipoles and provides access to unprecedented ligand motifs.

## Graphical abstract

Pnictaalumenes with a Pn═Al multiple bonds undergo azide or diazoalkene insertions rather than [3+2] cycloadditions. This results in AlN_2_P heterocycles, which show a great structural variety depending on the sterics of the azide. With TMS‐diazoalkane, thermal rearrangements afford rare cyanidoaluminates.

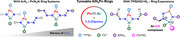

## Introduction

Since their discovery in the late 19^th^ century, 1,3‐dipoles such as azides or diazoalkanes have attracted considerable attention in organic synthesis.^[^ ^1^, ^2^ ^]^ They are indispensable building blocks for N‐heterocycles (*cf*. aza‐Wittig‐cyclization),^[^ ^3^ ^]^ facilitate functional group transformations (*cf*. Staudinger ligation),^[^ ^4^ ^]^ or serve as synthons for electron deficient species like nitrenes or carbenes, respectively.^[^ ^5^, ^6^, ^7^, ^8^, ^9^, ^10^, ^11^, ^12^ ^]^ In 1960, Huisgen disclosed his groundbreaking work on 1,3‐dipolar cycloadditions of alkynes with azides, as 1,3‐dipole molecules, to give triazoles.^[^ ^13^, ^14^, ^15^ ^]^ These highly selective, reliable, and bioorthogonal reactions have become a cornerstone of “click chemistry” (in case of the Cu‐catalyzed variant), with widespread synthetic applications.^[^ ^16^, ^17^, ^18^ ^]^

**Scheme 1:**
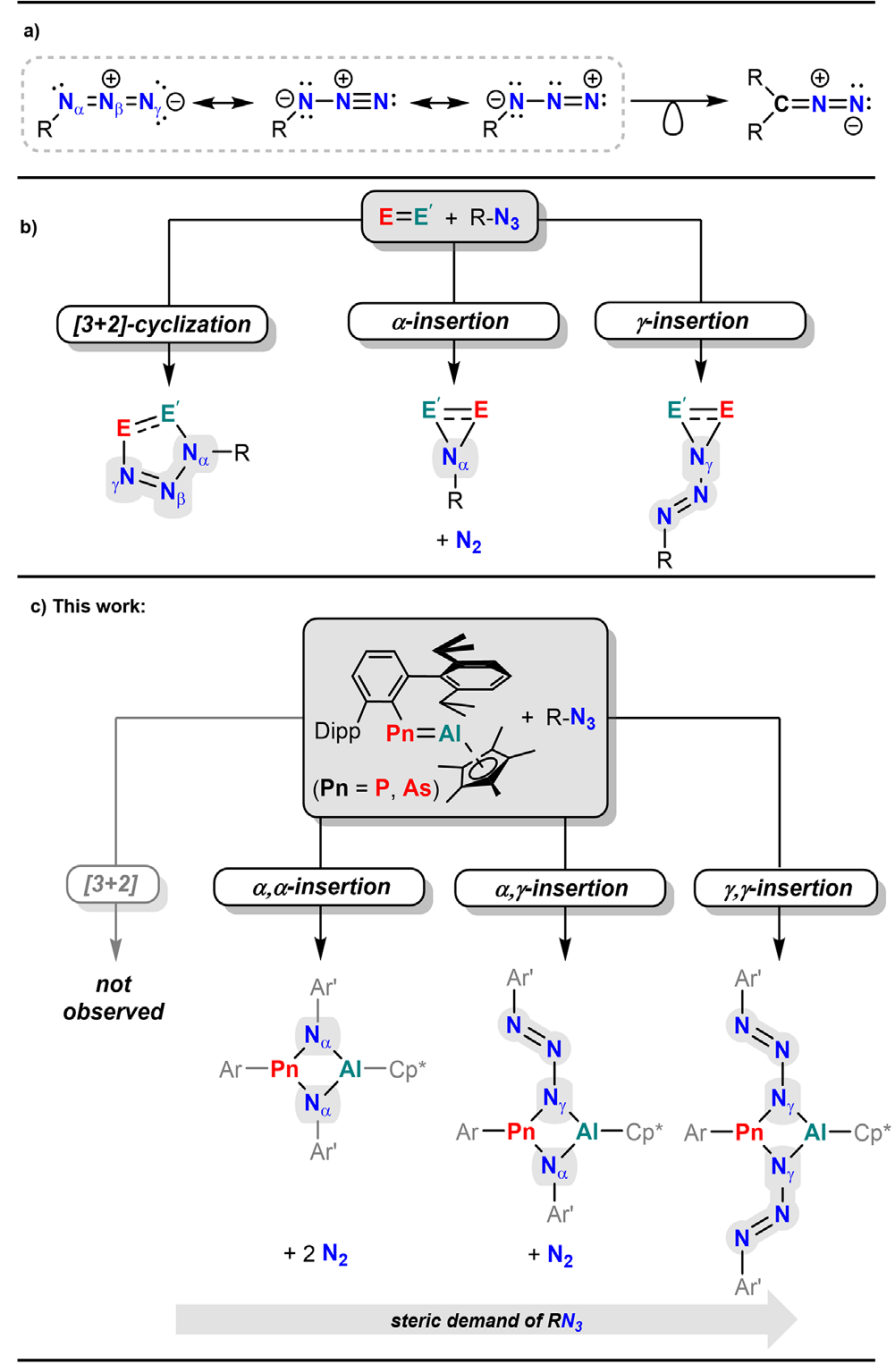
a) Resonance scheme of organic azides and their isolobal relation to diazaalkanes, b) reactivity pattern of azides toward element‐element multiple bonds, and c) outline of this study.

As per definition, 1,3‐dipole molecules contain a delocalized electron system over three atoms, with opposing charges in 1,3‐position in one of the resonance structures.^[^ ^13^ ^]^ In azides, this charge separation results in a nucleophilic N_α_ and electrophilic N_γ_ atom, enabling either N_α_ nitrene insertion with simultaneous N_2_ release or terminal N_γ_ insertion under preservation of the N_3_ chain (Scheme 1a,b).^[^ ^19^ ^]^ Heterodiatomic multiple bond species like iminoboranes, with a formal B≡N triple bond, show similar “inorganic” [3+2] cyclization reactions with azides, which give five‐membered tetraazaboroles (Scheme 1b).^[^ ^20^, ^21^ ^]^ However, due to the highly polarized nature of the E═E’ bonds (E, E’ = different main group elements), reaction pathways beyond [3+2] cyclizations are possible.^[^ ^22^, ^23^, ^24^, ^25^, ^26^, ^27^ ^]^

Although [3+2] cyclizations with azides have been reported for iminoalanes (RAlNR’),^[^ ^28^, ^29^, ^30^, ^31^, ^32^, ^33^, ^34^ ^]^ the reactivity of the heavier pnictatrielenes, RE^13^═E^15^R’, toward azides remains largely unexplored. In this context, Wang and coworkers recently reported the reaction of a germyl‐substituted gallaarsene with adamantyl azide in a [3+2] fashion, which was followed by migration of the germyl substituent from As to N.^[^ ^35^ ^]^ Similarly, an arsanyl‐substituted gallaphosphene was shown to react with *t*BuN_3_ in a [3+2] cyclization to give a GaPN_3_ heterocycle.^[^ ^36^ ^]^

In 2021, we synthesized the first examples of isolable pnictaalumenes ^Ar^TerPn═AlCp* (Pn  =  P, As; Ter  =  2,6‐Ar_2_C_6_H_3_; Ar  =  Dipp, 2,6‐*i*Pr_2_C_6_H_3_; Tipp, 2,4,6‐*i*Pr_3_C_6_H_2_) with a pnictogen–aluminum multiple bond.^[^ ^37^ ^]^ In these compounds, the *π* component of the Pn═Al bond is highly polarized toward the pnictogen center, rendering the Pn atom nucleophilic, whereas the aluminum atom carries a net positive charge.^[^ ^38^ ^]^ Reactions with alkynes (*cf*. tolan) or alkenes (*cf*. styrene), ^Ar^TerPn═AlCp* revealed typical *π* bond behavior and [2+2] cycloadditions were observed. Depending on the specific reactants, the initial [2+2] addition products can undergo further reaction with a second or third equivalent of the C─C multiple bond system to form six‐membered rings or barrelene‐type cage structures.^[^ ^39^ ^]^ Given the *π* reactivity of ^Ar^TerPn═AlCp*, its potential for [3+2] cycloadditions with azides is enticing. Alternatively, the formation of insertion products is also plausible due to the highly polarized nature of the Pn═Al bond and the steric constraints imposed by the bulky ^Dipp^Ter substituent.

Herein, we outline diverse reactivity patterns of pnictaalumenes (Pn  =  P, As) with azides and a related diazoalkane (Scheme 1c). Structural motifs obtained from the reaction with azides include four‐membered AlN_2_Pn ring systems, one of which is containing a N_3_PnN_3_ chain unit. A similar heterocycle is also initially formed upon reaction of ^Dipp^TerPn═AlCp* with Me_3_Si(H)C═N_2_. Upon heating, this the P congener undergoes two consecutive rearrangements to terminal Al─CN complexes and an unusual NNN‐pincer ligand by stepwise deconstruction of the diazoalkane moiety is formed. The synthetic work is supplemented by theoretical studies to elucidate the bonding situation in these novel heterocyclic motifs.

## Results and Discussion

### Reactivity of ^Dipp^TerPn═AlCp* Toward Aryl Azides

**Scheme 2:**
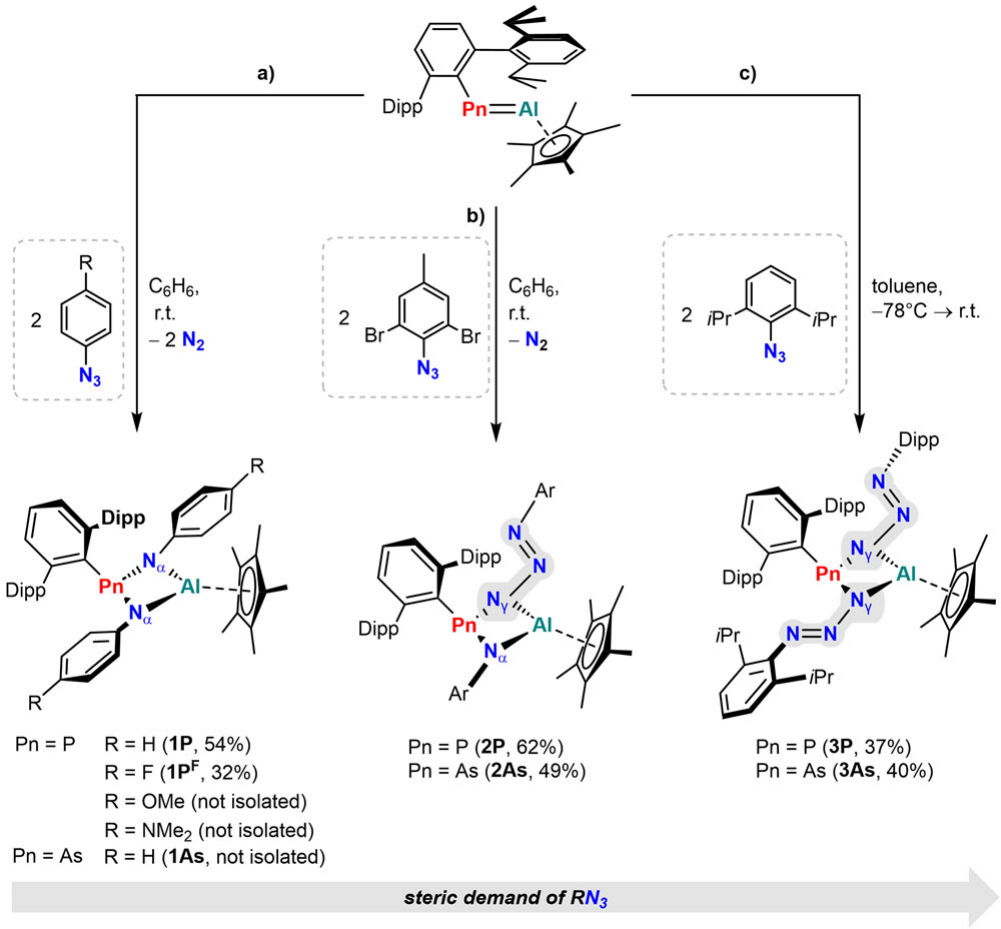
Synthesis of the four‐membered ring systems **1Pn**, **2Pn**, and **3Pn** by reaction of ^Dipp^TerPn═AlCp* with a) 4‐R‐C_6_H_4_‐N_3_, b) 2‐azido‐1,3‐dibromo‐5‐methylbenzene, and c) DippN_3_.

Considering the anticipated cycloaddition reactivity, ^Dipp^TerP═AlCp* was initially treated with one equivalent of PhN_3_. Monitoring the reaction by ^31^P{^1^H} NMR spectroscopy showed the formation of new species resonating at 84.2 ppm besides unreacted ^Dipp^TerP═AlCp* in a 1:1 ratio. Addition of a second equivalent of PhN_3_ resulted in full conversion of ^Dipp^TerP═AlCp*, giving this new species selectively. SCXRD analysis^[^ ^40^ ^]^ revealed the product as the four‐membered 1,3‐diaza‐2‐phospha‐4‐aluminatidine ^Dipp^TerP(*μ*‐NPh)_2_AlCp* (**1P**) (Scheme 2a), formed via twofold formal nitrene N_α_ insertion into the P═Al bond and concomitant N_2_ release. This reactivity can also be understood as a twofold Staudinger reaction at the P atom, therefore relating the formal [(PhN)_2_P^Dipp^Ter] unit to bis(imino)phosphoranes.^[^ ^41^ ^]^ The double N*_α_* insertion appears irrespective of the para substituent of the aryl azide. Analogous reactions of ^Dipp^TerP═AlCp* with 2 equivalents. *p*‐R‐C_6_H_4_‐N_3_ (R  =  F, OMe, NMe_2_) also afforded the corresponding N*_α_* insertion products as the major species according to ^31^P{^1^H} NMR spectroscopy (see pp. S149 ff.). Overall, the reactions with *para* substituted aryl azides were less selective, hampering efforts to isolate pure materials, which was only achieved for **1P^F^** in low yields of 33%. Additionally, the heavier homologous ^Dipp^TerAs═AlCp* was treated with two equivalents PhN_3_. Although the desired N*_α_* insertion product (**1As**) was identified as the major species by ^1^H NMR spectroscopy through comparison with **1P** (C*H*_3_^Cp*^ singlet at 1.61 ppm (**1As**) versus 1.57 ppm (**1P**); C*H* ^Dipp^ septet at 2.97 ppm (**1As**) versus 3.01 ppm (**1P**)), all attempts to isolate crystalline material resulted in unselective decomposition.

One decomposition product was crystallographically identified as the diaminoarsane ^Dipp^TerAs(NHPh)_2_ (see Figure S16), which is in line with hydrolysis of putative **1As** in the presence of trace amounts of water and formation of (Cp*AlO)_n_ as a byproduct. This suggests that the diaminopnictanes RPn(NHAr)_2_ might be accessible through the intentional hydrolysis of **1Pn**.

When ^Dipp^TerP═AlCp* was treated with the sterically more demanding *ortho*‐dibromo‐substituted *para*‐tolyl‐azide in a 1:2 ratio, the selective formation of an asymmetric product was indicated by NMR spectroscopy (Scheme 2b). The ^31^P{^1^H} NMR spectrum of the reaction mixture shows a resonance at 77.9 ppm, whereas in the ^1^H NMR spectrum, four signals for the methine protons of the Dipp groups and two singlets for the para‐Me groups of the azide were detected. This species, **2P**, was later revealed by SCXRD analysis (vide infra) as the mixed N*_α_*/N*_γ_‐*insertion product. A similar ^1^H NMR spectrum was obtained when using ^Dipp^TerAs═AlCp* as starting material, confirming the formation of **2As**. To the best of our knowledge, compounds **2P** and **2As** are the first examples of simultaneous N*_γ_* and nitrene N*_α_* insertion into element–element multiple bonds. Of note is a report on the reactivity of a diimine supported digallane, with a Ga─Ga single bond, toward AdN_3_, giving a related asymmetric LGa(*μ*‐NAd)(*μ*‐N_3_Ad)GaL species (L  =  (1,2‐DippNC)_2_C_10_H_6_).^[^ ^42^ ^]^ Related P(N_3_R)Al motifs have been reported by Uhl and coworkers using P/Al‐FLPs in the reaction with azides.^[^ ^43^ ^]^ The groups of Bourissou^[^ ^44^ ^]^ and Rivard^[^ ^45^ ^]^ reported reactions of P/B‐FLPs (P/B  =  *i*Pr_2_P(C_6_H_4_)BCy_2_; Cy = *cyclo‐*hexyl) to the corresponding phosphazide derivatives, which show P(N_3_R)B units.

A third class of ring systems was obtained when the sterically even more hindered DippN_3_ was reacted with ^Dipp^TerPn═AlCp* (Pn  =  P, As) (Scheme 2c). In both cases no gas evolution was observed, though the color of the pnictaalumene rapidly faded when the reaction mixtures were warmed from −78 °C to ambient temperature. Subsequent workup afforded the double N*_γ_*‐insertion products ^Dipp^TerPn(*μ*‐N_3_Dipp)_2_AlCp* (**3P** and **3As**) in moderate isolated yields after recrystallization. Twofold N_γ_ insertions of azides into E═E’ bonds have not been reported to date. Compared to **1P** and **2P**, **3P** shows a shielded ^31^P{^1^H} NMR resonance at 57.1 ppm. In the ^1^H NMR spectrum of **3P** and **3As**, all four Dipp groups are magnetically inequivalent, as evidenced by four partially overlapping resonances for the methine protons, indicating *C*_s_ symmetry due to hindered rotation in solution. In both compounds, a singlet resonance is observed for the Cp* groups (**3P** 1.78 ppm; **3As** 1.79 ppm), which is in line with **1Pn** and **2Pn** and an *η* ^5^‐coordination to the aluminum atom. Using AtomAccess,^[^ ^46^ ^]^ the accessibility of the N*_α_* and N*_γ_* atoms in the three employed azides was visualized and revealed the N_α_ atom in PhN_3_ as the most accessible, whereas as expected, the N*_α_* atom in DippN_3_ is the most sterically shielded one. This is in line with facile N_2_ elimination when PhN_3_ is utilized, whereas the Dipp substituent kinetically suppresses N_2_ elimination at ambient temperature. However, samples of **2P** and **3P** were thermally labile when heated to 80 °C over a period of 6 h, as indicated by selective formation of a new major species in the ^31^P{^1^H} NMR spectra (*δ*(^31^P)  =  101.0 ppm (**2P**); 102.1 ppm (**3P**)). It can be reasoned that these are the corresponding N*_α_* insertion products based on calculated ^31^P NMR shifts (Tables S9 and S10). This is furthermore in line with significant interaction between the P and N_α_ atoms when inspecting the Kohn–Sham orbitals (vide infra). Considering the facile formation of **3P** and its putative conversion to the N_α_ insertion product upon heating, we conclude that the initial step in all cases is the N_γ_ insertion, followed by intramolecular N_2_ elimination to give the double N_α_ insertion products. In addition, we investigated whether insertion of two different azides is feasible to yield analogous ring systems with two different substituents. If ^Dipp^TerP═AlCp* is combined with a DippN_3_ and 2,6‐Br_2_‐Tol‐N_3_ in a 1:1:1 ratio in toluene‐d_8_ at −78 °C, the formation of a mixture of products with ^31^P{^1^H} NMR signals at 77.7 (**2P**), 60.8, and 57.0 ppm (**3P**), respectively, is detected. This new species at 60.8 ppm corresponds to the mixed N_γ_ insertion product **3P_mixed** (Figure S7), which was confirmed by SCXRD experiments. Separation of the products from the reaction mixture by fractional crystallization was not possible.

In additional experiments, more azides were tested, which all resulted in unselective reactions with various ^31^P NMR resonances (pp. S149 ff.). In case of the reaction of ^Dipp^TerP═AlCp* with MesN_3_, one of the reaction products was identified by SCXRD experiments as a threefold addition/insertion product with C─H activation of one of the mesityl Me groups (compound **7P**, Figure S15). It can thus be concluded that the reactivity of pnictaalumenes of the type ^Dipp^TerPn═AlCp* toward aryl azides is greatly influenced by the steric profile of the respective azides, whereas the pnictogen atom plays no decisive role in the outcome of the reactions. The fact that [3+2] cyclization reactions are not observed is attributed to steric overcrowding of the P atom. The initial step of a [3+2] pathway would be nucleophilic attack of the N_γ_ atom at the Al atom, whereas the Pn center would then bond the N_α_ atom resulting in a steric clash between the ^Dipp^Ter substituent and the R group on the azide, even for smaller azides like Me_3_SiN_3_.

### Alternative Access to 1Pn

**Scheme 3:**
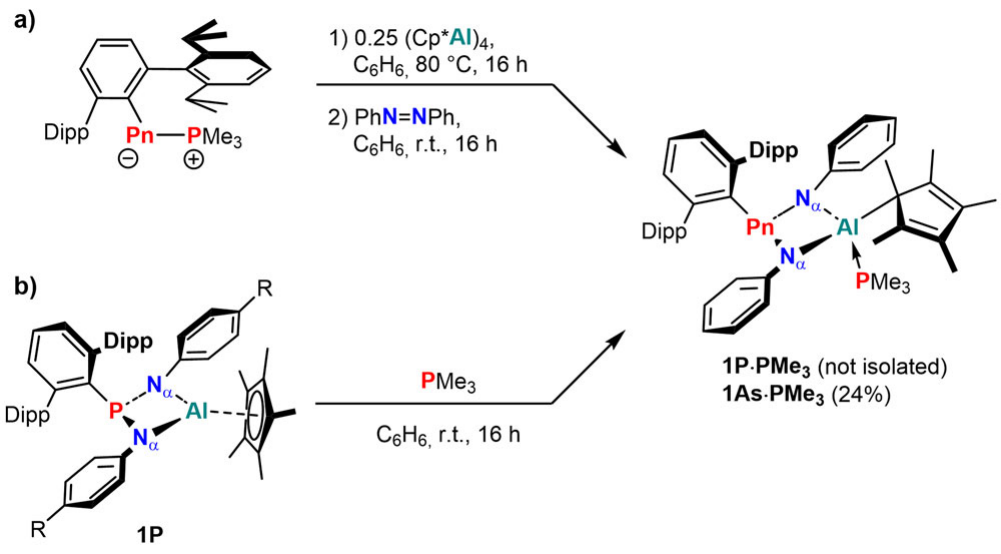
Formation of PMe_3_ adducts **1Pn·PMe_3_** from a) in situ generated pnictaalumene in presence of PMe_3_ and b) direct reaction of **1P** with PMe_3_.

Interestingly, compound **1P** was also obtained when ^Dipp^TerP═AlCp* was treated with *E*‐azobenzene in a 1:1 ratio. A [2+2] cycloaddition product was not detected in solution even at low temperatures. The same reaction with isolated *Z*‐azobenzene^[^ ^47^ ^]^ gave identical results, despite the N═N bond being more accessible for a [2+2] cycloaddition in the *Z* isomer. Even though ^31^P{^1^H} and ^1^H NMR spectroscopy unambiguously confirmed the formation of **1P** as the major product in both cases, isolation proved difficult. Considering the high polarization of the Pn═Al bond in ^Dipp^TerPn═AlCp*, the N═N bond scission resembles recently reported reactivities of FLPs (P/Sc and P/Si).^[^ ^48^, ^49^ ^]^ When *E*‐azobenzene was added to a freshly prepared sample of ^Dipp^TerP═AlCp* without prior removal of PMe_3_, the ^31^P{^1^H} NMR spectrum of the reaction mixture showed two broad resonances at 108.5 and −47.6 ppm (Scheme 3a). SCXRD experiments revealed the formation of **1P·PMe_3_** through Al···PMe_3_ adduct formation. Although **1P·PMe_3_** could not be isolated, the analogous reaction with in situ generated ^Dipp^TerAs═AlCp* afforded **1As·PMe_3_**, which was isolated in low yields of 24%. Compound **1As·PMe_3_** serves as an indirect structural confirmation of **1As** (see Scheme 2a) and exhibits a characteristic doublet at 0.30 ppm in the ^1^H NMR spectrum for the PMe_3_ group (*δ*(^31^P{^1^H})  =  −49.7 ppm), as well as a singlet at 1.81 ppm for the *η* ^1^‐Cp* group, indicating fast haptotropic rearrangement in solution.

Attempts to isolate **1P·PMe_3_** from the direct treatment of **1P** with PMe_3_ were likewise not successful, though its formation was confirmed by NMR spectroscopy (Scheme 3b).

### Solid‐State Structures of 1P, 1As·PMe_3_, 2Pn, and 3Pn

**Figure 1:**
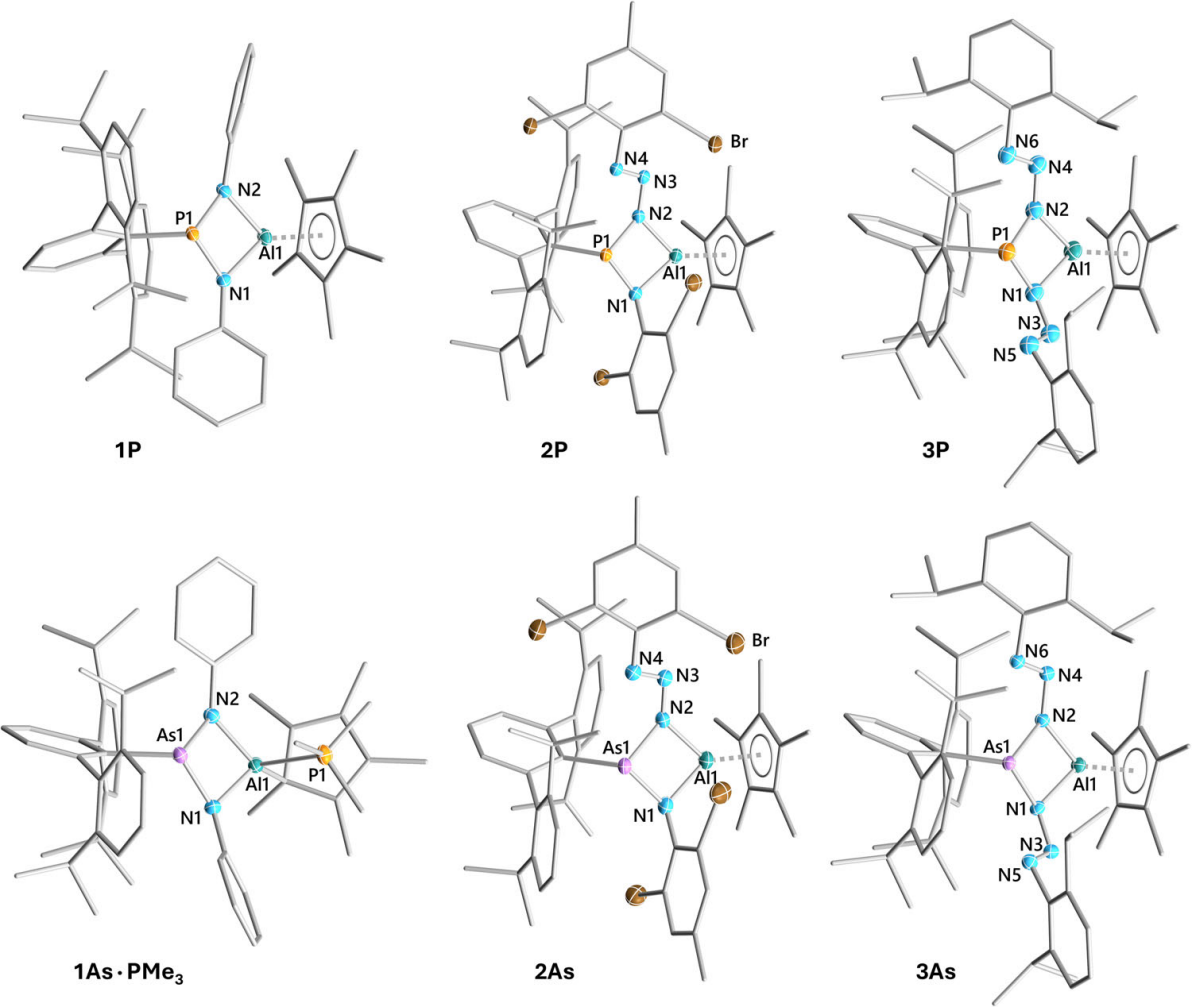
Molecular structures of **1P**, **1As·PMe_3_**, **2Pn**, and **3Pn**. Ellipsoids are set at 50% probability (100(2) K or 150(2) K for **1As·PMe_3_**, **2As**, **3As**) with H atoms omitted. Carbon atoms rendered as wireframe for clarity. For selected bond lengths (Å) and angles (°) see Table 1 and Supporting Information.

The molecular structures in the solid state of **1P**, **1Pn·PMe_3_**, **2Pn**, and **3Pn** were elucidated by SCXRD experiments (Figure 1). Selected structural parameters are provided in Table 1 (for full bond data see Supporting Information). All phosphorus compounds **1P**–**3P** feature kite‐shaped central AlN_2_Pn rings, with the nearly equidistant P─N bonds being substantially shorter than the N─Al bonds. Along with an increasing steric demand of the N‐aryl groups, the AlN_2_Pn rings adopt a slightly butterfly‐like geometry, as evident in increasing dihedral P1─N1─Al1─N2 angles from **1P** (6.63(6)°) to **3P** (10.49(7)°). Following this trend, the P─N bonds also widen when going from **1P** to **3P**, whereas the Al─N distances remain mostly constant. Notably, all P─N and Al─N bonds in **1P**–**3P** are considerably shorter than the sum of their covalent radii (*cf*. *Σr*_cov_(P─N)  =  1.82 Å;^[^ ^50^ ^]^ *Σr*_cov_(Al─N)  =  1.97 Å^[^ ^50^ ^]^), which is likely due to their incorporation in a highly strained four‐membered ring system. Likewise, the short Al1─P1 distances in **1P**–**3P** (2.63–2.75 Å, *cf. Σr*_vdW_(P─Al)  =  3.64 Å^[^ ^51^ ^]^) are also a result of ring strain rather than from transannular bonding as both centers are coordinatively saturated. This coordinative saturation is provided by the *η* ^5^‐coordinating Cp* ligands at the Al1 atoms, as evidenced by nearly equal Al1─C^Cp^ distances. The P1 atoms in **1P**–**3P** are in a trigonal pyramidal coordination environment, indicating the presence of a lone pair of electrons (LP), whereas the ^Dipp^Ter substituent is in all cases oriented away from the N‐aryl moieties to compensate for steric strain. Similarly, for some of the N atoms incorporated in the four‐membered rings, a distorted trigonal planar coordination environment due to the steric overload is noted (*cf*. **3P** *Σ*∢(N1)  =  339.0(4)).

**Table 1:** Selected bond lengths and angles of **1P**, **1Pn·PMe_3_**, **2Pn**, and **3Pn**.

Phosphorus compounds				
	1P	1P·PMe_3_	2P	3P
P1─Al1 (Å)	2.6333(5)	2.6496(7)	2.6926(9)	2.7448(7)
P1─N1/P1─N2 (Å)	1.717(1)/1.722(1)	1.737(1)/1.727(1)	1.754(2)/1.760(2)	1.769(2)/1.776(2)
Al1─N1/Al1─N2 (Å)	1.851(1)/1.839(1)	1.873(1)/1.856(1)	1.848(2)/1.853(2)	1.846(2)/1.855(2)
P1─N1─Al1─N2 (°)	6.23(5)	14.67(6)	7.8(1)	10.49(7)
Σ∢(P1) (°)	305.3(2)	307.6(3)	303.9(3)	296.0(3)
N*_α_* ═N*_β_* (Å)			1.276(3)	1.274(2)/1.267(2)
N*_β_* ─N*_γ_* (Å)			1.336(3)	1.348(2)/1.359(2)
N*_α_* ═N*_β_* ─N*_γ_* ─P (°)				19.7(2)/23.9(2)

Arsenic compounds				
	1As	1As·PMe_3_	2As	3As
As1─Al1 (Å)		2.7584(4)	2.8117(9)	2.8663(4)
As1─N/1As1─N2 (Å)		1.874(1)/1.886(1)	1.907(2)/1.913(2)	1.920(1)/1.931(1)
Al1─N1/Al1─N2 (Å)		1.852(1)/1.866(1)	1.855(3)/1.838(3)	1.840(1)/1.852(1)
As1─N1─Al1─N2 (°)		14.5(5)	8.2(1)	11.23(4)
Σ∢(As1) (°)		302.0(2)	297.7(3)	289.0(2)
N*_α_* ═N*_β_* (Å)			1.278(4)	1.271(2)/1.268(2)
N*_β_* ─N*_γ_* (Å)			1.327(3)	1.342(2)/1.348(2)
N*_α_* ═N*_β_* ─N*_γ_* ─As (°)				18.4(1)/21.6(1)

Unlike **1P**, compounds **2P** and **3P** feature intact N_3_ chains. The N_γ_═N_β_ and N_β_─N_α_ distances correspond to elongated double and contracted single bonds (Σ*r*_cov_(N─N)  =  1.42 Å); (N═N)  =  1.20 Å),^[^ ^50^ ^]^ respectively, indicating a certain degree of *π*‐electron delocalization. These *E*‐configured triazene units in **2P** and **3P** are akin to recent reports on geminal Al/P^[^ ^43^ ^]^ and Zr/P^[^ ^52^ ^]^ FLPs. The same trends are also observed for both the arsenic compounds **2As** and **3As**. Interestingly, the torsion angles Pn─N1─N3─N5 (P: 23.9(2); As: 21.6(1)°) and Pn─N2─N4─N6 (P: 19.7(2); As: 18.4(1)°) in **3Pn** show an almost coplanar arrangement of both N_3_‐chains, indicating conjugation along the N_3_PnN_3_ chain. The corresponding PMe_3_ adduct of **1P**, **1P·PMe_3_**, features similar bond data when compared to **1P**. The system adjusts to the higher coordination at the Al atom (coordination number  =  4) by the AlN_2_Pn ring (∢(Pn1─N1─Al1─N2) ≈14.5°) adopting a more pronounced butterfly conformation and by the Cp* substituent shifting to *η* ^1^‐coordination mode. Concomitantly, the Al─N distances are significantly elongated compared to **1P**.

### Reactivity of ^Dipp^TerPn═AlCp* Toward a Diazomethane Derivative

**Scheme 4:**
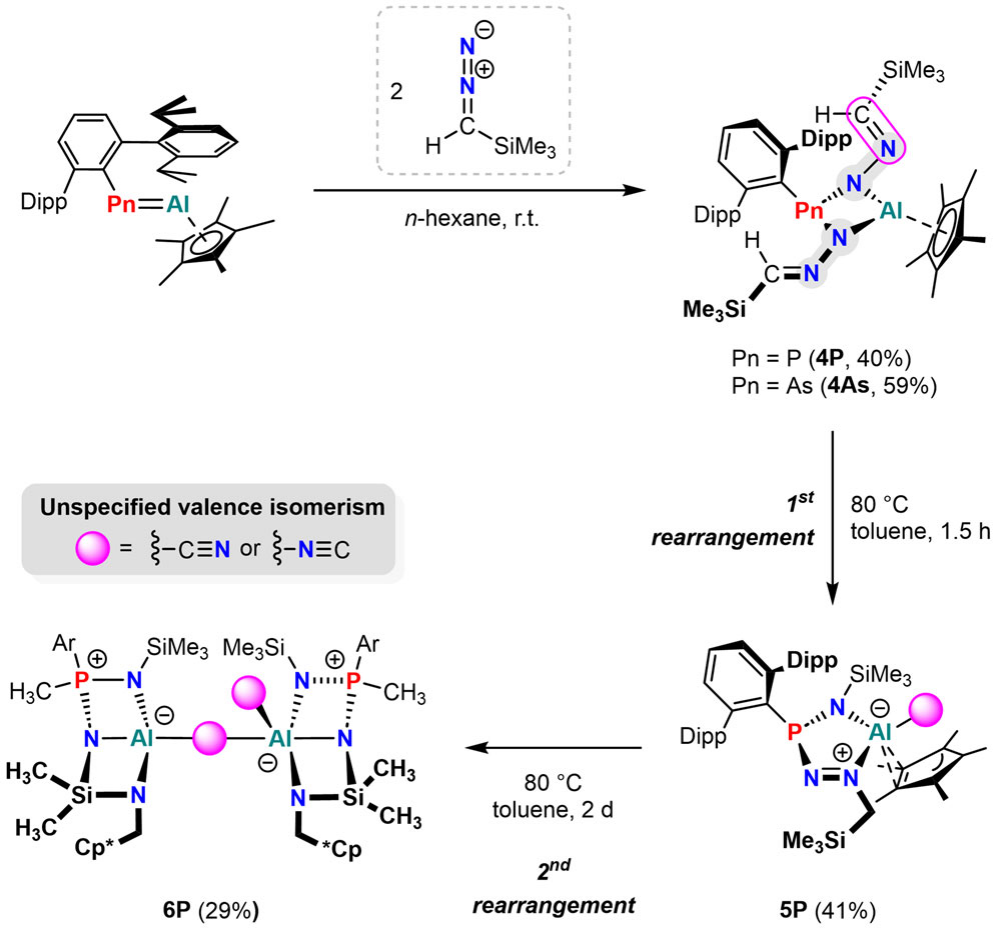
Reactivity of pnictaalumenes toward Me_3_SiC(H)N_2_ and thermal rearrangements of **4P** to **5P** and **6P**, respectively.

Intrigued by the unexpectedly rich structural diversity in reactions of ^Dipp^TerPn═AlCp* with aryl azides, the reactivity scope was extended to diazoalkanes as a different class of 1,3‐dipole molecules. As diazoalkanes are isoelectronic congeners of azides and can also act as carbene transfer reagents,^[^ ^5^, ^9^, ^10^ ^]^ we expected a [2+1] cycloaddition to the corresponding alumina–pnictirinanes, similar to reports for analogous reactions of diphosphenes^[^ ^53^ ^]^ and distibenes.^[^ ^54^ ^]^ Hence, ^Dipp^TerPn═AlCp* was reacted with equimolar amounts of the diazomethane Me_3_Si(H)C═N_2_ (Scheme 4).

In both cases, a ^29^Si{^1^H} NMR resonance at −9.9 ppm indicated the formation of identical products, independent of the pnictogen atom. Again, the ^31^P{^1^H} NMR spectrum revealed unreacted starting material alongside a new resonance at 46.8 ppm (cf. **3P** 57.1 ppm) in a 1:1 ratio in the case of Pn═P. Full conversion was only achieved upon addition of a second equivalent of Me_3_Si(H)C═N_2_. SCXRD analysis (vide infra) revealed the products as the twofold insertion of the terminal diazomethane N atoms, yielding the four‐membered, imine‐substituted AlN_2_Pn ring systems, **4P** and **4As**, akin to the azide insertion products **3Pn** (vide supra). In solution, **4P** and **4As** show characteristic ^1^H NMR signals for the aldimine C(*H*)SiMe_3_ protons (*δ*(^1^H, **4P**)  =  6.88 ppm, *δ*(^1^H, **4As**) = 6.78 ppm), along with two sets of signals for the Dipp groups of the ^Dipp^Ter moiety, indicative of hindered rotation about the P─C_Ar_ axis.

Next, we tested whether thermal stress would induce N_2_ liberation from **4Pn**. Although **4As** is thermally stable, even when heated to 80 °C over a period of 24 h, solutions of **4P** changed their color from pale yellow to red after heating for 1.5 h. SCXRD analysis (vide infra) revealed the product after heating to be five‐membered ring species **5P** (major species, >95% by ^31^P{^1^H} NMR spectroscopy). This thermally induced rearrangement results in the deconstruction of one of the diazomethane units and the formation of an aluminate–cyanide complex. The remaining N‐SiMe_3_ fragment μ‐bridges the P and Al atoms. Additionally, a ring expansion occurred through N insertion of the second diazomethane unit, accompanied by a hydrogen shift. Due to the ambidentate nature of the cyanide ligand, it is not possible to undoubtedly confirm the binding mode of CN^−^ in **5P**. Although ^13^C{^1^H} NMR spectroscopy is generally useful to elucidate the binding mode, no ^13^CN^−^ resonance was observed due to quadrupolar broadening by the aluminum nucleus.^[^ ^55^ ^]^ However, in the ^31^P{^1^H} NMR spectrum, a major product at 137.5 ppm was detected, along with a minor species at 136.8 ppm in a 8:2 ratio. We assign them to the two valence isomers Al─CN and Al─NC, which give minimally different ^31^P NMR signals according to quantum chemical studies (Table 3). In the infrared spectrum of **5P**, a weak vibration mode at 2261 cm^−1^ is within the range reported for [E^13^─CN]^−^ complexes (*cf. ṽ*([(CF_3_)B─CN]^−^)  =  2244 cm^−1^).^[^ ^56^ ^]^

Computational studies (see p. S159 ff.) found the rearrangement from **4P** to **5P** to be strongly exergonic (*Δ*_R_ *G*°_298_  =  −212.22 kJ·mol^−1^) and hence in line with a facile reaction, even though the underlying reaction mechanism could not yet be determined due to the complexity of the system. During the synthesis of **5P**, we observed a second thermal rearrangement of **5P** to **6P** when the reaction time was extended to two days. The ^1^H NMR spectrum of this second rearrangement product inferred a high molecular complexity, with a doublet resonance at 1.12 ppm (^2^ *J*_PH_  =  13.7 Hz), indicating a methyl transfer from one of the Me_3_Si groups to the phosphorus center. Consequently, the ^1^H NMR spectra now also showed a new singlet resonance for a SiMe_2_ group, as well as one remaining SiMe_3_ group. Furthermore, the spectra revealed four signals for *η* ^1^‐bound Cp* substituent. The absolute constitution of **6P** was revealed by SCXRD analysis (vide infra) and is in line with the ^1^H NMR spectral data, as well as a ^31^P{^1^H} NMR resonance at 38.7 ppm in the typical region of a four‐coordinate phosphorus atom. Compound **6P** is the formal insertion product of one of the SiMe_3_ groups into the N═N bond of **5P**, whereas one of the Me groups is shifting onto the phosphorous atom and the Cp*‐ligand is migrating to the methylene group in favor of a new N─Al bond. One decomposition product of **6P**, which was obtained by crystal picking, is the formal HCN addition product **6P^H^**, featuring a protonated amido arm and a dicyanoaluminate structural motif (see Figure S14). Generally, Al‐CN/NC species are scarce. Heilmann and coworkers recently reported a dimeric and anionic Al–cyanide,^[^ ^57^ ^]^ complexed by a NON‐ligand, similar to the formal trisamido NNN‐pincer‐type ligand in **6P**. Apart from this, such a ligand architecture is reminiscent of the work by Berben and coworkers on redox‐noninnocent tridentate bis(imino)pyridine ligands coordinating an Al^III^ center.^[^ ^58^ ^]^

### Solid‐State Structures of 4Pn, 5P, and 6P

**Figure 2:**
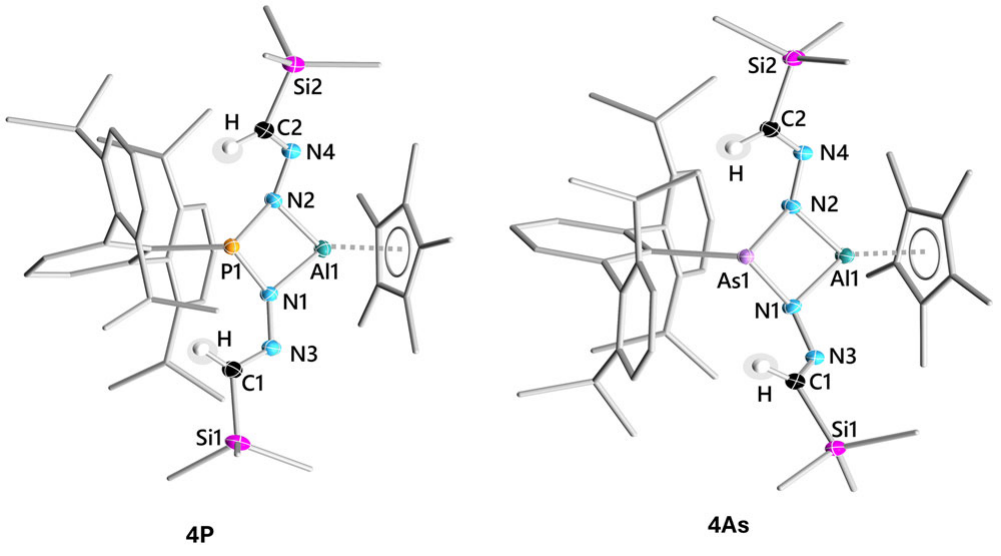
Molecular structures of **4P** (left) and **4As** (right). Ellipsoids are set at 50% probability (100(2) K) with H atoms omitted (except on C1 and C2). ^Dipp^Ter, Cp*, and SiMe_3_ substituents rendered as wireframe for clarity (except C1 and C2). For selected bond lengths (Å) and angles (°), see Table 2 and Supporting Information.

**Figure 3:**
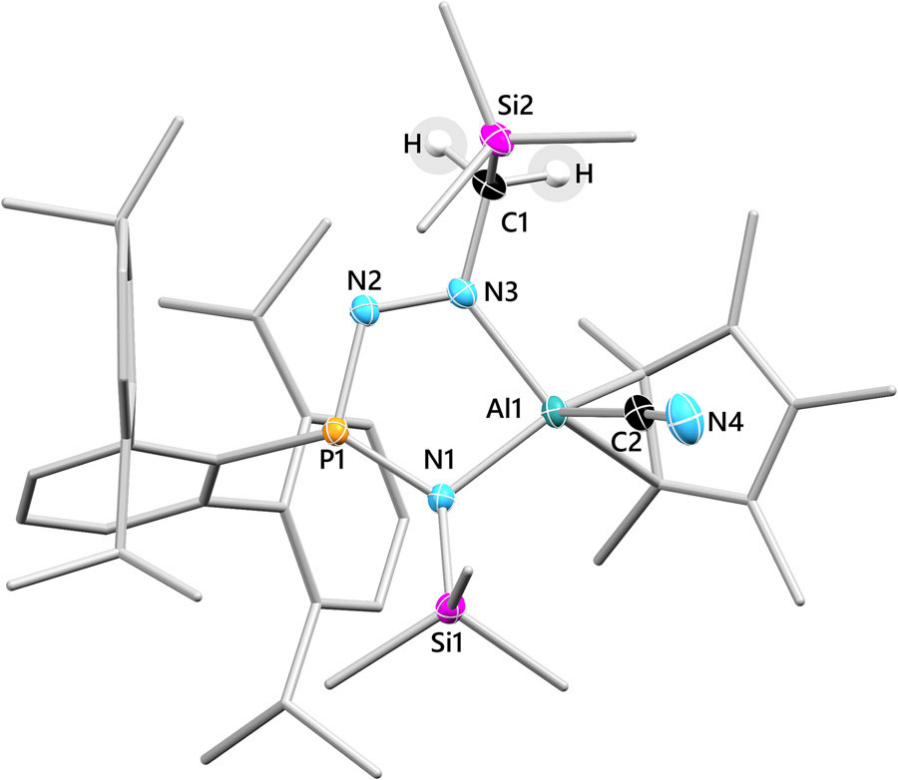
Molecular structure of **5P**. Ellipsoids are set at 50% probability (100(2) K) with H atoms omitted (except C1). C atoms of ^Dipp^Ter, Cp*, and SiMe_3_ substituents rendered as wireframe for clarity (except C1 and C2). Selected bond lengths (Å) and angles (°): P1─N1 1.673(1), P1─N2 1.678(1), Al1─N1 1.896(1), Al1─N3 1.981(1), N2─N3 1.293(2), N3─C1 1.468(2); Σ∢(P1) 330.2(2).

**Figure 4:**
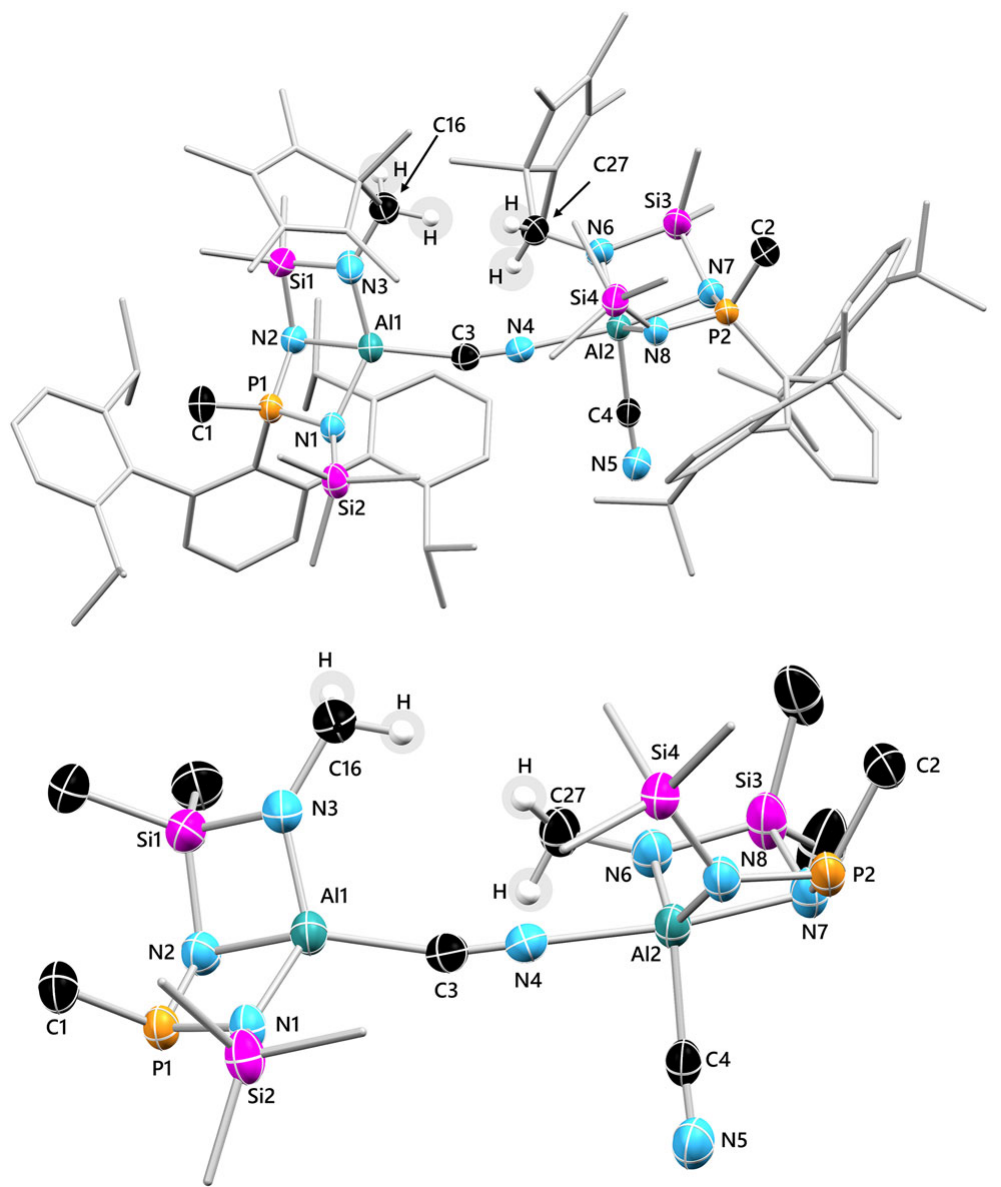
Top: Molecular structure of **6P**. Ellipsoids are set at 50% probability (100(2) K) with H atoms omitted (except C16 and C27). C atoms of ^Dipp^Ter, Cp*, and SiMe_3_ substituents rendered as wireframe for clarity (except C1, C2, C3, C4, C16, and C27). Bottom: NNN‐pincer cores with hidden organic periphery. Selected bond lengths (Å) and angles (°): P1─N1 1.651(2), P1─N2 1.606(2), P2─N8 1.639(2), P2─N7 1.571(2), Al1─N1 1.883(2), Al1─N3 1.802(2), Al2─N8 1.955(2), Al2─N6 1.842(2), C4─N5 1.160(3), Si1─N2 1.765(2), Si1─N3 1.728(2), Si1─N7 1.751(2), Si1─N6 1.721(2); P1─N1─Al1─N2 2.9(1), P2─N8─Al2─N7 9.0(1), N2─Al1─N3─Si1 1.0(1), N6─Al2─N7─Si2 4.4(1).

SCXRD experiments were performed on both compounds **4Pn** (Figure 2), as well as on **5P** (Figure 3) and **6P** (Figure 4). Selected structural parameters of **4Pn** are provided in Table 2 (for full bond data see Supporting Information). Similar to compounds **1Pn**–**3Pn**, both **4P** and **4As** feature kite‐shaped, slightly puckered AlN_2_Pn ring motifs with Pn1─N1─Al1─N2 angles (**4P**: 7.80(5), **4As**: 9.64(5)°), which are best compared to **2Pn** (**2P**: 7.8(1), **2As**: 8.2(1)°).

**Table 2:** Selected bond lengths and angles of **4Pn**.

	4P	4As
Pn1─Al1 (Å)	2.7121(5)	2.8390(4)
Pn1─N1/Pn1─N2 (Å)	1.753(1)/1.754(1)	1.907(1)/1.926(1)
Al1─N1/Al1─N2 (Å)	1.833(1)/1.849(1)	1.832(1)/1.8400(1)
Pn1─N1─Al1─N2 (°)	7.80(5)	9.64(5)
Σ∢(Pn1) (°)	298.2(2)	290.4(1)
C1═N3/C2═N4 (Å)	1.287(2)/1.291(2)	1.291(2)/1.287(2)
N1─N3/N2─N4 (Å)	1.380(2)/1.367(1)	1.358(1)/1.366(1)
C1═N3─N1─Pn (°) C2═N4─N2─Pn (°)	19.4(2) 20.1(2)	18.2(2) 16.1(2)

The pnictogen atoms in **4Pn** are again in a trigonal pyramidal coordination environment and the Pn─N and Al─N distances are considerably shorter than the sum of their covalent radii (*cf. Σr*_cov_(P─N) = 1.82 Å;^[^ ^50^ ^]^ *Σr*_cov_(As─N)  =  1.92 Å;^[^ ^50^ ^]^ *Σr*_cov_(Al─N)  =  1.97 Å^[^ ^50^ ^]^). Notably, the Me_3_Si(H)CN_2_ units exhibit minimally elongated C═N aldimine double bonds (*Σr*_cov_(C═N)  =  1.27 Å^[^ ^50^ ^]^) and contracted N─N bonds (*Σr*_cov_(N─N)  =  1.42 Å, *Σr*_cov_(N═N)  =  1.20 Å),^[^ ^50^ ^]^ which indicates conjugation in the P(N_2_CHR)_2_ unit. The Cp* ligand is *η* ^5^‐coordinated to Al1 with nearly equidistant Al1─C^Cp^ bonds. The five‐membered AlN_2_PN ring in **5P** is nearly planar, as evident from the small deviation of the atoms from the least‐square plane (root‐mean‐square = 0.0008(5) to 0.1148(7) Å).

All heterodiatomic bonds, as well as the N═N bond within the ring motif are shorter than the sum of the respective single bond covalent radii but elongated compared to **1P**–**4P**, either due to the reduced ring strain or the absence of mesomeric effects. The P atom is in a trigonal pyramidal coordination environment, whereas the Al atom is now four‐coordinate due to the CN^−^ substitution, enforcing a shift of the Cp* ligand from *η* ^5^ to *η* ^1^ in **5P**. Refinement of **5P** as its Al─CN valence isomer shows a better fit than the Al─NC isomer for the selected single crystal. However, no isomer ratio could be obtained from SCXRD data (vide supra). Although the terminal CN unit in **6P** is also best refined as an Al─CN unit, the bridging CN^−^ shows CN/NC isomerization disorder, which permits the discussion of related bond parameters. Both of the NNN‐pincer fragments comprise a butterfly‐shape, which is slightly more pronounced in the pincer motif with the five‐coordinate Al atom (47.4(8)° tilted versus 50.5(8)° tilted). The Al atoms are in a distorted tetrahedral (left fragment, Figure 4) and trigonal‐bipyramidal (right fragment, Figure 4) coordination environment, respectively, whereas both P atoms are tetrahedrally coordinated due to the methyl‐shift.

Again, the heterodiatomic bonds within the pincer motif are shorter than the sum of their respective single bond covalent radii (*cf. Σr*_cov_(N─Al)  =  1.97 Å;^[^ ^50^ ^]^ *Σr*_cov_(N═Al)  =  1.73 Å,^[^ ^50^ ^]^ *Σr*_cov_(N─Si)  =  1.87 Å;^[^ ^50^ ^]^ *Σr*_cov_(N═Si)  =  1.67 Å^[^ ^50^ ^]^). Interestingly, the short N─P distances are reminiscent of those in reported iminophosphonamido complexes, suggesting a similar bonding situation in **6P**.^[^ ^59^, ^60^ ^]^

### DFT Calculations

Theoretical studies of **1Pn**–**4Pn**, as well as **5P** and **6P** were performed and the geometries were optimized at the PBE0^[^ ^61^, ^62^, ^63^ ^]^‐D3^[^ ^64^, ^65^ ^]^/def2‐SVP^[^ ^66^ ^]^ level of theory and the gas‐phase structures showed sufficient agreement with the experimentally determined molecular structures in the solid state. Preceding the bonding analysis, we determined the gas phase ^31^P NMR shifts of all compounds **1P**–**6P** using the GIAO method^[^ ^67^, ^68^, ^69^, ^70^, ^71^ ^]^ and a double reference system^[^ ^72^ ^]^ to assess whether this method can serve as a reliable tool to predict the structure of such systems in future studies. Overall, the GIAO‐predicted gas‐phase ^31^P NMR shifts were in good agreement with those experimentally determined for all four‐membered ring species **1P**–**4P** (Table 3). For the thermal rearrangement product of **4P** to **5P**, we also attempted to probe the coordination isomerism (**5P^CN^** versus **5P^NC^**) via the respective calculated ^31^P NMR shifts of the individual isomers. Although the obtained ^31^P NMR shifts are generally within the expected range, a clear distinction between **5P^CN^** and **5P^NC^** is not possible in this case.

**Table 3:** Comparison of experimental and gas‐phase ^31^P NMR shifts (PBE0‐D3/def2TZVP//PBE0‐D3/def2SVP).

	*δ*_exp._(^31^P) [ppm]	*δ*_calc._(^31^P) [ppm]
**1P**	84.2	80.0
**1P·PMe_3_**	108.5/−47.6	108.9/−43.6
**2P**	77.9	70.6
**3P**	57.1	58.3
**4P**	46.8	45.3
**5P^CN^**	137.5	147.8
**5P^NC^**	136.8	148.3
**6P**	39.0	43.6

**Figure 5:**
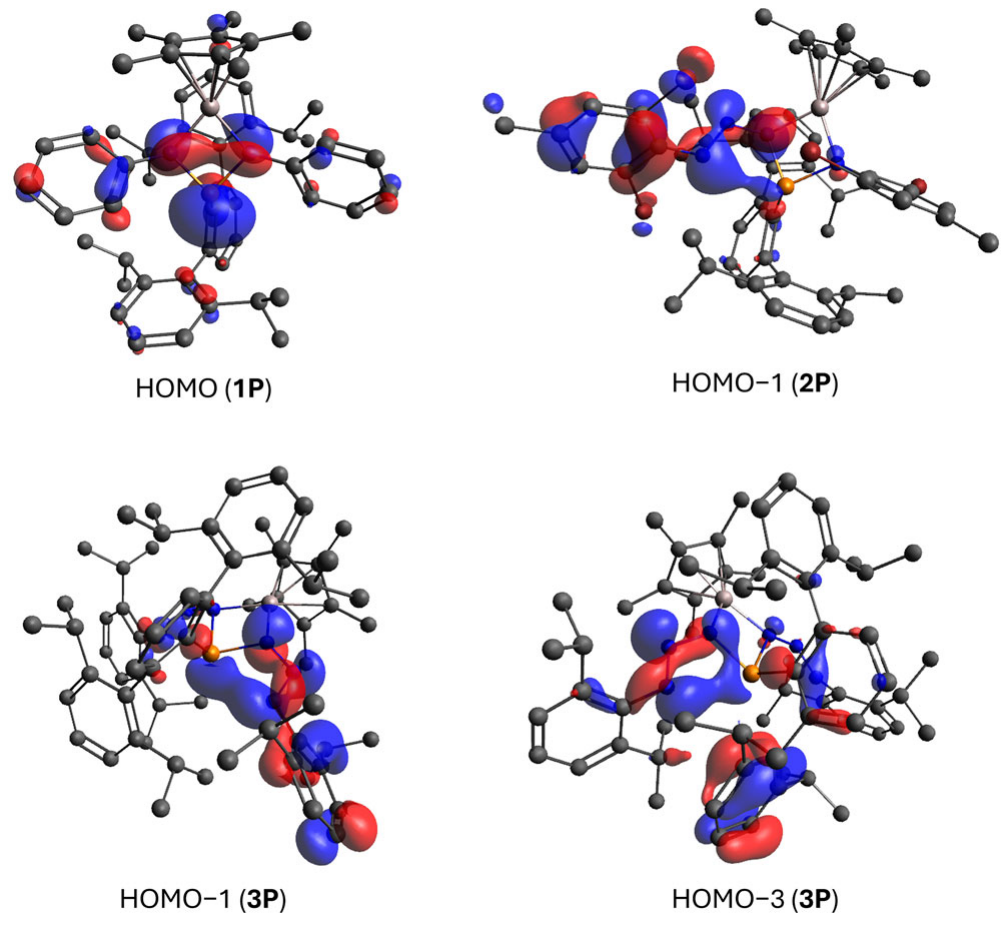
Selected Kohn–Sham orbitals of **1P** (top, left), **2P** (top, right), and **3P** (bottom) at the PBE0‐D3/def2‐TZVP level of theory.

An inspection of the Kohn–Sham orbitals and NBO analyses using the densities at the PBE0‐D3/def2‐TZVP level were carried out to elucidate the bonding in **1Pn**–**4Pn** (Figure 5). The bonding in those ring species seems to be best described by four covalent bonds within in the AlN_2_Pn rings. The HOMO in **1P** is best described as n‐type with contributions from a LP on the P atom with high s‐character and two p‐type LPs on the N atoms, which overlap through the four‐membered ring. The LUMO on the other hand is centered on the ^Dipp^Ter unit, whereas LUMO+1 and LUMO+2 have significant contribution from a p‐type lone valence (LV) on the Al atom (see Figure S115), in line with facile PMe_3_ coordination in **1P·PMe_3_**. The HOMO of **2P** is like that of **1P** with additional contribution of sp^2^‐type LPs on the intact azide unit. Interestingly, the HOMO−1 in **2P** reveals a weakly bonding character between P and the N_α_ of the intact azide unit, basically a snapshot of the four‐membered transition state that would lead to N_2_ liberation. Similar orbitals are observed for **3P** in HOMO−1 and HOMO−3 (Figure 5, bottom), which confirmed the conjugation in the intact azide fragment and support the experimental finding that both, **2P** and **3P**, undergo thermal N_2_ liberation when heated to 80 °C over a prolonged period.

NBO analysis^[^ ^73^, ^74^, ^75^, ^76^ ^]^ revealed surprisingly low Wiberg bond indices (WBI) for the Al─N bonds (WBI_Al−N_ < 0.4) for all AlN_2_Pn species, indicative of highly polarized bonds with a high ionic character. This is further corroborated by a high positive natural charge on the Al atoms of ca. +2 and accumulation of negative charge on the ring N atoms (Table S11). The P─N bonds are in the range of strongly polarized single bonds, with ca. 25% of the electron density at the Pn atoms and ca. 75% at the N atoms, respectively.

**Scheme 5:**
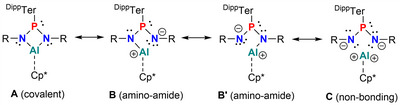
Resonance scheme of four‐membered AlN_2_P‐ring species.

**Figure 6:**
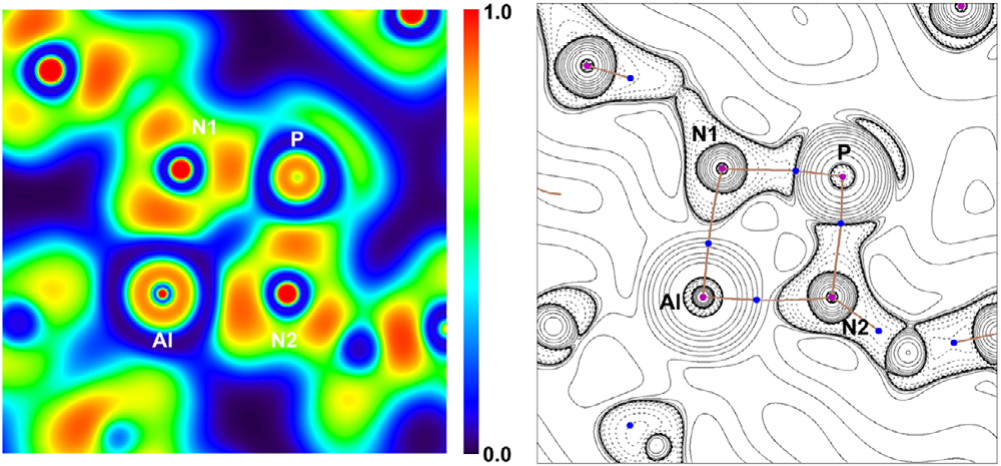
Depiction of the ELF (left) and contour‐line plot of the Laplacian of the electron density (right) of **1P** in the AlN_2_P‐plane.

According to NBO analyses, two LPs are present at each N atom in **1P**–**4P**, respectively, one of which is sp^2^ hybridized, whereas the other one exhibits predominantly p character. The sp^2^‐type LP is delocalized considerably into the LVs at the Al atom and thus best describes the highly polar Al─N bonds. Consequently, in the NBO picture, the bonding in all four‐membered rings is best described by at least four resonance forms: a covalent (**A**), two amino–amido forms (**B**, **B’**) and a nonbonding (**C**) form (Scheme 5). Especially **1P** can, therefore, be described as a dicationic Al(III) complex of a doubly reduced bis(imino)phosphorane. This description holds for all four‐membered species, however, the negative charge on the N_γ_ atoms in **2Pn**, **3Pn**, and **4Pn** is depleted, in line with delocalization of π‐electron density into the π* orbitals of the intact N_3_R or N_2_CR_2_ units, respectively. To further support the findings of the NBO analyses, the electron localization function (ELF) and the Laplacian of the electron density in the AlN_2_P plane were investigated.^[^ ^77^ ^]^ The results are discussed for **1P** only as these are similar for all rings independent of the pnictogen center. The ELF in the AlN_2_P plane in **1P** (Figure 6, left) clearly shows depletion of localized electron density between Al and N, along with signatures for the sp^2^‐type LPs on the N atoms as well as a LP on the P atom with high s character. Investigation of the Laplacian of the electron density in the same plane also clearly shows valence shell charge concentrations (VSCCs) at the N atoms and bond critical points that are shifted toward Al and P (Figure 6, right), respectively, in line with a high polarization of these bonds. The electron density at the bond critical points (BCP) of the Al─N paths is low (0.085 e·bohr^−3^) compared to the P─N bonds (0.16 e·bohr^−3^). The ellipticity of the electron density at the BCPs is identical for the Al─N and P─N BCPs (0.15).

The low electron densities at the Al─N BCPs are in line with highly polar covalent bonds, which is further supported by rather small, yet negative values of the Laplacian at the BCPs. Based on QTAIM analysis, highly polar bonds prevail in the four‐membered rings, which eventually should render them valuable synthons in transmetalation reactions as iminophosphonamido ligands.

## Conclusion

In summary, we have synthesized and characterized a series of 1,3‐diaza‐2‐pnicta‐4‐aluminatidines (^Dipp^TerPn(*µ*‐NR)_2_AlCp*) by aryl azide insertion into the Pn–Al double bond or by nitrene transfer from scission of azobenzene. Different insertion motifs (**1Pn**: N_α_/N_α_, **2Pn**: N_α_/N_γ_, **3Pn**: N_γ_/N_γ_) were obtained by varying the steric demand of the aryl substituent. DFT calculations revealed the highly polar covalent bonding situation, which should render **1Pn**–**4Pn** valuable synthons in transmetalation reactions. The N_3_PnN_3_‐chains in **3Pn** are the first examples of dianionic pnictabistriazenes. Using the related diazomethane derivative, Me_3_SiC(H)N_2_ yields a similar structural motif (**4Pn**), in which the *μ*‐bridging ring N atoms are imine‐substituted. In contrast to **2P** and **3P**, where heating led to N_2_ elimination, **4P** undergoes two complex rearrangements in an unprecedented intramolecular reaction of the diazomethane fragment. The first rearrangement generates the zwitterionic cyanido aluminate complex **5P** under deconstruction of the Me_3_SiC(H)N_2_ unit. Subsequently, in a second, more complex rearrangement, an NNN‐pincer cyanido aluminate complex **6P** is formed.

The highly polar Pn═Al multiple bonds in pnictaalumenes allows the synthesis of novel inorganic ring systems, that can otherwise not be assembled and the propensity of aluminum to react in salt metathesis reactions will render these valuable synthons in both main group and transition metal chemistry.

## Supporting Information

The authors have cited additional references within the Supporting Information.^[^ ^78^, ^79^, ^80^, ^81^, ^82^, ^83^, ^84^, ^85^, ^86^, ^87^, ^88^, ^89^, ^90^, ^91^, ^92^, ^93^, ^94^, ^95^, ^96^, ^97^, ^98^, ^99^, ^100^, ^101^, ^102^, ^103^ ^]^

## Conflict of Interests

The authors declare no conflict of interest.

## Supporting information



Supporting Information

Supporting Information

Supporting Information

## Data Availability

The data that support the findings of this study are available in the Supporting Information of this article.
