# Supplementary material for: Reactivity of Pnictaalumenes Towards 1,3‐Dipole Molecules
Source: Angew Chem Int Ed Engl. 2025 Jun 17;64(31):e202506356. doi: 10.1002/anie.202506356 (PMC12304809; doi:10.1002/anie.202506356)
Supplement: Supplementary file 1 — Supporting Information [file ANIE-64-e202506356-s003.pdf]

SUPPORTING INFORMATION

Reactivity of Pnictaalumenes towards 1,3-Dipole Molecules

Tim Wellnitz, Edgar Zander, Leonie Wüst, Jakob Boardman, Max Neubauer, Samuel Nees, Holger Braunschweig, Christian Hering-Junghans**

This file includes:

1 Experimental 2

2 Structure elucidation 6

3 Syntheses of starting materials 32

4. Syntheses of compounds 71

5. Computational details 153

6. References 170

1 Experimental

General Information. If not stated otherwise, all manipulations were carried out under oxygen- and moisture-free conditions in an inert argon atmosphere using standard Schlenk or drybox techniques. All glassware was heated three times *in vacuo* using a heat gun (650 °C) and cooled under argon atmosphere. Solvents were transferred using syringes, which were purged three times with argon prior to use or steel cannulas and PTFE cannulas, which were stored at 120 °C or 80 °C and purged three times with argon prior to use. Solvents and reactants were either obtained from commercial sources or synthesized as detailed in ***Table S1***.

Table S1. Origin and purification of solvents and reactants.

Substance	Origin	Purification
Benzene	Sigma Aldrich	Dried over Na/benzophenone, freshly distilled prior to use. Degassed (freeze-pump-thawn), stored and further dried over molecular sieves (4 Å)
Diethylether	≥99.9%, suitable for HPLC, inhibitor-free	Purified with the Grubbs-type column system "Pure Solv MD-5". Degassed (freeze-pump-thawn), further dried and stored over molecular sieves (4 Å)
Ethanol (abs.)	Fisher Chemicals, ≥99.8%	used as received
n-Hexane	Local trade<br>or<br>Geyer, CHROMASOLV®, for HPLC, ≥97.0% (GC)	Dried over Na/benzophenone, freshly distilled prior to use. Degassed (freeze-pump-thawn), stored and further dried over molecular sieves (4 Å)<br>or<br>Purified with the Grubbs-type column system "Pure Solv MD-5". Dried over Na / benzophenone / tetraglyme, distilled, degassed (freeze-pump-thawn), stored and further dried over molecular sieves (4 Å)
n-Pentane	Local trade<br>Or<br>Sigma Aldrich, anhydrous, ≥99.0%	Dried over Na/benzophenone, freshly distilled prior to use. Degassed (freeze-pump-thawn), stored and further dried over molecular sieves (4 Å)<br>or<br>degassed (freeze-pump-thawn), further dried and stored over molecular sieves (4 Å)

Toluene	Fischer Chemicals, for HPLC<br>or<br>Local trade	Purified with the Grubbs-type column system "Pure Solv MD-5". Degassed (freeze-pump-thawn), further dried and stored over molecular sieves (4 Å)<br>or<br>Dried over Na/benzophenone, freshly distilled prior to use. Degassed (freeze-pump-thawn), stored and further dried over molecular sieves (4 Å)
Hexamethyldisiloxan (HMDSO)	Sigma Aldrich, ≥98.0%	Dried over Na/benzophenone, freshly distilled prior to use. Degassed (freeze-pump-thawn), stored and further dried over molecular sieves (4 Å)
Benzene-d_6	Euriso-top	Dried over Na, freshly distilled prior to use. Stored and further dried over molecular sieves (4 Å)
Toluene-d_8	Euriso-top	Dried over Na, freshly distilled prior to use. Stored and further dried over molecular sieves (4 Å)
Aluminum trichloride	Sigma Aldrich, anhydrous, powder, 99.99% trace metal basis	Used as received, stored under argon
Arsenic trichloride	Synthesized[78]	dried over P_4O_{10}<br>freshly distilled and degassed (freeze-pump-thaw) prior to use
Phosphorous trichloride	Merck	Refluxed over P_4O_{10}. Freshly distilled prior to use
1-Azido-4-fluorobenzene	Synthesized[7980]	Purified according to literature.
E/Z-Azobenzene	Thermo Fischer Scientific, ≥97.0%	Used as received
Z-Azobenzene	*E/Z*-Azobenzene	Isomer mixture dissolved in *n*-pentane and irradiated at ambient temperature for 24 h with Hg / Xe UV Lamp. Solvent removed at ambient temperature under light exclusion and purified by column chromatography (eluent: *n*-hexane / ethyl acetate 3:1).
2-Azido-1,3-dibromo-5-methylbenzene	synthesized[81]	Carefully dried *in vacuo* (1×10^{-3} mbar) at ambient temperature for 2 h
n-Butyllithium (2.5 M in *n*-hexane)	Acros Organics	Used as received
Pentamethylcyclopenta-dien (Cp*H)	synthesized[82]	Purified according to literature
1,3-Dichlorobenzene	TCI, ≥90.0%	Used as received
1-Azido-2,6-di*iso*propylphenyl ($DippN_3$)	synthesized[83]	Purified according to literature

1-Amino-2,6-di*iso*propylphenyl ($DippNH_2$)	TCI, ≥90.0%	Distilled prior to use
Hydrobromic acid (aq. HBr, 47%)	TCI	Used as received
Hydrochloric acid (aq. HCl, 37%)	Fisher Chemical, analytical reagent grade	Used as received
Iodine	TCI, ≥98.0%	Used as received
Potassium hydride (KH)	Sigma Aldrich, in mineral oil	Fourfold extraction with *n*-hexane, solid residue dried in vacuo (1×10^{-3} mbar)
Lithium aluminum hydride	abcr, 97%	Extracted with Et_2O, removal of insoluble impurities by filtration, drying of the filtrate to get pure, colorless $LiAlH_4$
Magnesium turnings	Thermo Scientific, ≥99%	Used as received
Magnesium sulfate ($MgSO_4$)	Fischer Chemical	Used as received
Sodium carbonate (Na_2CO_3)	Fluka. puriss.	Used as received
Sodium nitrite ($NaNO_2$)	Abcr, 98%	Used as received
Sodium sulfite ($NaSO_3$)	Thermo Scientific, ≥98%	Used as received
Sodium thiosulfate ($Na_2S_2O_3$)	Sigma Aldrich, 99%	Used as received
Trimethylphosphane (PMe_3)	synthesized[84]	Purified according to literature
Phenylazide (PhN_3)<br>or<br>PhN_3 (0.5 M solution in 2-methyltetrahydrofurane)	synthesized<br>or<br>Sigma-Aldrich, ≥95%	Purified according to literature<br>or<br>Degassed (freeze-pump-thaw), dried over molecular sieves
(Diazomethyl)trimethylsilane (TMSC(H)N_2, 10% in *n*-hexane)	Fisher Scientific	Used as received
Zinc dust	Acros Organics, ≥98%	Used as received, stored under argon

NMR spectra were recorded on Bruker spectrometers (AVANCE 400, AVANCE 500, Fourier 300 or Bruker Avance Neo I 600) and were referenced internally to the deuterated solvent (C_6D_6 δ_{ref} = 128.4 ppm, toluene-d_8 $\delta_{ref,1}$ = 20.4 ppm, $\delta_{ref,2}$ = 125.5 ppm, $\delta_{ref,3}$ = 128.3 ppm, $\delta_{ref,4}$ = 129.2 ppm, $\delta_{ref,5}$ = 137.9 ppm), to protic impurities in the deuterated solvent (C_6HD_5 δ_{ref} = 7.16 ppm, toluene-d_8 $\delta_{ref,1}$ = 2.08 ppm, $\delta_{ref,2}$ = 6.98 ppm, $\delta_{ref,3}$ = 7.00 ppm, $\delta_{ref,4}$ = 7.09 ppm) or externally (^{19}F: $CFCl_3$ δ_{ref} = 0 ppm, ^{29}Si: tetramethylsilane δ_{ref} = 0 ppm, ^{31}P: 85% H_3PO_4 δ_{ref} = 0 ppm). NMR signals were assigned using experimental data (e.g. chemical shifts, coupling constants, integrals where applicable).

IR spectra of crystalline samples were recorded on a Bruker Alpha II FT-IR spectrometer equipped with an ATR unit at ambient temperature under argon atmosphere. Relative intensities are reported according to the following intervals: very weak (vw, 0–20%), weak (w, 20−40%), medium (m, 40–60%), strong (s, 60–80%) and very strong (vs, 80−100%).

Elemental analyses of **1P**, **2P**, **3P**, **4P**, **4As**, **5P**, **6P**, **1P_F** were obtained using a Vario MICRO Cube elemental analyzer. Data for **2As** and **3As** were obtained using a Leco Tru Spec Mico elemental analyzer.

Mass spectra were recorded on a Thermo Electron MAT 95-XP sector field mass spectrometer and a Thermo Scientific Exactive Plus spectrometer in LIFDI mode using crystalline samples.

2 Structure elucidation

X-ray structure determination

X-ray quality crystals of **1P**, **1P·PMe$_3$**, **2P**, **3P**, **3P_mixed 4P**, **4As**, **5P**, **6P**, **6P^H** and **7P** were selected in Fomblin© Y perfluoroether (Sigma Aldrich) at ambient temperature under a constant stream of argon. X-ray quality crystals of ^{Dipp}TerAs(NPh)$_2$, **1As·PMe$_3$**, **2As** and **3As** were selected in Fomblin YR-1800 perfluoroether (Alfa Aesar) at ambient temperature without a stream of inert gas.

The samples were cooled to the temperature given in ***Table S2-S6*** during measurement. The data of **1P**, **1P·PMe$_3$**, **2P**, **3P**, **3P_mixed 4P**, **4As**, **5P**, **6P**, **6P^H** and **7P** and were collected on a Rigaku XtaLAB Synergy-R diffractometer with a HPA area detector and multi-layer mirror monochromated CuK$_\alpha$ radiation (λ = 1.54178 Å). The data of ^{Dipp}TerAs(NPh)$_2$, **1As·PMe$_3$**, **2As** and **3As** were collected on a Bruker Kappa Apex II diffractometer using CuK$_\alpha$ radiation (λ = 1.54178 Å) or Mo K$_\alpha$ radiation (λ = 0.71073 Å).

The structures ^{Dipp}TerAs(NPh)$_2$, **1As·PMe$_3$**, **2As** and **3As** were solved by iterative methods (SHELXT)[85] and refined by full matrix least squares procedures (SHELXL).[86] Semi-empirical absorption corrections were applied (SADABS).[87] All non-hydrogen atoms were refined anisotropically, hydrogen atoms were included in the refinement at calculated positions using a riding model.

The structures **1P**, **1P·PMe$_3$**, **2P**, **3P**, **3P_mixed 4P**, **4As**, **5P**, **6P**, **6P^H** and **7P** were solved using intrinsic phasing method,[85] refined with the SHELXL program[86] and expanded using Fourier techniques. All non-hydrogen atoms were refined anisotropically, hydrogen atoms were included in the refinement at calculated positions using a riding model.

Special refinement details

All special refinement details for disordered structures, molecular structure representations as well as a compilation of standard crystallographic details are summarized below.

In **1P•PMe$_3$** the *n*-pentane molecule is on a symmetry element and is isotropically refined as a special position disorder. The displacement parameters of atoms C1 to C5 of the residue 13 were restrained to the same value with similarity restraint SIMU with esd = 0.008.

In **1As·PMe$_3$** the asymmetric unit contains half a molecule *n*-hexane, which was split in two parts. The occupancy of each part was allowed to refine freely.

In **3P** a *n*-hexane molecule is on a symmetry element and is isotropically refined as a special position disorder. A low-resolution reflection affected by beam stop [0 0 2] was removed from refinement. Some other reflections [5 1 1], [-1 2 2], [2 1 1], [-2 2 2], [1 6 3], [0 4 6] were removed as outliers.

In **3P_mixed** the Cp* substituent is disordered. An idealized geometry for the disordered part is applied using AFIX 106. The atomic displacement parameters of all Cp* atoms C1_2 to C10_21 were restrained with RIGU keyword with esd = 0.008 in ShelXL input ('enhanced rigid bond' restraint for all bonds in the connectivity list). The displacement parameters of all Cp* atoms C1_2 to C10_21 were restrained to the same value with similarity restraint SIMU with esd = 0.016. One azide substituent shows an occupational disorder regarding to the dibromotolyl and Dipp substituent with an occupancy factor of 0.72 for the dibromotolyl-group. The atomic displacement parameters of the carbon atoms of these disorder C17_2 to C3_421 were restrained with RIGU keyword with esd = 0.008 in ShelXL input ('enhanced rigid bond' restraint for all bonds in the connectivity list). The displacement parameters of the carbon atoms of these disorder C17_2 to C3_421 were restrained to the same value with similarity restraint SIMU with esd = 0.008. The U_{ii} displacement parameters the carbon atoms of

these disorder C17_2 to C3_421 were restrained with ISOR keyword with esd = 0.004 to approximate isotropic behavior. The distances between atoms C18_41 to C1_411 were restrained to the value of 1.53 with esd = 0.01 using DFIX. It can therefore be concluded that the crystal represents co-crystallized **3P_mixed** (0.72) as the major component, with **3P** (0.28) also being present.

In **5P** the *n*-pentane molecule is on a symmetry element and is isotropically refined as a special position disorder. The displacement parameters of atoms C1 to C5 of the residue 13 were restrained to the same value with similarity restraint SIMU (esd = 0.008).

In **6P** The reflections [8 8 0], [7 7 0], [7 2 0] and [-3 3 1] were removed from refinement as outliers. One hexane molecule is on a symmetry element and is isotropically refined as a special position disorder. The other two hexane molecules, two isopropyl groups, one pentamethylcyclopentadienyl substituent and the central CN unit are disordered. For the central CN unit a CN/NC isomerisation disorder was applied with an occupancy factor of 46% (seventh free variable). The atomic displacement parameters of the hexane atoms C1_21 to C6_221, of the isopropyl group atoms C95_19 to C97_191 and C80_14 to C82_141, of the pentamethylcyclopentadienyl substituent atoms C16_4 to C26_41 and the atoms of the central CN unit N4_1 to C3_111 were restrained with RIGU keyword with esd = 0.016 in ShelXL input ('enhanced rigid bond' restraint for all bonds in the connectivity list). The displacement parameters of the hexane atoms C1_20 to C6_221, of the isopropyl group atoms C95_19 to C97_191 and C80_14 to C82_141, of the pentamethylcyclopentadienyl substituent atoms C16_4 to C26_41 and the atoms of the central CN unit N4_1 to C3_111 were restrained to the same value with similarity restraint SIMU with esd = 0.008. The U_{ii} displacement parameters of the hexane atoms C1_21 to C6_221, of the isopropyl group atoms C95_19 to C97_191 and C80_14 to C82_141, of the pentamethylcyclopentadienyl substituent atoms C16_4 to C26_41 and the atoms of the central CN unit N4_1 to C3_111 were restrained with ISOR keyword with esd = 0.004 to approximate isotropic behavior. The distances between the hexane

atoms C to C2, C2 to C3, C3 to C4, C4 to C5, C5 to C6 and C1 to C3, C3 to C5, C2 to C4, C4 to C6 were restrained to the value of 1.533 with esd = 0.003 and 2.541 with esd = 0.004 using DFIX.

In **6P^{H}** the reflections [-1 -2 5], [4 2 5], [-2 -3 5], [-2 1 3] and [-3 -4 2] were removed from refinement as outliers. The MePNSi(Me_3)$_3$ fragment of the main part of the molecule is disordered with an occupation factor of 95%.The atomic displacement parameters of atoms P1_1 to C3_21 were restraint with RIGU keyword with esd = 0.008 in ShelXL input ('enhanced rigid bond' restraint for all bonds in the connectivity list). The displacement parameters of atoms P1_1 to C3_21 were restrained to the same value with similarity restraint SIMU with esd = 0.008. The U_{ii} displacement parameters of atoms P1_1 to C3_21 were restrained with ISOR keyword to approximate isotropic behavior with esd = 0.008. The distances between atoms P1_1 to N1_1 and P1_11 to N1_11; P1_1 to N2_1 and P1_11 to N2_1; P1_1 to C1_1 and P1_11 to C1_11; N1_1 to Si1_2 and N1_11 to Si1_21 were restrained during refinement to the same value using SADI with esd = 0.001. Additionally idealized distances in the disordered trimethylsilyl group were restrained during refinement to the value of 1.880 with esd = 0.005 using DFIX for the Si-C bonds and idealized 1,3 distances for C-Si-C were restrained to the value of 3.000 with esd = 0.005 using DANG. Both CN groups at the aluminium atom were refined as CN/NC disorder with an occupancy factor of 44% and 81%.

In **7P** one *n*-pentane molecule is disordered. The other disorder is a solvent mixture of 78% toluene and 22% *n*-pentane molecules, due to the refinement of the second free variable. The atomic displacement parameters of all *n*-pentane atoms C1_14 to C5_15 were restrained with RIGU keyword in ShelXL input with esd = 0.032 ('enhanced rigid bond' restraint for all bonds in the connectivity list). The displacement parameters of all *n*-pentane atoms C1_14 to C5_15 were restrained to the same value with similarity restraint SIMU with esd = 0.032. The U_{ii} displacement parameters of all *n*-pentane atoms C1_14 to C5_15 were restrained with ISOR keyword with esd = 0.032 to approximate isotropic behaviour. The distances between the single C−C bonds in

pentane atoms C1 to C2, C2 to C3, C3 to C4, C4 to C5 were restrained to the value of 1.531 with esd = 0.002. The distances between the C−C bonds in the *n*-pentane atoms C1 to C3, C3 to C5 and C2 to C4 were restrained to the value of 2.552 with esd = 0.003[8].

Table S2: Crystallographic details of **1P**, **^{Dipp}TerAs(NPh)$_2$**, **1P·PMe$_3$**.

Compound	**1P**	**^{Dipp}TerAs(NHPh)$_2$**	**1P·PMe$_3$**
Chem. Formula	$C_{52}H_{62}AlN_2P$	$C_{42}H_{49}AsN_2$	$C_{115}H_{154}Al_2N_4P_4$
Formula weight [g/mol]	772.98	656.75	1770.25
Colour	colourless	colourless	colourless
Crystal system	monoclinic	monoclinic	monoclinic
Space group	$P2_1/c$	$P2_1/n$	$P2_1/c$
a [Å]	20.1021(3)	12.5153(8)	12.96210(10)
b [Å]	11.9767(2)	16.8483(10)	17.77090(10)
c [Å]	18.2230(3)	17.7055(11)	23.00720(10)
α [°]	90	90	90
β [°]	91.6500(10)	109.2890(10)	91.7760(10)
γ [°]	90	90	90
V [Å^3]	4385.49(12)	3523.8(4)	5297.12(6)
Z	4	4	2
$\rho_{calcd.}$ [g/cm^3]	1.171	1.238	1.110
μ [mm^{-1}]	1.018	0.996	1.174
T [K]	99.98(10)	150(2)	100(2)
Measured reflections	41778	47375	103481
Independent reflections	7726	9584	10672
Reflections with $I > 2\sigma(I)$	6748	7964	9115
R_{int}	0.0379	0.0303	0.0622
$F(000)$	1664	1392	1916
$R_1(R[F^2>2\sigma(F^2)])$	0.0351	0.0335	0.0532
w$R_2(F^2)$	0.0896	0.0875	0.1513
GooF	1.057	1.035	1.071
No. of Parameters	518	420	577
CCDC #	2394499	2394510	2394500

Table S3: Crystallographic details of **1As·PMe$_3$, 2P, 2As**.

Compound	**1As·PMe$_3$**	**2P**	**2As**
Chem. Formula	$C_{55}H_{71}AlAsN_2P \cdot 0.5 (C_6H_{14})$	$C_{54}H_{62}AlBr_4N_4P$	$C_{54}H_{62}AlAsBr_4N_4$
Formula weight [g/mol]	936.09	1144.66	717.63
Colour	yellow	colourless	colourless
Crystal system	monoclinic	monoclinic	monoclinic
Space group	$P2_1/c$	$P2_1/c$	$P2_1/c$
a [Å]	13.0690(4)	18.51350(10)	18.6526(8)
b [Å]	17.8479(6)	11.28450(10)	11.4101(5)
c [Å]	22.9496(7)	24.9383(2)	24.8385(11)
α [°]	90	90	90
β [°]	91.4410(10)	93.6390(10)	93.394(2)
γ [°]	90	90	90
V [Å^3]	5351.4(3)	5199.49(7)	5277.1(4)
Z	4	4	4
$\rho_{calcd.}$ [g/cm^3]	1.162	1.462	1.496
μ [mm^{-1}]	1.570	4.530	4.874
T [K]	150(2)	100(2)	150(2)
Measured reflections	47490	119918	77240
Independent reflections	9487	10429	9327
Reflections with $I > 2\sigma(I)$	8897	9198	8926
R_{int}	0.0328	0.0460	0.0337
$F(000)$	2004	2328	2400
$R_1(R[F^2>2\sigma(F^2)])$	0.0267	0.0334	0.0370
w$R_2(F^2)$	0.0715	0.0875	0.1030
GooF	1.021	1.046	1.039
No. of Parameters	612	592	592
CCDC #	2394501	2394502	2394748

Table S4: Crystallographic details of **3P, 3As, 4P**.

Compound	**3P**	**3As**	**4P**
Chem. Formula	$C_{67}H_{93}AlN_6P$	$C_{64}H_{86}AlAsN_6{\cdot}0.5(C_7H_8)$	$C_{48}H_{72}AlN_4PSi_2$
Formula weight [g/mol]	1040.42	1087.35	819.22
Colour	colourless	yellow	yellow
Crystal system	monoclinic	monoclinic	monoclinic
Space group	$P2_1/c$	$P2_1/c$	$P2_1/n$
a [Å]	23.9224(3)	24.0385(13)	12.63970(10)
b [Å]	11.08550(10)	11.1516(6)	15.87560(10)
c [Å]	22.9448(2)	23.0388(13)	24.1562(2)
α [°]	90	90	90
β [°]	90.0460(10)	90.068(2)	91.4040(10)
γ [°]	90	90	90
V [Å^3]	6084.77(11)	6176.0(6)	4845.80(6)
Z	4	4	4
$\rho_{calcd.}$ [g/cm^3]	1.136	1.169	1.123
μ [mm^{-1}]	0.869	1.210	1.411
T [K]	100(2)	150(2)	100(2)
Measured reflections	137030	66371	112890
Independent reflections	12105	10925	9808
Reflections with $I > 2\sigma(I)$	9538	10596	8790
R_{int}	0.0708	0.0281	0.0323
F(000)	2260	2332	1776
$R_1(R[F^2>2\sigma(F^2)])$	0.0521	0.0265	0.0331
w$R_2(F^2)$	0.1455	0.0711	0.0830
GooF	1.063	1.025	1.029
No. of Parameters	694	670	524
CCDC #	2394749	2394503	2394504

Table S5: Crystallographic details **4As, 5P, 6P**.

Compound	**4As**	**5P**	**6P**
Chem. Formula	$C_{48}H_{72}AlAsN_4Si_2$	$C_{101}H_{156}Al_2N_8P_2Si_4$	$C_{111}H_{179}Al_2N_8P_2Si_4$
Formula weight [g/mol]	863.17	1710.59	1853.87
Colour	colourless	red	colourless
Crystal system	monoclinic	monoclinic	monoclinic
Space group	$P2_1/n$	$P2_1/n$	$P2_1/c$
a [Å]	12.57780(10)	12.06910(10)	20.6386(2)
b [Å]	17.67630(10)	34.54760(10)	21.8174(2)
c [Å]	22.03430(10)	12.86850(10)	25.0900(3)
α [°]	90	90	90
β [°]	95.11	107.0240(10)	91.1460(10)
γ [°]	90	90	90
V [Å^3]	4879.36(5)	5130.52(7)	11295.3(2)
Z	4	2	4
$\rho_{calcd.}$ [g/cm^3]	1.175	1.107	1.090
μ [mm^{-1}]	1.845	1.351	1.259
T [K]	100(2)	100(2)	100(2)
Measured reflections	112853	119735	265075
Independent reflections	9751	10277	22562
Reflections with $I > 2\sigma(I)$	9573	9662	18262
R_{int}	0.0311	0.0576	0.0680
F(000)	1848	1860	4052
$R_1(R[F^2>2\sigma(F^2)])$	0.0263	0.0366	0.0661
$wR_2(F^2)$	0.0713	0.0974	0.1970
GooF	1.057	1.043	1.026
No. of Parameters	524	545	1473
CCDC #	2394505	2394506	2394507

Table S6: Crystallographic details **3P_mixed**, **6P^{H}, 7P**.

Compound	**3P_mixed**	**6P^{H}**	**7P**
Chem. Formula	$C_{60.42}H_{77.40}AlBr_{1.43}N_6P$	$C_{49}H_{73}AlN_5PSi_2$	$C_{78.56}H_{105.89}AlN_5P$
Formula weight [g/mol]	1060.18	846.25	1178.20
Colour	colourless	yellow	colourless
Crystal system	monoclinic	triclinic	monoclinic
Space group	$P2_1/n$	$P\bar{1}$	$P2_1/n$
a [Å]	18.9461(3)	11.3487(2)	12.44880(10)
b [Å]	12.53010(10)	12.3219(2)	17.2108(2)
c [Å]	24.6952(3)	19.0077(4)	32.7832(4)
α [°]	90	88.015(2)	90
β [°]	109.0400(10)	86.623(2)	94.3810(10)
γ [°]	0.090	70.216(2)	90
V [Å^3]	5541.82(12)	2496.46(9)	7003.40(13)
Z	4	2	4
$\rho_{calcd.}$ [g/cm^3]	1.271	1.126	1.117
μ [mm^{-1}]	2.132	1.391	0.805
T [K]	100(2)	100(2)	100(2)
Measured reflections	130267	59917	96286
Independent reflections	11113	9833	13960
Reflections with $I > 2\sigma(I)$	10074	8226	11054
R_{int}	0.0568	0.0379	0.0496
$F(000)$	2240	916	2561
$R_1(R[F^2>2\sigma(F^2)])$	0.0827	0.0528	0.0491
$wR_2(F^2)$	0.2256	0.1500	0.1258
GooF	1.057	1.039	1.043
No. of Parameters	810	645	888
CCDC #	2432521	2394508	2394509

Figure S1: Molecular structure of **1P**. Ellipsoids are set at 50% probability (100.0(1) K) with H-atoms omitted. Both Dipp-groups and the Me-groups of Cp* rendered as wireframe for clarity. Selected bond lengths [Å] and angles [°]: P1−N4 1.717(1), P1−N2 1.722(1), N2−Al3 1.839(1), Al3−N4 1.851(1); P1−N4−Al3 95.07(6), N4−P1−N2 88.12(6), P1−N2−Al3 95.31(6), N2−Al3−N4 80.78(5), P1−N2−Al3−N4 6.23(5).

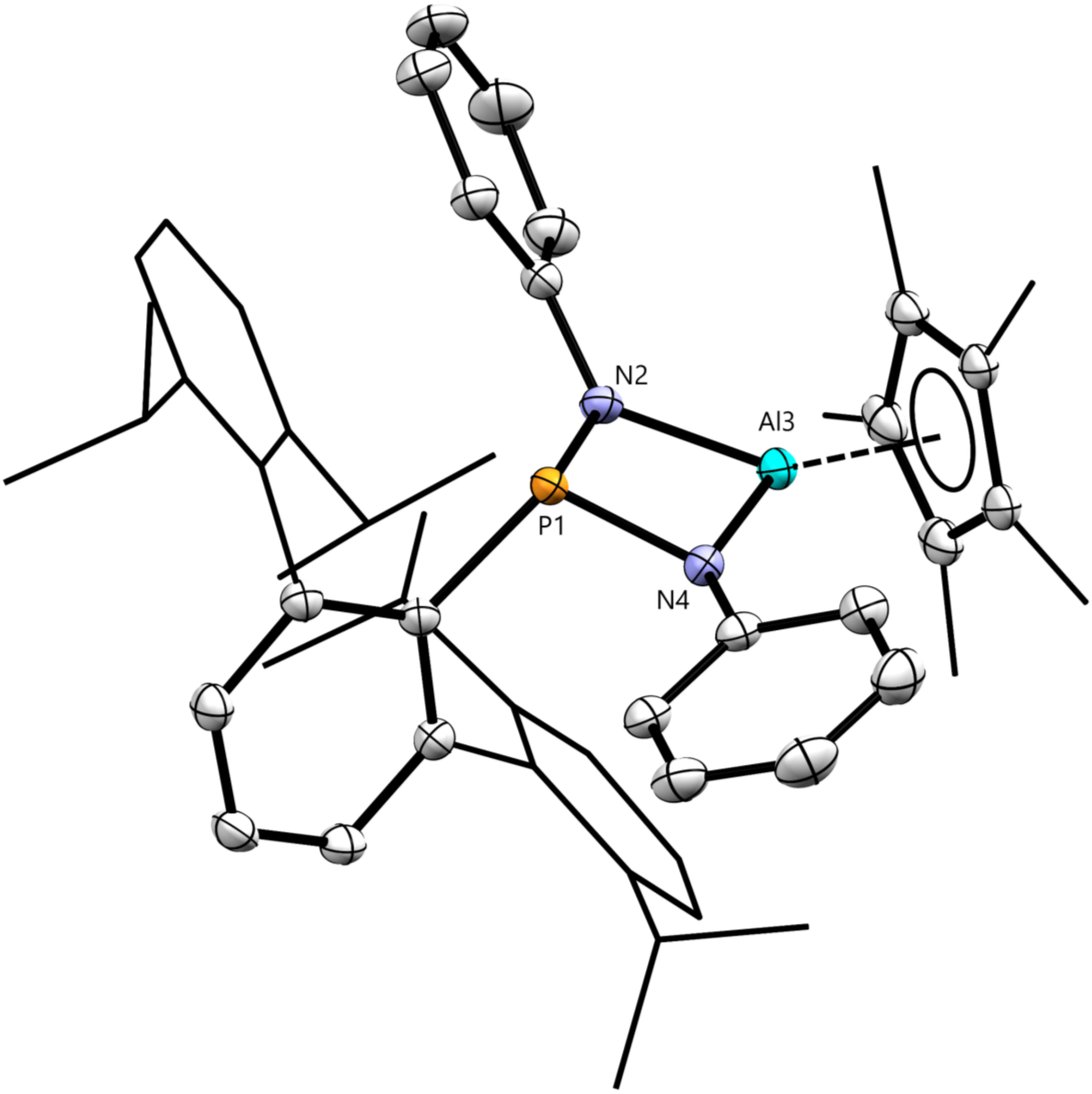

Figure S2: Molecular structure of **1P•PMe₃**. Ellipsoids are set at 50% probability (100(2) K) with H-atoms and *n*-pentane molecule omitted. Both Dipp-groups and the Me-groups of Cp* rendered as wireframe for clarity. Selected bond lengths [Å] and angles [°]: P1−N2 1.727(1), P1−N1 1.737(1), Al1−N2 1.856(1), Al1−N1 1.873(1), Al1−P2 2.4619(6); N2−P1−N1 87.16(6), N2−Al1−N1 79.63(6), N1−Al1−P2 101.03(5), N2−Al1−P2 100.92(4), P1−N1−Al1 94.35(6), P1−N2−Al1 95.34(6); N1−P1−N2−Al1 14.67(6), P2−Al1−N1−P1 85.54(5), P2−Al1−N2−P1 85.58(5).

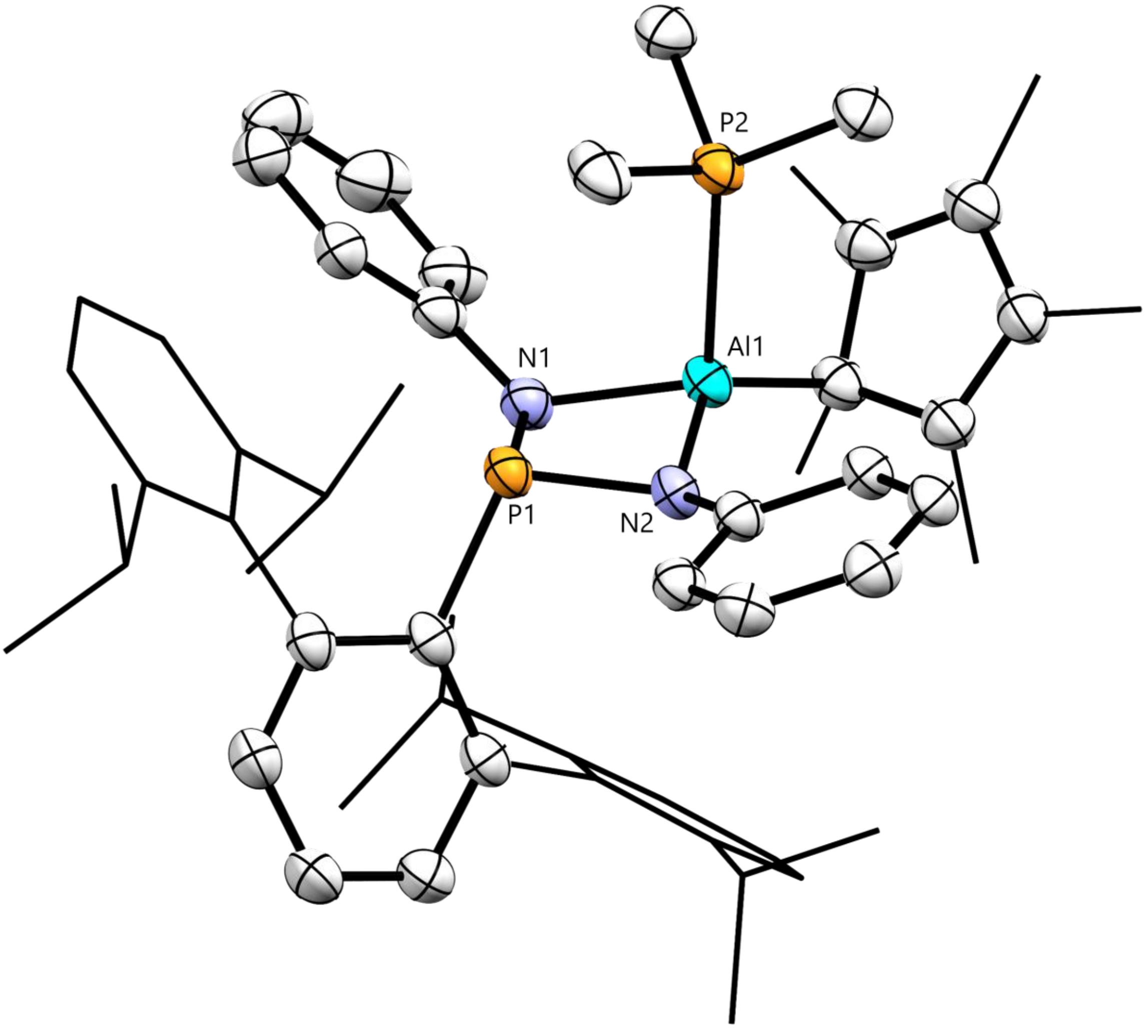

Figure S3: Molecular structure of **1As•PMe₃**. Ellipsoids are set at 50% probability (150(2) K) with H-atoms and *n*-hexane molecule omitted. Both Dipp-groups and the Me-groups of Cp* rendered as wireframe for clarity. Selected bond lengths [Å] and angles [°]: As1−N2 1.886(1), As1−N1 1.874(1), Al1−N2 1.866(1), Al1−N1 1.852(1), Al1−P1 2.4634(5), Al1−C43 2.030(1); N2−As1−N1 82.51(5), N2−Al1−N1 83.61(5), N1−Al1−P1 101.21(4), N2−Al1−P1 101.00(4), As1−N1−Al1 95.52(5), As1−N2−Al1 94.66(5); As1−N2−Al1−N1 −14.53(5) As1−N2−Al1−P1 −50.3(1), As1−N2−Al1−P1 85.69(4).

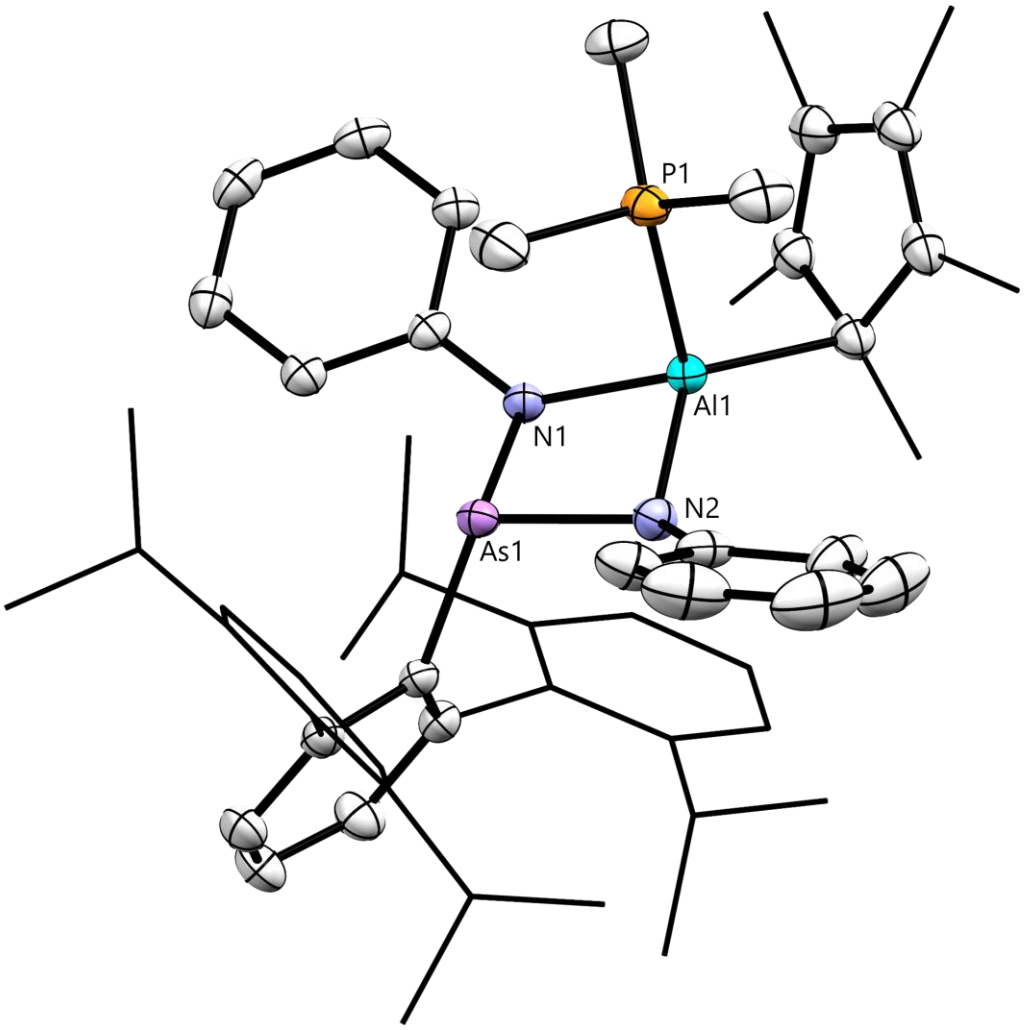

Figure S4: Molecular structure of **2P**. Ellipsoids are set at 50% probability (100(2) K) with H-atoms omitted. Both Dipp-groups and the Me-groups of Cp* rendered as wireframe for clarity. Selected bond lengths [Å] and angles [°]: P1−N1 1.754(2), P1−N2 1.760(2), Al1−N1 1.848(2), Al1−N2 1.853(2), N2−N3 1.336(3), N3−N4 1.276(3); N1−P1−N2 85.61(10), N1−Al1−N2 80.35(9), P1−N1−Al1 96.7(1), P1−N2−Al1 96.3(1), N2−P1−N1−Al1 7.8(1).

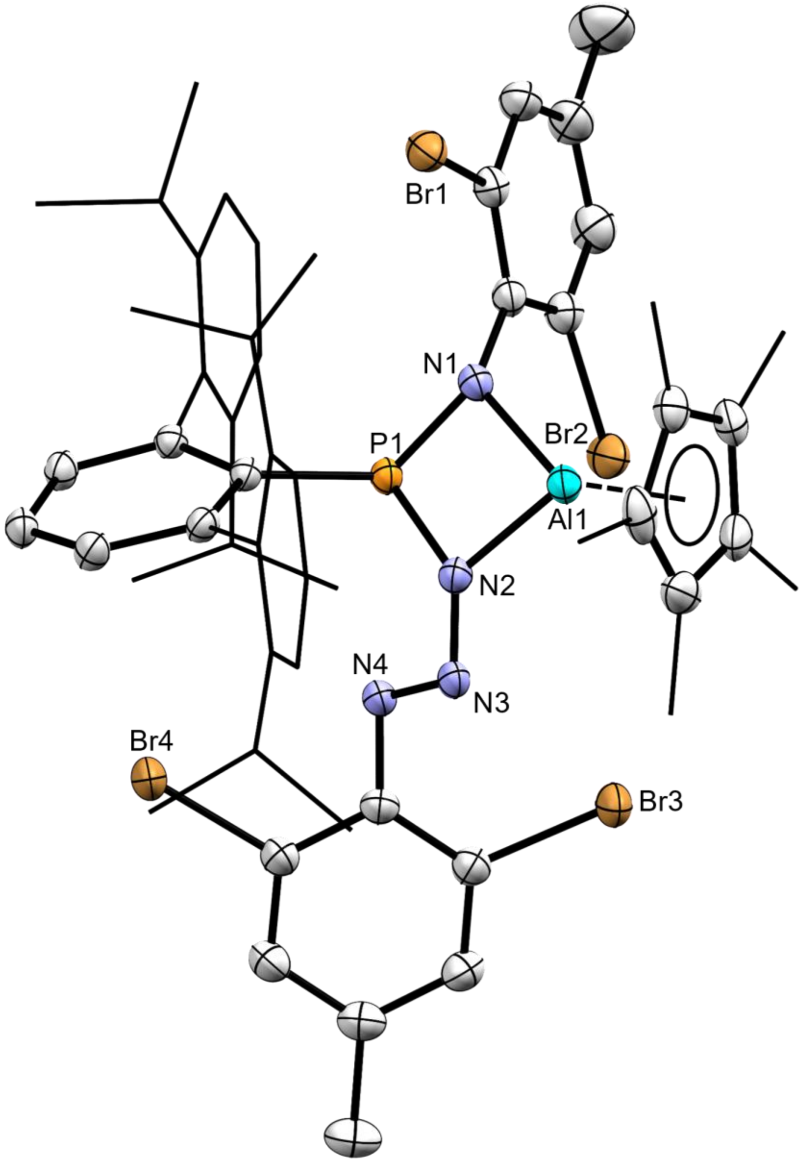

Figure S5: Molecular structure of **2As**. Ellipsoids are set at 50% probability (150(2) K) with H-atoms omitted. Both Dipp-groups and the Me-groups of Cp* rendered as wireframe for clarity. Selected bond lengths [Å] and angles [°]: C31–N3 1.416(4), N3–N2 1.278(3), N2–N1 1.327(4), N1–As1 1.907(2), N1–Al1 1.855(3), N4–Al1 1.838(3), N4–As1 1.913(2), N4–C38 1.415(4), C31–N3–N2 113.8(2), N3–N2–N1 113.0(2), Al1–N1–As1 96.7(1), N1–As1–N4 80.8(1), As1–N4–Al1 97.1(1), N1–Al1–N4 84.2(1), N2–N1–As1 117.3(2), As1–N4–C38 112.5(2), C31–N3–N2–N1 176.0(2), N3–N2–N1–As1 −15.0(3), As1–N1–Al1–N4 8.2(1), N1–Al1–N4–C38 −134.2(3).

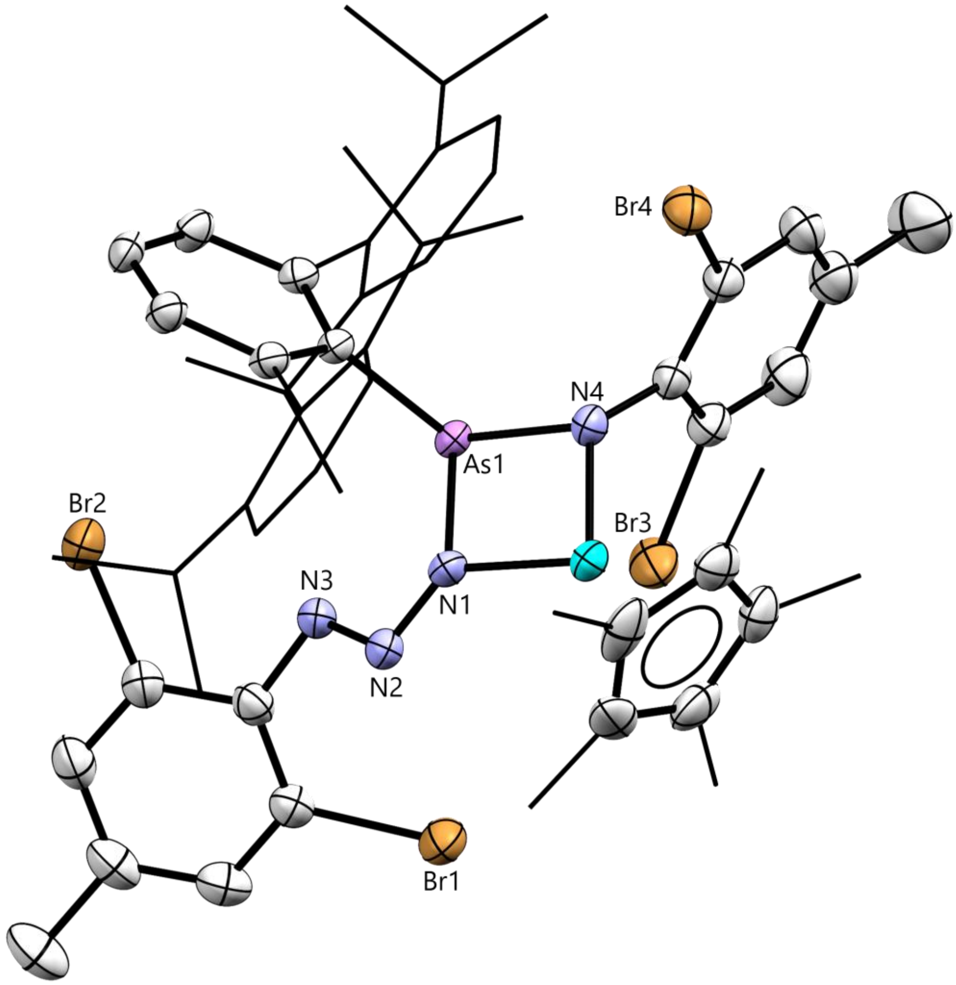

Figure S6: Molecular structure of **3P**. Ellipsoids are set at 50% probability (100(2) K) with H-atoms and hexane molecule omitted. Both Dipp-groups, the Me-groups of Cp* and the *i*-Pr groups rendered as wireframe for clarity. Selected bond lengths [Å] and angles [°]: P1−N1 1.769(2), P1−N4 1.776(2), Al1−N1 1.846(2), Al1−N4 1.855(2), N1−N2 1.348(2), N2−N3 1.274(2), N4−N5 1.359(2), N5−N6 1.267(2); N1−P1−N4 82.61(7), N1−Al1−N4 78.45(7), P1−N1−Al1 98.79(8), P1−N4−Al1 98.20(8), N3−N2−N1 111.98(15), N6−N5−N4 112.79(15), N4−Al1−N1−P1 10.49(7).

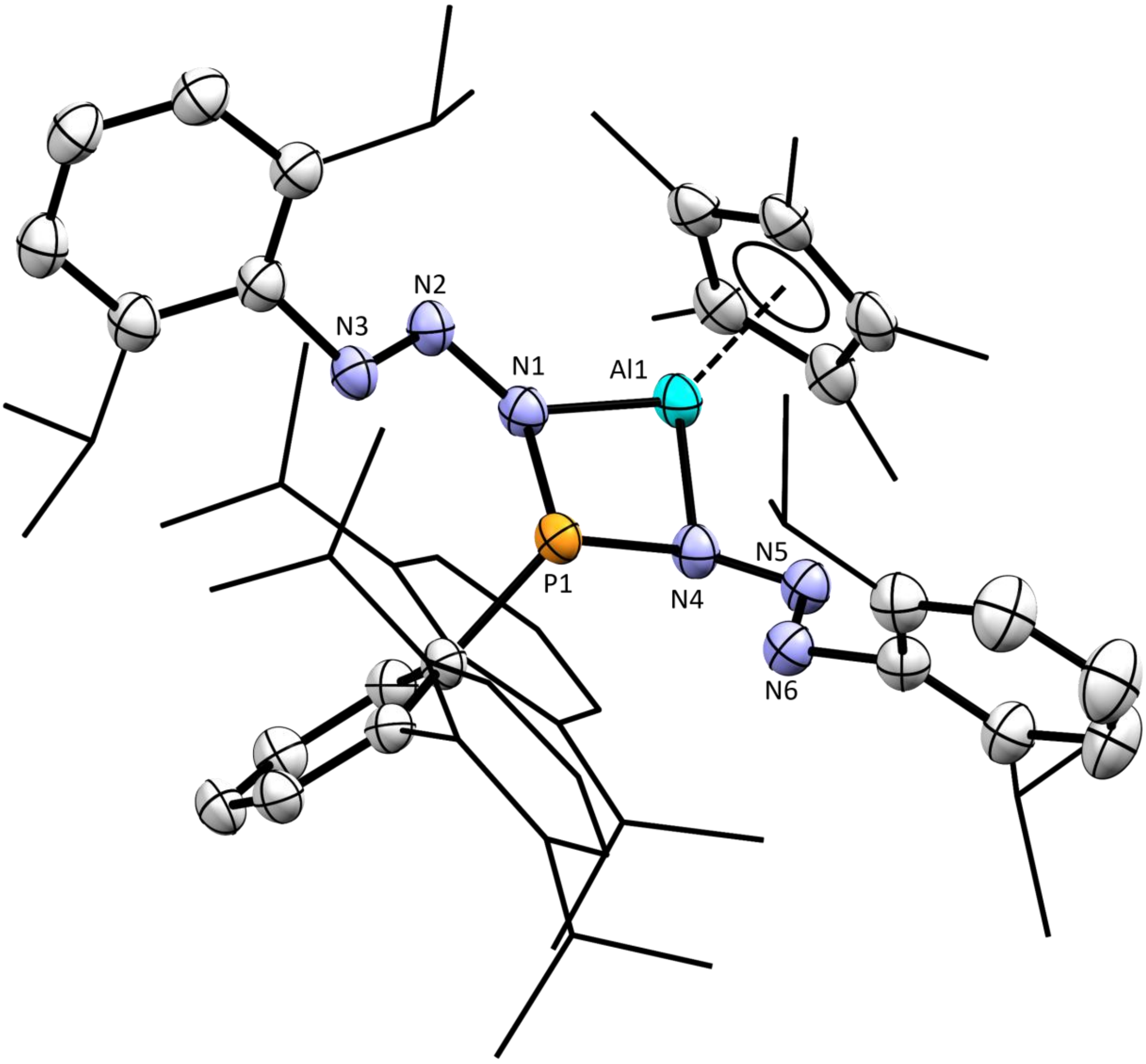

Figure S7: Molecular structure of **3P_mixed**. **3P_mixed** co-crystallizes with **3P**, and the minor component (**3P**) is not shown. Ellipsoids are set at 50% probability (100(2) K) with H-atoms omitted with exception at C1. All carbon atoms are rendered as wireframe. Selected bond lengths [Å] and angles [°]: P1−N2 1.772(3), P1−N1 1.776(3), P1−Al1 2.7436(16), Al1−N1 1.842(4), Al1−N2 1.846(3), N1−N3 1.344(4), N2−N4 1.366(5), N3−N5 1.269(5), N4−N6 1.267(5); N2−P1−N1 82.22(16), N1−Al1−N2 78.45(15), N3−N1−P1 121.8(3), N3−N1−Al1 135.6(3), P1−N1−Al1 98.64(17), N4−N2−P1 119.4(3), N4−N2−Al1 123.6(3), P1−N2−Al1 98.61(16), N5−N3−N1 113.0(3), N6−N4−N2 113.9(3); N2−P1−N1−Al1 11.16(18), P1−N1−N3−N5 14.7(5), P1−N2−N4−N6 21.5(5).

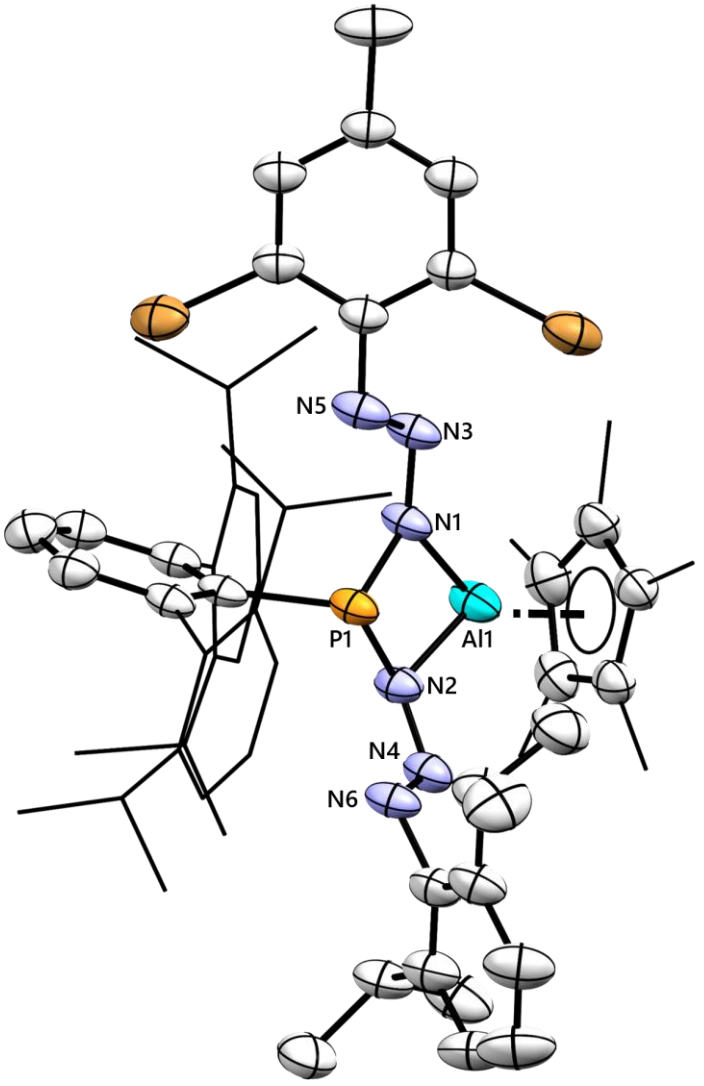

Figure S8: Molecular structure of 3**As**. Ellipsoids are set at 50% probability (150(2) K) with H-atoms and hexane molecule omitted. Both Dipp-groups, the Me-groups of Cp* and the *i*-Pr groups rendered as wireframe for clarity. Selected bond lengths [Å] and angles [°]: N6–C53 1.439(2), N5–N6 1.268(1), N5–N4 1.348(1), N4–As1 1.931(1), As1–N1 1.920(1), N4–Al1 1.852(1), Al1–N1 1.840(1), N1–N2 1.342(1), N2–N3 1.271(1), N3–C41 1.435(2), C53–N6–N5 114.0(1), N6–N5–N4 113.20(9), N5–N4–As1 115.68(7), As1–N4–Al1 98.50(5), N4–As1–N1 77.95(4), As1–N1–Al1 99.29(5), N1–Al1–N4 81.99(5), As1–N1–N2 117.64(7), N1–N2–N3 112.47(9), N2–N3–C41 115.0(1), C53–N6–N5–N4 -168.4(1), N6–N5–N4–As1 21.6(1), Al1–N4–As1–N1 11.23(4), As1–N1–N2–N3 -18.4(1), N1–N2–N3–C41 173.0(1).

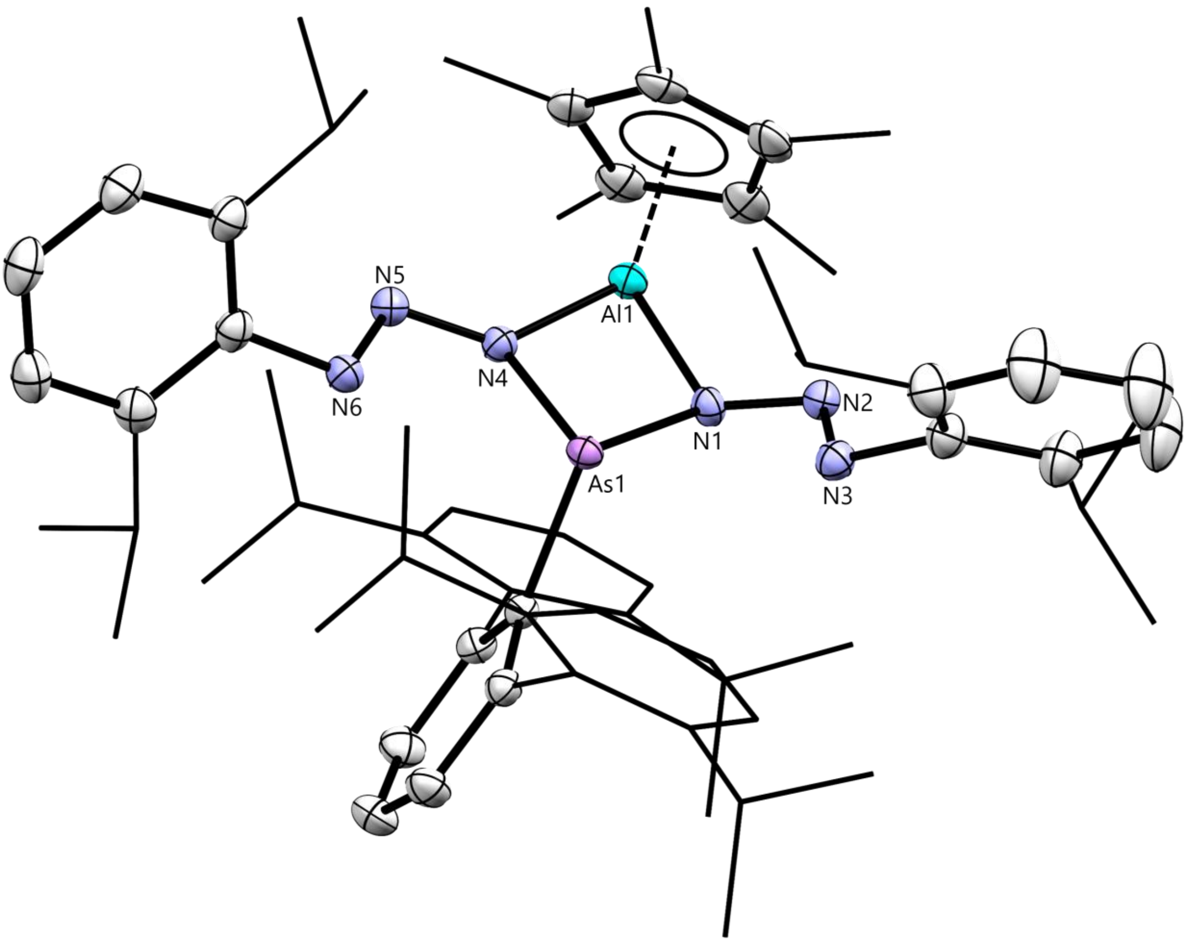

Figure S9: Molecular structure of **4P**. Ellipsoids are set at 50% probability (100(2) K) with H-atoms omitted with exception at C1 and C2. Both Dipp-groups and the Me-groups of Cp* rendered as wireframe for clarity. Selected bond lengths [Å] and angles [°]: P1–N1 1.753(1), P1–N3 1.754(1), Al1–N1 1.833(1), Al1–N3 1.849(1), N1–N2 1.367(1), N3–N4 1.380(2), C1–N2 1.291(2), C2–N4 1.287(2); N1–P1–N3 83.93(5), N1–Al1–N3 79.09(5), P1–N1–Al1 98.26(5), P1–N3–Al1 97.62(5), C1–N2–N1 120.36(11), C2–N4–N3 120.94(11), N3–P1–N1–Al1 8.13(5).

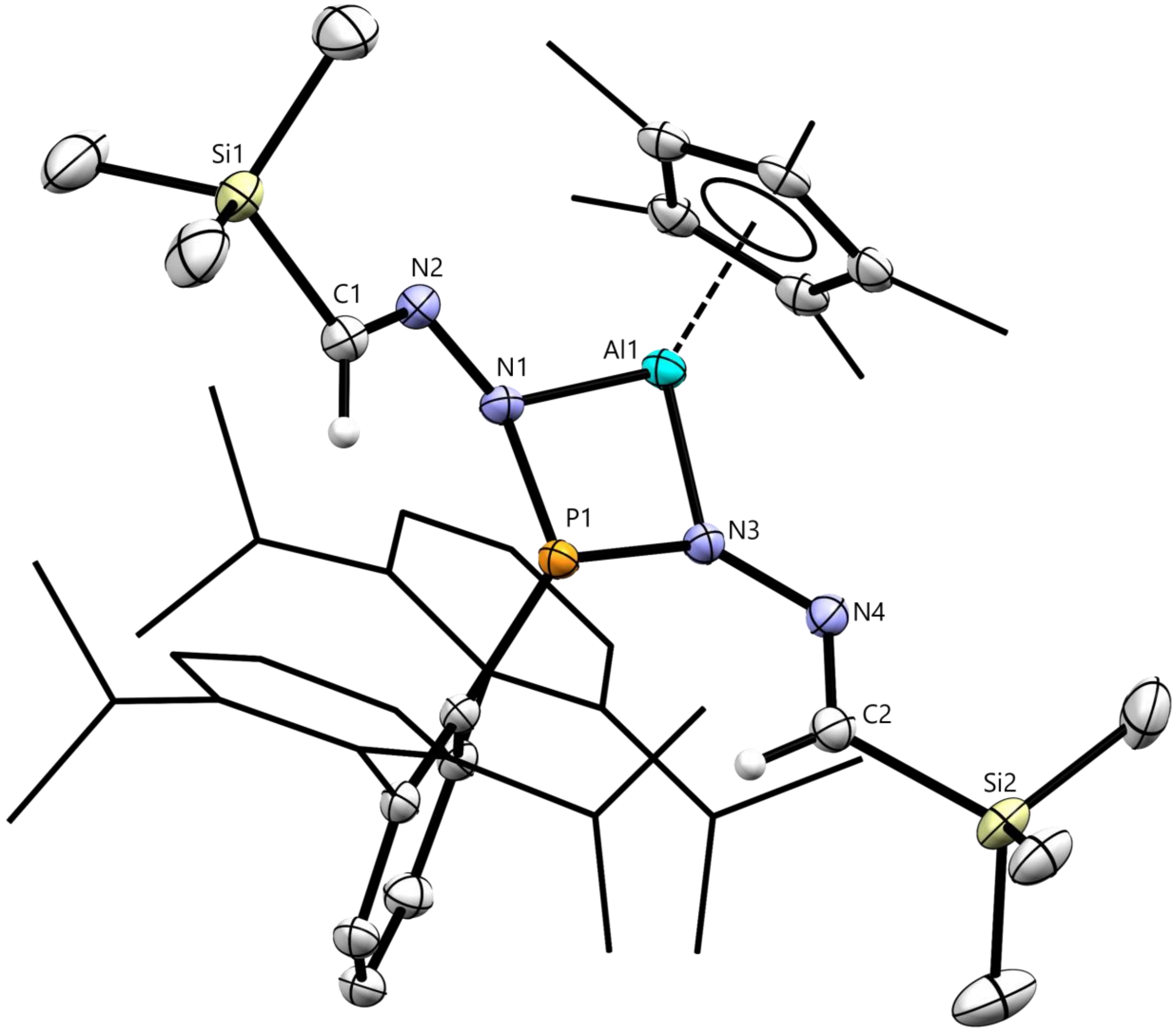

Figure S10: Molecular structure of **4As**. Ellipsoids are set at 50% probability (100(2) K) with H-atoms omitted with exception at C1 and C2. Both Dipp-groups and the Me-groups of Cp* rendered as wireframe for clarity. Selected bond lengths [Å] and angles [°]: As1−N1 1.907(1), As1−N3 1.926(1), Al1−N1 1.832(1), Al1−N3 1.840(1), N1−N2 1.358(2), N3−N4 1.366(1), C1−N2 1.291(2), C2−N4 1.287(2); N1−As1−N3 78.82(4), N1−Al1−N3 83.01(5), As1−N1−Al1 98.80(5), As1−N3−Al1 97.83(5), C1−N2−N1 121.06(11), C2−N4−N3 122.6(1), N3−As1−N1−Al1 9.64(5).

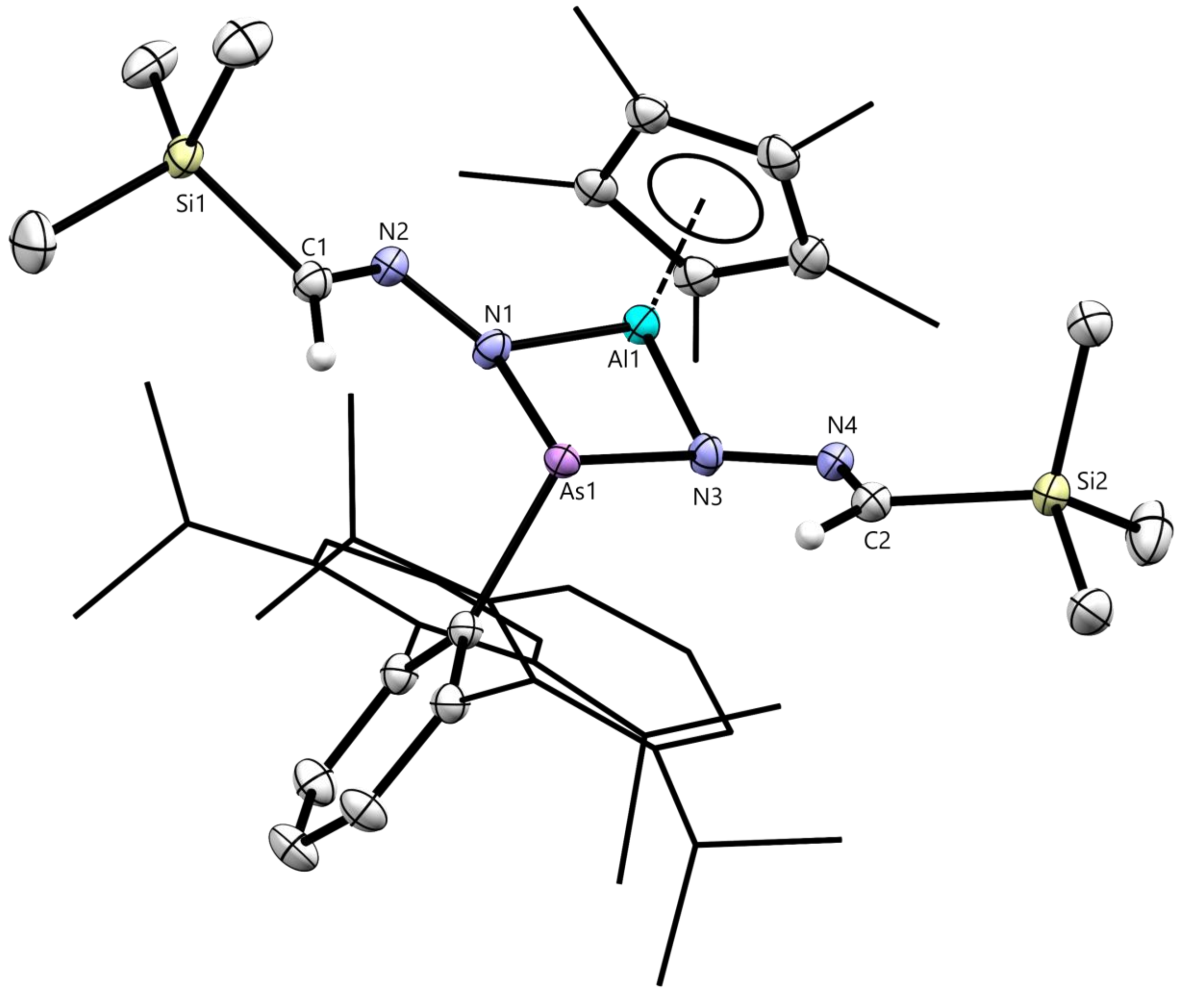

Figure S11: Molecular structure of **5P**. Ellipsoids are set at 50% probability (100(2) K) with *n*-pentane molecule and H-atoms omitted with exception at C1. Both of the Dipp-groups, the Me-groups of Cp* and Si(Me$_3$)$_3$ rendered as wire-frame for clarity. Selected bond lengths [Å] and angles [°]: P1−N3 1.673(1), P1−N1 1.678(1), Al1−N3 1.896(1), Al1−C49 1.965(1), Al1−N2 1.981(1), N1−N2 1.293(2), C1−N2 1.468(2), C1−Si1 1.927(2), N3−Si2 1.776(1), C49−N50 1.148(2); N50−C49−Al1 175.1(2); N3−P1−N1−N2 19.8(1).

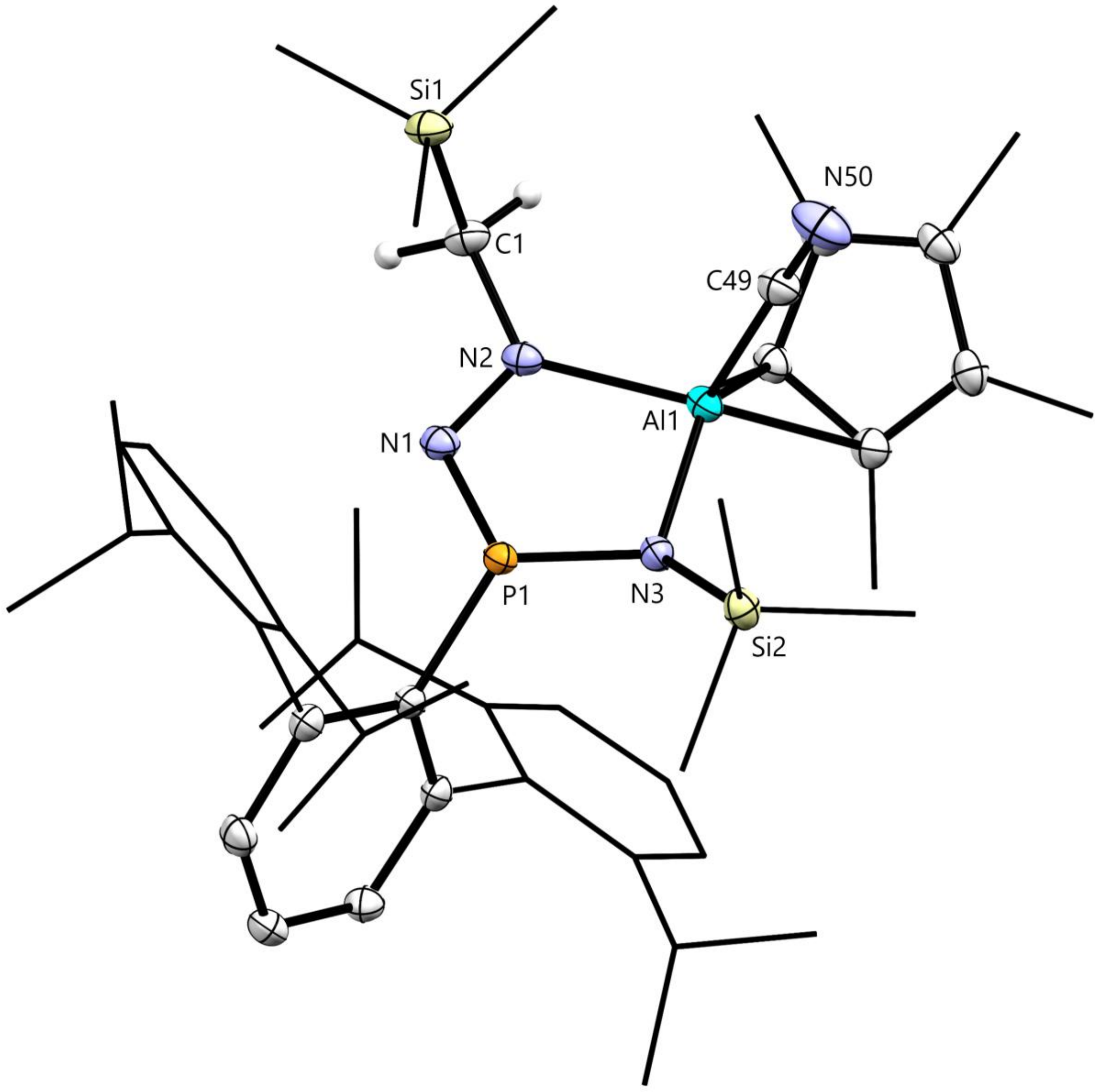

Figure S12: Molecular structure of **6P.** Ellipsoids are set at 50% probability (100(2) K) with H-atoms except on C16 and C27, *n*-hexane, disordered isopropyl groups and disordered Cp* omitted. . Both of the Dipp-groups, the Me-groups of Cp* and $Si(Me_3)_3$ rendered as wire-frame for clarity. Selected bond lengths [Å] and angles [°]: P1−N2 1.606(2), P1−N1 1.651(2), P1−C1 1.802(3), P1−Al1 2.5050(9), P2−N7 1.571(2), P2−N8 1.639(2), P2−C2 1.795(3), P2−Al2 2.5589(9), Al1−N3 1.802(2), Al1−N1 1.883(2), Al1−N2 1.959(2), Al1−Si1 2.6067(10), Al2−N6 1.842(2), Al2−N8 1.955(2), Al2−C4 1.962(3), Al2−N7 2.062(2), Al2−N4 2.132(19), Al2−Si2 2.6675(10), Si1−N3 1.728(2), Si1−N2 1.765(2), Si2−N6 1.721(2), Si2−N7 1.751(2), N1−Si3 1.755(2), N8−Si4 1.747(2), C4−N5 1.160(3); N2−P1−N1 100.1(1), N7−P2−N8 102.8(1), N3−Al1−N1 131.9(1), N1−Al1−N2 81.02(9); N2−P1−N1−Al1 3.6(1), N3−Si1−N2−Al1 1.0(1).

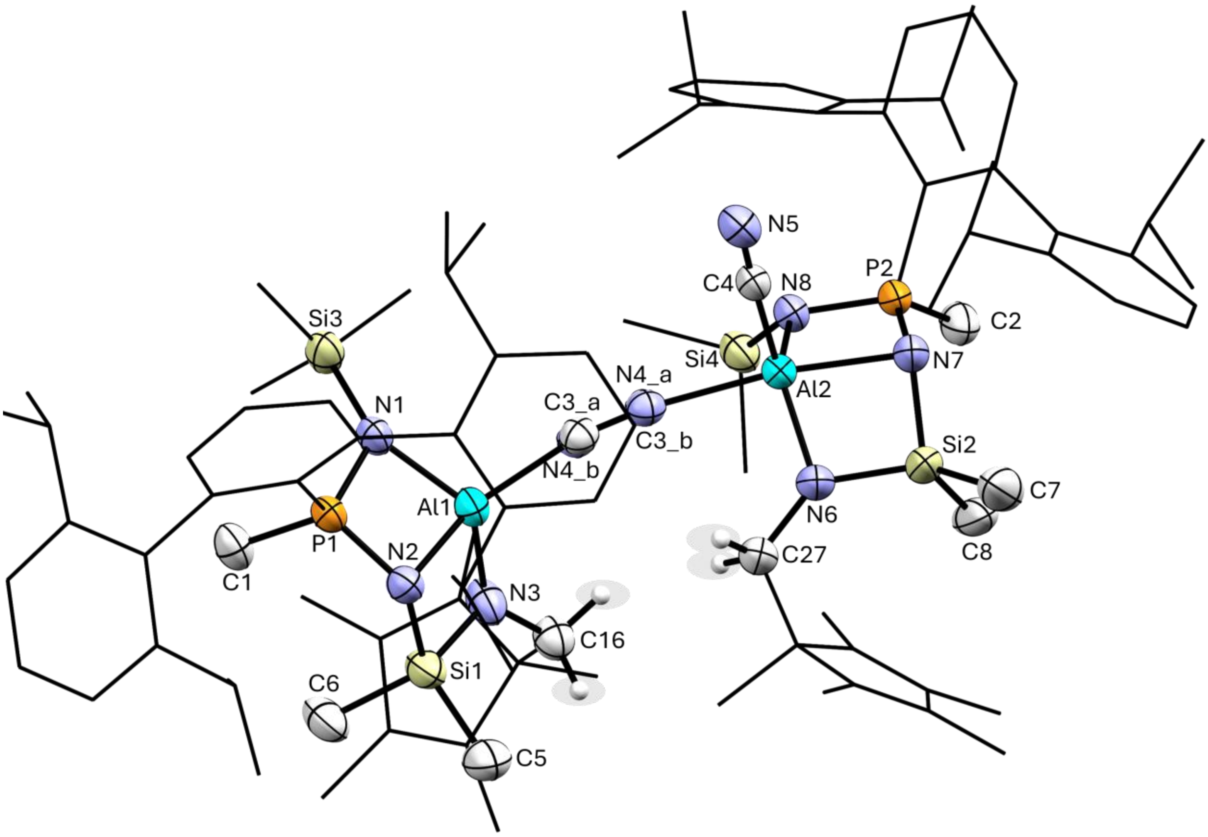

Figure S13: Molecular structure of **6P** (detailed view). Ellipsoids are set at 50% probability (100(2) K) with H-atoms except on C16 and C27, *n*-hexane, Cp* and ^{Dipp}Ter substituent omitted. $Si(Me_3)_3$ rendered as wire-frame for clarity.

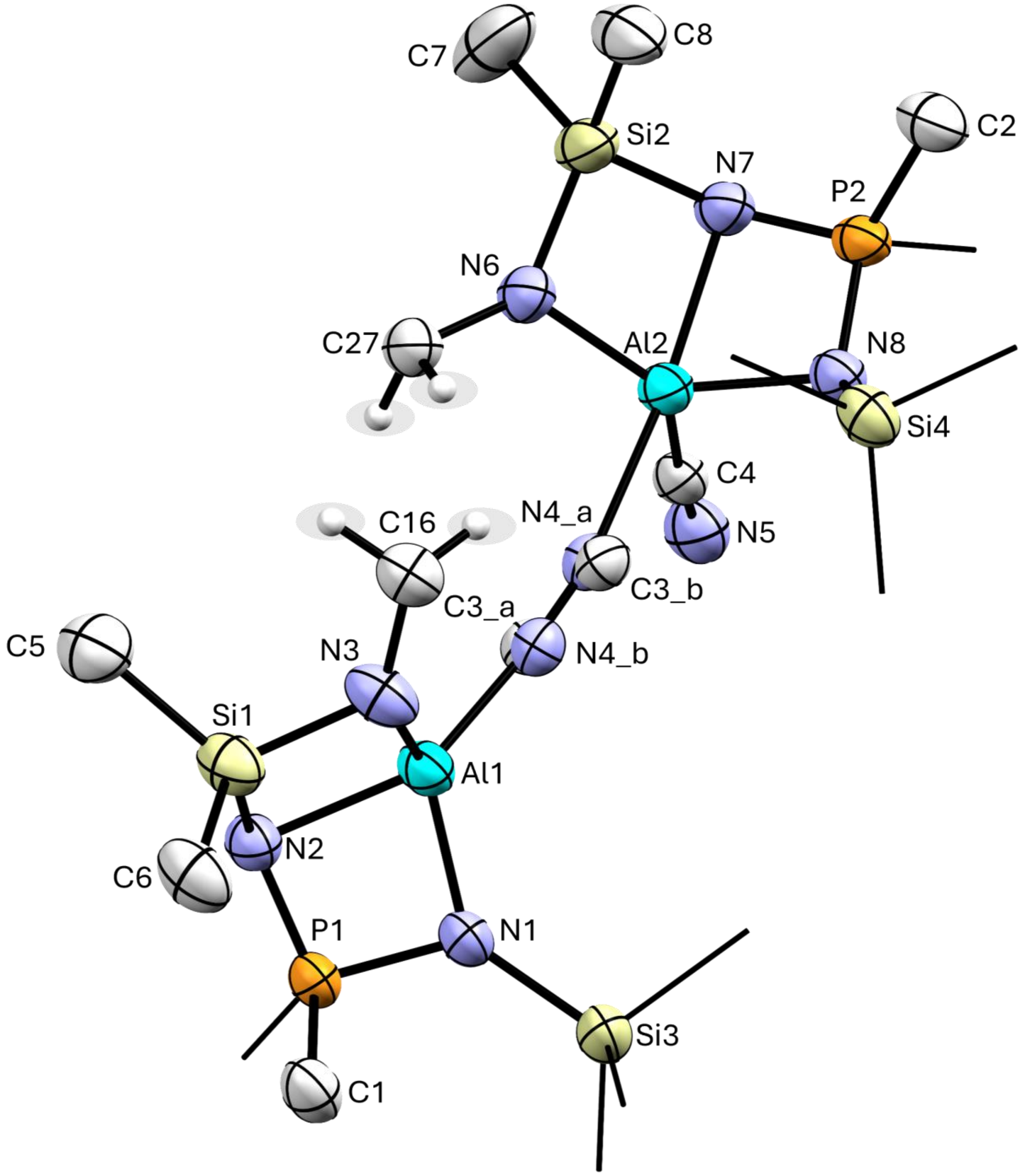

Figure S14: Molecular structure of **6P^H^**. Ellipsoids are set at 50% probability (100(2) K) with H-atoms and disordered MePNSi(Me$_3$)$_3$ part omitted. Both of the Dipp-groups, the Me-groups of Cp* and Si(Me$_3$)$_3$ rendered as wire-frame for clarity. Selected bond lengths [Å] and angles [°]: P1−C1 1.809(3), P1−N1 1.616(2), P1−N2 1.6121(18), Si2−N2 1.7065(18), Si2−N3 1.796(2), Al1−N1 1.968(2), Al1−N2 1.953(2), Al1−N3 2.151(2), Al1−C3_b 1.67(4), Al1−N4_b 1.89(2), Al1−N5_a 1.985(7), Al1−C2_a 2.06(3), C2_a−N4_a 1.11(3), N4_b−C2_b 1.17(3), N5_a−C3_13 1.069(14), C3_b−N5_b 1.33(5); N2−P1−N1 98.81(10), N2−Si2−N3 89.82(9), N2−Al1−N1 77.37(8), N5_a−Al1−C2_a 107.1(9), C3_b−Al1−N4_b 95.3(2); N2−P1−N1−Al1 4.6(1), N3−Si2−N2−Al1 28.71(9).

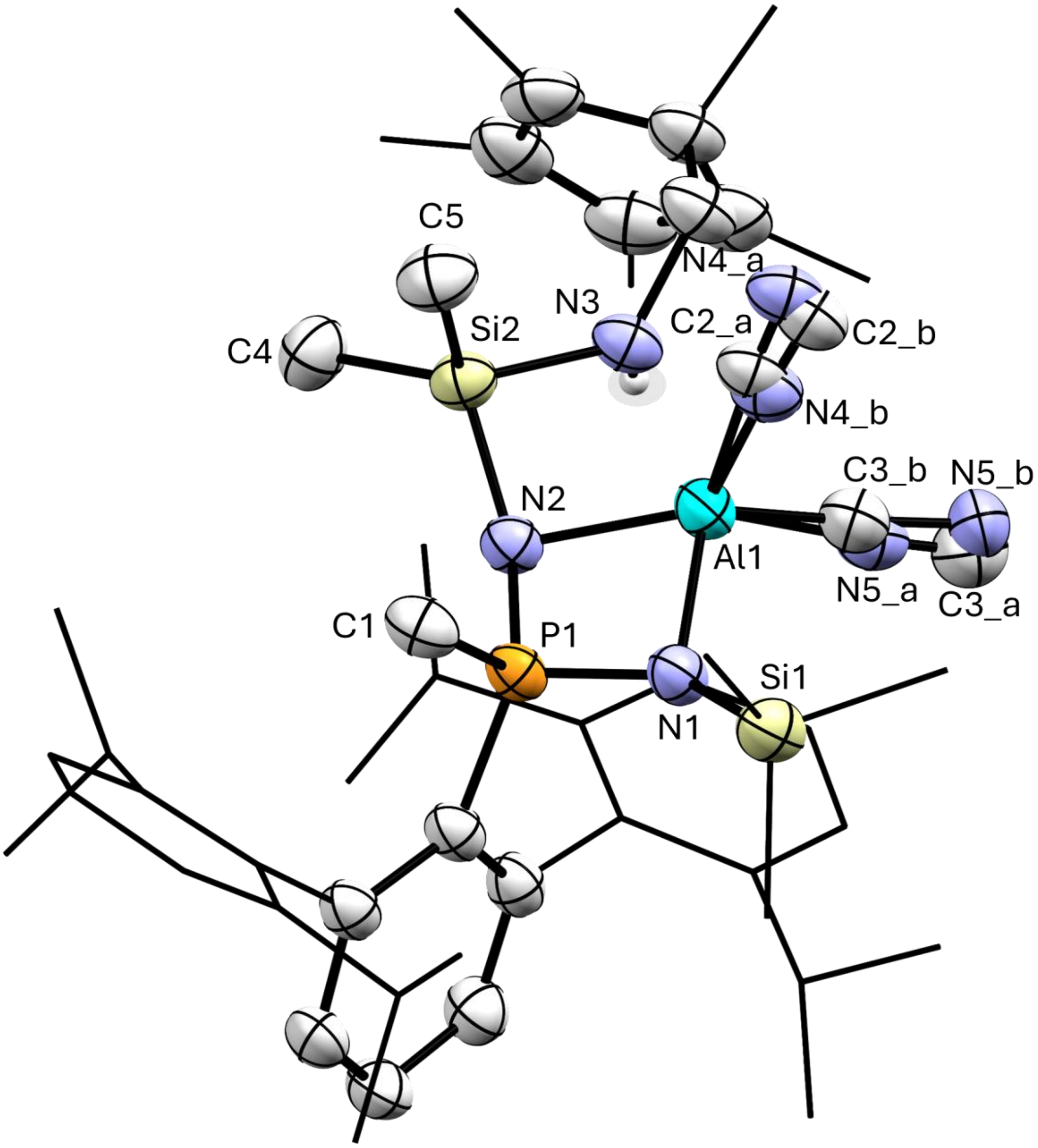

Figure S15: Molecular structure of **7P**. Ellipsoids drawn at 50% (100(2) K) with two pentane molecules, one toluene and H-atoms omitted with exception at C27. Both Dipp-groups and the Me-groups of Cp* rendered as wireframe for clarity. Selected bond lengths [Å] and angles [°]: P1−N1 1.647(2), P1−N6 1.653(2), P1−C27 1.824(2), Al1−N4 1.806(2), Al1−N6 1.952(2), Al1−N1 1.962(2), N1−N2 1.364(2), N2−N3 1.272(2); N1−P1−N6 92.29(7), N6−Al1−N1 74.87(6), P1−N1−Al1 95.94(7), N3−N2−N1 112.58(14), P1−N6−Al1 96.14(7), N6−P1−N1−Al1 7.04(7).

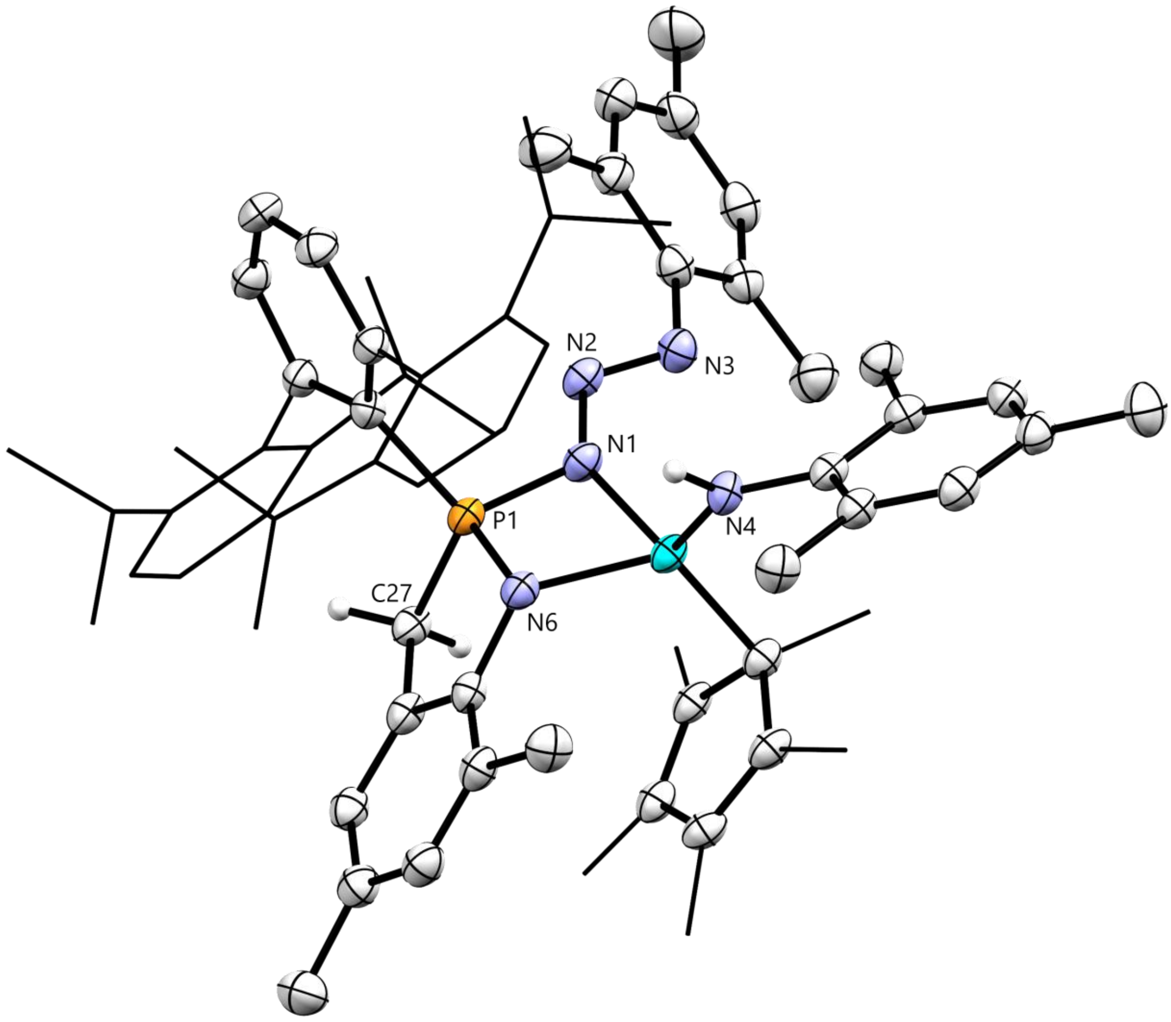

Figure S16: Molecular structure of **^{Dipp}TerAs(NPh)$_2$**. Ellipsoids are set at 50% probability (150(2) K) with H-atoms omitted. Both Dipp-groups and the Me-groups of Cp* rendered as wireframe for clarity. Selected bond lengths [Å] and angles [°]: N1−As1 1.833(2), N2−As1 1.861(1), As1−C1 1.970(1), C31−N1 1.408(2), C37−N2 1.395(2); N1−As1−N2 101.42(7), N1−As1−C1 102.60(6), N2−As1−C1 93.11(6), C31−N1−As1 121.9(1), C37−N2−As1 122.6(1); C31−N1−As1−N2 103.67(14), C31−N1−As1−C1 −160.49(13), C37−N2−As1−N1 −97.34(14), C37−N2−As1−C1 159.13(14).

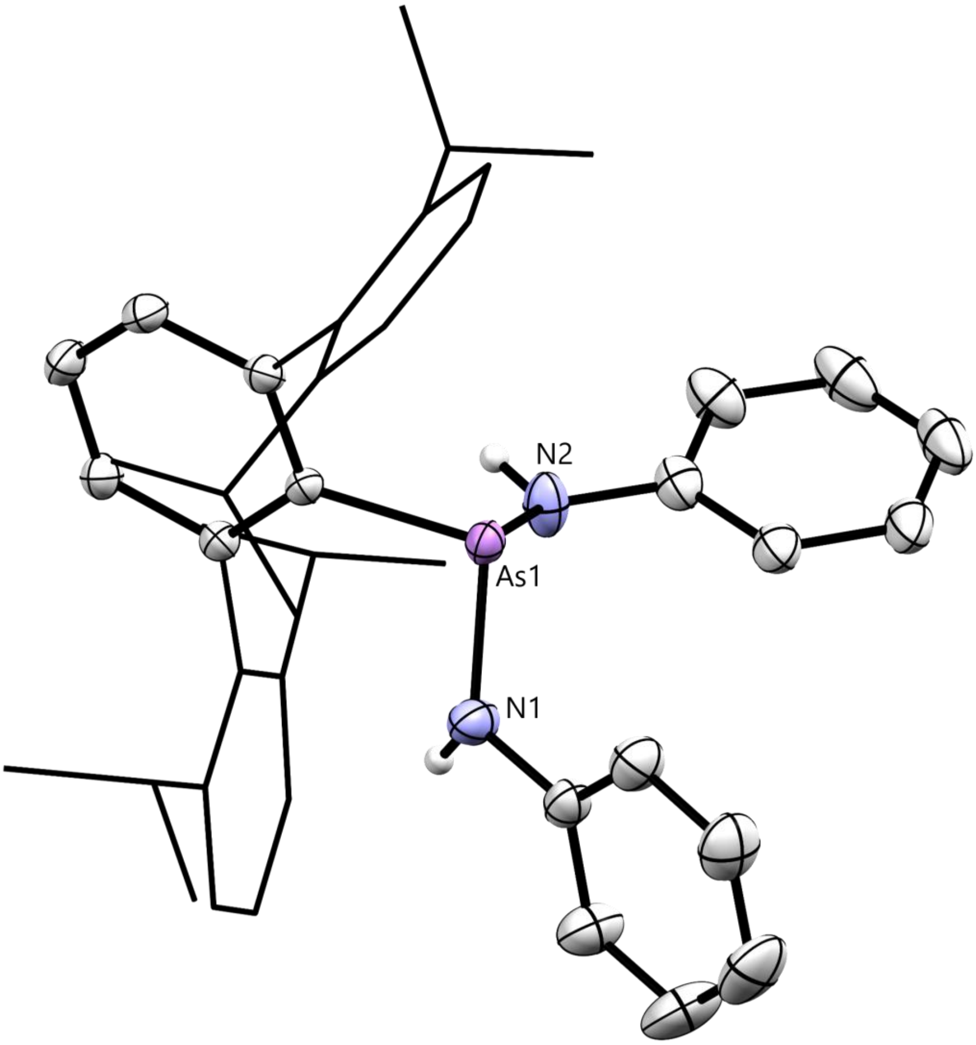

3 Syntheses of starting materials

3.1 Cp*K

$$\text{Cp*H} \xrightarrow{\text{KH}} \text{Cp*K}$$

*Cp*K was synthesized according to a slightly modified literature procedure.*[88]

KH (6.19 g, 154 mmol) was suspended in toluene (75 mL) in a Schlenk flask equipped with a pressure release valve. The suspension was cooled to −78 °C (CO_2/EtOH cooling bath) and a solution of Cp*H (26.3 g, 193 mmol) in toluene (75 mL) was added by cannular transfer. Afterwards the cooling bath was removed, and the reaction mixture was stirred over night at ambient temperature. At the next day the reaction mixture was placed in an oil bath and was heated to 50 °C for 3 days. *Attention: The addition of Cp*H leads to the evolution of H_2! The gas evolution rate should be finished after 3 days of reaction time.* The suspension was transferred to a large frit (see figure 16, top) by using a bend glass tube. The solvent was removed by filtration, and the colourless solid on the frit was washed five times with toluene (20 mL each) and afterwards three times with *n*-hexane (40 mL each). The residual colourless solid was dried on the frit *in vacuo* (1×10^{-3} mbar) over a period of 60 min by placing two heat guns (set to 80 °C) under the frit (see figure 16, bottom). During the drying process, the frit was shacked from time to time to enable uniformly drying. The product was collected without further purification as a fluffy colourless powder. *Attention: Cp*K is pyrophoric.* Yield: 23.6 g (136 mmol, 87%).

Figure S17: Dimensions of the glass frit, that was used to collect Cp*K (*top*). To dry the product on the frit two heat guns (set to 80 °C) were placed under it (*bottom*).

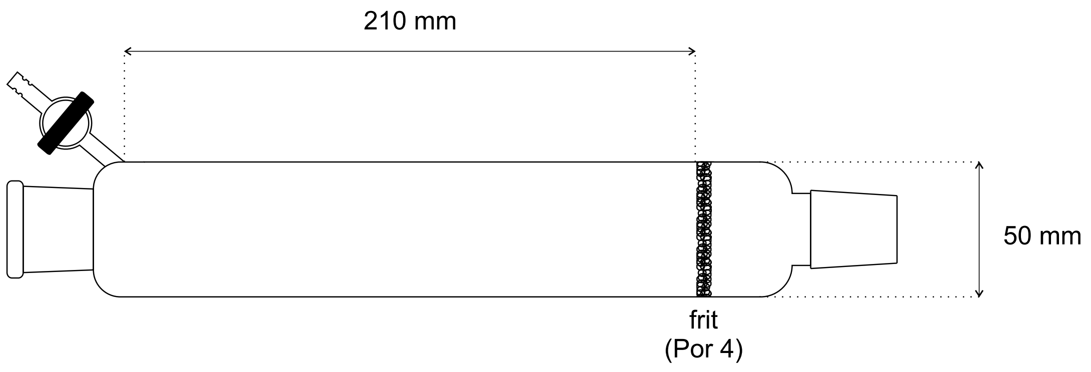

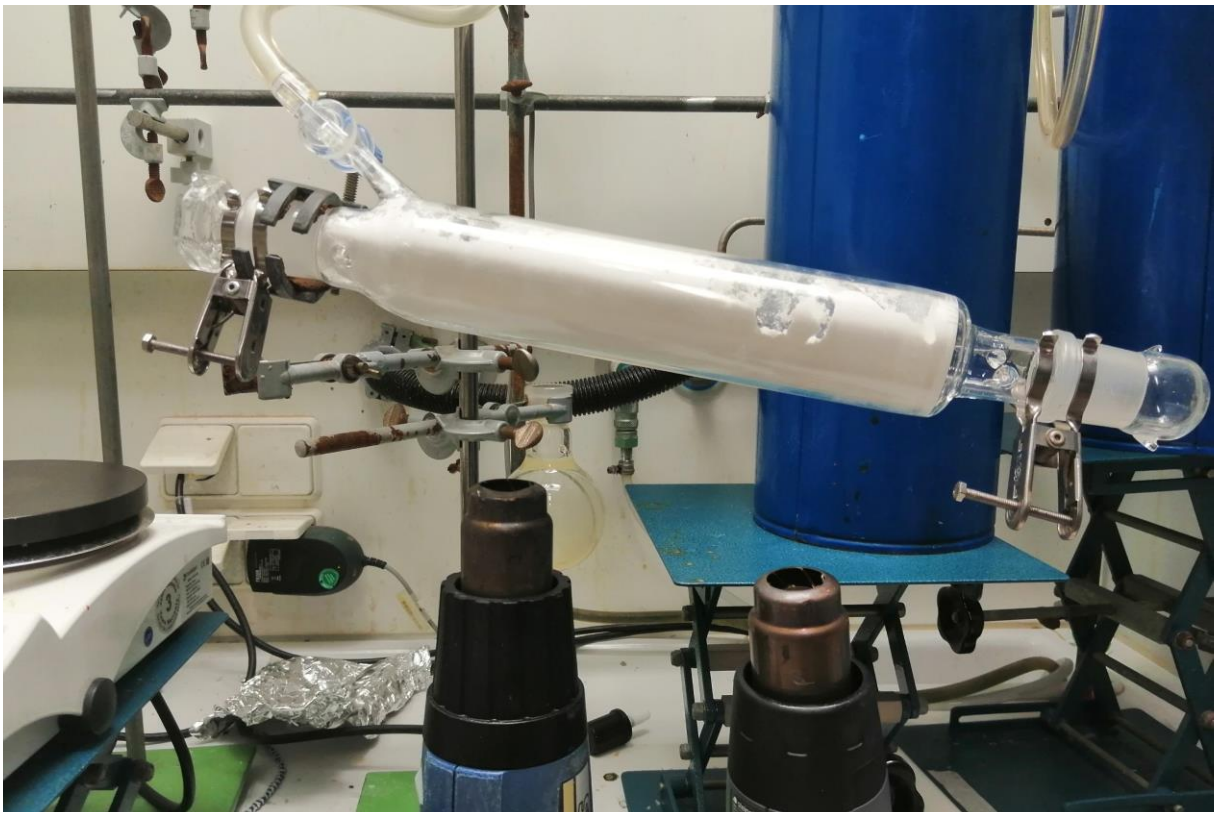

3.2 {Cp*Al}$_4$

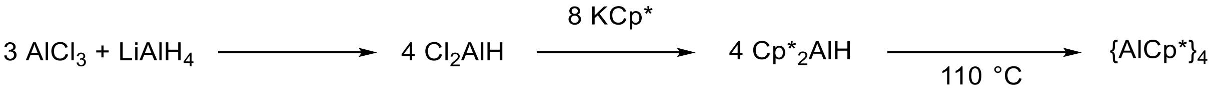

*(Cp*Al)$_4$ was synthesized according to slightly modified literature procedures.*[88,89]

$AlCl_3$ (2.87 g, 21.5 mmol) was suspended in pre-cooled (−78 °C) Et_2O (125 mL). The suspension was allowed to warm up until $AlCl_3$ dissolved completely. The solution was cooled to −78 °C ($EtOH/CO_2$ cooling bath) and a solution of $LiAlH_4$ in Et_2O (1 mol/L, 7.2 mL, 7.2 mmol) was added over a period of 2 min. The cooling bath was removed, and the resulting colorless suspension was stirred for 2.5 h at ambient temperature. The solid was removed by cannular filtration and the filtrate (a solution of Cl_2AlH) was directly dropped into a suspension of Cp*K (10.0 g, 57.4 mmol) in Et_2O (100 mL) over a period of 90 min at ambient temperature. The suspension was stirred overnight. Afterwards, the solids were removed by cannular filtration, and the solids were extracted again with Et_2O (40 mL). The filtrates were combined, and the solvent was removed *in vacuo*. The remaining solid was dried at 1×10^{-3} mbar over a period of 30 min at 45 °C (water bath). *n*-Hexane (125 mL) was added, and remaining solids were removed by cannula filtration. The filtrate was concentrated *in vacuo* to ~30 ml and was stored at −30 °C (freezer) overnight to obtain colorless crystals of $Cp*_2AlH$. The supernatant was removed by syringe was concentrated to get further crops of crystals, in the same way as described above. The crystals were dried *in vacuo* (1×10^{-3} mbar) for 30 min at ambient temperature. In a typical reaction 6.18 g (20.7 mmol, 72%) of $Cp*_2AlH$ were collect, distributed over three fractions. All $Cp*_2AlH$ was used without further purification for the final conversion to (AlCp*)$_4$.

The $Cp*_2AlH$ was placed in a 50 mL Schlenk flask and was dissolved in toluene (12 mL). The solution was degassed (freeze-pump-thaw) and was heated to 110 °C (oil bath) over a period of 30 min, whereby {AlCp*}$_4$ precipitated as yellow microcrystalline solid. The supernatant was removed by cannula filtration, and the solid residue was extracted again with boiling *n*-hexane (20 mL). The remaining yellow product was dried *in vacuo*

(1×10^{-3} mbar) at 50 °C (water bath) over a period of 30 min. Filtrate and wash solution were combined and all volatile compounds were removed *in vacuo*. The residue contained unreacted $Cp^*{}_2AlH$, that was used to synthesized further amounts of $(AlCp^*)_4$. Therefore, the decomposition reaction was proceeded as mentioned above by using 5 mL toluene for the reaction and 10 ml of hexane for the final extraction. Yield: 2.72 g (4.19 mmol, 58%).

CHN calc. (found) in %: C 74.05 (66.99), H 9.32 (8.72); deviations probably due to incomplete combustion, repeated measurements with and without adding V_2O_5 as additional oxidation reagent did not result in better agreement. **^{1}H NMR** (400.1 MHz, C_6D_6, 298 K): δ = 1.91 (s, 15 H, $C_5(CH_3)$) ppm. **IR** (ATR, 32 scans, cm^{-1}): $\tilde{\nu}$ = 2972 (m), 2902 (s), 2853 (s), 2719 (m), 1490 (m), 1428 (s), 1373 (vs), 1062 (w), 1020 (m), 950 (w), 800 (m), 596 (m), 585 (m). **MS** (CI, pos., isobutene, m/z): 325 $[Cp^*{}_2Al_2+H]^+$, 163 $[Cp^*Al+H]^+$, 137 $[Cp^*+2H]^+$.

Figure S18: ^{1}H NMR spectrum of $(Cp^*Al)_4$ (400.1 MHz, C_6D_6, 298 K).

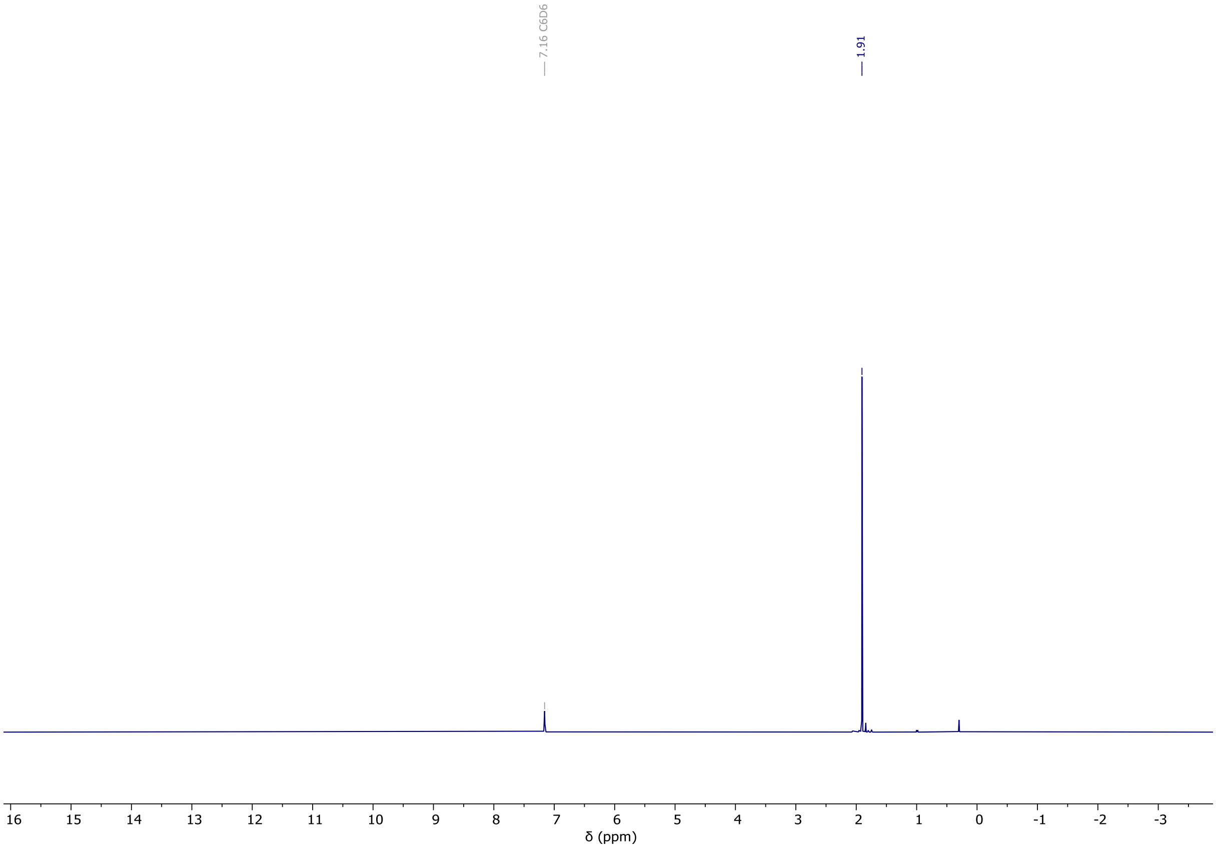

Figure S19: IR spectrum of $(Cp^*Al)_4$ (ATR, 32 scans, cm^{-1}, powder).

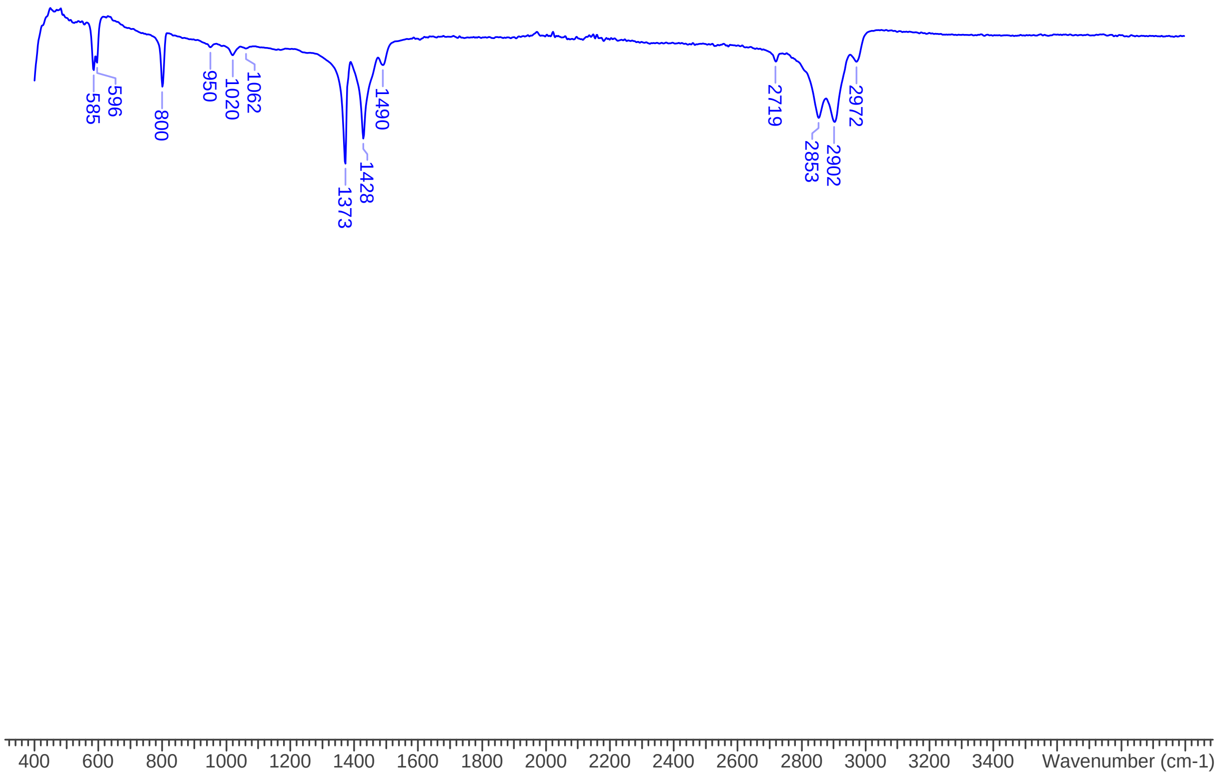

3.3 DippBr

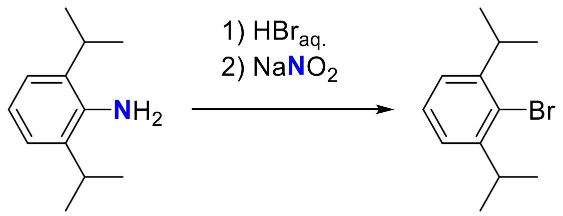

DippBr was synthesized according to a slightly modified literature procedure.[90]

The whole procedure was done under *non-inert* conditions. $HBr_{aq.}$ (47%, 500 mL, 4.45 mol) was placed in a 2 L round bottom flask equipped with an overhead stirrer. The solution was cooled to approx. 0 °C (ice bath) and to the stirred solution, $DippNH_2$ (115 g, 0.649 mol) was added over a period of 5 min. The resulting suspension was cooled to −78 °C (CO_2/EtOH cooling bath) and was diluted by the addition of Et_2O (100 mL). Afterwards, $NaNO_2$ (80.0 g, 1.16 mol) was added to the stirred reaction

mixture in small portions by a spatula over a period of 70 min at −78 °C. (*Attention: Upon addition of $NaNO_2$ the diazonium salt $DippN_2^+Br^−$ will be generated. This requires a strict temperature control. A magnetic stir bar would not be sufficient due to the high viscosity of the reaction mixture*.) The reaction mixture was allowed to warm to ambient temperature in the cooling bath overnight. $HCl_{aq.}$ (37%, 20 mL, 0.24 mol) was added at ambient temperature and all solids were removed by filtration by using a funnel with a sintered glass disc. The solid was washed twice with Et_2O (50 mL each) and the solid was discarded. Filtrate and washing solutions were combined in a separation funnel. Organic and aqueous phase were separated. The aqueous phase was neutralised by adding Na_2CO_3 (~200 g) (*Attention: Gas evolution!*) followed by the addition of $Na_2S_2O_3$ (15.0 g, 0.949 mol). Afterwards, the aqueous and organic phase were carefully re-combined in a separation funnel (*Attention: Gas evolution!*) and were blended rigorously. The organic phase was separated, and the aqueous phase was re-extracted twice with Et_2O (300 mL each). The organic phases were combined and dried over $MgSO_4$. The drying agent was removed by filtration and the filtrate was concentrated by using a rotary evaporator, until a brown oil remained. The oil was purified by fractional distillation at reduced pressure (1×10^{-3} mbar) by using an apparatus consisting of a Liebig condenser, a short Vigreux column, distilling receiver (Bredt-type) and an oil bath as heating source (T_{oil} = 105 °C). The product was collected in the first fraction with a boiling point of 50 °C. *Note: The second fraction which does not contain the intended product has almost the same boiling point. However, it was easily possible to differentiate between the two fractions due because the first fraction was colourless, whereby the second fraction was yellow. It was sufficient to collect the first fraction up to the point in time where the first yellow drop of condensate was formed in the Liebig condenser*. DippBr was stored in the absence of light under *non-inert* conditions. Yield: 78.9 g (0.649 mol, 69%).

^{1}H NMR (400.1 MHz, $CDCl_3$, 298 K): δ = 1.25 (d, $^3J_{(H\text{-}H)}$ = 6.8 Hz, 12 H, C(H)(CH_3)$_2$), 3.51 (hept, $^3J_{(H\text{-}H)}$ = 6.8 Hz, 2 H, C(H)(CH_3)$_2$), 7.11 – 7.15 (m, 2 H, *m*-C*H*), 7.22 – 7.27 (m, 1 H, *p*-C*H*) ppm. **^{13}C{^{1}H} NMR** (100.6 MHz, $CDCl_3$, 298 K): δ = 23.2 (s, C(H)(CH_3)$_2$), 33.7 (s,

$C(H)(CH_3)_2$),124.3 (s, *m*-CH), 126.7 (s, *C*-Br), 127.5 (s, *p*-CH), 147.9 (s, *C*-iPr) ppm. **IR** (ATR, 32 scans, cm^{-1}): $\tilde{\nu}$ = 3061 (vw), 2963 (s), 2928 (m), 2887 (w), 2869 (w), 1684 (vw), 1584 (w), 1521 (vw), 1507 (vw), 1460 (m), 1417 (m), 1384 (m), 1362 (m), 1342 (w), 1321 (w), 1303 (w), 1250 (w), 1197 (vw), 1181 (w), 1162 (w), 1150 (vw), 1123 (w), 1107 (w), 1056 (m), 1013 (s), 966 (vw), 928 (w), 895 (vw), 885 (vw), 865 (vw), 789 (vs), 761 (vw), 728 (vs), 632 (w), 602 (m), 567 (vw), 534 (vw), 504 (w), 473 (vw), 442 (vw). **MS** (EI, 70 eV, m/z, rel. int. > 10%): 242 (34) $[M]^+$, 240 (33) $[M]^+$, 228 (12) $[M-CH_3]$, 227 (94) $[M-CH_3]$, 226 (14) $[M-CH_3]$, 225 (100) $[M-CH_3]$, 131 (10), 129 (10), 128 (15), 117 (29), 115 (22), 91 (19).

Figure S20: ^{1}H NMR spectrum of DippBr (400.1 MHz, $CDCl_3$, 298 K).

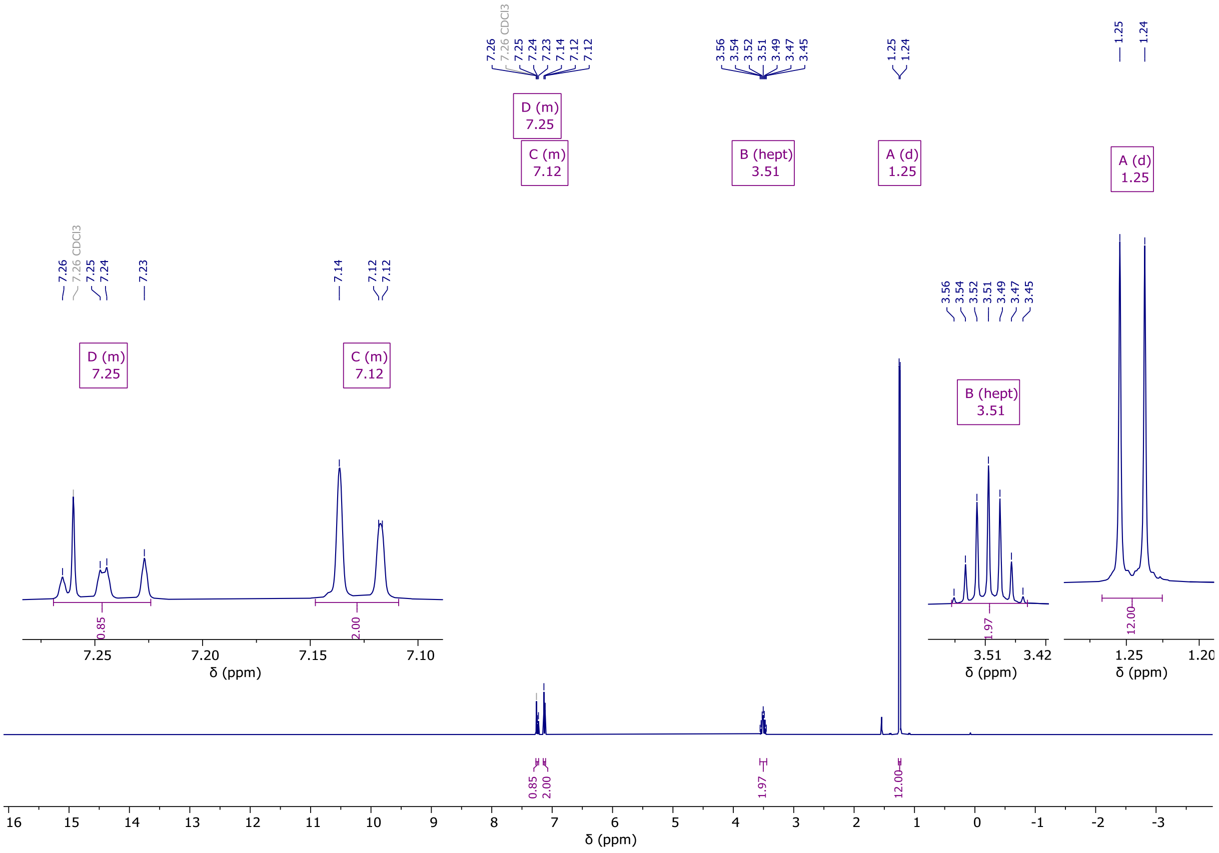

Figure S21: $^{13}C\{^{1}H\}$ NMR spectrum of DippBr (100.6 MHz, $CDCl_3$, 298 K).

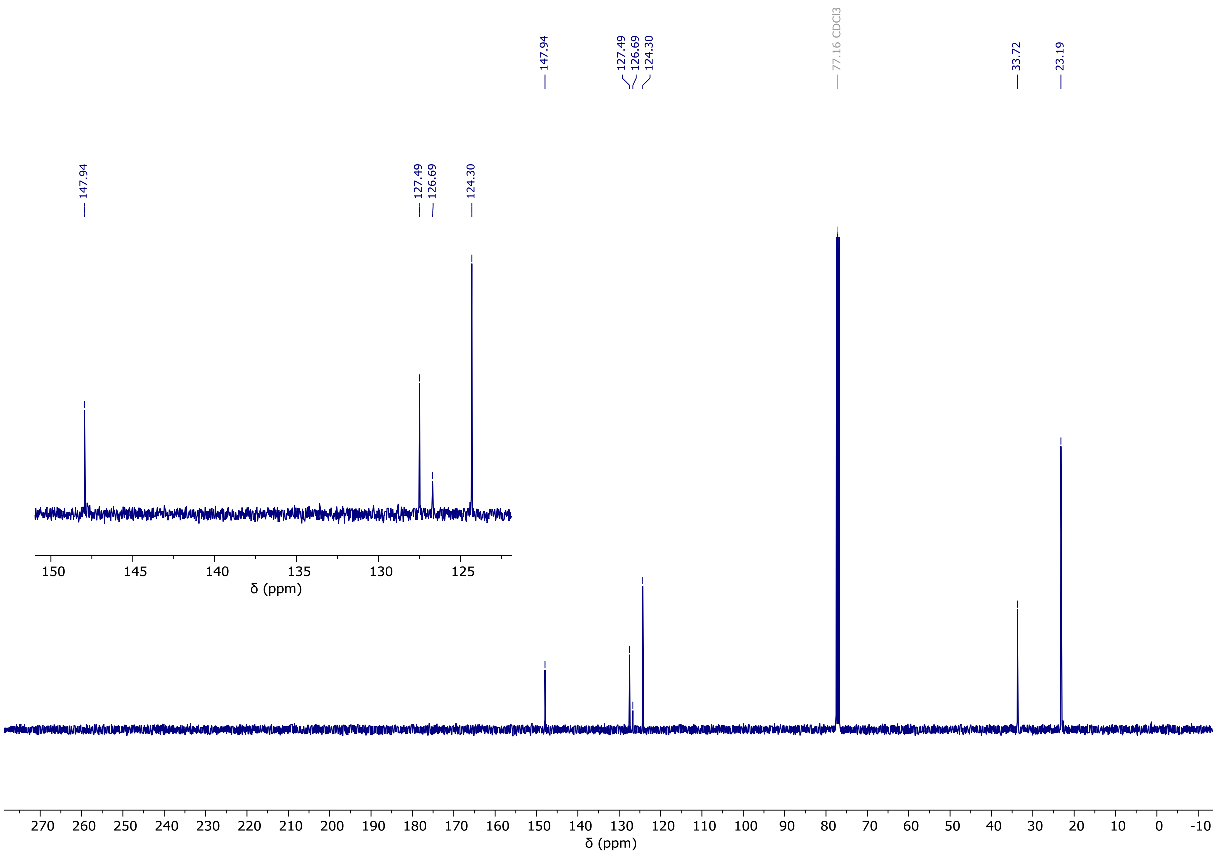

Figure S22: IR spectrum of DippBr ((ATR, 32 scans, cm^{-1}, liquid).

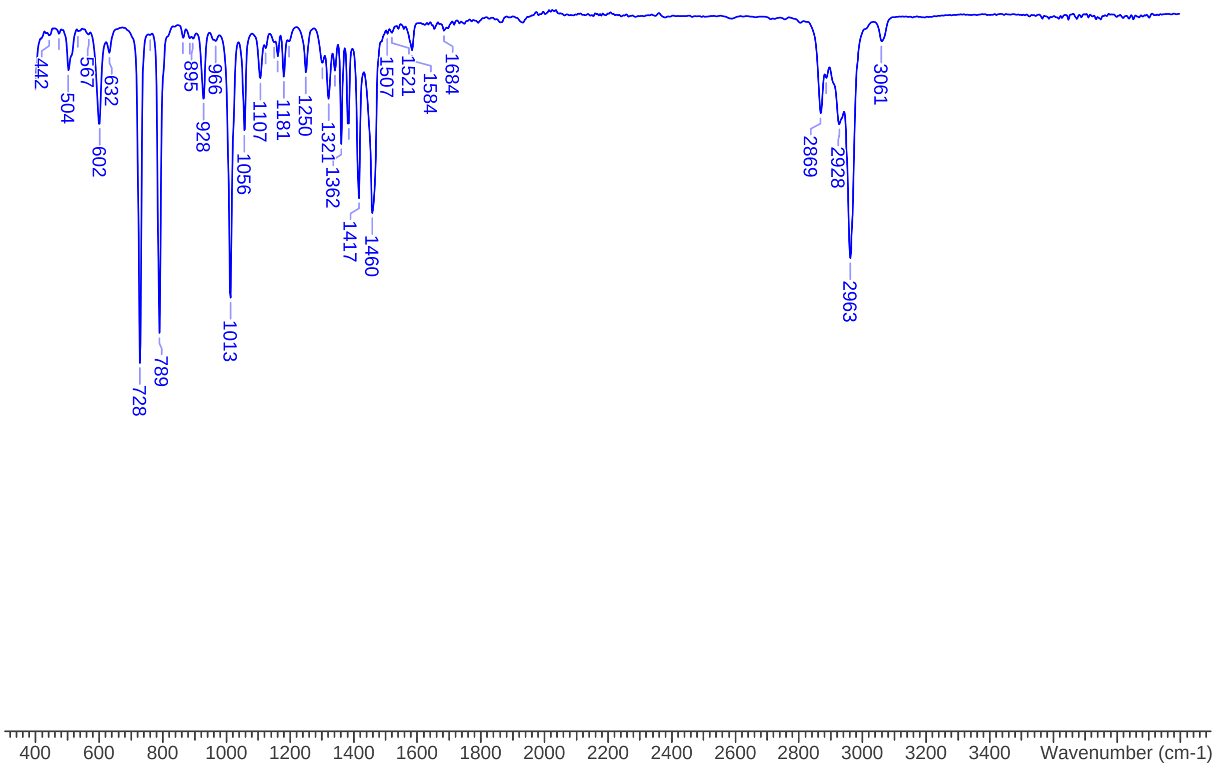

3.4 ^{Dipp}TerI

a)

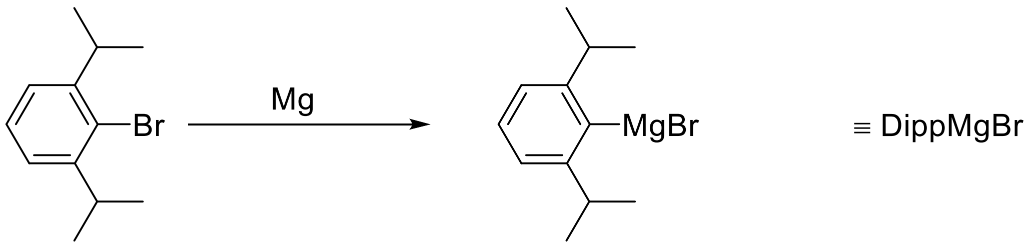

b)

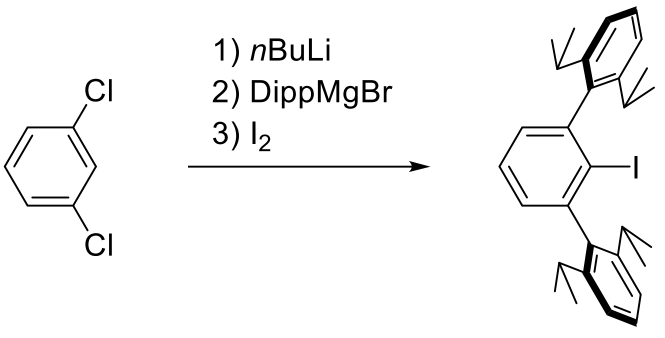

The synthesis of ^{Dipp}TerI, starting from 1,3-dichlorobenzene has already been reported. [91] *In the context of this publication various modifications have been made that were adapted from the recently published procedure for the preparation of 2,6-dimesitylphenyliodide (^{Mes}TerI).*[92] The synthesis began with the preparation of a 2,6-diisopropylmagnesiumbromide (DippMgBr) solution, which was used in the further synthesis at the same day.

a) Mg turnings (12.3 g, 506 mmol) were suspended in THF (250 mL) in a 1 L three-necked flask equipped with a long reflux condenser (length cooling coil: 40 cm) and a pressure relief valve. A solution of DippBr (110.9 g, 460 mmol) in THF (100 mL) was slowly added at ambient temperature *via* a dropping additionfunnel, until a color change to brown and warming of the reaction mixture was observed.[i] At that time, an ice bath was placed under the flask and the remaining DippBr solution was added over a period of 30 min. The ice bath was removed and the Grignard solution was carefully

[i] If the Gringnard reaction does not start within the addition of the first 50 mL of the DippBr solution, a small piece of I_2 can be added and/or the reaction mixture can be heated with a heatgun until the reaction mixture starts to reflux.

warmed to ambient temperature. The reaction mixture was refluxed for 2 h (heating mantle) and was subsequently cooled to ambient temperature. Deviation of the reflux time of +1 h had no effect on the following reaction steps and were sometimes necessary due to delays in the lithiation of 1,3-dichlorobenzene which was carried out simultaneously:

b) *This reaction part was performed parallel to reaction part* ***a)*** *(above) and was started when the Grignard solution began to reflux.* 1,3-dichlorobenzene (26.6 g, 181 mmol) was dissolved in THF (600 mL) and cooled down to −78 °C (CO_2/EtOH cooling bath). *n*BuLi (73 mL, 2.5M in *n*-hexane, 183 mmol) was added over a period of 30 min. The reaction mixture was stirred for 10 min at −78 °C and then the Grignard solution from part **a)** was added *via* cannula transfer over a period of 20 min at −78 °C. The reaction was warmed to ambient temperature overnight under stirring. On the next day, the reaction mixture was refluxed for 2 h (heating mantle) and subsequently cooled down to ambient temperature. I_2 (84.6 g, 333 mol) was added in portions until the reaction mixture stayed consistently brown (for approx. 15 min). The work-up proceeded under non-inert conditions. Therefore, the reaction was quenched by adding a solution of Na_2SO_3 (37.7 g, 299 mmol) in water (300 mL). The aqueous phase was separated and extracted 3 times with Et_2O (200 mL each). The organic phases were combined, and all volatile compounds were removed until an orange oil remained by using a rotary evaporator. To the oil was added EtOH (60 ml) and colorless crystals formed within 20 min by standing at ambient temperature. The crystals were separated by using a funnel filter with sintered glass disc and were washed with cold EtOH (−110 °C) until the product remained as colorless solid. Mother liquor and washing solution were combined and concentrated to get further crops of crystals. The product was dried in vacuo (1×10−3 mbar) at 50 °C (water bath) for 2 h. Yield: 57.2 g (109 mmol, 60%)

CHN calc. (found) in C 68.70 (68.55), H 7.11 (6.96). **^{1}H NMR** (400.1 MHz, C_6D_6, 298 K): δ = 1.10 (d, $^3J_{(H\text{-}H)}$ = 6.9 Hz, 12 H, C(H)(C*H*$_3$)(CH$_3$)), 1.36 (d, $^3J_{(H\text{-}H)}$ = 6.9 Hz, 12 H, C(H)(CH$_3$)(C*H*$_3$)), 2.74 (ψ-hept, $^3J_{(H\text{-}H)}$ = 6.9 Hz, 4 H, C(*H*)(CH$_3$)(CH$_3$)), 6.99-7.02 (m, 2 H,

m-C*H* (^{Dipp}Ter)), 7.04-7.08 (m, 1 H, *p*-C*H* (^{Dipp}Ter)), 7.20 (m, 4 H, *m*-C*H* (Dipp)), 7.34 (m, 2 H, *p*-C*H* (Dipp)) ppm. **^{13}C{^{1}H} NMR** (100.6 MHz, C_6D_6, 298 K): δ = 23.7 (s, C(H)(CH_3)(CH_3)), 25.1 (s, C(H)(CH_3)(CH_3)), 31.3 (s, *C*(H)(CH_3)(CH_3)), 110.6 (s, *C*-I), 123.3 (s, *m*-CH (Dipp)), 127.7 (s, *p*-CH (^{Dipp}Ter)), 128.5 (s, *m*-CH (^{Dipp}Ter)), 129.1 (s, *p*-CH (Dipp)), 143.0 (s, *i*-*C* (Dipp), 146.2 (s, *C*-*i*Pr), 147.3 (s, *C*-Dipp) ppm. **IR** (ATR, 32 scans, cm^{-1}): $\tilde{\nu}$ = 3067 (w), 2958 (s), 2925 (m), 2906 (m), 2865 (m), 1593 (vw), 1577 (w), 1461 (m), 1447 (m), 1433 (m), 1381 (m), 1360 (m), 1346 (w), 1327 (w), 1307 (w), 1251 (w), 1179 (w), 1160 (vw), 1121 (vw), 1105 (w), 1084 (vw), 1057 (m), 1041 (w), 1018 (w), 1002 (w), 938 (w), 901 (vw), 818 (w), 802 (m), 789 (s), 756 (vs), 738 (m), 686 (w), 633 (vw), 583 (w), 546 (w), 528 (vw), 466 (w), 453 (m). **MS** (CI, pos., isobutene, m/z): 526 [M+2 H]$^{+}$.

Figure S23: ^{1}H NMR spectrum of ^{Dipp}TerI (400.1 MHz, C_6D_6, 298 K).

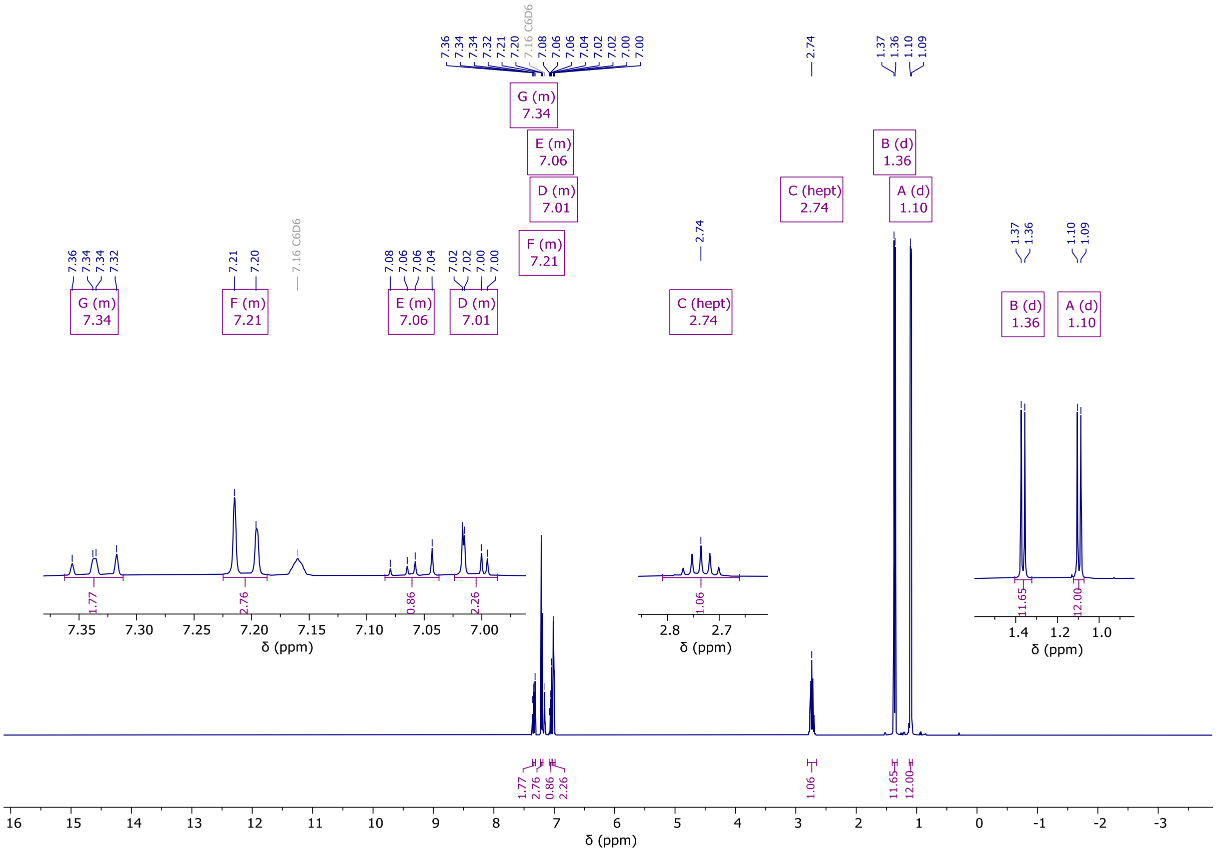

Figure S24: $^{13}C\{^{1}H\}$ NMR spectrum of ^{Dipp}TerI (100.6 MHz, C_6D_6, 298 K).

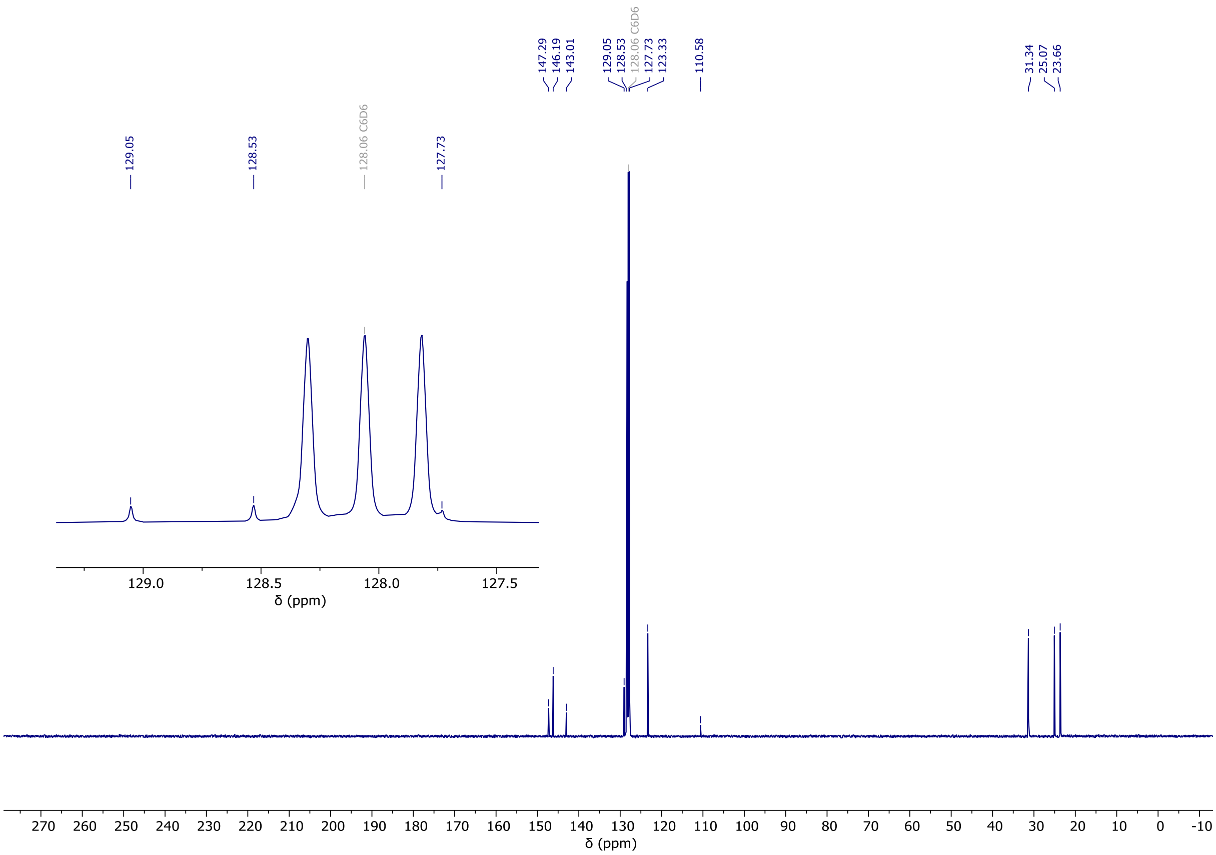

Figure S25: IR spectrum of ^{Dipp}TerI (ATR, 32 scans, cm^{-1}, powder).

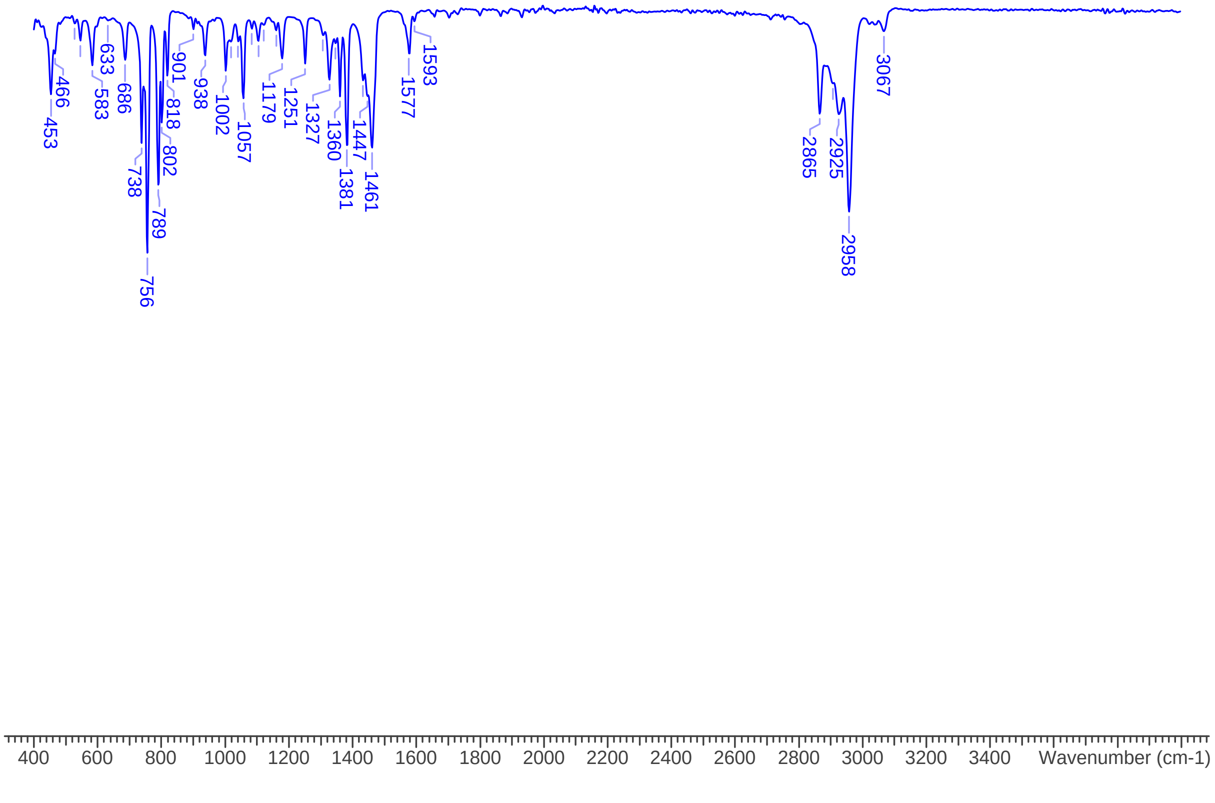

3.5 ^{Dipp}TerPCl$_2$

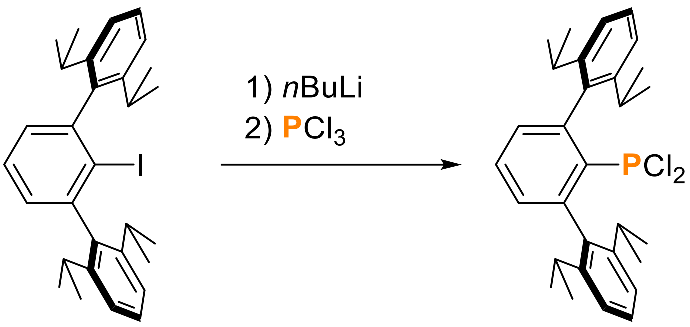

^{Dipp}TerPCl$_2$ was synthesized according to a slightly modified literature procedure.[93]

To a solution of ^{Dipp}TerI (15.0 g, 28.6 mmol) in *n*-hexane (100 mL), *n*BuLi (12.6 mL, 2.5 M in *n*-hexane, 31.5 mmol) was added over a period of 5 min at −78 °C (CO_2/EtOH cooling bath). The reaction mixture was allowed to warm to ambient temperature in the cooling bath overnight. A colorless solid formed and was allowed to settle, and the supernatant solution was removed by canula filtration. The remaining solid was washed twice with *n*-hexane (20 mL each) and was subsequently dried *in vacuo* (1×10^{-3} mbar) for 15 min at 45 °C (water bath). The solid was then suspended in toluene (100 mL) and was cooled to −78 °C (CO_2/EtOH cooling bath). At this temperature freshly degassed (freeze-pump-thaw) PCl_3 (neat, 4.85 g, 35.3 mmol) was added over a period of 5 min by syringe. The reaction mixture was warmed in the cooling bath to ambient temperature overnight. The solvent was removed under reduced pressure and the orange residue was dried *in vacuo* (1×10^{-3} mbar) for 3 h at 50 °C (water bath). The residue was suspended in *n*-hexane (150 mL) and the suspension was then placed in an ultrasound bath. The suspension was filtered hot (heat gun set to 80 °C until *n*-hexane started to reflux) over celite and the residue was extracted two more times with fresh *n*-hexane (20 mL each, at 80 °C).

The filtrate was stored overnight at ambient temperature whereby an orange by-product precipitated. The supernatant solution was decantated and concentrated (to approx. 20 mL) under reduced pressure in the warmth. The solution was allowed to cool down to ambient temperature overnight whereby large colourless crystals of

^{Dipp}TerPCl$_2$ where obtained. The supernatant was transferred to another flask and was used to get further fractions of crystalline product in an analogue manner. The isolated crystals were dried *in vacuo* (1×10^{-3} mbar) for 2 h at 60 °C (water bath)[ii] Yield: 8.52 g (28.6 mmol, 60%).

^{1}H NMR (400.1 MHz, C_6D_6, 298 K): δ = 1.01 (d, $^3J_{(H-H)}$ = 6.8 Hz, 12 H, C(H)(C*H*$_3$)(CH$_3$)), 1.30 (d, $^3J_{(H-H)}$ = 6.8 Hz, 12 H, C(H)(CH$_3$)(C$_3$)), 2.78 (*ψ*-hept, $^3J_{(H-H)}$ = 6.8 Hz, 4 H, C(*H*)(CH$_3$)(CH$_3$)), 6.99 – 7.12 (superimposed signals, 3 H, *m*- and *p*-C*H* (^{Dipp}Ter)), 7.14 – 7.17 (m, 4 H, *m*-C*H* (Dipp)), 7.31 (m, 2 H, *p*-C*H* (Dipp)) ppm. **^{13}C{^{1}H} NMR** (100.6 MHz, C_6D_6, 298 K): δ = 22.8 (s, C(H)(*C*H$_3$)(CH$_3$)), 25.7 (s, C(H)(CH$_3$)(*C*H$_3$)), 31.6 (s, *C*(H)(CH$_3$)(CH$_3$)), 123.1 (s, *m*-CH (Dipp)), 129.5 (s, *p*-CH (Dipp)), 130.9 (s, *p*-CH (^{Dipp}Ter)), 132.1 (s, *m*-CH (^{Dipp}Ter)), 136.1 (d, $^1J_{(C-P)}$ = 73 Hz, C-PCl$_2$), 136.6 (d, $^3J_{(C-P)}$ = 8 Hz, *i*-*C*(Dipp)), 145.8 (d, $^2J_{(C-P)}$ = 29 Hz, *o*-C (^{Dipp}Ter)), 147.3 (d, $^4J_{(C-P)}$ = 2 Hz, *o-C (Dipp)*) ppm. **^{31}P{^{1}H} NMR** (162.0 MHz, C_6D_6, 298 K): δ = 157.74 (s, *P*Cl$_2$) ppm. **IR** (ATR, 32 scans, cm^{-1}): $\tilde{\nu}$ = 3055 (vw), 2962 (m), 2927 (w), 2867 (w), 1591 (vw), 1577 (vw), 1560 (w), 1457 (w), 1445 (w), 1430 (w), 1383 (w), 1360 (w), 1325 (w), 1305 (vw), 1249 (w), 1177 (w), 1123 (vw), 1109 (w), 1080 (vw), 1070 (w), 1055 (w), 1039 (w), 936 (w), 822 (w), 806 (m), 794 (m), 759 (m), 750 (m), 701 (w), 606 (vw), 587 (w), 524 (vw), 501 (vs), 453 (vs), 416 (s). **MS** (CI, pos., isobutene, m/z): 503-500 [M+2 H]$^+$, 459-456 [M−C$_3$H$_7$]$^+$.

[ii] Sometimes further crystals of the orange side product were formed between the colorless crystals of the main product. These can be removed by crystal picking in the glovebox to get analytical pure compound. This step can be omitted for the subsequent synthesis of ^{Dipp}TerPPMe$_3$.

In cases were significant amounts of ^{Dipp}TerI are present after workup, a recrystallisation from a minimal amount of boiling THF was successful.

Figure S26: ^{1}H NMR spectrum of ^{Dipp}TerPCl$_2$ (400.1 MHz, C_6D_6, 298 K).

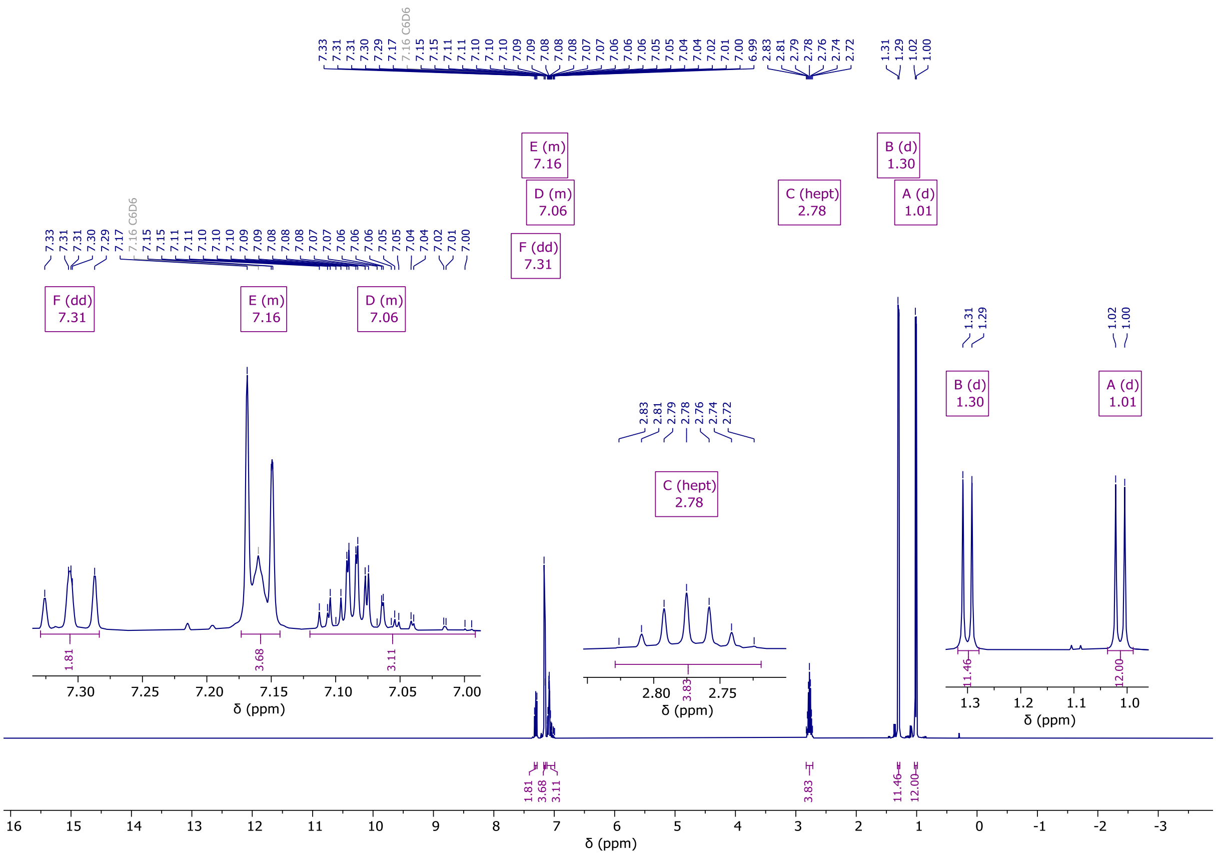

Figure S27: ^{31}P{^{1}H} NMR spectrum of ^{Dipp}TerPCl$_2$ (162.0 MHz, C_6D_6, 298 K).

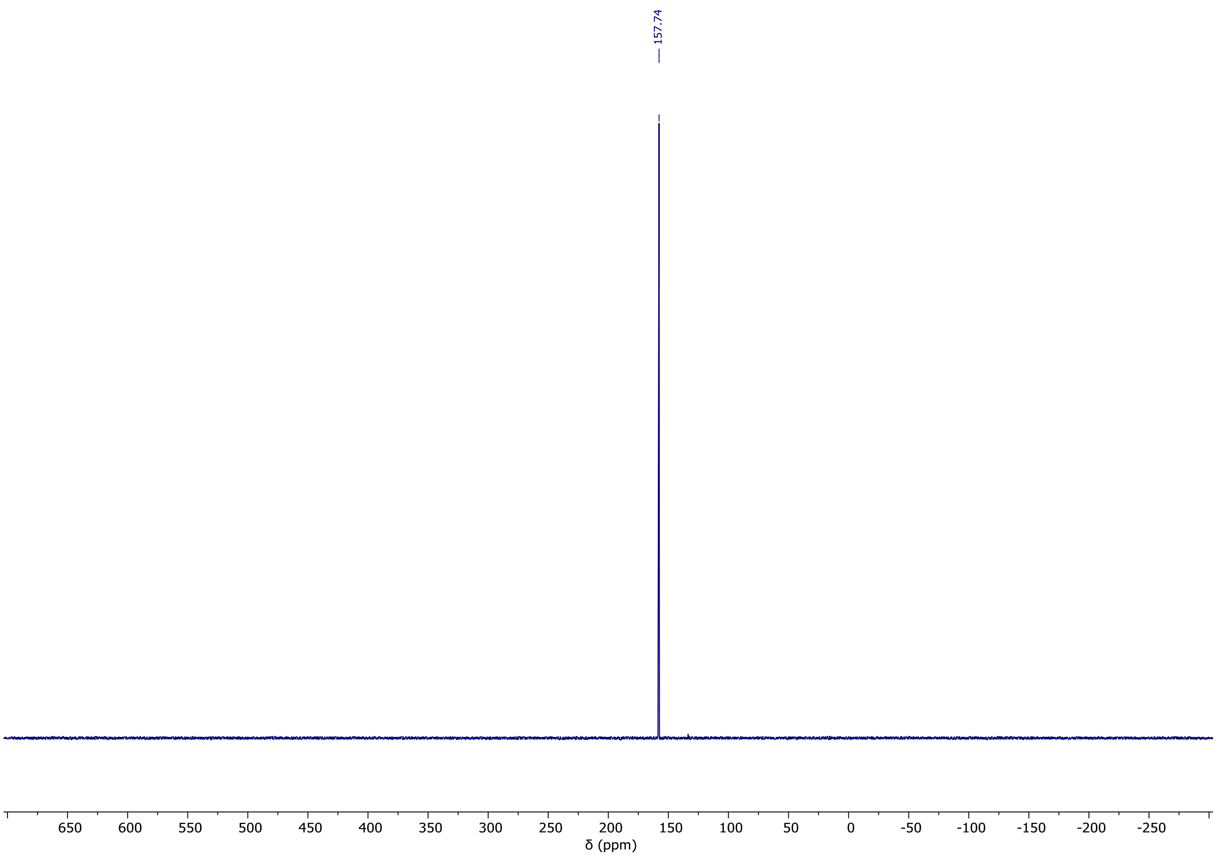

Figure S28: $^{13}C\{^{1}H\}$ NMR spectrum of $^{Dipp}TerPCl_2$ (100.6 MHz, C_6D_6, 298 K).

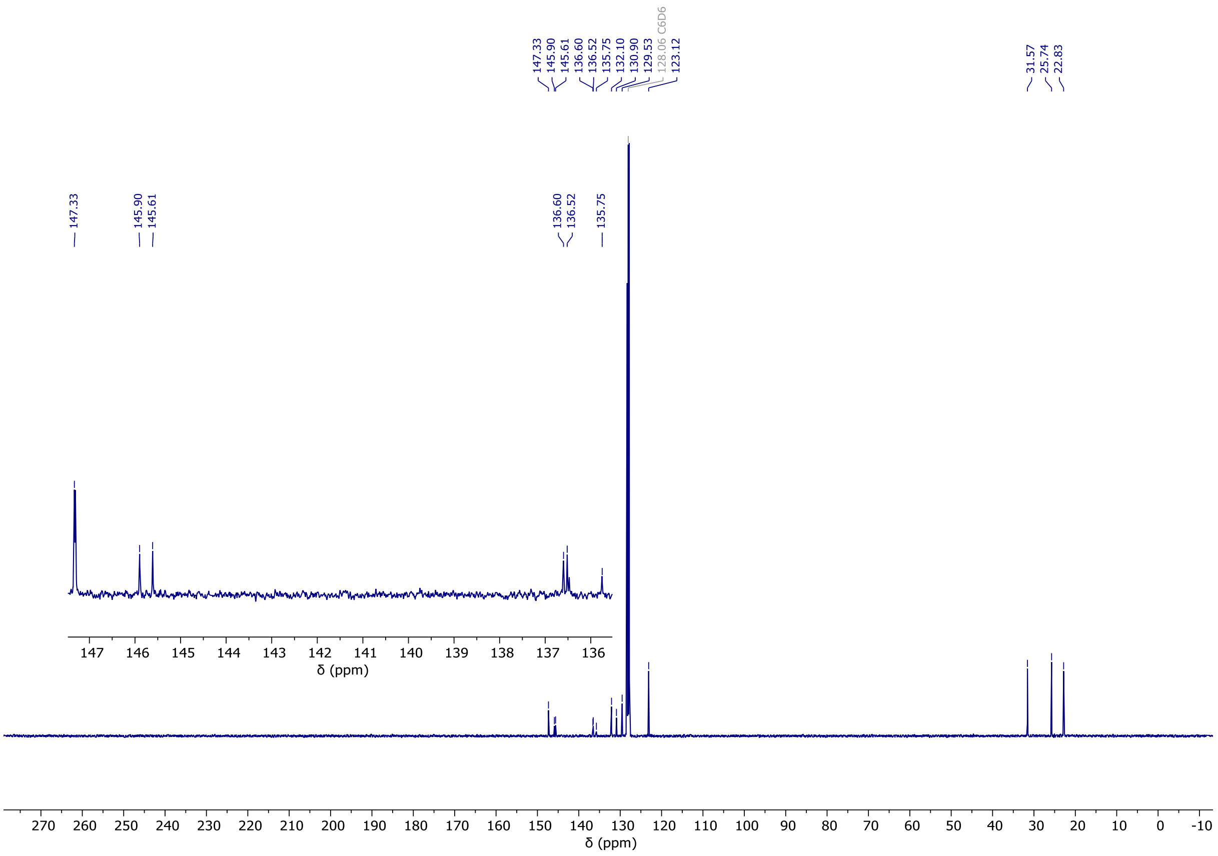

Figure S29: IR spectrum of $^{Dipp}TerPCl_2$ (ATR, 32 scans, cm^{-1}, powder).

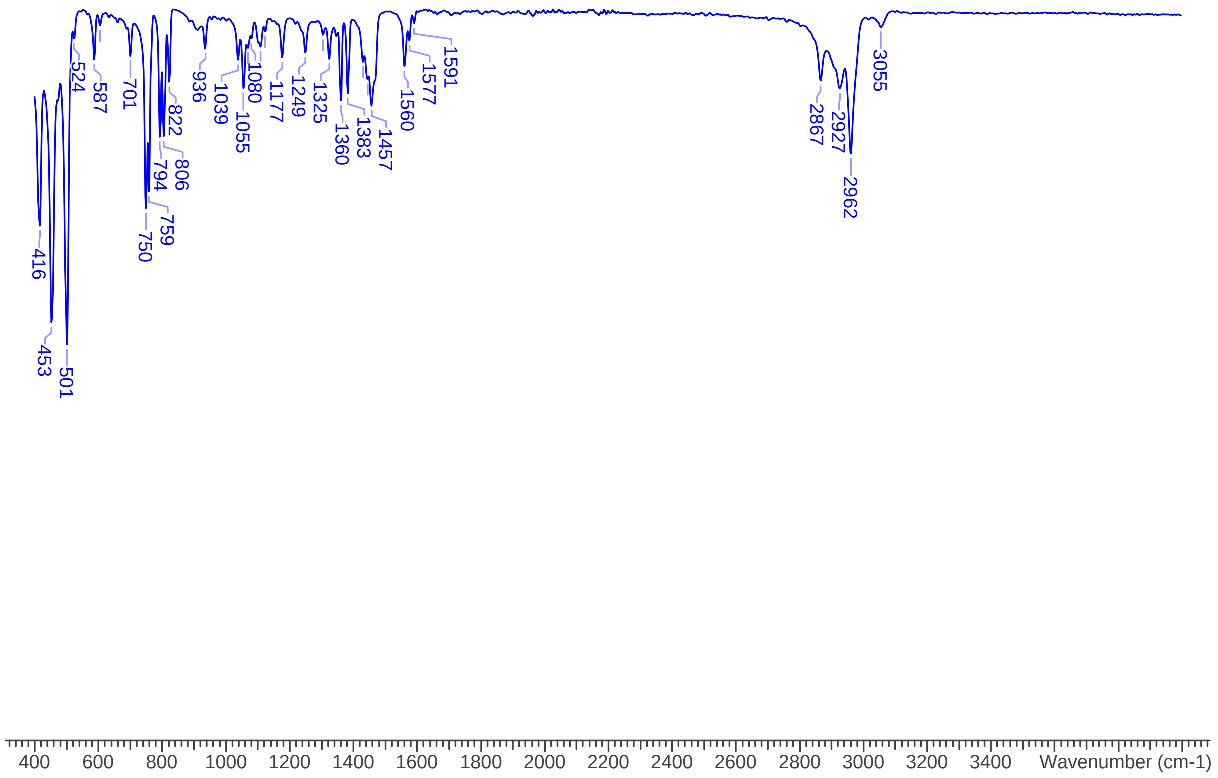

3.6 ^{Dipp}TerAsCl$_2$

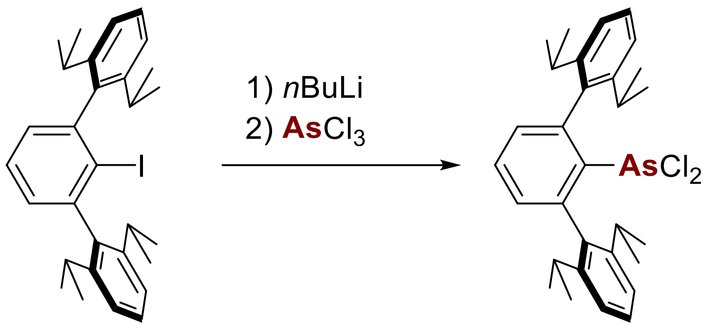

^{Dipp}TerAsCl$_2$ was synthesized according to a slightly modified literature procedure.[37]

To a solution of ^{Dipp}TerI (15.0 g, 28.6 mmol) in *n*-hexane (100 mL), *n*BuLi (12.6 mL, 2.5 M in *n*-hexane, 31.5 mmol) was added over a period of 5 min at −78 °C (CO_2/EtOH cooling bath). The reaction mixture was allowed to warm to ambient temperature in the cooling bath overnight. The colorless solid formed was allowed to settle, and the solvent was removed by canula filtration. The remaining solid was washed twice with *n*-hexane (20 mL each) and was subsequently dried *in vacuo* (1×10^{-3} mbar) for 15 min at 45 °C (water bath). The solid was then suspended in toluene (100 mL) and was cooled to −78 °C (CO_2/EtOH cooling bath). At this temperature freshly degassed (freeze-pump-thaw) $AsCl_3$ (neat, 6.2 g, 34.3 mmol) was added over a period of 10 min by syringe. The reaction mixture was stirred for 1 h at −78 °C. Afterwards the cooling bath was removed, and the reaction mixture was stirred overnight at ambient temperature. The solvent was removed under reduced pressure and the orange residue was dried *in vacuo* (1×10^{-3} mbar) for 1 h at 50 °C (water bath). The residue was suspended in *n*-hexane (100 mL) and the suspension was then placed in an ultrasound bath. The suspension was filtered hot (heat gun set to 80 °C until *n*-hexane started to reflux) over celite and the residue was extracted four times with fresh *n*-hexane (10 mL each, at 80 °C).

The filtrate was stored overnight at ambient temperature whereby an orange by-product precipitated. The supernatant solution was decantated and concentrated (to approx. 40 mL) under reduced pressure in the warmth. The solution was allowed to

cool down to ambient temperature overnight whereby large colourless crystals of $^{Dipp}TerAsCl_2$ where obtained. The supernatant was transferred to another flask and the crystals were washed twice with *n*-hexane (5 mL each). Supernatant and washing solution were combined to get a further fraction of product analogue to the procedure described above. The isolated crystalline fractions were dried *in vacuo* (1×10^{-3} mbar) for 1 h at 50 °C (water bath)[iii] Yield: 9.82 g (18.1 mmol, 63%).

CHN calc. (found) in %: C 66.30 (66.24), H 6.86 (6.42); it was necessary to add V_2O_5 to achieve complete combustion. **^{1}H NMR** (400.1 MHz, C_6D_6, 298 K): δ =1.01 (d, $^3J_{(H-H)}$ = 6.8 Hz, 12 H, C(H)(C*H*$_3$)(CH$_3$)), 1.31 (d, $^3J_{(H-H)}$ = 6.8 Hz, 12 H, C(H)(CH$_3$)(C$_3$)), 2.83 (*ψ*-hept, $^3J_{(H-H)}$ = 6.8 Hz, 4 H, C(*H*)(CH$_3$)(CH$_3$)), 7.05 – 7.09 (m, 1 H, *p*-C*H* (^{Dipp}Ter)), 7.10 – 7.13 (m, 2 H, *m*-C*H* (^{Dipp}Ter)), 7.13 – 7.16 (m, 4 H, *m*-C*H* (Dipp)), 7.27 – 7.32 (m, 2 H, *p*-C*H* (Dipp)) ppm. **^{13}C{^{1}H} NMR** (100.6 MHz, C_6D_6, 298 K): δ = 22.9 (s, C(H)(*C*H$_3$)(CH$_3$)), 25.8 (s, C(H)(CH$_3$)(*C*H$_3$)), 31.5 (s, *C*(H)(CH$_3$)(CH$_3$)), 123.2 (s, *m*-CH (Dipp)), 129.9 (s, *p*-CH (Dipp)), 130.3 (s, *p*-CH (^{Dipp}Ter)), 132.1 (s, *m*-CH (^{Dipp}Ter)), 135.6 (s, *C*-Dipp), 142.3 (s, *C*-AsCl$_2$), 145.1 (s, *i*-*C* (Dipp), 147.8 (s, *o*-*C* (Dipp)) ppm. **IR** (ATR, 32 scans, cm^{-1}): $\tilde{\nu}$ = 3057 (vw), 2960 (s), 2925 (m), 2867 (m), 1591 (vw), 1575 (w), 1562 (w), 1457 (m), 1445 (m), 1383 (m), 1362 (m), 1346 (w), 1325 (w), 1305 (w), 1251 (w), 1177 (w), 1101 (w), 1080 (w), 1055 (m), 1041 (w), 934 (w), 886 (vw), 820 (m), 806 (s), 794 (s), 759 (vs), 746 (s), 693 (w), 686 (w), 585 (w), 456 (w), 412 (w). **MS** (CI, pos., *iso*-butene, m/z): 546-543 $[M+H]^+$, 511-508 $[M+H-Cl]^+$, 399-398 $[M+H-AsCl_2]^+$.

[iii] Sometimes further crystals of the orange side product were formed between the colorless crystals of the main product. These can be removed by crystal picking in the glovebox to get analytical pure compound. This step can be omitted for the subsequent synthesis of $^{Dipp}TerAsPMe_3$.

Figure S30: ^{1}H NMR spectrum of $^{Dipp}TerAsCl_2$ (400.1 MHz, C_6D_6, 298 K).

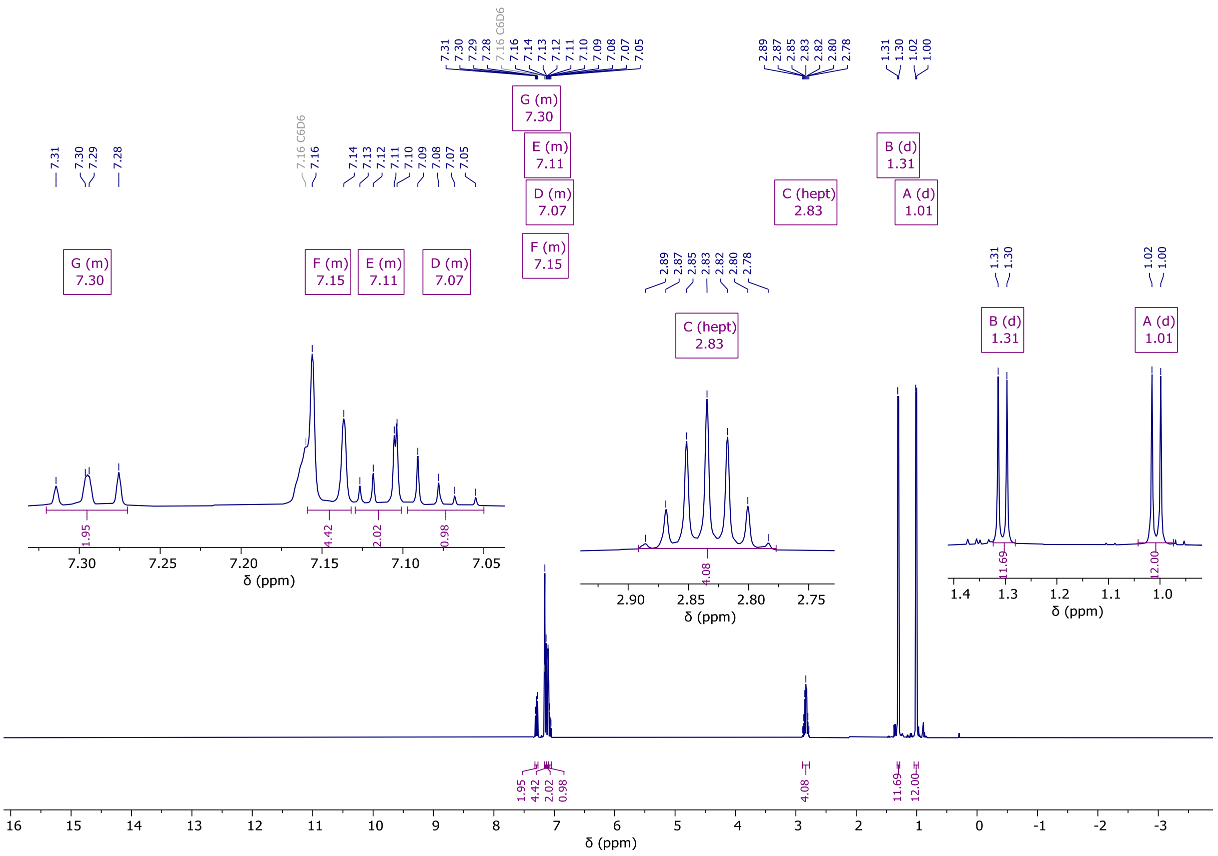

Figure S31: $^{13}C\{^{1}H\}$ NMR spectrum of $^{Dipp}TerAsCl_2$ (100.6 MHz, C_6D_6, 298 K).

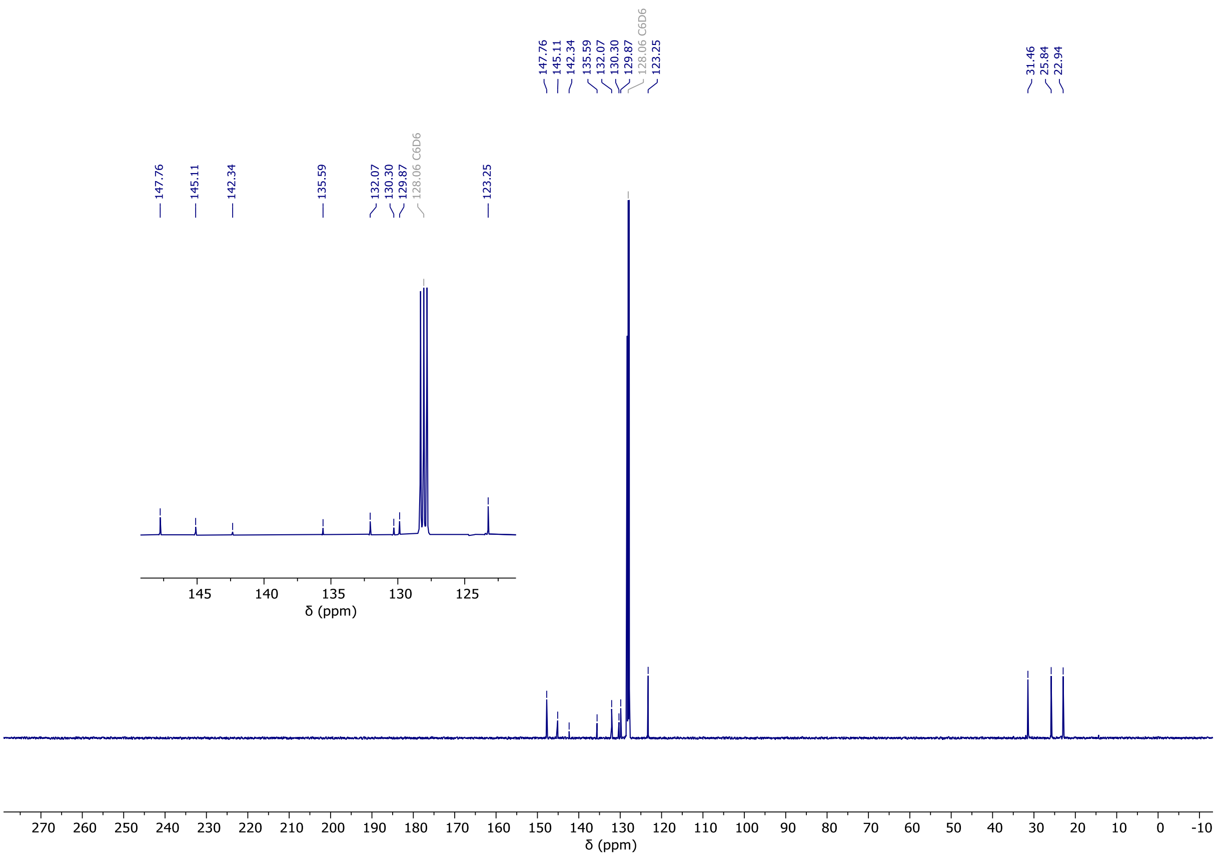

Figure S32: IR spectrum of ^{Dipp}TerAsCl_2 (ATR, 32 scans, cm^{-1}, powder).

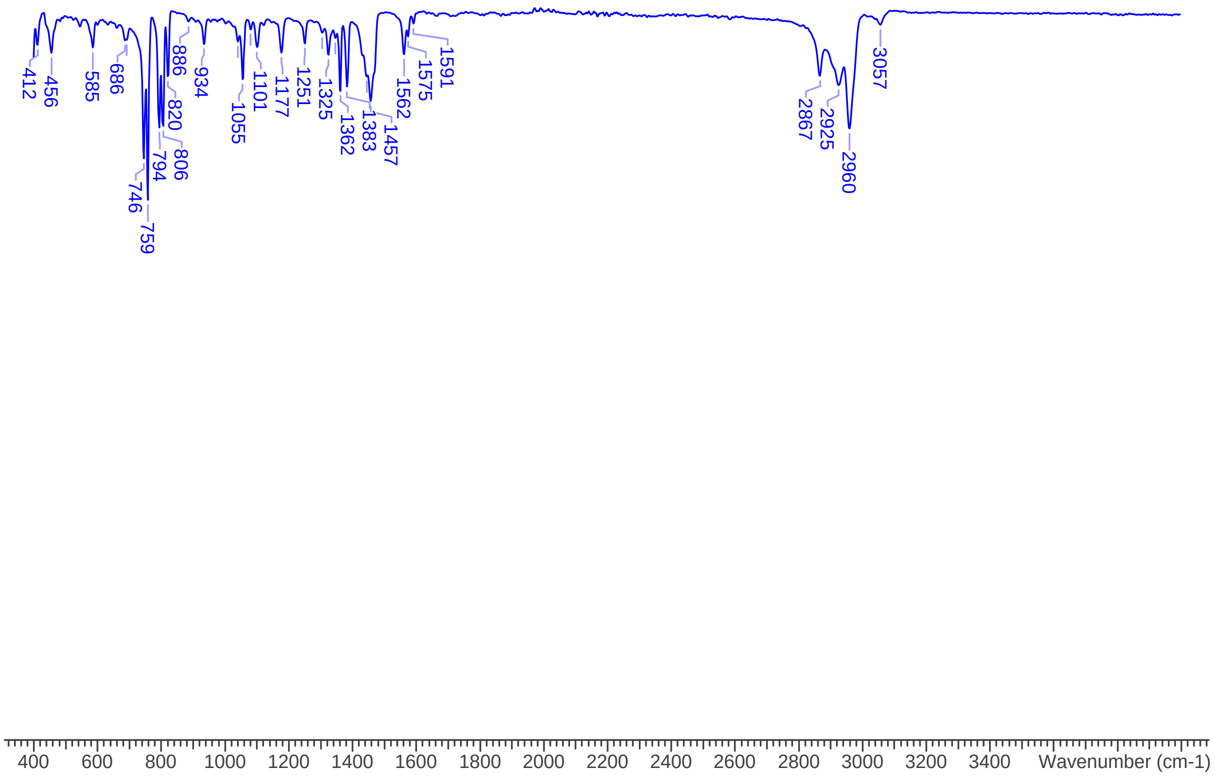

3.7 $^{Dipp}TerPPMe_3$

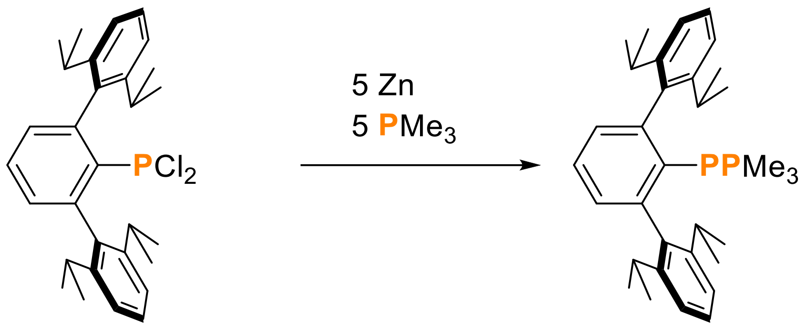

$^{Dipp}TerPPMe_3$ was synthesized according to a slightly modified literature procedure.[94]

$^{Dipp}TerPCl_2$ (2.6 g, 5.1 mmol) and zinc powder (1.7 g, 25.5 mmol) were suspended in THF (30 mL) at ambient temperature. To this suspension, a solution of PMe_3 (3.3 ml, 2.4 g, 32.0 mmol) in THF (10 mL) was added at ambient temperature. The reaction mixture was stirred for 24 h in the dark at 30 °C (oil bath). The supernatant was collected by cannula filtration, and the residue was re-extracted with THF (30 mL). The filtrates were combined, and all volatile components were removed *in vacuo*. The residual yellow solid was suspended and washed twice with cold (−30 °C) *n*-hexane (10 mL each). The residual yellow compound was dried *in vacuo*. Yield: 1.96 g (3.89 mmol, 76%).

CHN calc. (found) in %: C 78.54 (76.68), H 9.19 (8.91); deviations probably due to incomplete combustion, repeated measurements with and without adding V_2O_5 as additional oxidation reagent did not result in better agreement. **^{1}H NMR** (400.1 MHz, C_6D_6, 298 K): δ = 0.62 (dd, $^2J_{(H\text{-}P)}$ = 11.9 Hz, $^3J_{(H\text{-}P)}$ = 3.3 Hz, 9 H, P(C*H*$_3$)$_3$), 1.17 (d, $^3J_{(H\text{-}H)}$ = 6.8 Hz, 12 H, C(H)(C*H*$_3$)(CH$_3$)), 1.44 (d, $^3J_{(H\text{-}H)}$ = 6.8 Hz, 12 H, C(H)(CH$_3$)(C*H*$_3$)), 3.15 (ψ-hept, $^3J_{(H\text{-}H)}$ = 6.8 Hz, 4 H, *C*(H)(CH$_3$)(CH$_3$)), 7.00 – 7.10 (m, 1 H, *p-CH* (^{Dipp}Ter)), 7.10 – 7.15 (m, 2 H, *m-CH* (^{Dipp}Ter)), 7.22 – 7.26 (m, 4 H, *m-CH* (Dipp)), 7.29 – 7.34 (m, 2 H, *p-CH* (Dipp)) ppm. **^{13}C{^{1}H} NMR** (100.6 MHz, C_6D_6, 298 K): δ = 17.3 (dd, $^1J_{(C\text{-}P)}$ = 42 Hz, $^2J_{(C\text{-}P)}$ = 15 Hz, P(CH$_3$)$_3$), 24.2 (d, $J_{(C\text{-}P)}$ = 3 Hz, C(H)(CH$_3$)(CH$_3$)), 26.0 (s, C(H)(CH$_3$)(CH$_3$)), 31.1 (d, $J_{(C\text{-}P)}$ = 2 Hz, *C*(H)Me$_2$), 123.6 (s, *m*-CH (Dipp)), 128.1(s, *p*-CH (Dipp)), 128.4 (s, *p*-CH (^{Dipp}Ter)), 130.2 (s, *m*-CH (^{Dipp}Ter)), 142.9 (d, $J_{(C\text{-}P)}$ = 4 Hz, *i-C* (Dipp)), 144.5 (dd,

$^{1}J_{(C\text{-}P)}$ = 65 Hz, $^{2}J_{(C\text{-}P)}$ = 10 Hz, *C*-PPMe3), 146.3 (dd, $J_{(C\text{-}P)}$ = 14 Hz, $J_{(C\text{-}P)}$ = 11 Hz, *C*-Dipp), 147.3 (s, *o*-*C* (Dipp)) ppm. **^{31}P{^{1}H} NMR** (162.0 MHz, C_6D_6, 298 K): δ = −116.45 (d, $^{1}J_{(P\text{-}P)}$ = 561.1 Hz, 1 P, *P*PMe3), −3.08 (d$^{1}J_{(P\text{-}P)}$ = 561.1 Hz, 1 P, P*P*Me3) ppm. **IR** (ATR, 32 scans, cm^{-1}): $\tilde{\nu}$ = 3053 (w), 3038 (w), 2958 (m), 2927 (m), 2865 (m), 1573 (w), 1457 (m), 1445 (m), 1424 (w), 1410 (m), 1369 (m), 1358 (m), 1282 (m), 1249 (w), 1226 (w), 1177 (w), 1119 (w), 1055 (w), 1031 (w), 1000 (w), 948 (vs), 843 (w), 806 (m), 787 (m), 756 (s), 748 (s), 728 (m), 695 (w), 678 (s), 655 (w), 585 (w), 550 (w), 505 (w), 493 (m), 472 (w), 464 (w), 427 (w). **MS** (CI, pos., isobutene, m/z): 506 [M+2 H]$^{+}$, 462 [M−C_3H_7+H]$^{+}$. **HR-MS** (ESI pos., MeCN + HC(O)OH, m/z) Calc: 505.3143 [M+H]$^{+}$; Found: 505.3158.

Figure S33: ^{1}H NMR spectrum of ^{Dipp}TerPPMe$_3$(400.1 MHz, C_6D_6, 298 K).

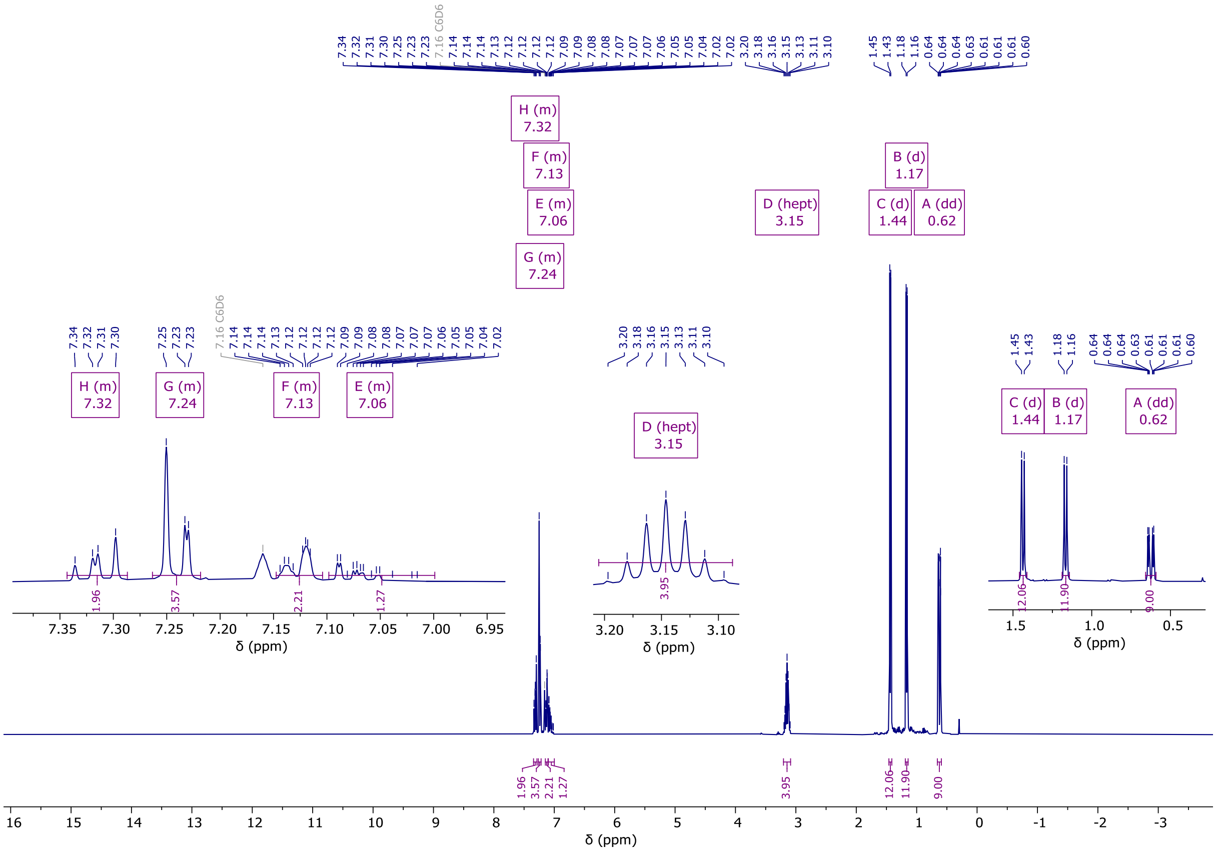

Figure S34: $^{31}P\{^{1}H\}$ NMR spectrum of $^{Dipp}TerPPMe_3$ (162.0 MHz, C_6D_6, 298 K).

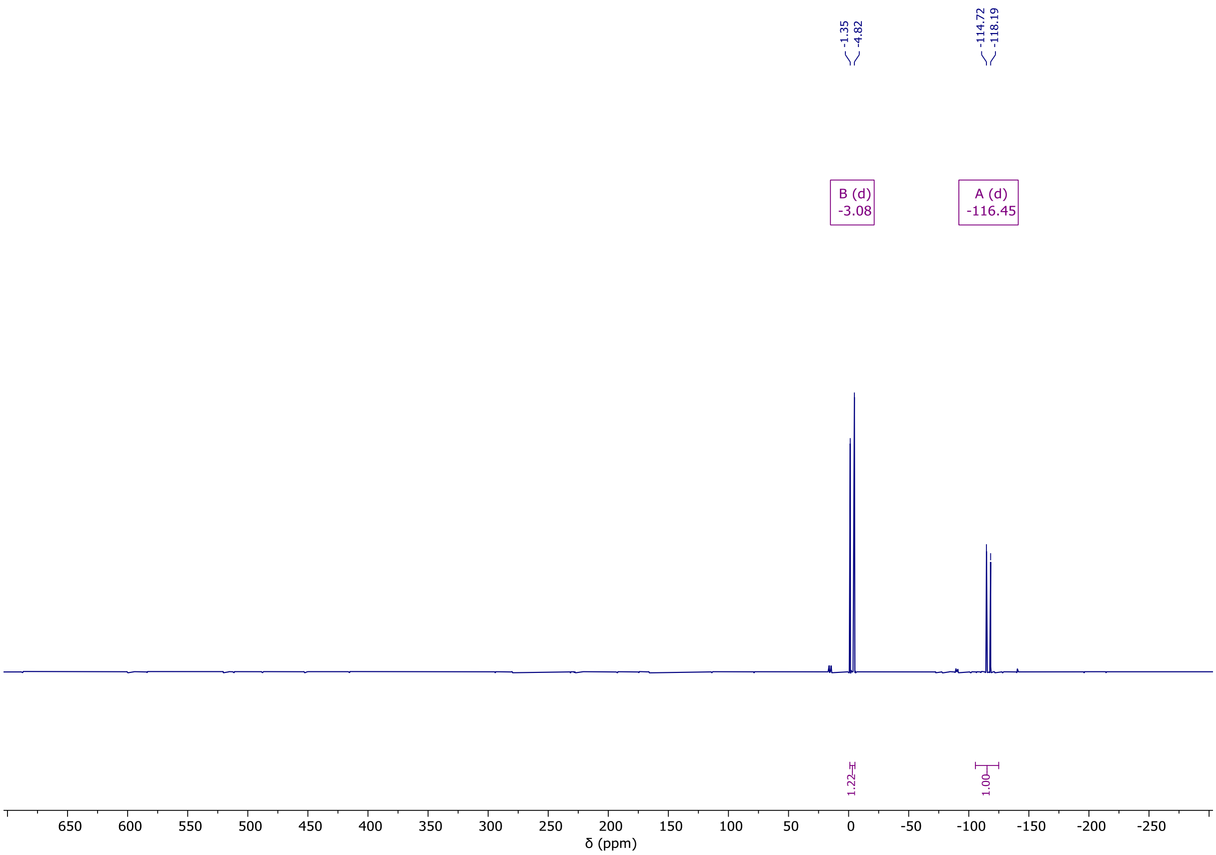

Figure S35: $^{13}C\{^1H\}$ NMR spectrum of ^{Dipp}TerPPMe$_3$ and comparison of the aromatic region in the $^{13}C\{^1H\}$ and ^{13}C-dept-135 NMR spectrum, showing an overlap of signals from ^{Dipp}TerPPMe$_3$ and C_6D_6 (100.6 MHz, C_6D_6, 298 K).

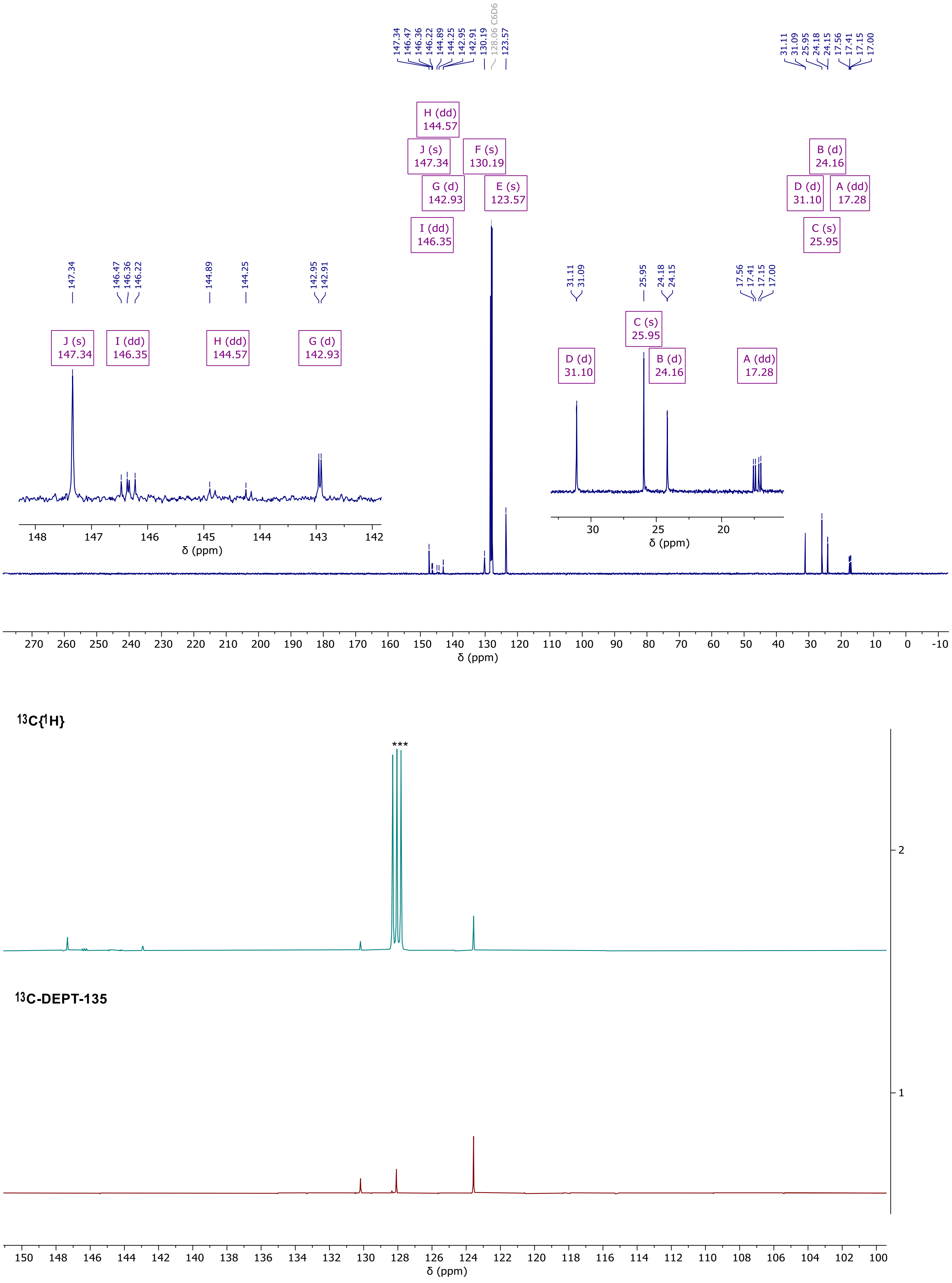

Figure S36: IR spectrum of ^{Dipp}TerPPMe$_3$ (ATR, 32 scans, cm^{-1}, powder).

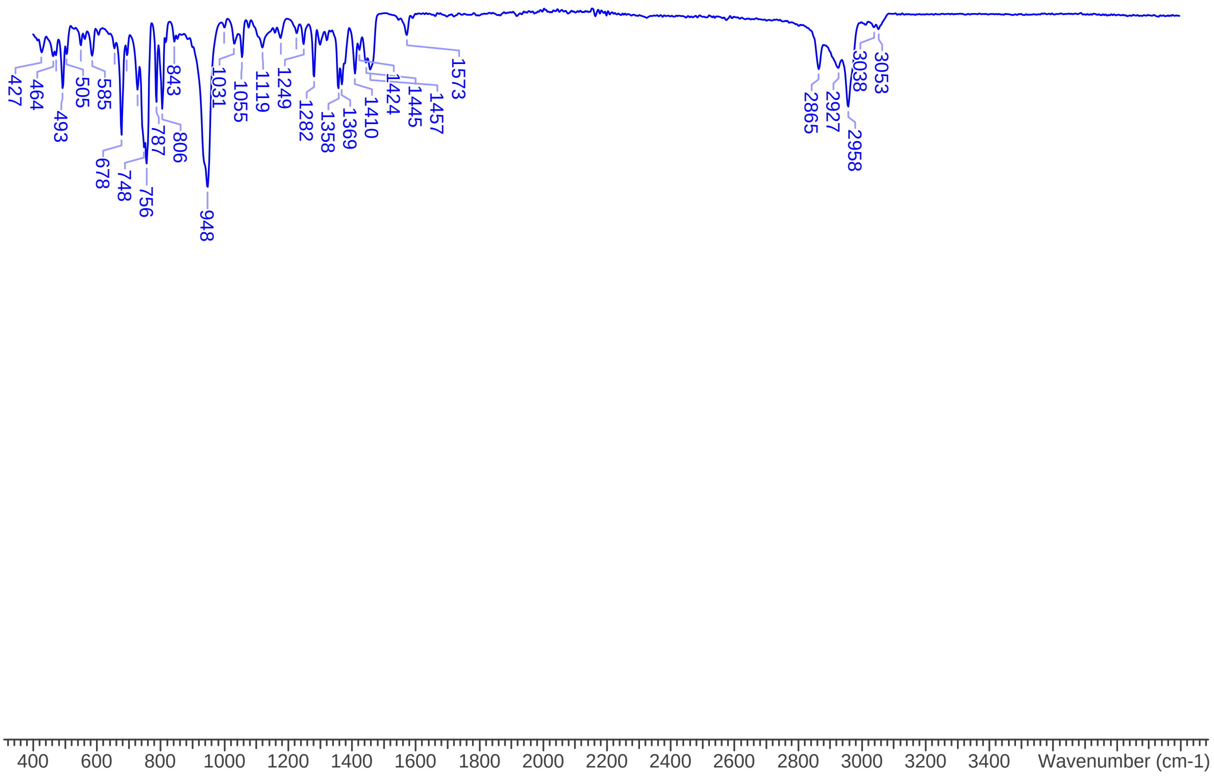

3.8 ^{Dipp}TerAsPMe$_3$

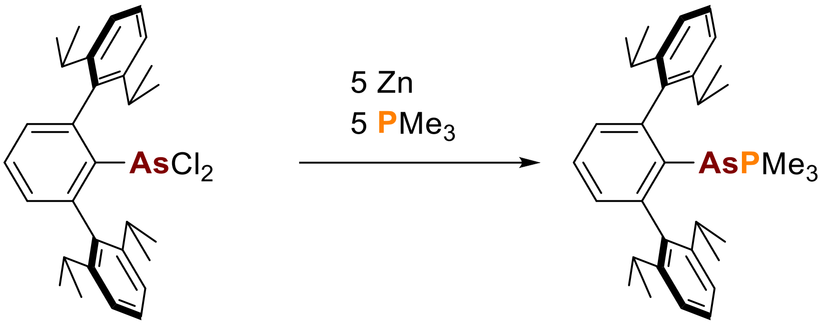

^{Dipp}TerAsPMe$_3$ was synthesized according to a slightly modified literature procedure.[37]

^{Dipp}TerAsCl$_2$ (5.0 g, 9.2 mmol) and zinc dust (3.01 g, 46.0 mmol) were suspended in THF (100 mL). To this suspension was added a solution of PMe$_3$ (4.7 mL, 46.0 mmol) in THF (5 mL). The suspension was stirred for 2.5 h at ambient temperature under the exclusion of light. The suspension was filtered using celite-padded frit and the residue was extracted twice with fresh THF (50 mL each). Next the volatile components of the filtrate were removed *in vacuo* and the remaining solid was dried for 15 min at 45 °C (water bath) at 1×10^{-3} mbar. The solid was suspended in *n*-pentane (100 mL) and was placed in an ultrasound bath. The suspension was then cooled to −78 °C (CO_2/EtOH cooling bath) and the yellowish solid was allowed to settle. The supernatant was removed by cannula filtration. The solid was washed with cold (−78 °C) *n*-pentane (20 mL). The remining deeply yellow colored solid was dried for 2.5 h at ambient temperature at 1×10^{-3} mbar under the exclusion of light. ^{Dipp}TerAsPMe$_3$ was used without further purification (see impurities in the ^{1}H NMR). Yield: 3.679 g (6.71 mmol, 73%)

CHN calc. (found) in %: C 72.25 (70.17), H 8.45 (7.12); deviations probably due to impurities (see ^{1}H NMR) or incomplete combustion, repeated measurements with and without adding V_2O_5 as additional oxidation reagent did not result in better agreement. **NMR** (300.2 MHz, C_6D_6, 298 K): δ = 0.70 (d, $^{2}J_{(H-P)}$ = 11.9 Hz, 9 H, P(CH_3)$_3$), 1.17 (d, $^{3}J_{(H-H)}$ = 6.8 Hz, 12 H, C(H)(CH_3)(CH$_3$)), 1.45 (d, $^{3}J_{(H-H)}$ = 6.9 Hz, 12 H, C(H)(CH$_3$)(CH_3)), 3.18 (ψ-hept, $^{3}J_{(H-H)}$ = 6.8 Hz, 4 H C(H)(CH$_3$)(CH$_3$)), 7.09 – 7.12 (superimposed signals, 3 H,

m- and *p*-C*H* (^{Dipp}Ter)), 7.22-7.26 (m, 4 H, *m*-C*H* (Dipp)), 7.29 – 7.35 (m, 2 H, *p*-C*H* (Dipp)) ppm. **^{13}C{^{1}H} NMR** (75.5 MHz, C_6D_6, 298 K): δ = 17.5 (d, $^{1}J_{(C\text{-}P)}$ = 35 Hz, $P(CH_3)_3$), 24.2 (s, $C(H)(CH_3)(CH_3)$), 25.9 (s, $C(H)(CH_3)(CH_3)$), 31.1 (s, *C*(H)(CH$_3$)(CH$_3$)), 123.6 (s, *m*-*C*H (Dipp)), 123.7 (s, *p*-CH (^{Dipp}Ter)), 128.0 (s, *p*-CH (Dipp)), 130.0 (s, m-CH (^{Dipp}Ter)), 134.2 (s, *i*-*C* (Dipp)), 146.1 (*C*$_{quart.}$ (^{Dipp}Ter)), 146.2 (*C*$_{quart.}$ (^{Dipp}Ter)), 147.4 (s, *o*-*C* (Dipp)) ppm. **^{31}P{^{1}H} NMR** (162.0 MHz, C_6D_6, 298 K): δ = −14.59 (s, *P*Me$_3$) ppm. **^{13}C{^{1}H} NMR** (75.5 MHz, C_6D_6, 298 K): δ = 17.5 (d, $^{1}J_{(C\text{-}P)}$ = 35 Hz, $P(CH_3)_3$), 24.2 (s, $C(H)(CH_3)(CH_3)$), 25.9 (s, $C(H)(CH_3)(CH_3)$), 31.1 (s, *C*(H)Me$_2$), 123.6 (s, *m*-CH (Dipp)), 123.7 (s, *p*-CH (^{Dipp}Ter)), 128.0 (s, *p*-CH (Dipp)), 130.0 (s, m-CH (^{Dipp}Ter)), 134.2 (s, *i*-*C* (Dipp)), 146.1 (*C*$_{quart.}$ (^{Dipp}Ter)), 146.2 (*C*$_{quart.}$ (^{Dipp}Ter)), 147.4 (s, *C*-*i*Pr) ppm. **IR** (ATR, 32 scans, cm^{-1}): $\tilde{\nu}$ = 3055 (w), 2958 (m), 2925 (m), 2910 (w), 2865 (m), 1575 (w), 1562 (vw), 1457 (m), 1443 (w), 1424 (w), 1412 (w), 1381 (m), 1360 (m), 1323 (w), 1301 (w), 1278 (w), 1249 (w), 1231 (vw), 1177 (w), 1158 (vw), 1078 (vw), 1055 (w), 1020 (vw), 1002 (vw), 946 (s), 816 (w), 804 (m), 785 (m), 759 (vs), 742 (s), 688 (vw), 670 (m), 585 (w), 460 (m). **MS** (CI, pos., *iso*-butene, m/z): 474 [M – PMe$_3$+ 2 H]$^+$.

Figure S37: ^{1}H NMR spectrum of $^{Dipp}TerAsPMe_3$ (300.2 MHz, C_6D_6, 298 K).

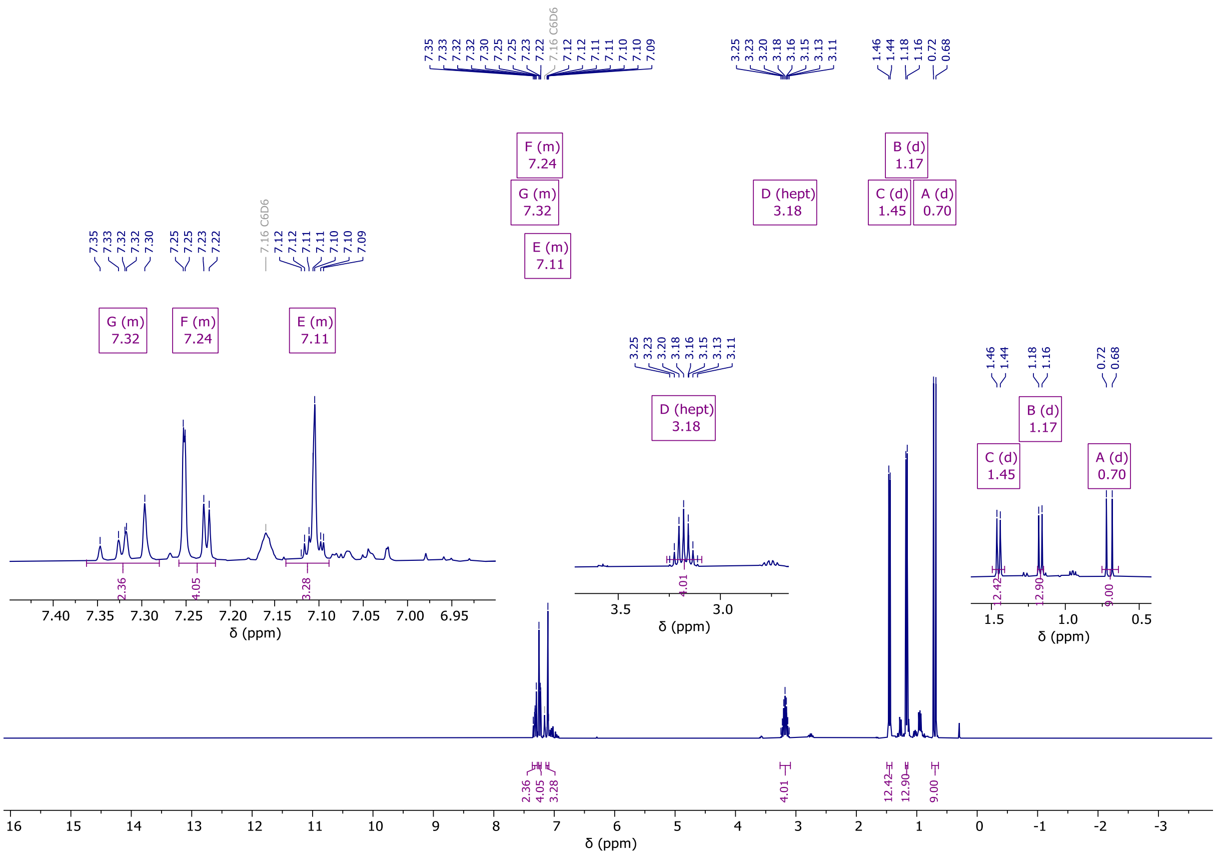

Figure S38: $^{31}P\{^{1}H\}$ NMR spectrum of $^{Dipp}TerAsPMe_3$ (162.0 MHz, C_6D_6, 298 K).

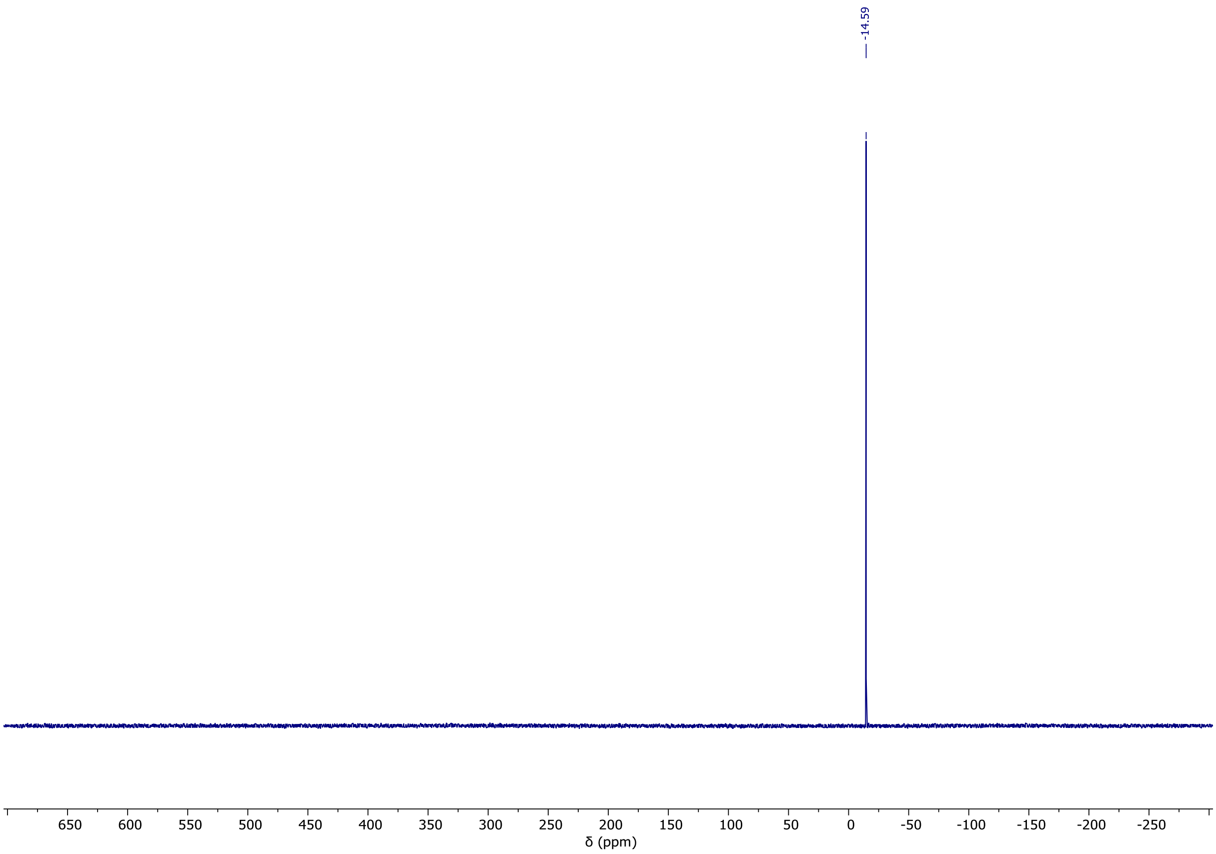

Figure S39: $^{13}C\{^{1}H\}$ NMR spectrum of $^{Dipp}TerAsPMe_3$ and comparison of the aromatic region in the $^{13}C\{^{1}H\}$ and ^{13}C-dept-135 NMR spectrum, showing an overlap of signals from $^{Dipp}TerAsPMe_3$ and C_6D_6 (75.5 MHz, C_6D_6, 298 K).

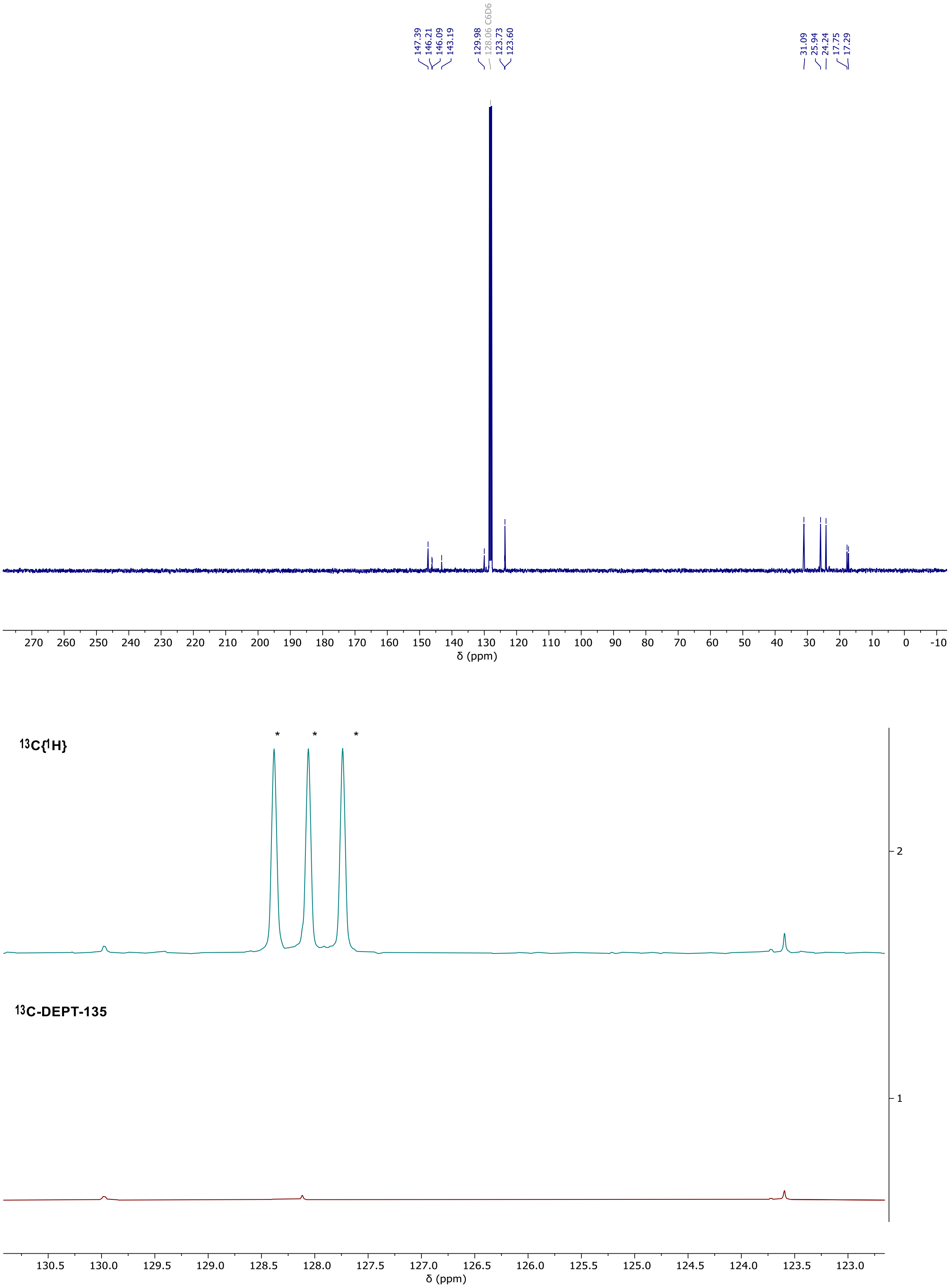

Figure S40: IR spectrum of ^{Dipp}TerAsPMe$_3$ (ATR, 32 scans, cm^{-1}, powder).

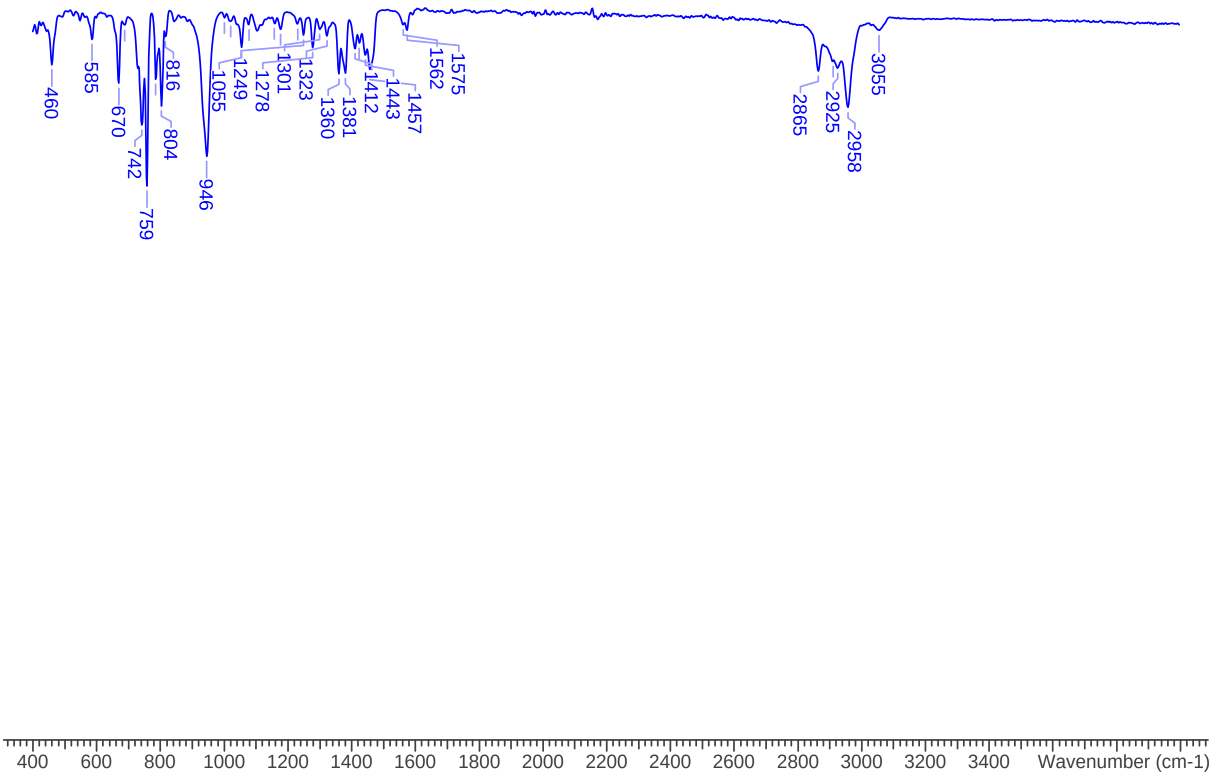

3.9 ^{Dipp}TerPAlCp*

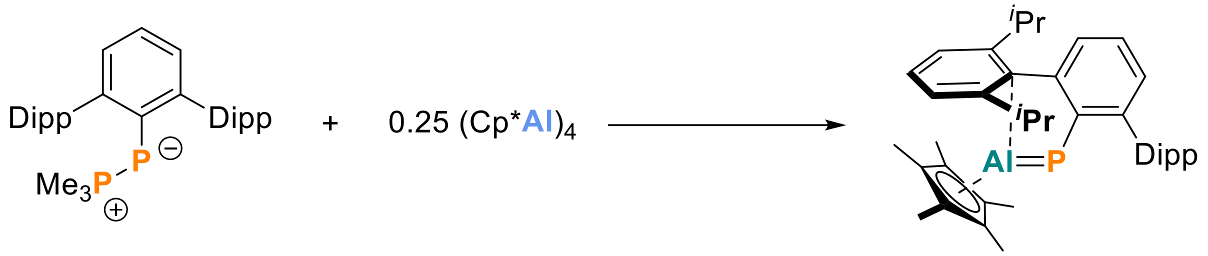

^{Dipp}TerPAlCp was synthesized according to a slightly modified literature procedure.*[39]

^{Dipp}TerPPMe$_3$ (1.04 g, 2.05 mmol) and {Cp*Al}$_4$ (0.39 g, 0.60 mmol) were suspended in toluene (40 mL). The suspension was degassed (3 x freeze-pump-thaw) and heated under stirring for 17 h to 80 °C (oil bath). Within this timeframe the color of the reaction mixture changed from yellow to violet. Next, all volatile components were removed *in vacuo* and the residual solid was dried for 30 min at 50 °C (water bath) at 1×10^{-3} mbar. *n*-Pentane (60 mL) was added, and the resulting suspension was extracted by repeated filtration and recondensation of the solvent (two times). The filtrate was concentrated to incipient crystallization (approx. 30 mL) and was slowly cooled down by leaving it in a bowl of warm water (initially 45 °C) in the fridge (+5 °C) overnight, resulting in the deposition of large violet to blue crystals. The supernatant was removed by syringe and the crystals were washed twice with cold (−78 °C) *n*-pentane (10 mL each). The washing solution was discarded, and the mother liquor was concentrated further to get another crops of crystals, in the same way as described above. The isolated crystals were dried *in vacuo* (1×10^{-3} mbar) for 30 min at ambient temperature. Yield: 0.98 g (1.66 mmol, 81%).

CHN calc. (found) in %: C 81.32 (80.98), H 8.87 (8.26); it was necessary to add V_2O_5 to achieve complete combustion. **^{1}H NMR** (300.2 MHz, C_6D_6, 298 K): δ = 1.15 (d, $^3J_{(H-H)}$ = 6.9 Hz, 12 H, C(H)(C*H*$_3$)(CH$_3$)), 1.44 – 1.54 (superimposed signals, 27 H, C(H)(CH$_3$)(C*H*$_3$) and C$_5$(C*H*$_3$)$_5$), 3.04 (ψ-hept, $^3J_{(H-H)}$ = 6.9 Hz, 4 H, C(*H*)(CH$_3$)(CH$_3$)), 6.84 – 6.89 (m, 2 H, *m*-C*H* (^{Dipp}Ter)), 6.90 – 6.97 (m, 2 H, *m*-C*H* (^{Dipp}Ter)), 7.18 – 7.22 (m, 4 H,

m-C*H* (Dipp)), 7.23 – 7.30 (m, 2 H, *p*-C*H* (Dipp)) ppm. **^{13}C{^{1}H} NMR** (75.5 MHz, C_6D_6, 298 K): δ = 10.2 (d, $J_{(C-P)}$ = 4 Hz, $C_5(CH_3)_5$)25.0 (d, $J_{(C-P)}$ = 2 Hz, C(H)(CH_3)(CH_3)), 25.0 (s, C(H)(CH_3)(CH_3)), 30.9 (s, *C*(H)(CH_3)(CH_3)), 116.3 (s, $C_5(CH_3)_5$)119.9 (s, *p*-CH (^{Dipp}Ter)), 124.3 (s, *m*-CH (Dipp)), 127.4 (d, $J_{(C-P)}$ = 3 Hz, *m*-CH (^{Dipp}Ter)), 128.3 (s, *p*-CH (Dipp)), 142.7 (d, $J_{(C-P)}$ = 16 Hz, *C*-Dipp), 144.6 (d, $J_{(C-P)}$ = 2 Hz, *i*-C Dipp), 147.7 (s, o-C Dipp), 151.6 (d, $^{1}J_{(C-P)}$ = 77 Hz, *i*-C ^{Dipp}Ter) ppm. **^{31}P{^{1}H} NMR** (162 MHz, C_6D_6, 298 K): δ = −203.9 (s) ppm. **IR** (ATR, 32 scans, cm^{-1}): $\tilde{\nu}$ = 3051 (vw), 3015 (vw), 2956 (s), 2921 (m), 2863 (m), 1571 (w), 1457 (m), 1422 (w), 1379 (m), 1369 (s), 1327 (w), 1245 (w), 1175 (w), 1111 (w), 1078 (w), 1055 (m), 1039 (m), 1004 (w), 950 (w), 932 (w), 806 (s), 781 (m), 756 (vs), 732 (s), 695 (w), 583 (s), 557 (vs), 546 (vs), 472 (m). **MS** (CI, pos., isobutene, m/z): 593-591 $[M+H]^+$.

Figure S41: ^{1}H NMR spectrum of ^{Dipp}TerPAlCp* (300.2 MHz, C_6D_6, 298 K).

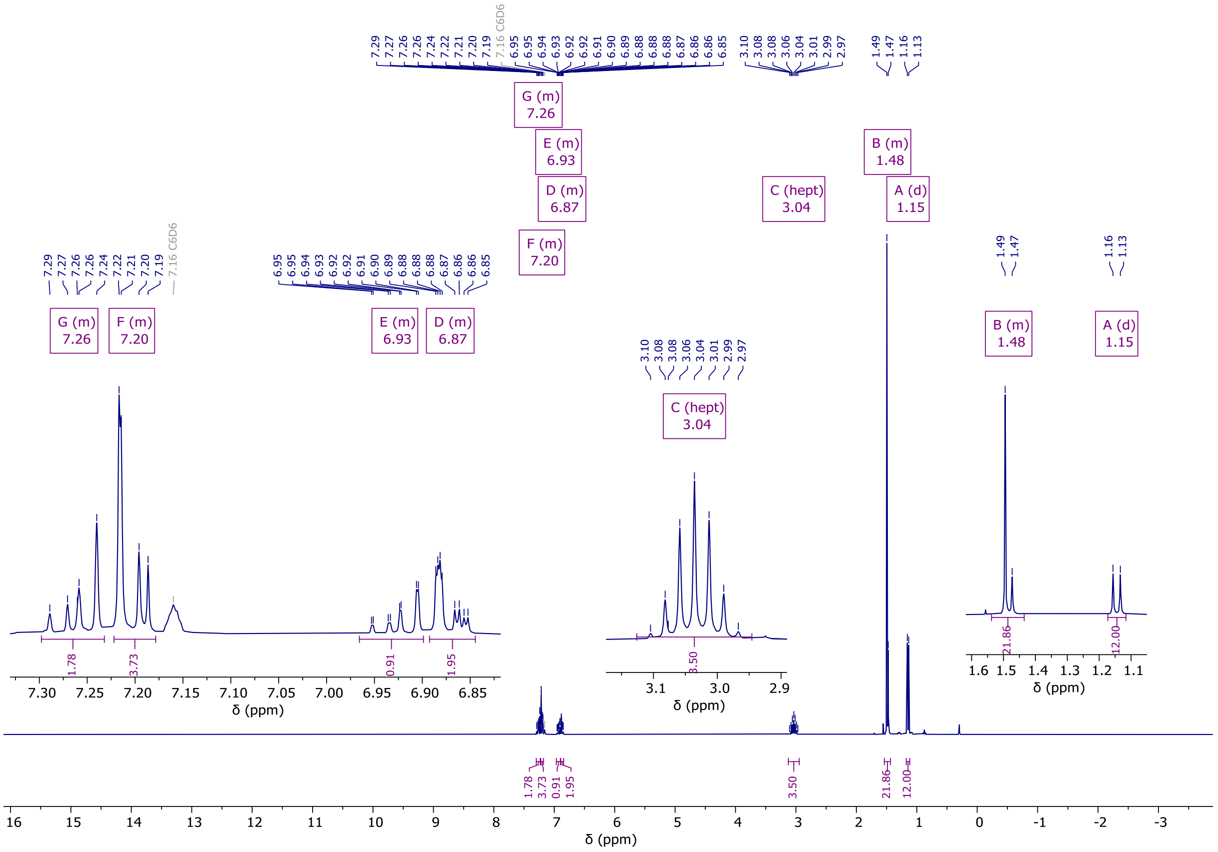

Figure S42: $^{31}P\{^1H\}$ NMR spectrum of ^{Dipp}TerPAlCp* (121.6 MHz, C_6D_6, 298 K).

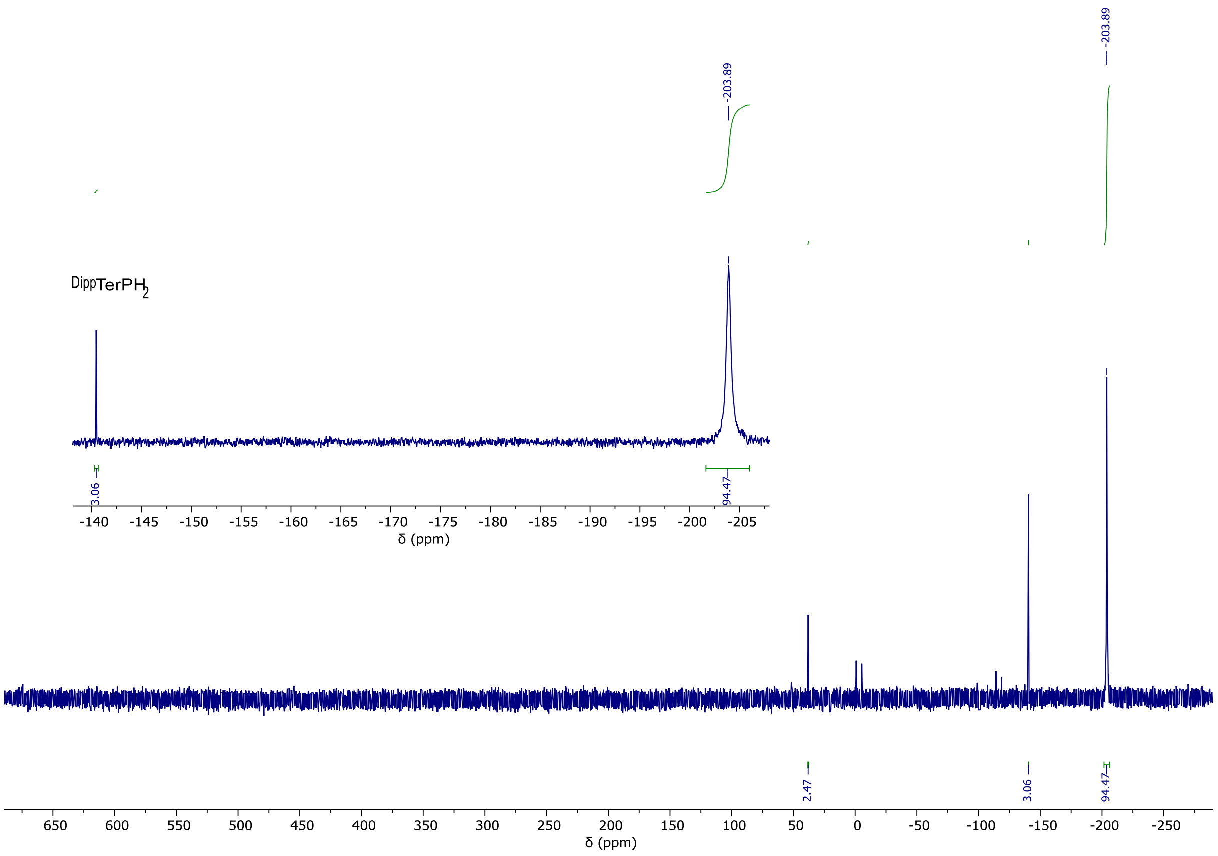

Figure S43: $^{13}C\{^1H\}$ NMR spectrum of ^{Dipp}TerPAlCp* and comparison of the aromatic region in the $^{13}C\{^1H\}$ and ^{13}C-dept-135 NMR spectrum, showing an overlap of signals from ^{Dipp}TerPAlCp* and C_6D_6 (75.5 MHz, C_6D_6, 298 K).

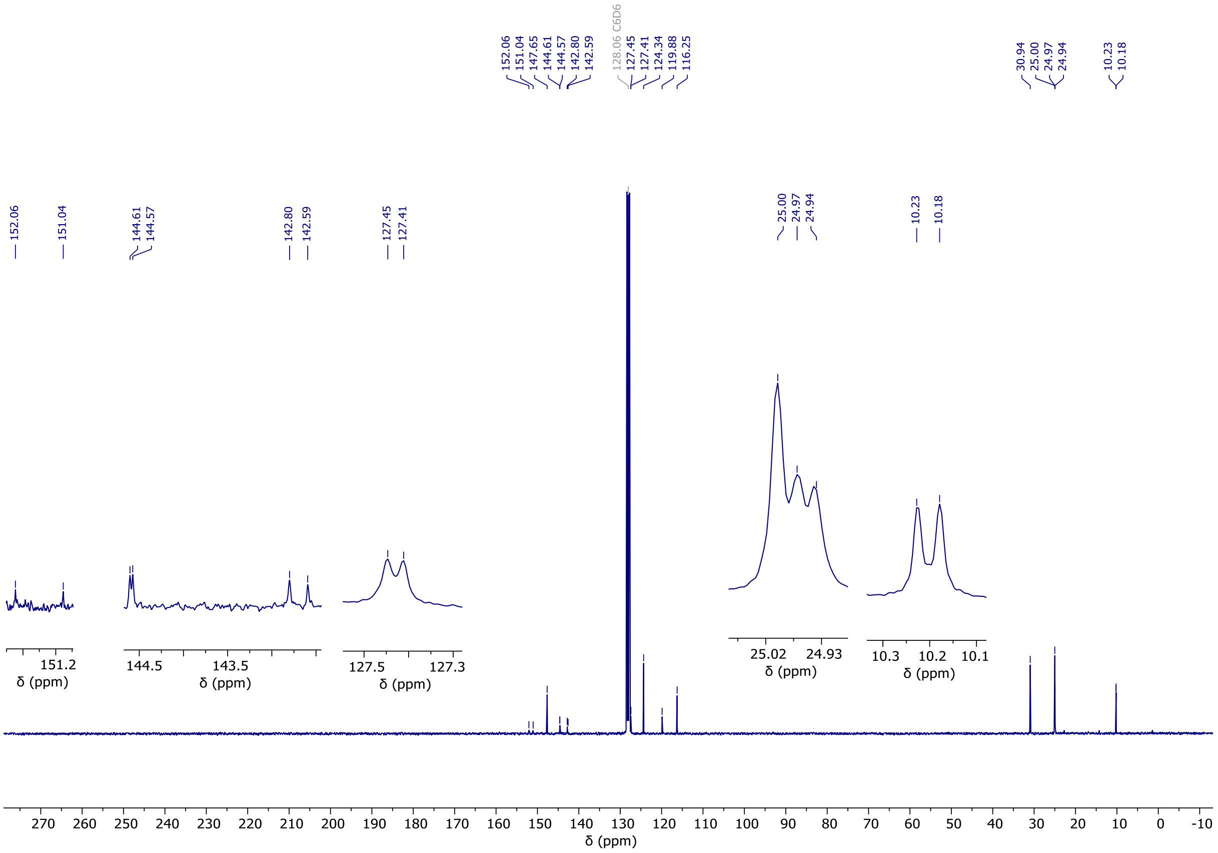

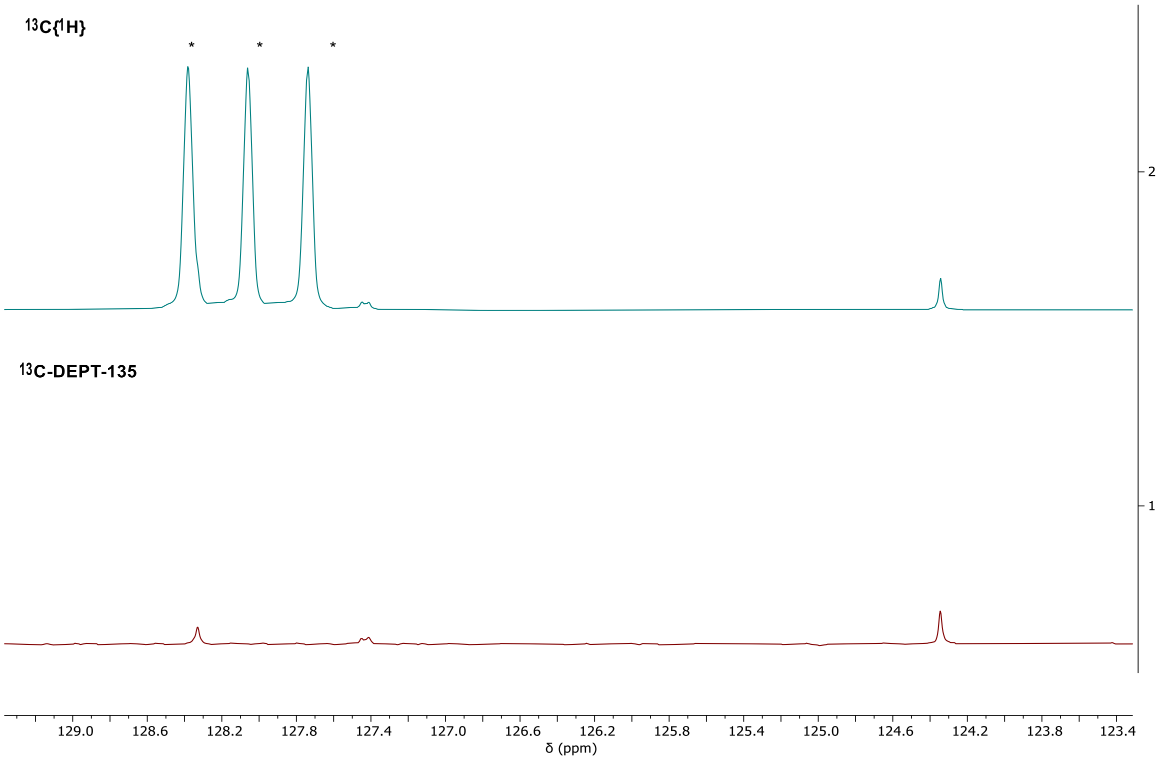

Figure S44: IR spectrum of ^{Dipp}TerPAlCp* (ATR, 32 scans, cm^{-1}, powder).

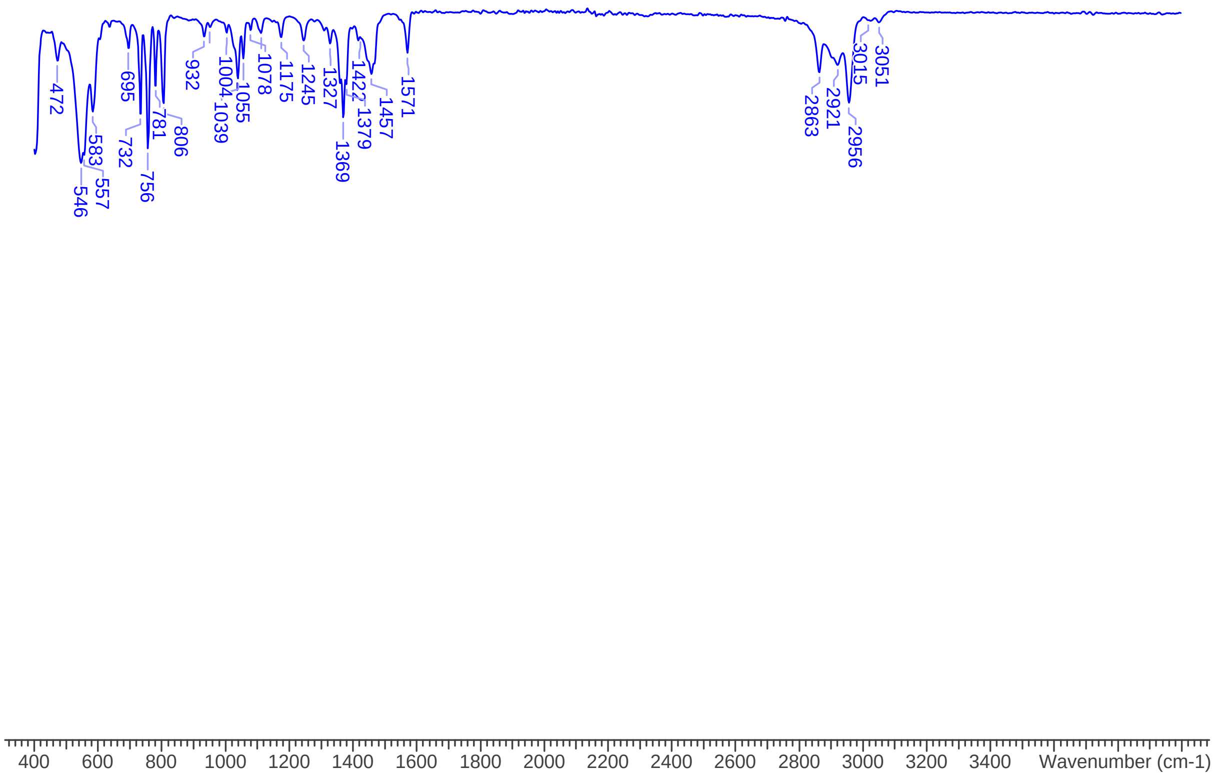

3.10 ^{Dipp}TerAsAlCp*

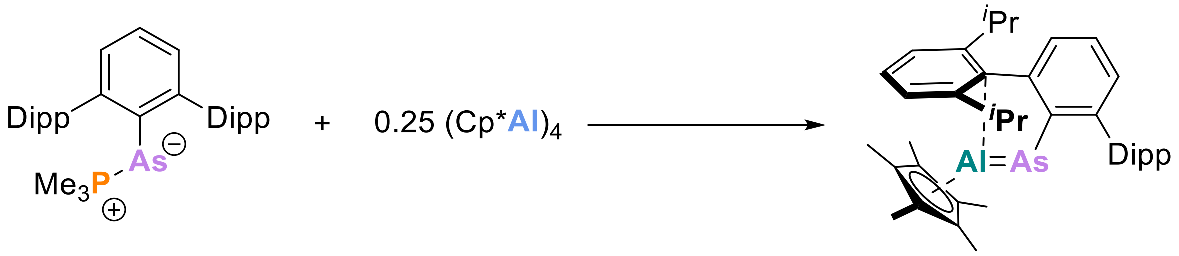

^{Dipp}TerAsAlCp was synthesized according to a slightly modified literature procedure.*[37]

^{Dipp}TerAsPMe$_3$ (1.00 g, 1.82 mmol) and {Cp*Al}$_4$ (0.30 g, 0.46 mmol) were suspended in toluene (60 mL). The suspension was degassed (3 x freeze-pump-thaw) and heated under stirring for 21 h to 80 °C (oil bath). Within this timeframe the colour of the reaction mixture changed from yellow to green. Then all volatile components were removed *in vacuo* and the residual solid was dried for 30 min at 50 °C (water bath) at 1×10^{-3} mbar. *n*-Pentane (50 mL) was added, and the resulting suspension was extracted by repeated filtration and recondensation of the solvent (two times). The filtrate was concentrated to incipient crystallization (approx. 40 mL) and was slowly cooled down by leaving it in a bowl of warm water (initially 45 °C) in the fridge (+5 °C) overnight, resulting in the deposition of large blue crystals. The supernatant was removed by syringe and the crystals were washed with cold (−78 °C) *n*-pentane (10 mL). The washing solution was discarded, and the mother liquor was concentrated to get another crop of crystals, in the same way as described above. Crystals were died *in vacuo* (1×10^{-3} mbar) for 30 min at ambient temperature. Yield: 0.41 g (0.64 mmol, 35 %).

CHN calc. (found) in %: C 75.69 (74.38), H 8.26 (7.33); deviations probably due to incomplete combustion, repeated measurements with and without adding V_2O_5 as additional oxidation reagent did not result in better agreement. **^{1}H NMR** (300.2 MHz, C_6D_6, 298 K): δ = 1.14 (d, $^3J_{(H\text{-}H)}$ = 6.9 Hz, 12 H, C(H)(CH_3)(CH$_3$)), 1.49 (s, 15 H, (C$_5$(CH_3)$_5$), 1.51 (d, $^3J_{(H\text{-}H)}$ = 6.9 Hz, 12 H, C(H)(CH$_3$)(CH_3)), 3.05 (ψ-hept, $^3J_{(H\text{-}H)}$ = 6.9 Hz, 4 H,

C(*H*)(CH$_3$)(CH$_3$)), 6.87 – 6.92 (m, 2 H, *m-CH* (^{Dipp}Ter)), 6.95 – 7.02 (m, 1 H, *p-CH* (^{Dipp}Ter)), 7.18 – 7.22 (m, 4 H, *m-CH* (Dipp)), 7.24 – 7.32 (m, 2 H, *p-CH* (Dipp)). **^{13}C{^{1}H} NMR** (75.5 MHz, C$_6$D$_6$, 298 K): δ = 10.2 (s, (C$_5$CH$_3$)$_5$), 25.1 (s, C(H)(CH$_3$)(CH$_3$)), 25.1 (s, C(H)(CH$_3$)(CH$_3$)), 31.0 (s, *C*(H)(CH$_3$)(CH$_3$)), 116.1 (s, (*C*$_5$(CH$_3$)$_5$ (Cp*)), 121.1 (s, *p*-CH (^{Dipp}Ter)), 124.2 (s, *m*-CH (Dipp)), 127.1 (s, *m*-CH (^{Dipp}Ter)), 128.3 (s, *p*-CH Dipp), 144.0 (s, C-Dipp), 145.6 (s, *i-C* Dipp), 147.5 (s, *o-C* Dipp), 152.0 (s, *i*-C^{Dipp}Ter) ppm. **IR** (ATR, 32 scans, cm^{-1}): $\tilde{\nu}$ = 3048 (vw), 3015 (vw), 2956 (m), 2923 (m), 2863 (m), 1571 (w), 1461 (m), 1445 (w), 1414 (w), 1371 (m), 1358 (m), 1327 (w), 1247 (w), 1239 (w), 1175 (w), 1160 (w), 1078 (w), 1024 (w), 1004 (w), 800 (m), 783 (m), 756 (s), 736 (m), 682 (w), 585 (m), 513 (vs), 458 (m). **MS** (CI, pos., *iso*-butene, m/z): 537-635 [M+H]$^+$.

Figure S45: ^{1}H NMR spectrum of ^{Dipp}TerAsAlCp* (300.2 MHz, C$_6$D$_6$, 298 K).

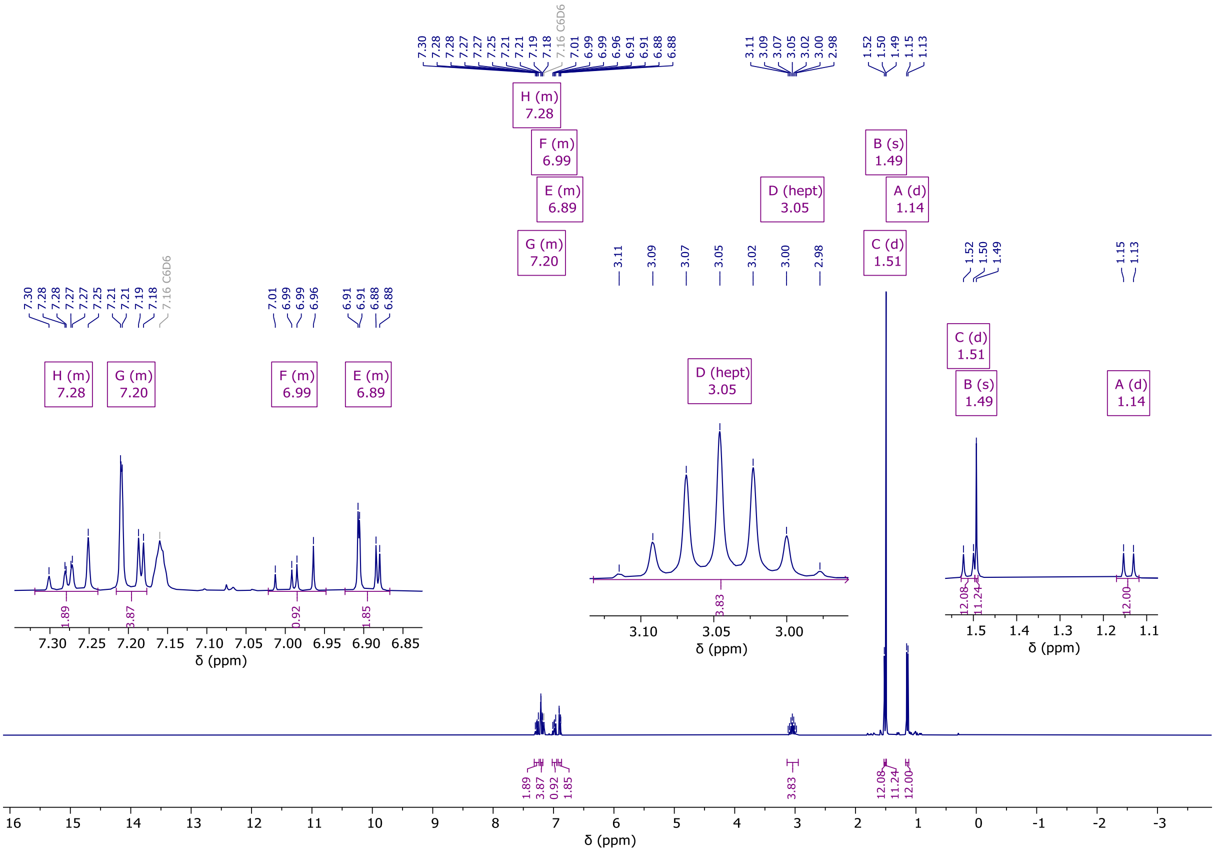

Figure S46: $^{13}C\{^1H\}$ NMR spectrum of ^{Dipp}TerAsAlCp* and comparison of the aromatic region in the $^{13}C\{^1H\}$ and ^{13}C-dept-135 NMR spectrum, showing an overlap of signals from ^{Dipp}TerAsAlCp* and C_6D_6 (75.5 MHz, C_6D_6, 298 K).

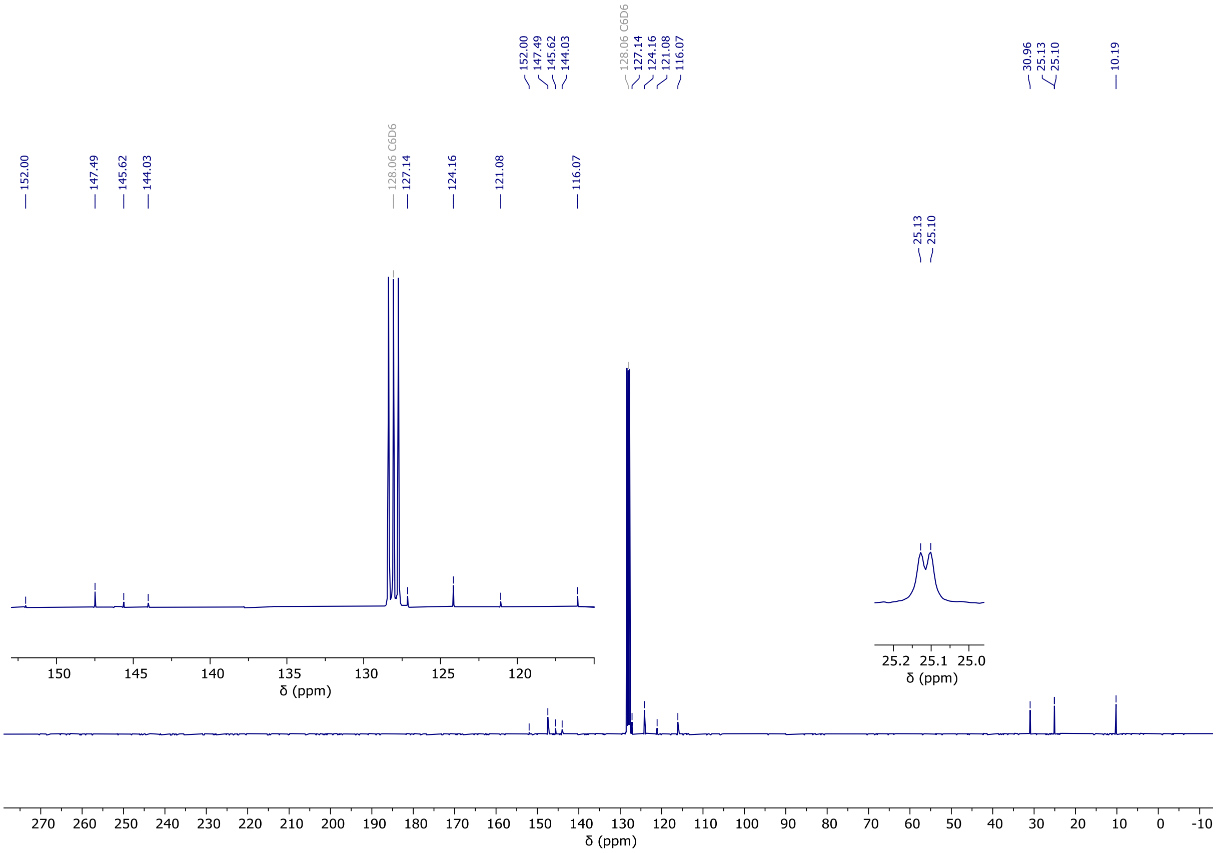

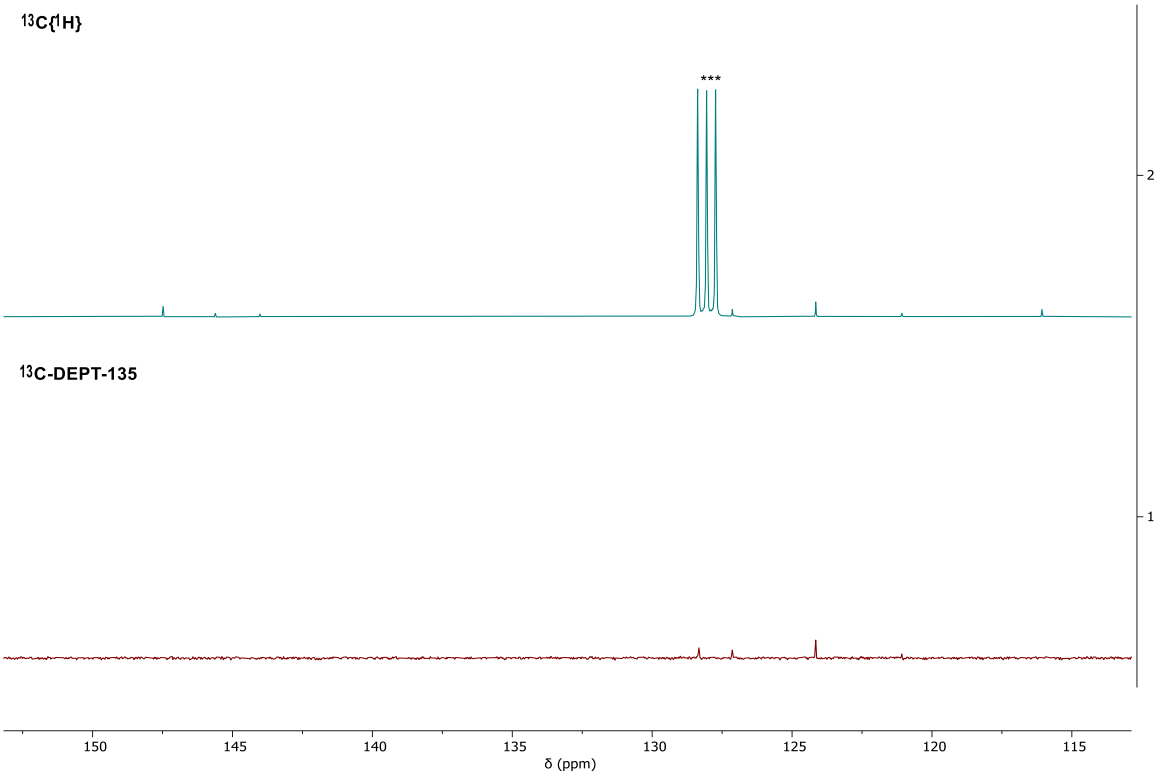

Figure S47: IR spectrum of ^{Dipp}TerAsAlCp* (ATR, 32 scans, cm^{-1}, powder).

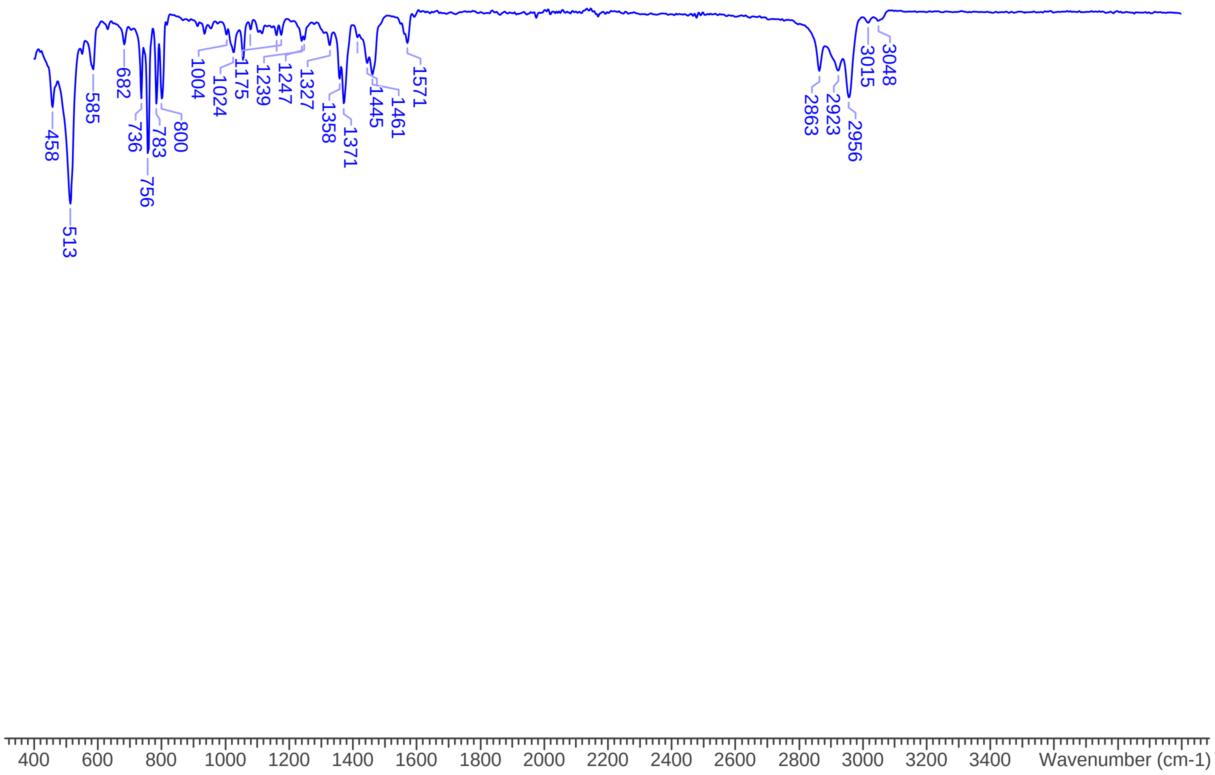

4. Syntheses of compounds

4.1 Compound 1P by reaction of DippTerPAlCp* with phenylazide

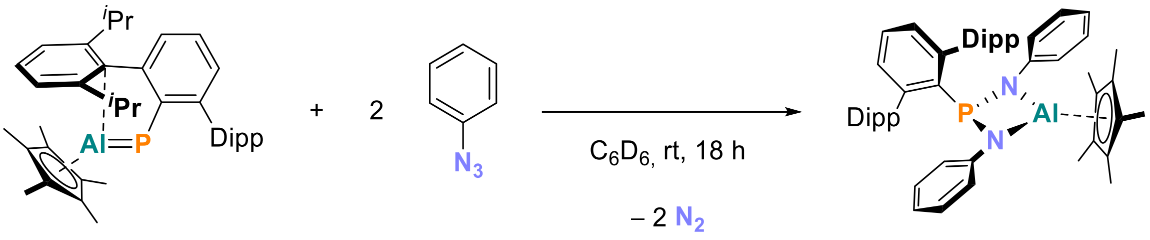

1P (55%)

DippTerPAlCp* (20.0 mg, 33.9 μmol, 1.00 eq.) was dissolved in C_6D_6 (0.4 mL) in a *J.-Young* NMR tube and phenylazide (8.3 mg, 71.2 μmol, 2.10 eq.) was added at ambient temperature. Upon addition, the colour of the reaction mixture instantly changed from deep purple to red. The sample was placed on a laboratory shaking plate at ambient temperature for 18 h. After 18 h, full conversion of **DippTerPAlCp*** to **1P** was confirmed via $^{31}P\{^{1}H\}$ NMR spectroscopy. The reaction mixture was cooled to –78 °C (N_2(l)/*i*-PrOH cooling bath) and the solvent was removed via sublimation *in vacuo* (~1×10^{-3} mbar) (lyophilisation). In a glovebox, the resulting orange powder was transferred to a pipette equipped with a glass microfiber paper and washed with cold (–30 °C) HMDSO (2 × 0.2 mL). The remaining pale orange solid was re-dissolved in benzene (0.5 mL), transferred into a *J.-Young* NMR tube, and lyophilised again to remove remaining traces of HMDSO and to obtain the analytically pure compound **1P**.

Yield of 1P: 14.3 mg (18.5 μmol, 55%) of a pale orange powder.

Suitable crystals for single crystal X-ray diffraction were obtained by slow evaporation of a saturated solution of **1P** in benzene at ambient temperature in a glovebox.

CHN calc.[iv] (found) in %: C 80.79 (73.42), H 8.08 (7.25), N 3.62 (4.15). **^{1}H NMR** (500.1 MHz, C_6D_6, 298 K): δ = 0.99 (d, $^3J_{(H-H)}$ = 6.8 Hz, 12 H, (C(H)(C*H*$_3$)(CH$_3$)), 1.02 (d, $^3J_{(H-H)}$ = 6.8 Hz, 12 H, C(H)(C*H*$_3$)(CH$_3$)), 1.57 (s, 15 H, C$_5$(C*H*$_3$)$_5$), 3.00 (ψ-hept, $^3J_{(H-H)}$ = 6.8 Hz, 4 H, C(*H*)(CH$_3$)(CH$_3$)), 6.81 – 6.85 (m, 6 H, *o*- and *p-H* (Ph)), 7.02 – 7.04 (m, 4 H, *m-H* (Ph)), 7.05 – 7.06 (m, 1 H, *p-H* (^{Dipp}Ter)), 7.07 – 7.09 (m, 2 H, *m-H* (^{Dipp}Ter)), 7.21 (d, $^3J_{(H-H)}$ = 7.6 Hz, 4 H, *m-H* (Dipp)), 7.34 (t, $^3J_{(H-H)}$ = 7.6 Hz, 2 H, *p-H* (Dipp)) ppm. **^{13}C{^{1}H} NMR** (125.8 MHz, C_6D_6, 298 K): δ = 11.1 (s, C$_5$(*C*H$_3$)$_5$), 22.9 (s, (CH(*C*H$_3$)(CH$_3$))$_2$), 26.1 (s, (CH(CH$_3$)(*C*H$_3$))$_2$), 31.3 (d, $J_{(C-P)}$ = 1.1 Hz, (*C*H(CH$_3$)(CH$_3$))$_2$), 115.4 (s, *C*$_5$(CH$_3$)$_5$), 120.5 (d, $J_{(C-P)}$ = 1.8 Hz, *p*-CH (Ph)), 122.9 (s, *m*-CH (Dipp)), 125.4 (d, $J_{(C-P)}$ = 13.8 Hz, *o*-CH (Ph)), 127.4 (s, *p*-CH or *m*-CH (^{Dipp}Ter)), 128.0 (s, *p*-CH (Dipp))[v], 28.4 (s, *m*-Ph-CH)[v], 132.5 (s, *p*-CH or *m*-CH (^{Dipp}Ter)), 140.9 (d, $J_{(C-P)}$ = 5.0 Hz, *i-C* (Dipp)), 146.3 (d, $J_{(C-P)}$ = 44.4 Hz, *o-C* (^{Dipp}Ter), 146.5 (d, $J_{(C-P)}$ = 68.3 Hz, *i-C*-P (^{Dipp}Ter)), 147.4 (d, $J_{(C-P)}$ = 1.2 Hz, *o-C* (Dipp)), 149.5 (d, $J_{(C-P)}$ = 16.9 Hz, *i-C* (Ph)) ppm. **^{31}P{^{1}H} NMR** (202.6 MHz, C_6D_6, 298 K): δ = 84.2 (s) ppm. **IR** (ATR, 32 scans, cm^{-1}): $\tilde{\nu}$ = 3360 (w), 3055 (w), 2960 (m), 2923 (w), 2867 (w), 1599 (m), 1496 (m), 1470 (m), 1443 (w), 1430 (w), 1383 (m), 1362 (w), 1286 (m), 1270 (m), 1249 (m), 1231 (m), 1177 (w), 1154 (w), 1125 (w), 1103 (w), 1078 (w), 1055 (w), 1031 (w), 996 (w), 983 (w), 954 (w), 909 (m), 870 (m), 822 (m), 806 (m), 794 (m), 748 (vs), 690 (s), 662 (m), 627 (m), 585 (m), 552 (m), 532 (m), 521 (m), 503 (s), 451 (s), 431 (m), 410 (m). **LIFDI-MS** (toluene): m/z calc: 772.4466 (100%); [$C_{52}H_{62}AlN_2P$]; m/z found: 569.3076 [M$^+$−AlCp*−*i*-Pr+2H].

[iv] Deviations likely due to incomplete combustion, repeated measurements did not give better results.
[v] Superimposed with C_6D_6 signal, assigned with ^{1}H/^{13}C HSQC NMR, DEPT-90 and DEPT-135.

Figure S48: ^{1}H NMR spectrum of isolated **1P** (500.1 MHz, C_6D_6, 298 K); 0.12 ppm: 10% HMDSO.

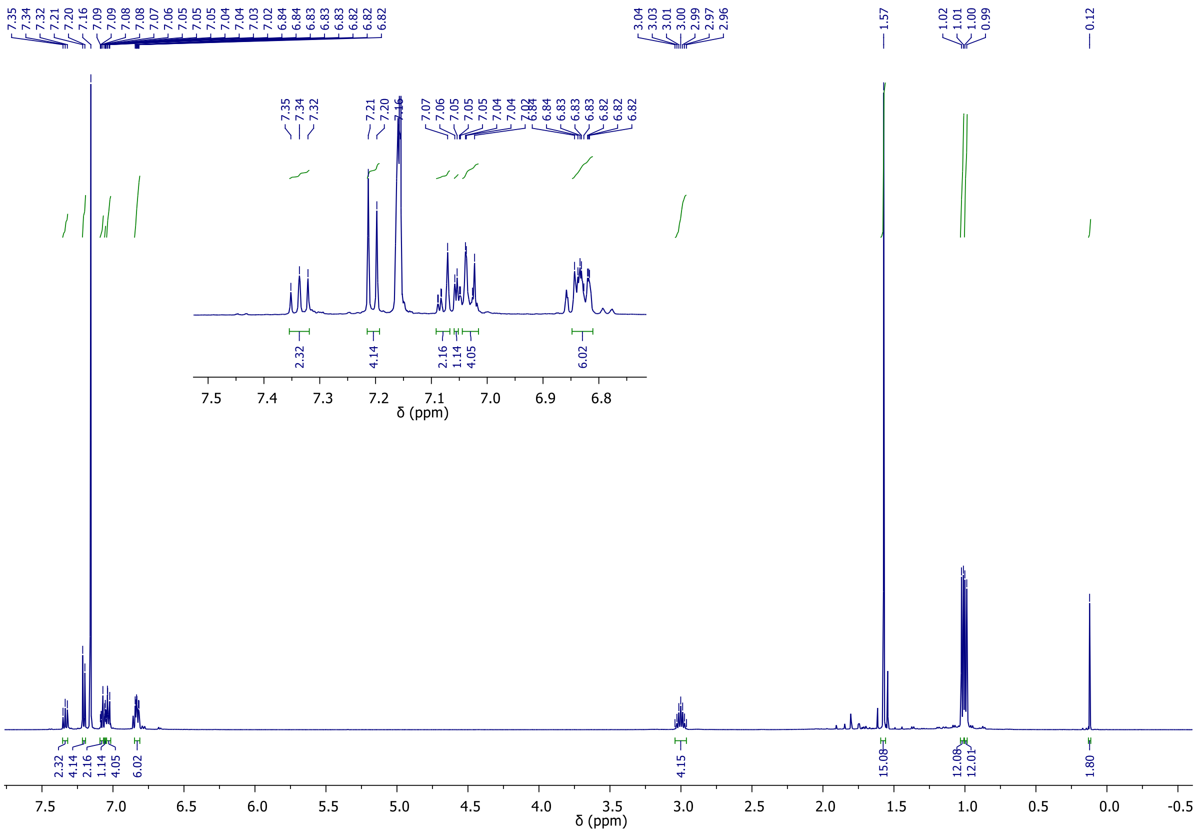

Figure S49: ^{13}C{^{1}H} NMR spectrum of isolated **1P** (125.8 MHz, C_6D_6, 298 K); 2.08 ppm HMDSO.

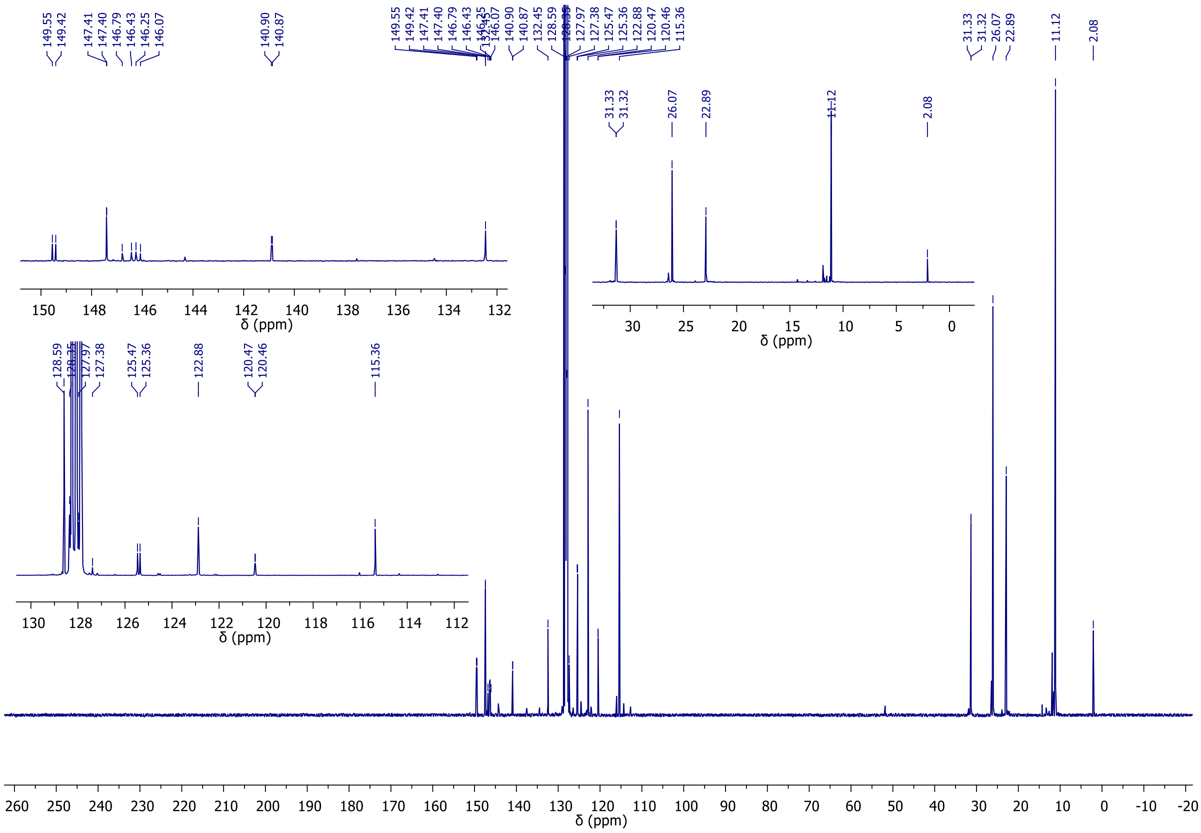

Figure S50: $^{31}P\{^1H\}$ NMR spectrum of isolated **1P** (202.6 MHz, C_6D_6, 298 K).

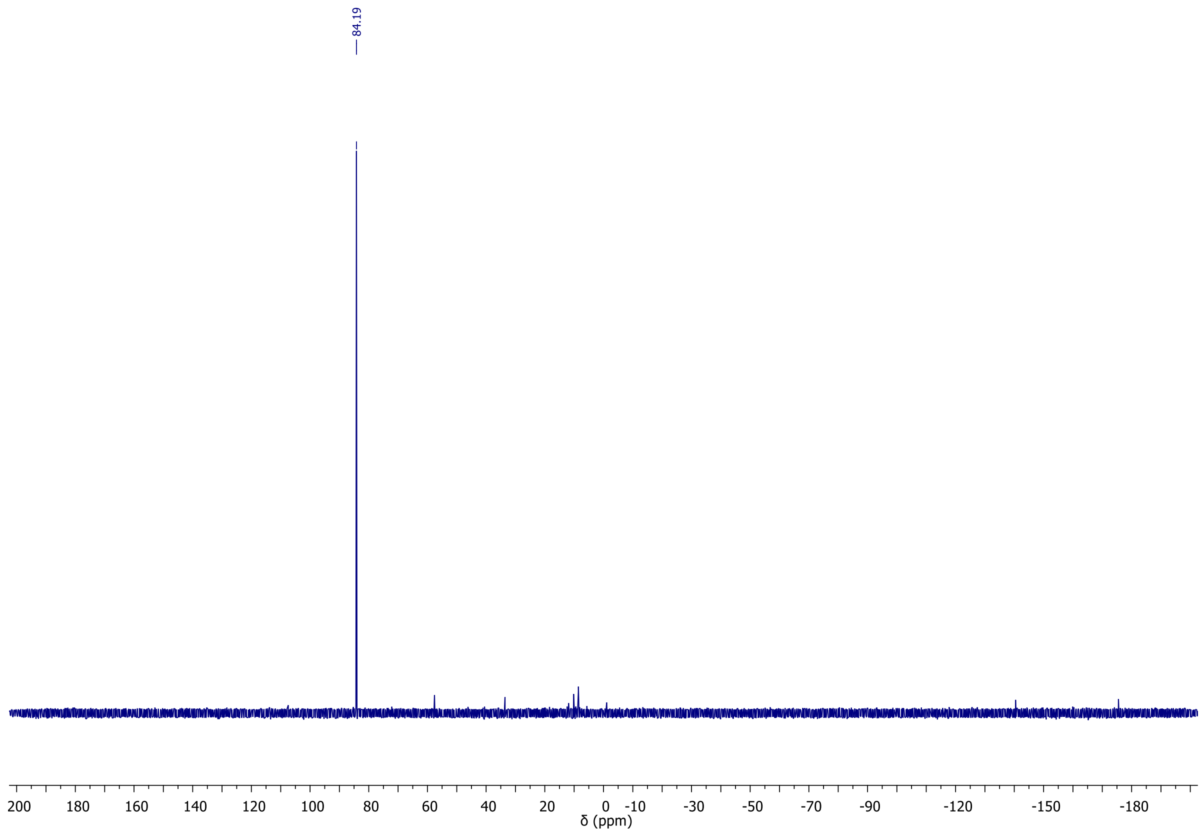

Figure S51: IR-spectrum of **1P** (ATR, 32 scans, cm^{-1}, powder; mode at 3360 cm^{-1} might arise from partial hydrolysis during measurement).

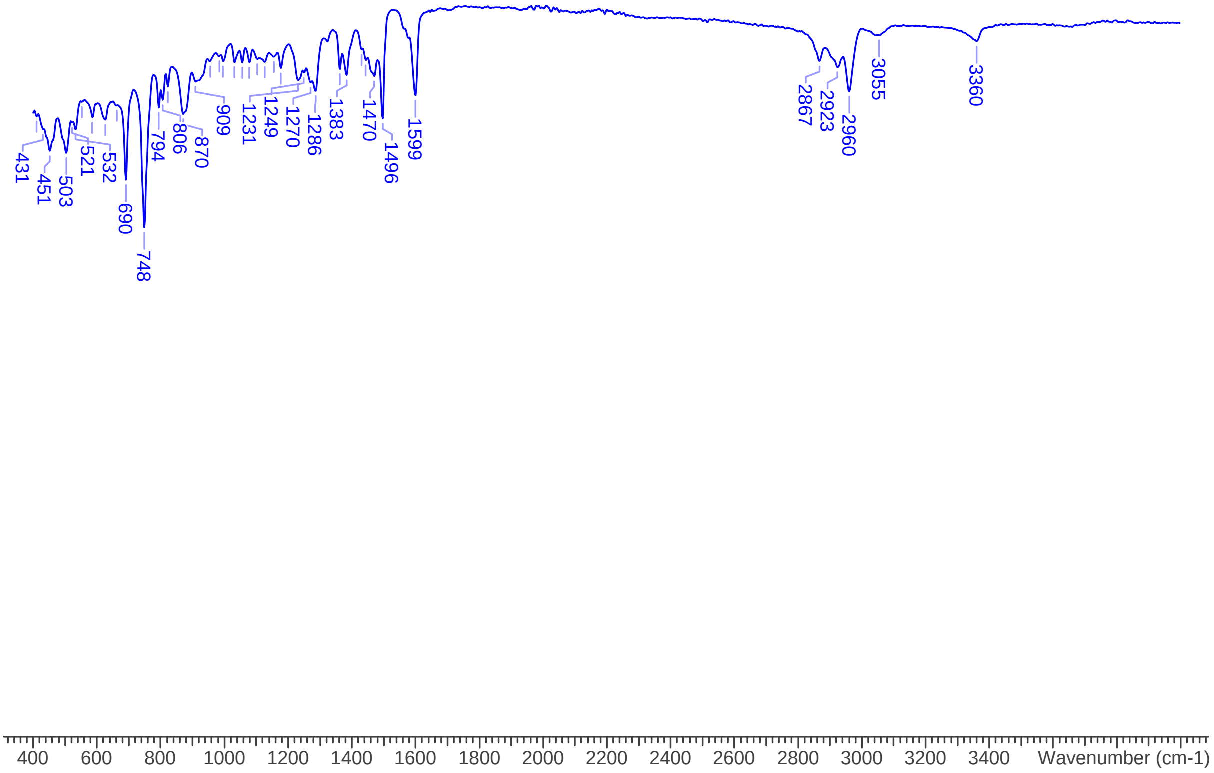

Figure S52: LIFDI mass spectrum of **1P** (toluene).

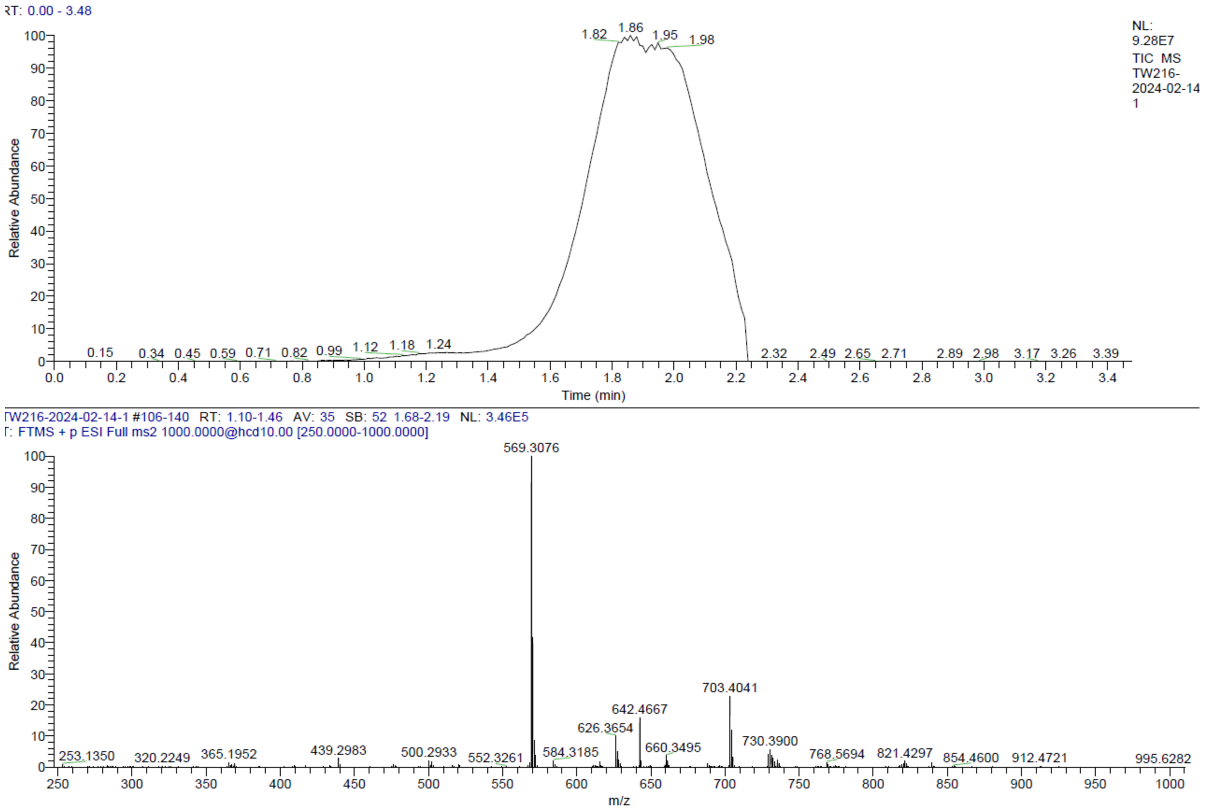

4.2 Attempts to synthesize 1As

It was tried to synthesize **1As** by two different approaches starting from ^{Dipp}TerAsAlCp*. The reaction mixtures after full conversion are compared at the end of this section by ^{1}H NMR spectroscopy (see figure S52).

a) Reaction of ^{Dipp}TerPAlCp* with phenylazide

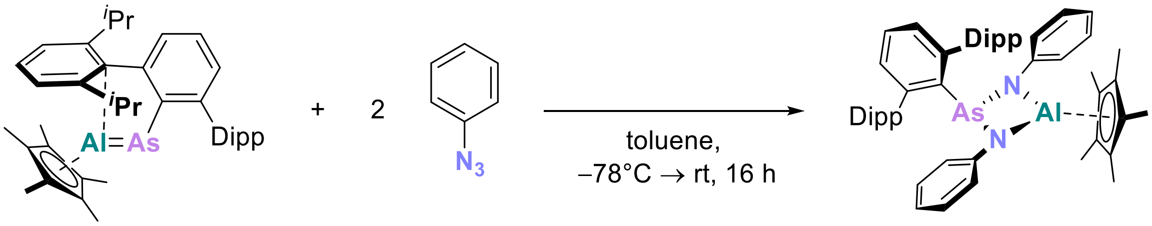

^{Dipp}TerAsAlCp* (50 mg, 79 μmol) was dissolved in toluene (2.5 mL) at ~40 °C (water bath). Afterwards, the solution was cooled to −78 °C (CO_2/EtOH cooling bath). At this temperature phenylazide (0.33 mL, 0.5 M in 2-methyltetrahydrofuran, 165 μmol) was added in the absence of light over a period of 2 min. During addition the colour changed from blue to wine red. The reaction mixture was allowed to warm to ambient temperature in the cooling bath overnight, giving an orange solution. The solvent was removed *in vacuo* and the residue was dried for 30 min at 1×10^{-3} mbar at ambient temperature. The residue was analysed by ^{1}H NMR spectroscopy (see Figure S52, middle), indicating a rather selective transformation. Unfortunately, all attempts to isolate the major product by crystallization from toluene or *n*-hexane have not been successful. One of the side-products of the reaction, ^{Dipp}TerAs(NHPh)$_2$ was identified by SC-XRD experiments, however, with only minimal amount of crystalline material this species was not characterized further. Only the signals of **1As** that could clearly be identified in the ^{1}H NMR spectrum are listed below.

^{1}H NMR (300.1 MHz, C_6D_6, 298 K): δ = 1.00 (d, $^{3}J_{(H-H)}$ = 6.7 Hz, 12 H, (C(H)(C*H*$_3$)(CH$_3$)), 1.01 (d, $^{3}J_{(H-H)}$ = 6.8 Hz, 12 H, C(H)(C*H*$_3$)(CH$_3$)), 1.61 (s, 15 H, C$_5$(C*H*$_3$)$_5$), 2.97 (hept, $^{3}J_{(H-H)}$ = 6.8 Hz, 4 H, C(*H*)(CH$_3$)(CH$_3$)), 7.21 (d, $^{3}J_{(H-H)}$ = 7.6 Hz, 4 H, *m-H* (Dipp)), 7.35 (t, $^{3}J_{(H-H)}$ = 7.6 Hz, 2 H, *p-H* (Dipp)) ppm.

b) Reaction of ^{Dipp}TerPAlCp* with azobenzene

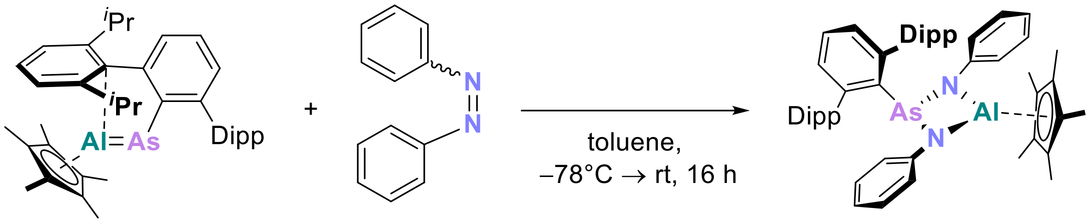

^{Dipp}TerAsAlCp* (50 mg, 79 μmol) was dissolved in toluene (2.5 mL) at ~40 °C (water bath). Afterwards, the solution was cooled to −78 °C (CO_2/EtOH cooling bath) and a solution of azobenzene (14 mg, 79 µmol) in toluene (2.5 mL) was added over a period of 5 min in the absence of light. The reaction mixture was allowed to warm to ambient temperature in the cooling bath overnight. During reaction the color of the reaction mixture changed from blue to green. The solvent was removed *in vacuo* and the residue was dried for 30 min at 1×10^{-3} mbar at ambient temperature. The residue was analysed by ^{1}H NMR spectroscopy (see Figure 52, bottom), indicating selectivity problems in the reaction. Unfortunately, all attempts to isolate one of the products by crystallization from toluene or *cyclo*-hexane have not been successful.

Figure S53: Comparison of the ^{1}H NMR spectra after conversion of ^{Dipp}TerAsAlCp* (top) with PhN_3 (middle) or azobenzene (bottom) (300.1 MHz, C_6D_6, 298 K)).

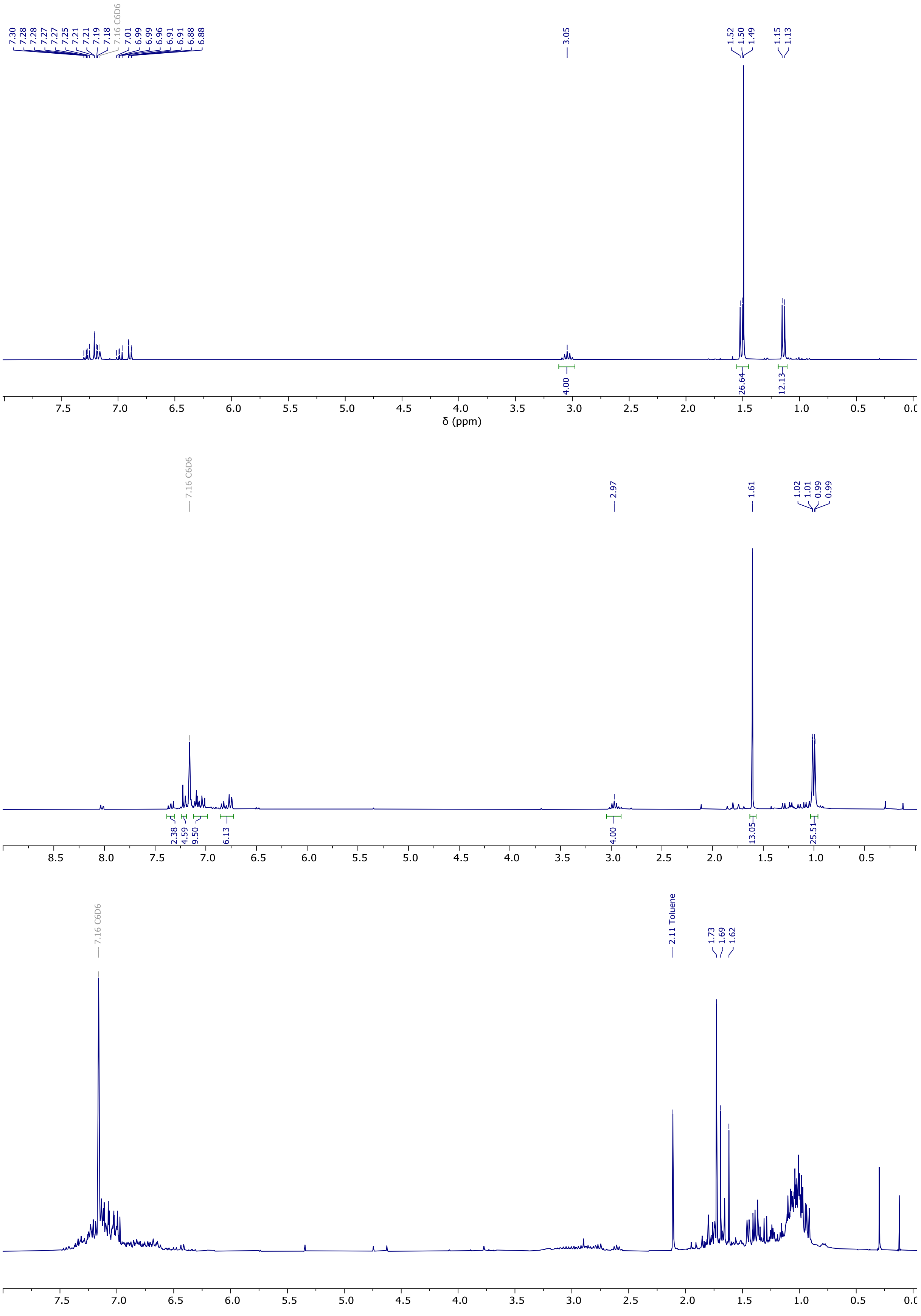

4.3 Compound 1P by reaction of ^{Dipp}TerPAlCp* with *E/Z*-azobenzene

Reaction with *E*-azobenzene

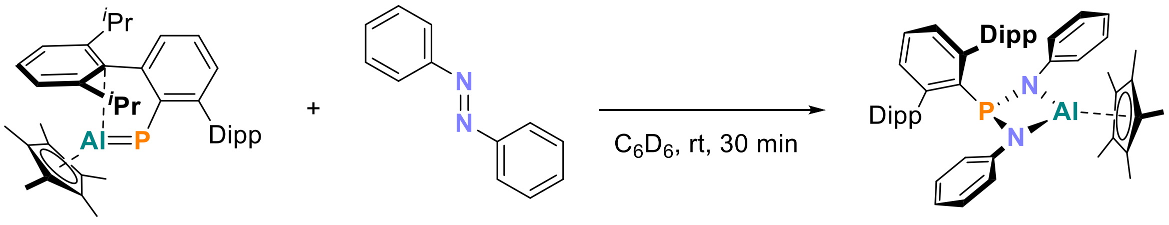

1P (n.d.)

^{Dipp}TerPAlCp* (20.0 mg, 34.0 μmol, 1.00 eq.) was dissolved in C_6D_6 (0.4 mL) in a *J.-Young* NMR tube and *E*-azobenzene (6.20 mg, 36.0 μmol, 1.05 eq.) was added at ambient temperature. An immediate colour change from deep purple to red orange was observed and after 30 min compound **1P** was identified via $^{31}P\{^1H\}$ NMR spectroscopy (1:1 ratio with a second, unidentified reaction product). Compound **1P** was not isolated from this reaction.

^{1}H NMR (400.3 MHz, C_6D_6, 298 K): δ = 0.99 (d, $^3J_{(H-H)}$ = 6.8 Hz, 12 H, C(H)(C*H*)$_3$(CH$_3$), **1P**), 1.02 (d, J = 6.8 Hz, 12 H, C(H)(CH)$_3$(C*H*$_3$), **1P**), 1.12 – 1.04 (m, 24 H, C(H)(C*H*)$_3$(C*H*$_3$), unidentified compound), 1.50 (s, 3 H, C$_5$(C*H*$_3$)(CH$_3$)$_2$(CH$_3$)$_2$, unidentified compound), 1.57 (s, 15 H, C$_5$(C*H*$_3$)$_5$, **1P**), 1.70 – 1.68 (m, 6 H, C$_5$(CH$_3$)(C*H*$_3$)$_2$(CH$_3$)$_2$, unidentified compound), 1.87 – 1.84 (m, 6 H, C$_5$(CH$_3$)(CH$_3$)$_2$(C*H*$_3$)$_2$, unidentified compound), 2.92 (m, 4 H, C(*H*)(CH)$_3$(CH$_3$), unidentified compound), 3.00 (ψ-hept, 4 H, C(*H*)(CH)$_3$(CH$_3$), **1P**) ppm.[vi] **P{^{1}H} NMR** (162.0 MHz, C_6D_6, 298 K): δ = 84.2 (s, **1P**), –148.6 (s, unidentified product) ppm.

[vi] Further assignment of the ^{1}H NMR resonances not possible form the spectra obtained from this reaction, due to strong overlap in the aryl area. See 4.1 for full characterization of compound **1P**.

Figure S54: ^{1}H NMR spectrum of the crude reaction mixture of **^{Dipp}TerPAlCp*** and *E*-azobenzene (400.3 MHz, C_6D_6, 298 K), marked signals correspond to **1P**.

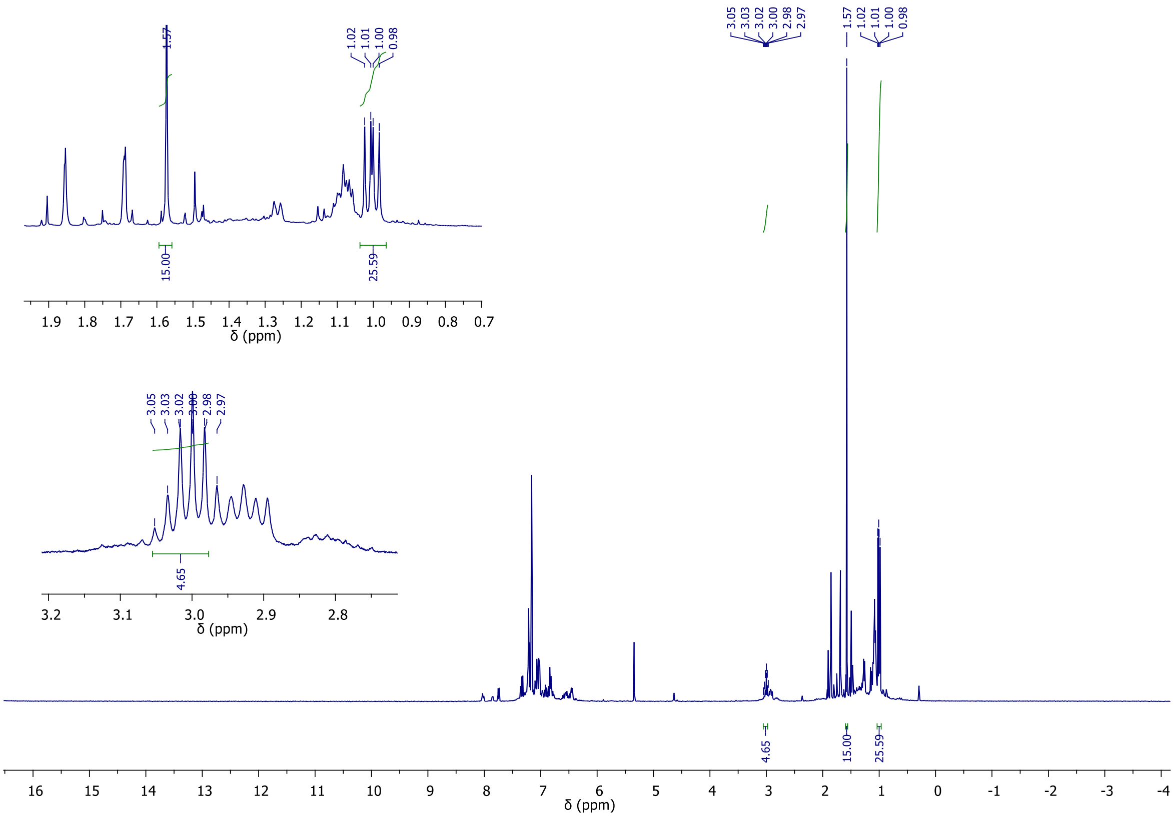

Figure S55: ^{1}H NMR spectrum of the crude reaction mixture of **^{Dipp}TerPAlCp*** and *E*-azobenzene (400.3 MHz, C_6D_6, 298 K), marked signals correspond to the second, unidentified reaction product.

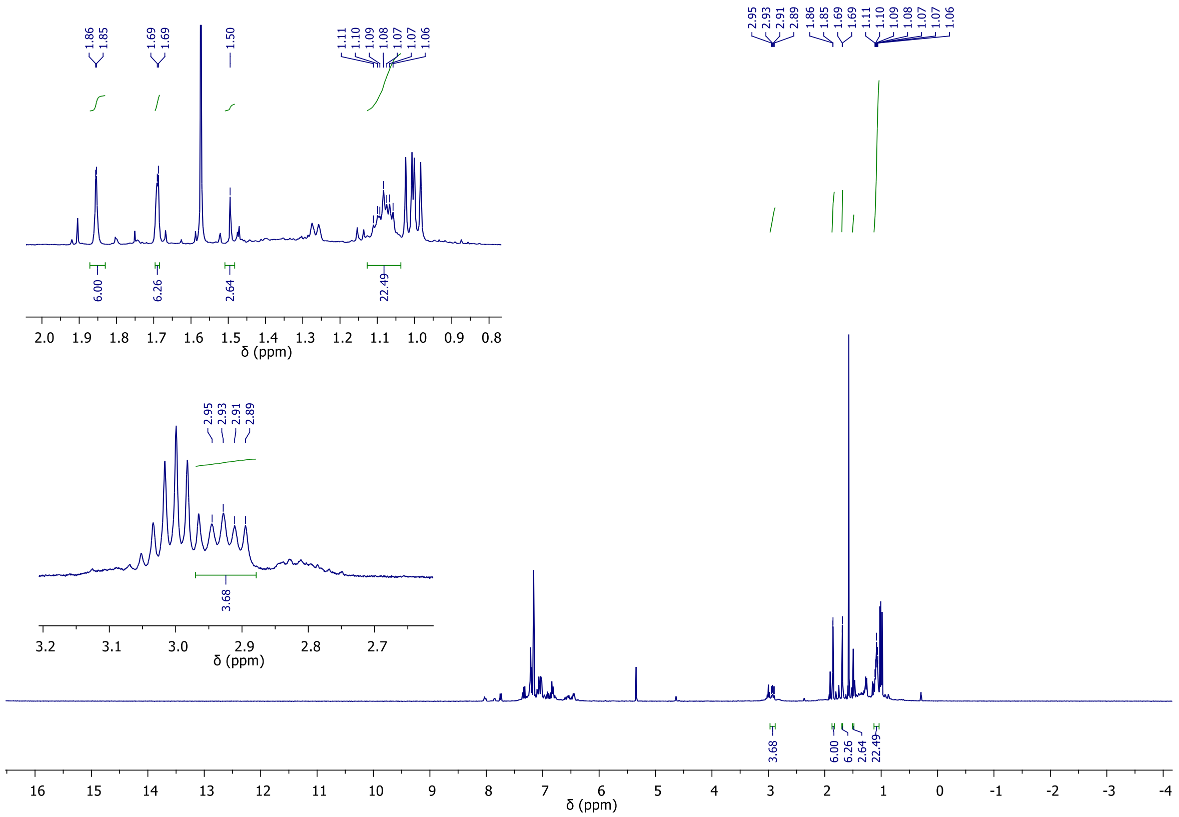

Figure S56: $^{31}P\{^1H\}$ NMR spectrum of the crude reaction mixture of **^{Dipp}TerPAlCp*** and *E*-azobenzene (162.0 MHz, C_6D_6, 298 K). Left resonance: **1P**, Right resonance: unidentified second product.

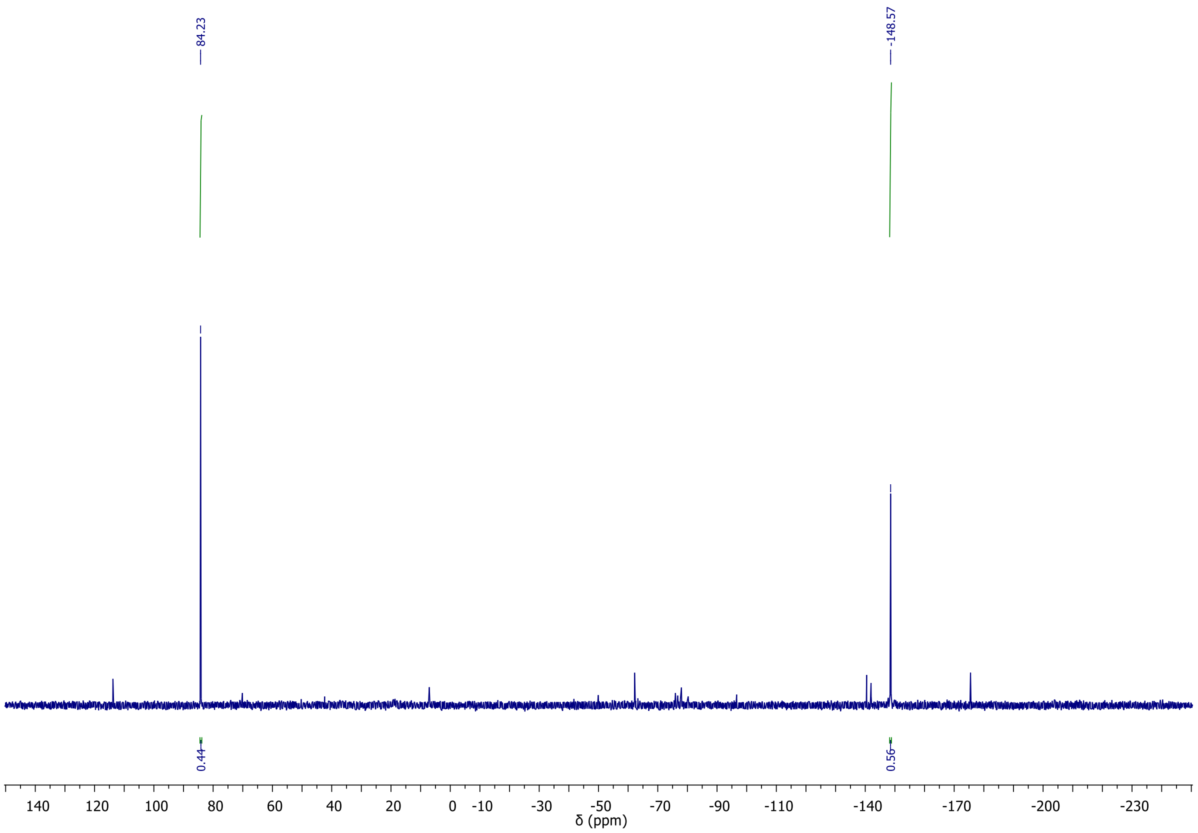

Reaction with *Z*-azobenzene

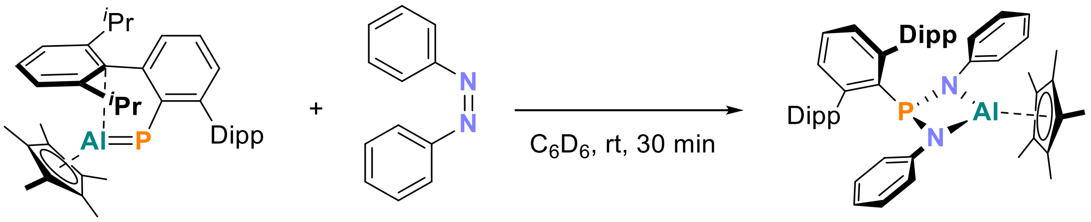

1P (n.d.)

^{Dipp}TerPAlCp* (10.0 mg, 17.0 μmol, 1.00 eq.) was dissolved in C_6D_6 (0.4 mL) in a brown glass *J.-Young* NMR tube and *Z*-azobenzene (6.20 mg, 18.0 μmol, 1.05 eq.) was added at ambient temperature under light exclusion. After 30 min compound **1P** was identified as the main reaction product via $^{31}P\{^{1}H\}$ NMR spectroscopy. **1P** was not isolated from this reaction.

^{1}H NMR (400.3 MHz, C_6D_6, 298 K): δ = 0.99 (d, $^{3}J_{(H\text{-}H)}$ = 6.9 Hz, 12 H, C(H)(C*H*)$_3$(CH$_3$)), 1.01 (d, $^{3}J_{(H\text{-}H)}$ = 6.8 Hz, 12 H, C(H)(CH)$_3$(C*H*$_3$)), 1.57 (s, 15 H, $C_5(CH_3)_5$), 3.06 – 2.95 (m, 4 H, C(*H*)(CH)$_3$(CH$_3$)) ppm.[vii] **$^{31}P\{^{1}H\}$ NMR** (162.0 MHz, C_6D_6, 298 K): δ = 84.2 (s) ppm.

[vii] Further assignment of the ^{1}H NMR resonances not possible form the spectra obtained from this reaction, due to strong overlap in the aryl area. See 4.1 for full characterization of compound **1P**.

Figure S57: ^{1}H NMR spectrum of the crude reaction mixture of **^{Dipp}TerPAlCp*** and *Z*-azobenzene (400.3 MHz, C_6D_6, 298 K), marked signals correspond to **1P**; 0.29 ppm silicon grease.

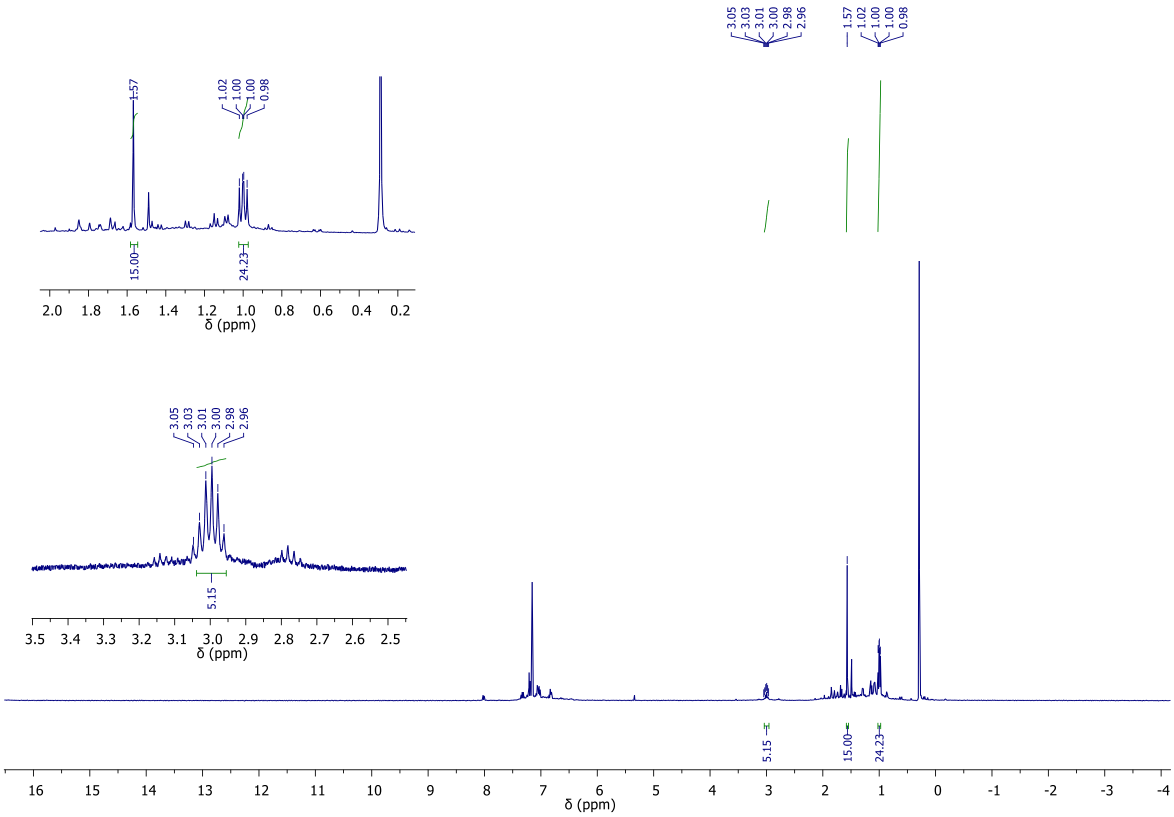

Figure S58: $^{31}P\{^{1}H\}$ NMR spectrum of the crude reaction mixture of **^{Dipp}TerPAlCp*** and *Z*-azobenzene (162.0 MHz, C_6D_6, 298 K); −140.5 ppm ^{Dipp}TerPH$_2$.

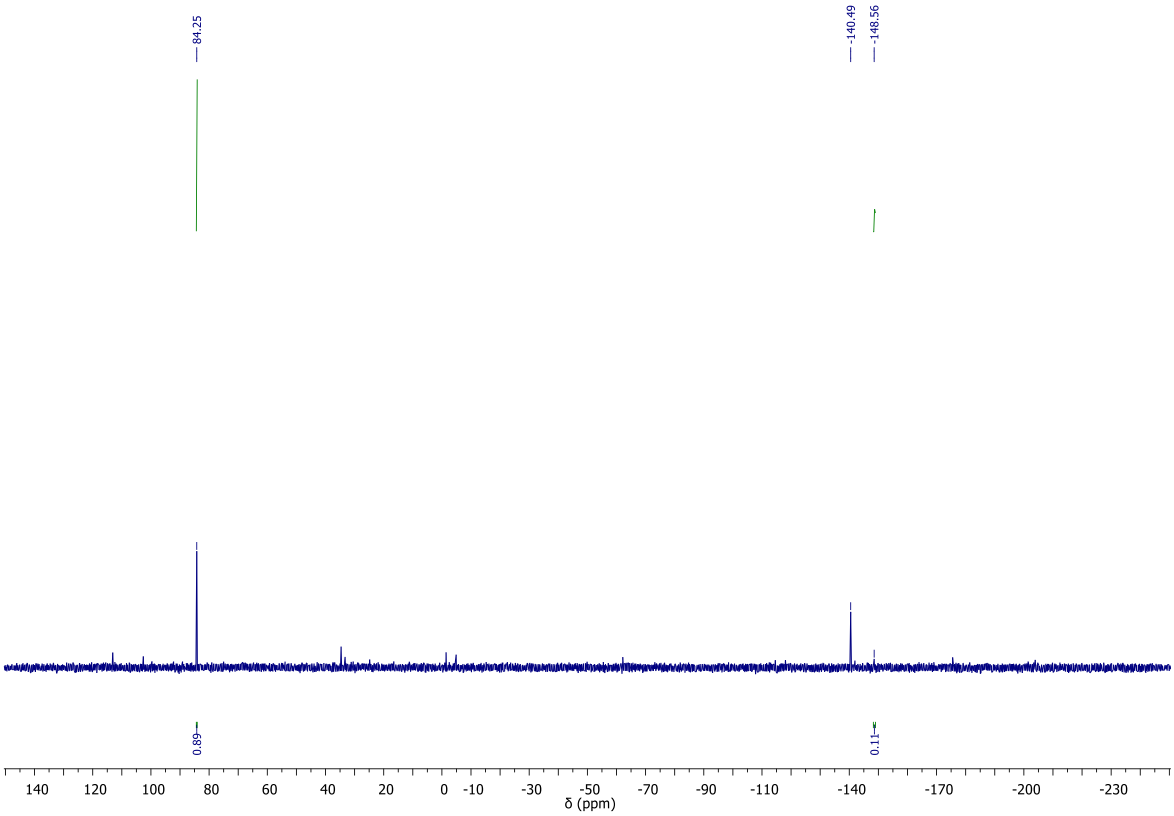

4.4 Compound 1P • PMe₃

Reaction of freshly prepared ^{Dipp}TerPAlCp* with *E/Z*-azobenzene

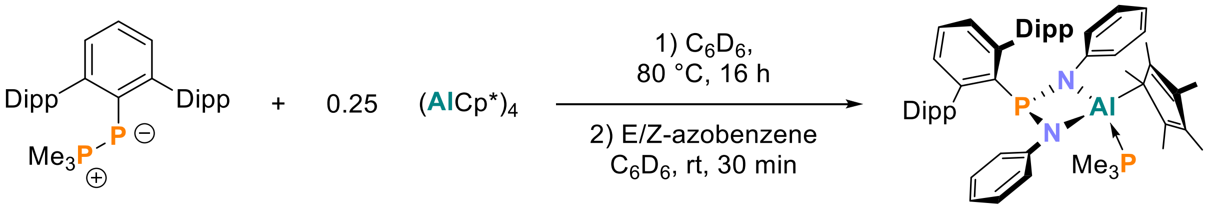

1P•PMe₃ (n.d.)

^{Dipp}TerPPMe₃ (30.0 mg, 56.0 µmol, 1.00 eq.) and **(AlCp*)₄** (13.6 mg, 21.0 µmol, 0.38 eq.) were dissolved in C_6D_6 (0.4 mL) in a *J.-Young* NMR tube and heated overnight at 80 °C (heating block). After 16 h, full conversion to **^{Dipp}TerPAlCp*** and liberation of PMe_3 was confirmed via $^{31}P\{^1H\}$ and ^1H NMR spectroscopy. At ambient temperature in a glovebox, *E*-azobenzene (10.2 mg, 56.0 µmol, 1.00 eq.) was added and an immediate colour change from deep purple to red-orange was observed. After 30 min, compound **1P•PMe₃** was identified via $^{31}P\{^1H\}$ NMR spectroscopy. The analytically pure compound **1P•PMe₃** could not be isolated from this reaction, hence no yield could be determined.[viii]

Few suitable crystals for single crystal X-ray diffraction were obtained by slow evaporation of a saturated solution of **1P•PMe₃** in *n*-pentane at −30 °C in a glovebox after 3 d.

[viii] See „Reaction of isolated **1P** with PMe_3 for additional analytical data of compound **1P•PMe₃**.

Reaction of isolated ^{Dipp}TerPAlCp* and PMe_3 with *E/Z*-azobenzene

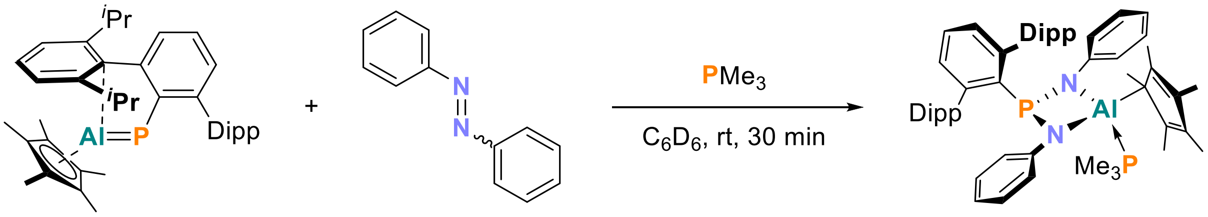

1P•PMe₃ (n.d.)

^{Dipp}TerPAlCp* (20.0 mg, 33.9 μmol, 1.00 eq.) was dissolved in C_6D_6 (0.4 mL) in a *J.-Young* NMR tube and PMe_3 (1 drop, exc.) was added on a Schlenk line using an NMR tube adapter with a syringe. Then, *E/Z*-azobenzene (6.12 mg, 33.9 μmol, 1.00 eq.) was added at ambient temperature in a glovebox. An immediate colour change from deep purple to red-orange was observed and after 30 min compound **1P•PMe$_3$** was identified via $^{31}P\{^1H\}$ NMR spectroscopy. The analytically pure compound **1P•PMe$_3$** could not be isolated from this reaction, hence no yield could be determined.[ix]

[ix] See „Reaction of isolated **1P** with PMe_3 for additional analytical data of compound **1P•PMe$_3$**.

Reaction of isolated 1P with PMe_3

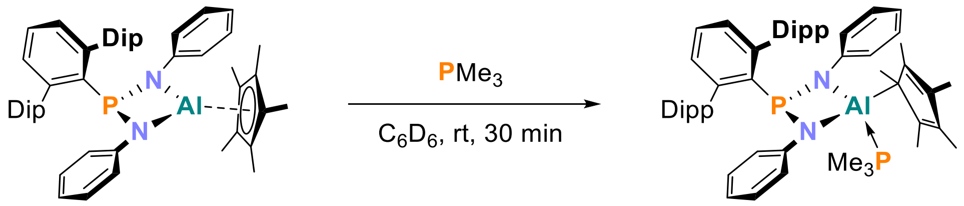

1P•PMe₃ (n.d.)

Compound **1P** (9.00 mg, 11.6 µmol, 1.00 eq.) was dissolved in C_6D_6 (0.4 mL) in a *J.-Young* NMR tube and a solution of PMe_3 (0.7 M in C_6D_6, 0.05 mL, 34.8 µmol, 3.00 eq.) was added at ambient temperature on a Schlenk line using an NMR tube adapter with a syringe. An immediate colour change from a pale orange to yellow was observed and after 30 min, full conversion of **1P** to compound **1P•PMe₃** was confirmed via $^{31}P\{^1H\}$ NMR spectroscopy. All volatile components were removed *in vacuo* (~1×10^{-3} mbar) at ambient temperature and the obtained pale-yellow oil was redissolved in C_6D_6.

Yield of 1P•PMe₃: not determined due to insufficient purity.

^{1}H NMR (400.6 MHz, C_6D_6, 298 K): δ = 0.24 (d, $^3J_{(H\text{-}P)}$ = 5.6 Hz, 9 H, P((C*H*₃)₃), 1.08 – 1.16 (m, 24 H, C(H)(C*H*₃)(CH₃) and C(H)(CH₃)(C*H*₃)),), 1.77 (s, 15 H, C₅(C*H*₃)₅), 3.14 (s, 4 H, C(*H*)(CH₃)(CH₃)), 6.46 – 6.60 (m, 6 H, *H* (Ph)), 6.95 – 7.02 (m, 6 H, *H* (Ph)), 7.25 – 7.39 (m, 9 H, *H* (^{Dipp}Ter and Dipp)) ppm. **^{31}P{^{1}H} NMR** (162.2 MHz, C_6D_6, 298 K): δ = −47.6 (br s, **1P•*P*Me₃**), 108.5 (br s, **1*P*•PMe₃**) ppm. **LIFDI-MS** (toluene): m/z calc: 848.4908 (100%) [$C_{55}H_{71}AlN_2P_2$]; m/z found: 569.3074 [M^+−AlCp*−*i*-Pr−PMe₃+2H].

Figure S59: ^{1}H NMR spectrum of the crude product **1P•PMe₃** (400.6 MHz, C_6D_6, 298 K).

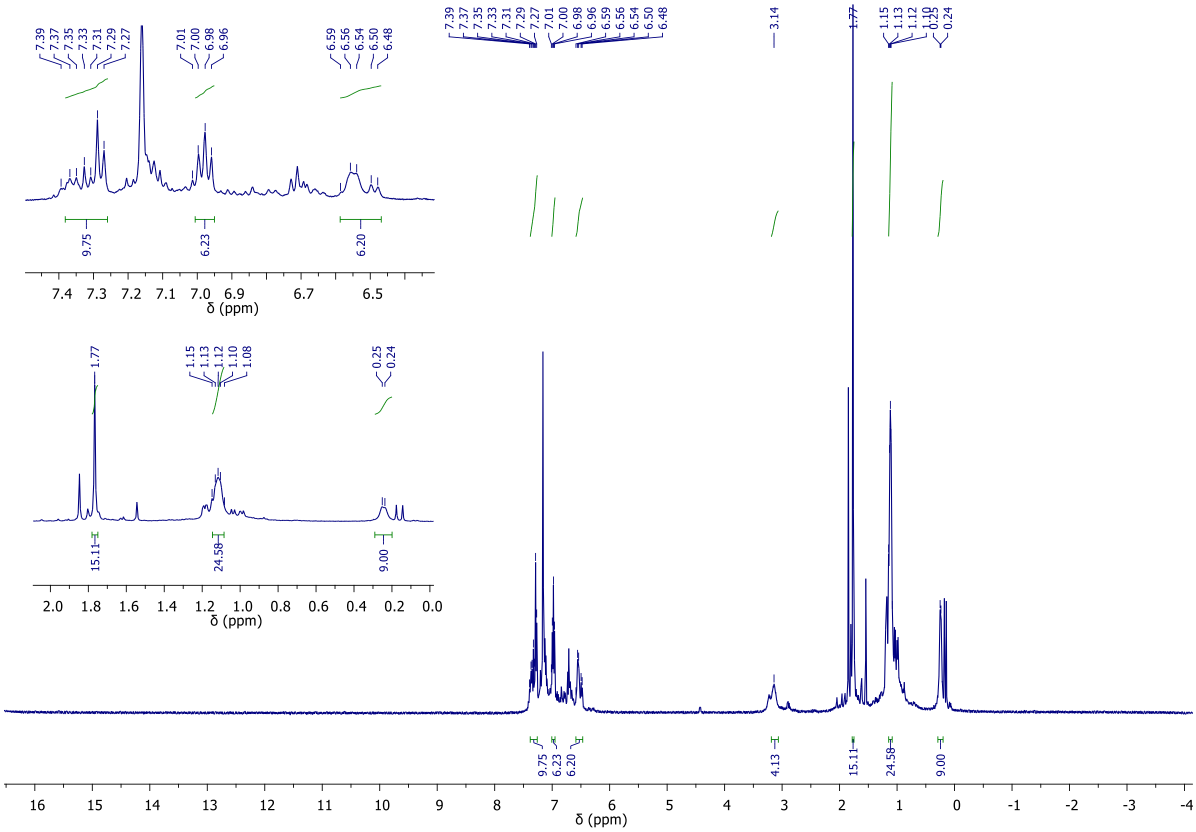

Figure S60: ^{31}P NMR spectrum of crude **1P•PMe₃** (162.2 MHz, C_6D_6, 298 K).

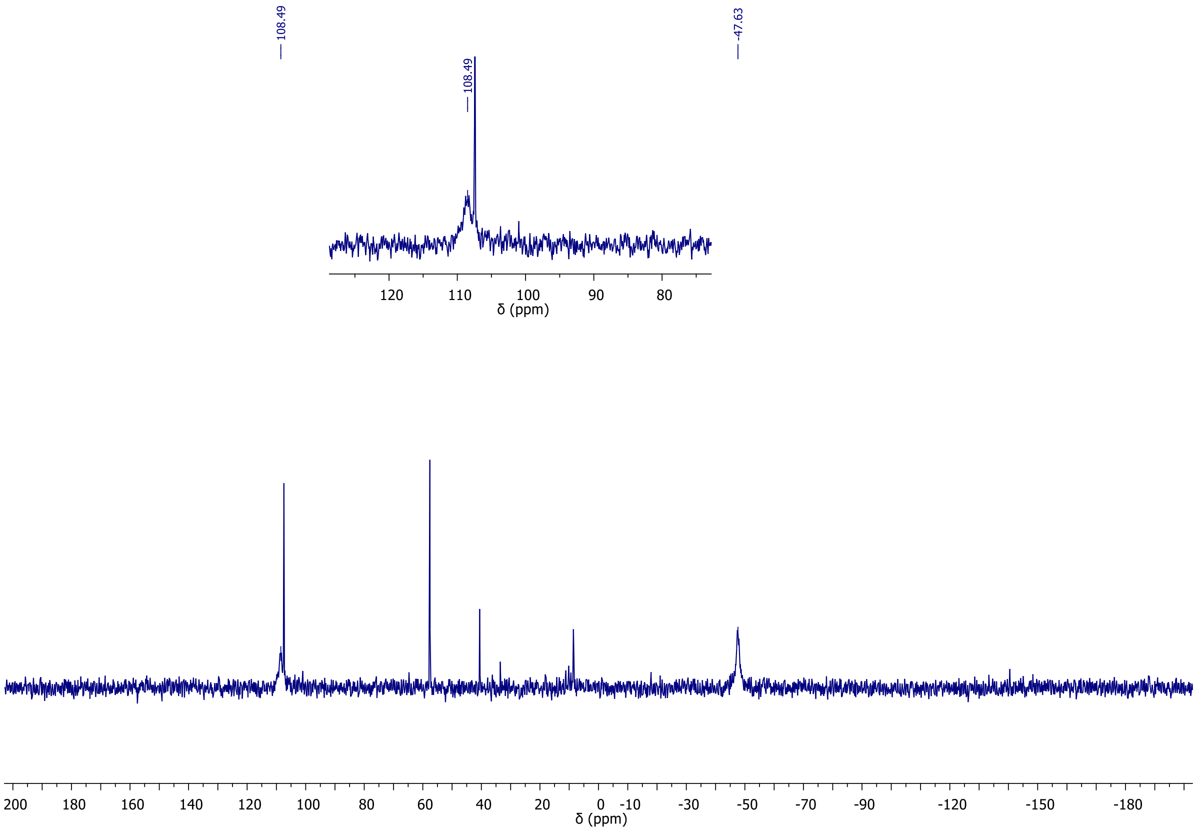

Figure S61: LIFDI mass spectrum of **1P·PMe₃** (toluene).

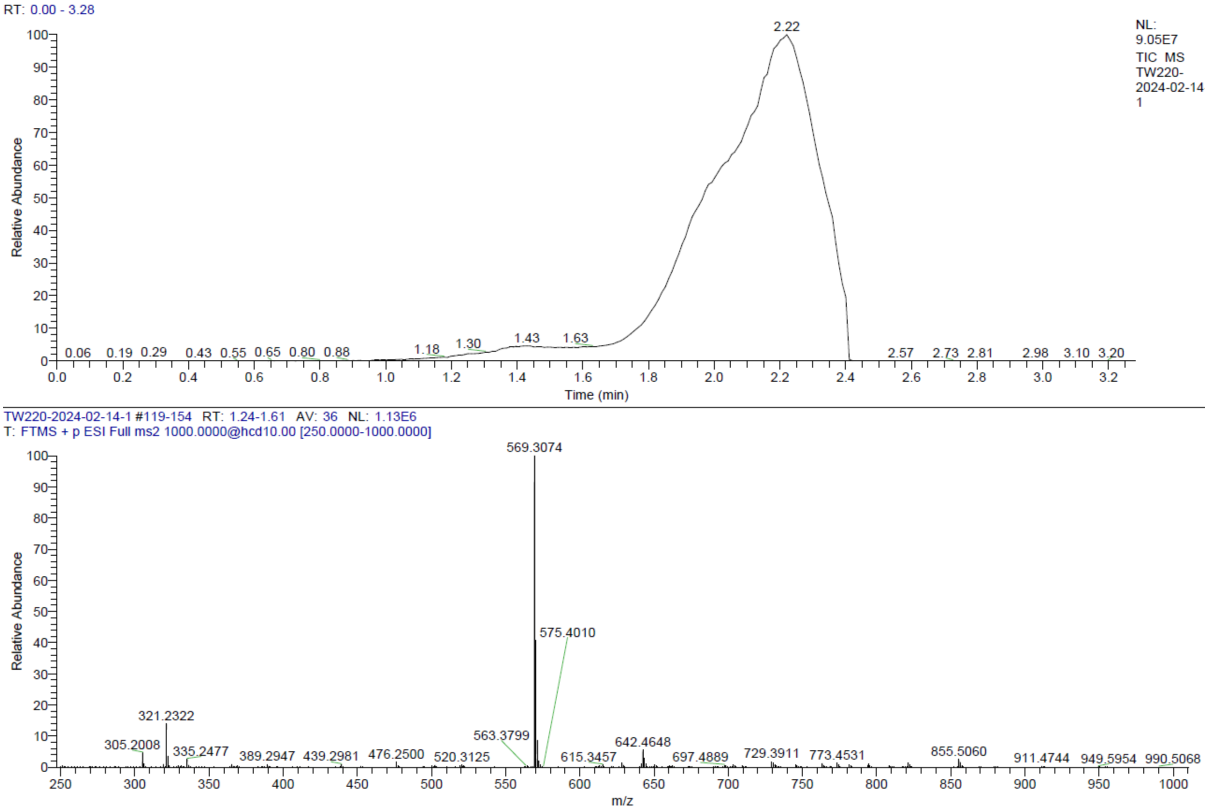

4.5 Compound 1As by reaction of DippTerAsAlCp* with *E/Z*-azobenzene

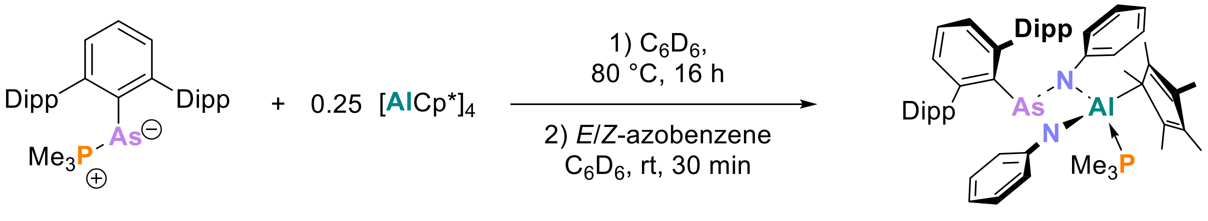

DippTerAsPMe$_3$ (54.0 mg, 98.0 μmol, 1.00 eq.) and **(AlCp*)$_4$** (15.9 mg, 24.5 μmol, 0.25 eq.) were dissolved in C_6D_6 (0.4 mL) in a *J.-Young* NMR tube and heated overnight at 80 °C (oil bath). After 16 h, full conversion to **DippTerAsAlCp*** and liberation of PMe_3 was confirmed via ^{1}H NMR spectroscopy. At ambient temperature in a glovebox, a solution of *E*-azobenzene (17.9 mg, 98.0 μmol, 1.00 eq.) in C_6D_6 (0.1 mL) was added and an immediate colour change from deep blue to orange was observed. After 2 h at room temperature, compound **1As•PMe$_3$** was identified via ^{1}H NMR spectroscopy. Afterwards the solvent was removed in vacuo and the residue was dried for 1 h (1×10^{-3} mbar). The residue was then dissolved in *n*-pentane (1 mL) and filtered through a pipette equipped with a glass filter paper and the filtrate was then placed in the freezer (−30 °C) to give **1As·PMe$_3$** as an orange crystalline solid.

Yield of **1As·PMe$_3$**: 21.0 mg (23.5 μmol, 24%) of a yellow/orange, sticky crystalline solid.

Crystals suitable for single crystal X-ray diffraction were obtained by placing a saturated solution of **1As•PMe$_3$** in *n*-hexane at −30 °C (freezer of the glovebox) for 2 d.

CHN satisfactory values were not obtained, even from isolated single crystals. **^{1}H NMR** (400.1 MHz, C_6D_6, 297 K): δ = 0.29 (d, $^2J_{(P-H)}$ = 7.3 Hz, P(C*H*$_3$)$_3$), 1.11 (d, $^3J_{(H-H)}$ = 6.7 Hz, 12 H, (C(H)(C*H*$_3$)(CH$_3$)), 1.15 (d, $^3J_{(H-H)}$ = 6.8 Hz, 12 H, C(H)(C*H*$_3$)(CH$_3$)), 1.81 (s, 15 H, C$_5$(C*H*$_3$)$_5$), 3.13 (hept, $^3J_{(H-H)}$ = 6.7 Hz, 4 H, C(*H*)(CH$_3$)(CH$_3$)), 6.43 – 6.49 (m, 4 H, *H* (Ph)), 6.64 – 6.72 (m, 2 H, *p-H* (Ph)), 7.05 – 7.06 (m, 1 H, *p-H* (DippTer)), 7.07 – 7.09 (m, 2 H, *m-H* (DippTer)), 6.95 – 7.03 (m, 4 H, *H* (Ph)), 7.11 – 7.18 (m, 3H, *p,m-CH* Ter, overlap with

C_6D_5H), 7.25 – 7.31 (m, 4 H, *m-H* (Dipp)), 7.33 – 7.39 (m, 2 H, *p-H* (Dipp)) ppm. **^{13}C{^{1}H} NMR** (100.6 MHz, C_6D_6, 297 K): δ = 12.4 (d (br), $^{1}J_{(P\text{-}C)}$ = 17.8 Hz, $P(CH_3)_3$), 13.3 (s, $C_5(CH_3)_5$), 22.4 (s, $(CH(CH_3)(CH_3))_2$), 26.6 (s, $(CH(CH_3)(CH_3))_2$), 31.7 (s, $(CH(CH_3)(CH_3))_2$), 117.1 (s, *p*-CH (Ph)), 119.7 (s, *m*-Ph-CH), 120.5 (s, $C_5(CH_3)_5$), 123.6 (s, *m*-CH (Dipp), 127.9 (s, *p*-CH or *m*-CH (^{Dipp}Ter))^{x}, 128.2 (s, *p*-CH (Dipp))^{x}, 129.0 (s, *o*-Ph-CH), 131.1 (s, *p*-CH or *m*-CH (^{Dipp}Ter)), 140.6 (s, *i-C* (Dipp)), 146.3 (d, $J_{(C\text{-}P)}$ = 44.4 Hz, *o-C* (^{Dipp}Ter), 147.6 (s, *o-C* (Dipp)), 149.6 (s, *i-C*-P (^{Dipp}Ter)), 153.8 (s, *i-C* (Ph)) ppm. **^{31}P{^{1}H} NMR** (162.0 MHz, C_6D_6, 297 K): δ = −49.7 (s) ppm.

Figure S62: ^{1}H NMR spectrum of isolated **1As·PMe$_3$** (400.1 MHz, C_6D_6, 297 K); 0.88 ppm: *n*-pentane.

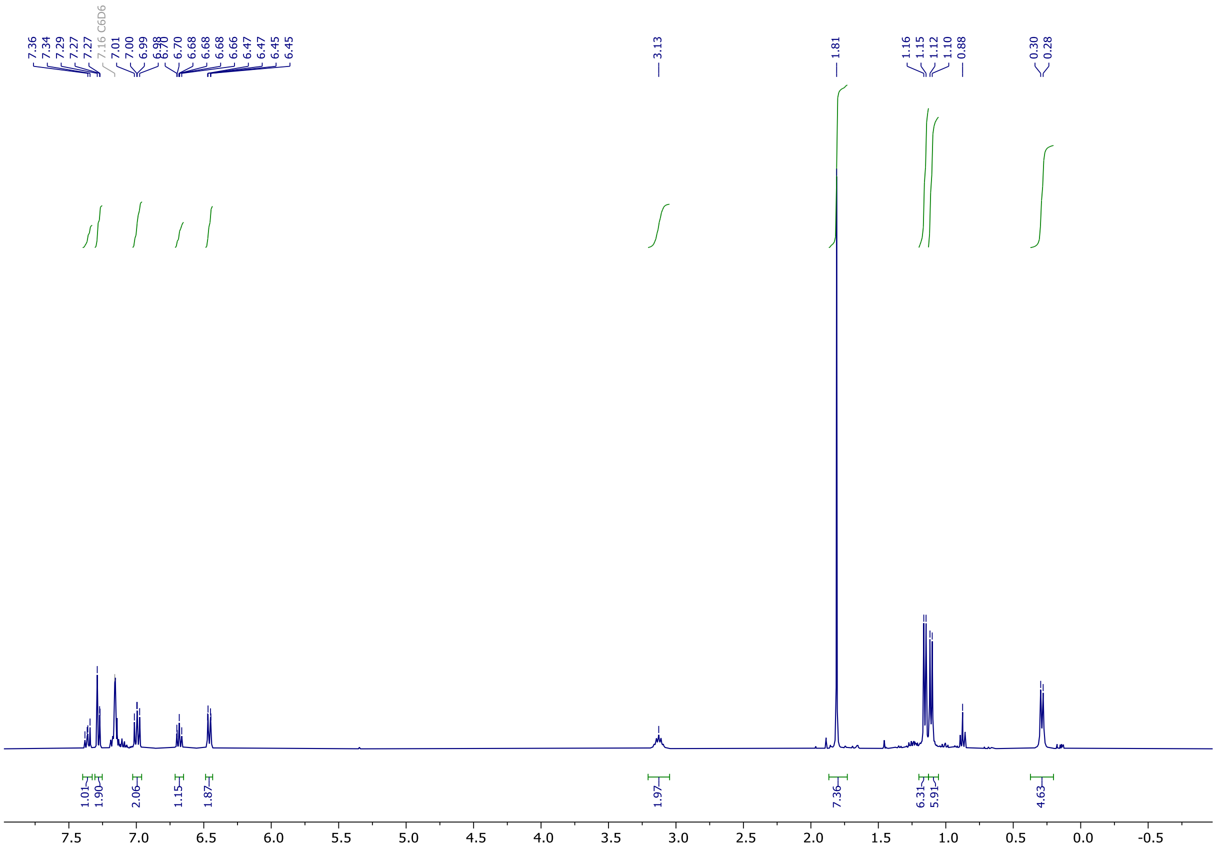

^{x} Superimposed with C_6D_6 signal, assigned with $^{1}H/^{13}C$ HSQC NMR, DEPT-90 and DEPT-135.

Figure S63: $^{13}C\{^{1}H\}$ NMR spectrum of isolated **1As·PMe₃** (100.6 MHz, C_6D_6, 297 K).

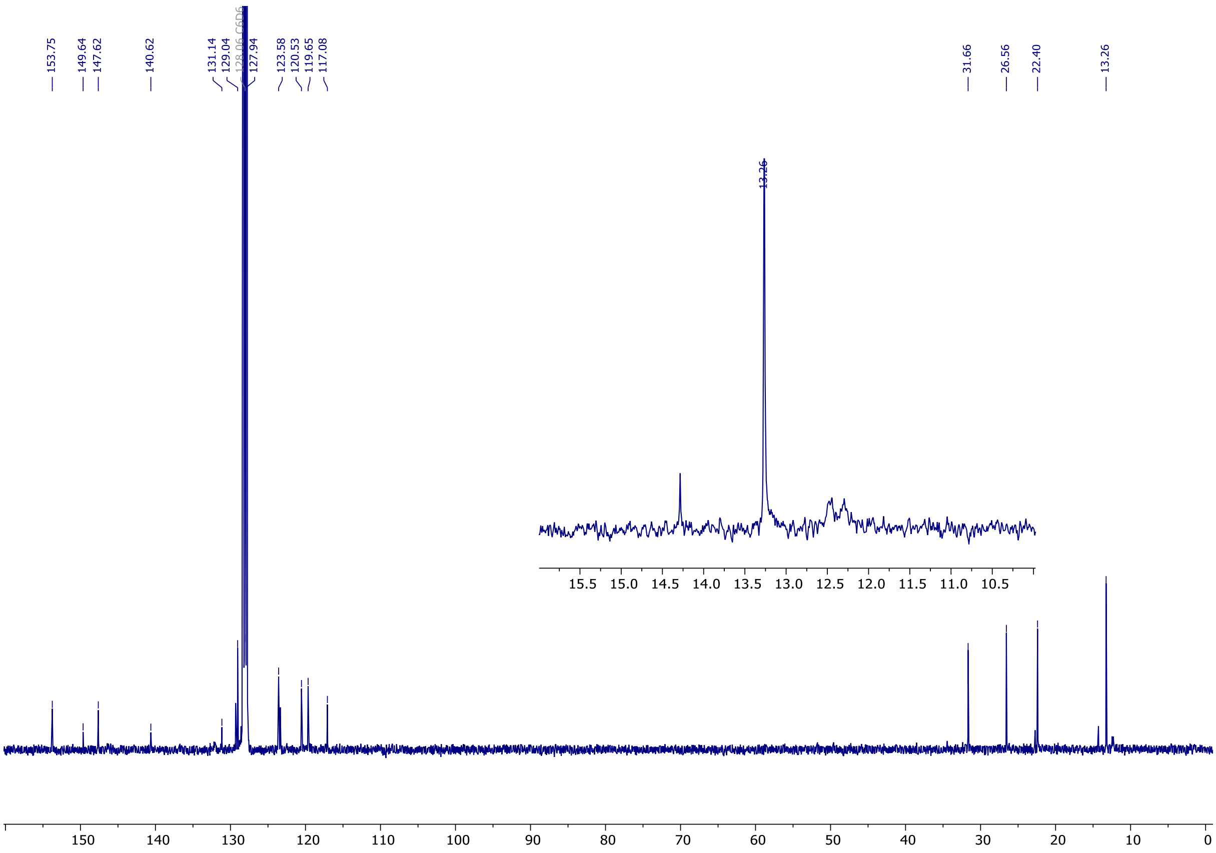

Figure S64: $^{31}P\{^{1}H\}$ NMR spectrum of isolated **1As·PMe₃** (162.0 MHz, C_6D_6, 297 K).

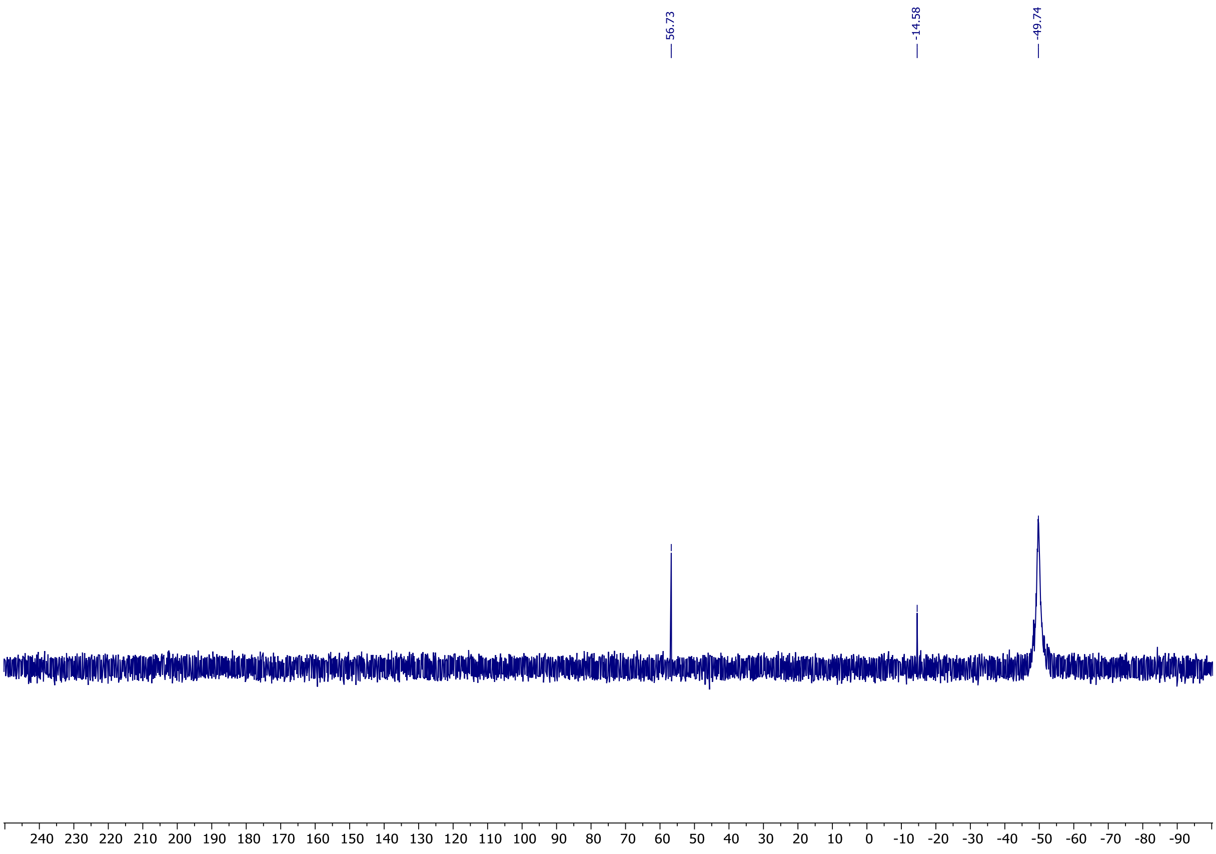

4.6 Compound 1P_F by reaction of ^{Dipp}TerPAlCp* with 1-azido-4-fluorobenzene

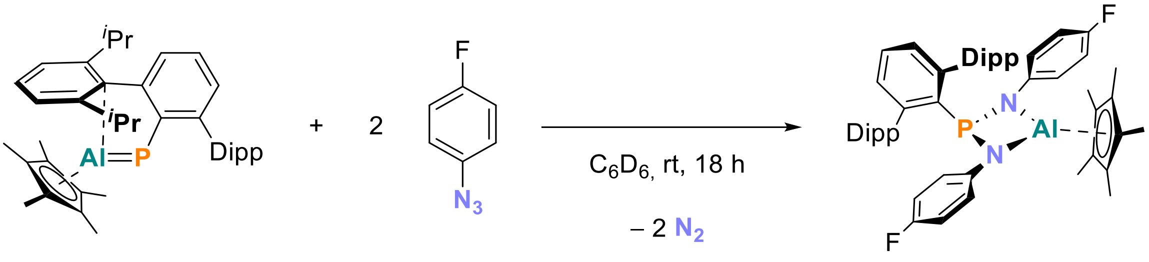

1P_F (33%)

^{Dipp}TerPAlCp* (20.0 mg, 33.9 µmol, 1.00 eq.) was dissolved in C_6D_6 (0.4 mL) in a *J.-Young* NMR tube and 1-azido-4-fluorobenzene (11.1 mg, 81.0 µmol, 2.40 eq.) was added at ambient temperature. Upon addition, the colour of the reaction mixture instantly changed from deep purple to dark orange. The sample was placed on a laboratory shaking plate at ambient temperature for 18 h. After 18 h, full conversion of **^{Dipp}TerPAlCp*** to **1P_F** was confirmed via $^{31}P\{^1H\}$ NMR spectroscopy. The reaction mixture was cooled to –78 °C (N_2(l)/*i*-PrOH cooling bath) and the solvent was removed via sublimation *in vacuo* (~1×10^{-3} mbar) (lyophilisation). In a glovebox, the resulting orange powder was transferred to a pipette equipped with a glass microfiber paper and washed with cold (–30 °C) *n*-pentane (2 × 0.2 mL). The remaining pale-yellow solid was re-dissolved in benzene (0.5 mL), transferred into a *J.-Young* NMR tube, and lyophilised again to remove remaining traces of *n*-pentane and obtain the analytically pure compound **1P_F**.

Yield of **1P_F:** 9.0 mg (11.1 µmol, 33%) of a pale-yellow solid.

Despite several crystallization attempts (C_6H_6, *n*-pentane, HMDSO / silicon grease diffusion or evaporation methods at ambient temperature or −30 °C), no suitable crystals of compound **1P_F** for single crystal X-ray diffraction could be obtained.

CHN (+V_2O_5) calc. (found) in %: C 77.20 (62.18), H 7.48 (6.47), N 3.46 (3.51)[xi]. **^{1}H NMR** (500.1 MHz, C_6D_6, 298 K): δ = 0.94 (d, $^3J_{(H-H)}$ = 6.8 Hz, 12 H, (CH(C*H*$_3$)(CH$_3$)), 0.98 (d, $^3J_{(H-H)}$ = 6.8 Hz, 12 H, (CH(CH$_3$)(C*H*$_3$)), 1.49 (s, 15 H, C_5(C*H*$_3$)$_5$), 2.88 (ψ-hept, $^3J_{(H-H)}$ = 6.8 Hz, 4 H, (C*H*(CH$_3$)(CH$_3$)), 6.62 – 6.65 (m, 4 H, *o-H* (FPh)), 6.70 – 6.74 (m, 4 H, *m-H* (FPh)), 7.03 – 7.04 (m, 3 H, *p-* and *m-H* (^{Dipp}Ter)), 7.14 – 7.15 (m, 4 H, *m-H* (Dipp))[xii], 7.30 (t, $^3J_{(H-H)}$ = 7.6 Hz, 2 H, *p-H* (Dipp) ppm. **^{13}C{^{1}H} NMR** (125.7 MHz, C_6D_6, 298 K): δ = 11.0 (s, C_5(CH$_3$)$_5$), 22.8 (s, (CH(*C*H$_3$)(CH$_3$))$_2$), 26.0 (s, (CH(CH$_3$)(*C*H$_3$))$_2$)), 31.3 (s, (*C*H(CH$_3$)(CH$_3$)), 114.7 (d, $^2J_{(C-F)}$ = 21.6 Hz, *m*-CH (FPh)), 115.3 (s, *C*$_5$(CH$_3$)$_5$)), 122.9 (s, *m*-CH (Dipp)), 126.3 (dd, $^3J_{(C-P)}$ = 13.3 Hz, $^3J_{(C-F)}$ = 7.3 Hz, *o*-CH (FPh)), 127.5 (s, *p-* or *m*-CH (^{Dipp}Ter)), 128.2 (s, *p*-CH (Dipp))[xiii], 132.4 (s, *p-* or *m*-CH (^{Dipp}Ter)), 140.7 (d, $J_{(C-P)}$ = 5.1 Hz, *i*-CH (Dipp)), 145.3 (dd, $^2J_{(C-P)}$ = 17.3 Hz, $^4J_{(C-F)}$ = 2.4 Hz, *i-C* (FPh)), 146.08 (d, $^1J_{(C-P)}$ = 89.9 Hz, *i-C* (^{Dipp}Ter),146.3 (d, $^1J_{(C-P)}$ = 22.4 Hz, *o-C* (^{Dipp}Ter), 147.2 (d, $J_{(C-P)}$ = 1.2 Hz, *o-C* (Dipp)), 158.2 (dd, $^1J_{(C-F)}$ = 238.6 Hz, $J_{(C-P)}$ = 2.6 Hz, *p-C* (FPh)). **^{19}F NMR** (470.5 MHz, C_6D_6, 298 K): δ = –124.06 – –124.14 (m) ppm. **^{31}P{^{1}H} NMR** (202.4 MHz, C_6D_6, 298 K): δ = 86.7 (s) ppm. **IR** (ATR, 32 scans, cm^{-1}): $\tilde{\nu}$ = 3364 (w), 3057 (w), 2960 (m), 2927 (w), 2867 (w), 1608 (w), 1593 (vw), 1575 (w), 1562 (w), 1505 (vs), 1457 (m), 1445 (m), 1383 (w), 1360 (m), 1325 (w), 1280 (m), 1261 (m), 1208 (s), 1179 (m), 1154 (m), 1097 (m), 1082 (m), 1055 (m), 880 (m), 820 (vs), 808 (s), 794 (s), 759 (s), 750 (s), 631 (m), 585 (s), 507 (s), 486 (s), 449 (s), 427 (s), 410 (s). **LIFDI-MS** (toluene): m/z calc: 808.4278 (100.0%) [$C_{52}H_{60}AlF_2N_2P$]; m/z found: 605.2879 [M−AlCp*−*i*-Pr+2H]$^+$.

[xi] Deviations probably due to incomplete combustion.
[xii] Superimposed with C_6D_6 signal, assigned with ^{1}H/^{1}H COSY NMR ^{1}H/^{13}C HSQC NMR.
[xiii] Superimposed with C_6D_6 signal, assigned with ^{1}H/^{13}C HSQC NMR.

Figure S65: ^{1}H NMR spectrum of **1P_F** (500.1 MHz, C_6D_6, 298 K).

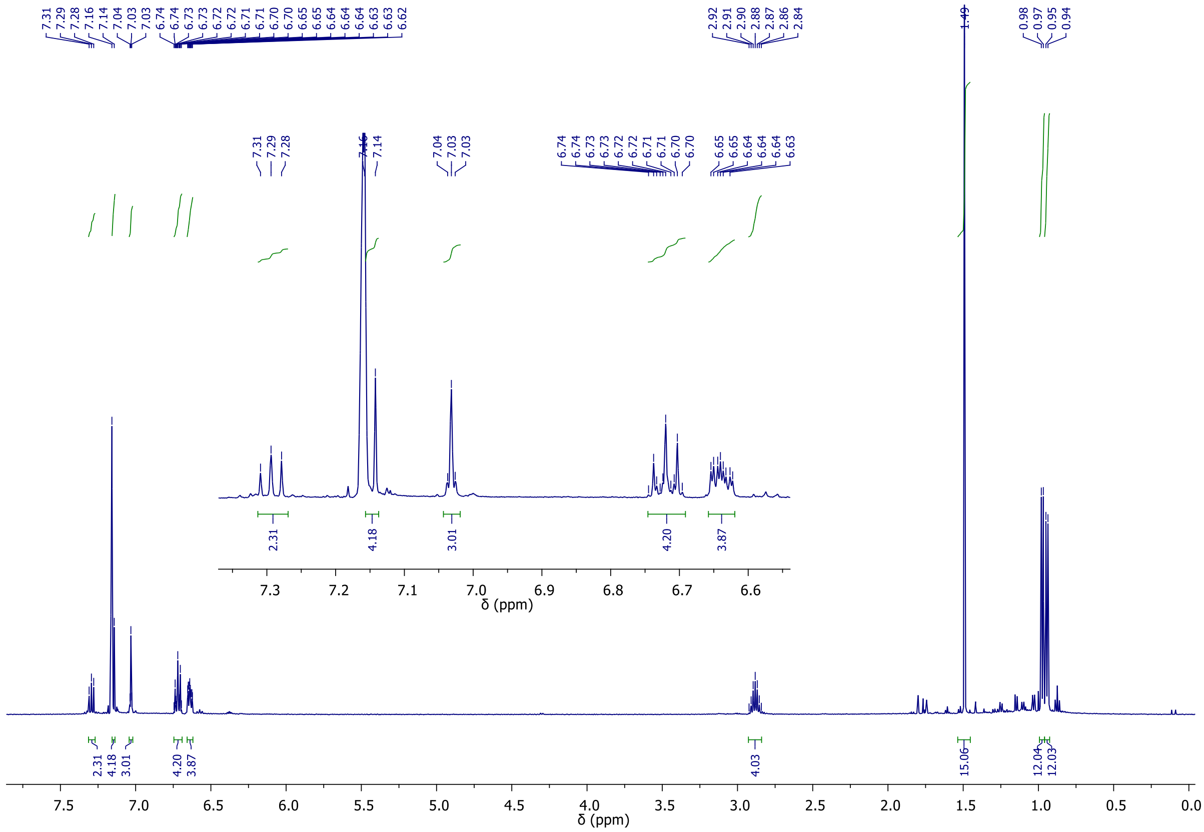

Figure S66: ^{13}C{^{1}H} NMR spectrum of **1P_F** (125.7 MHz, C_6D_6, 298 K).

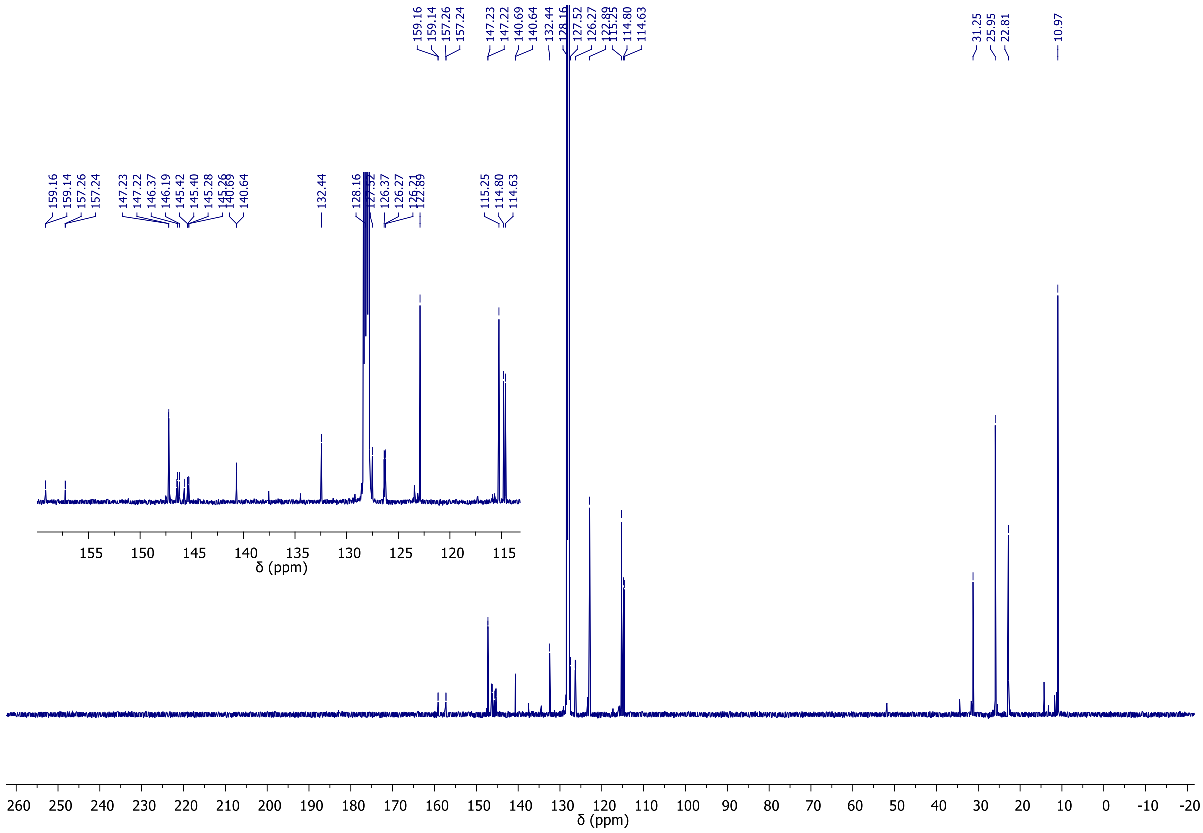

Figure S67: ^{19}F NMR spectrum of **1P_F** (470.5 MHz, C_6D_6, 298 K); unknown side product −125.0 ppm to −125.1 ppm.

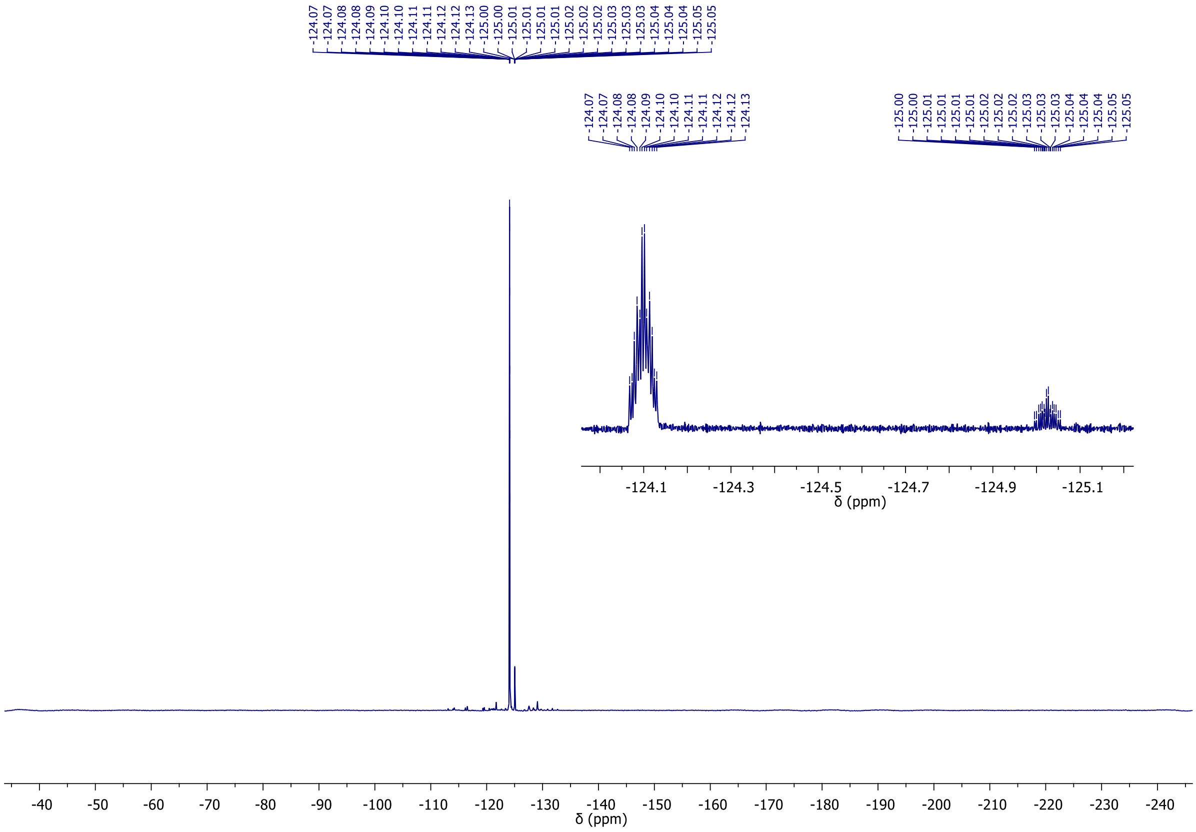

Figure S68: $^{31}P\{^1H\}$ NMR spectrum of **1P_F** (202.4 MHz, C_6D_6, 298 K).

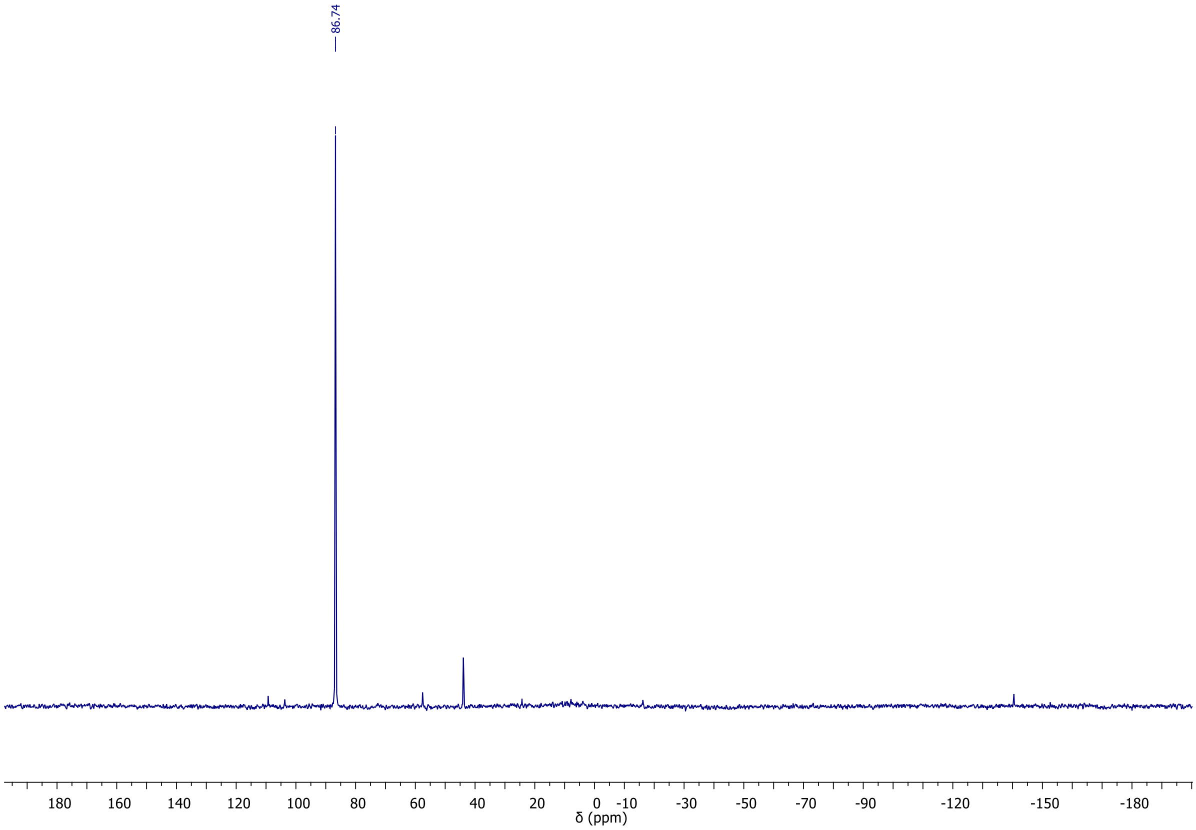

Figure S69: IR-spectrum of **1P_F** (ATR, 32 scans, cm^{-1}, powder; mode at 3364 cm^{-1} might arise from partial hydrolysis during measurement).

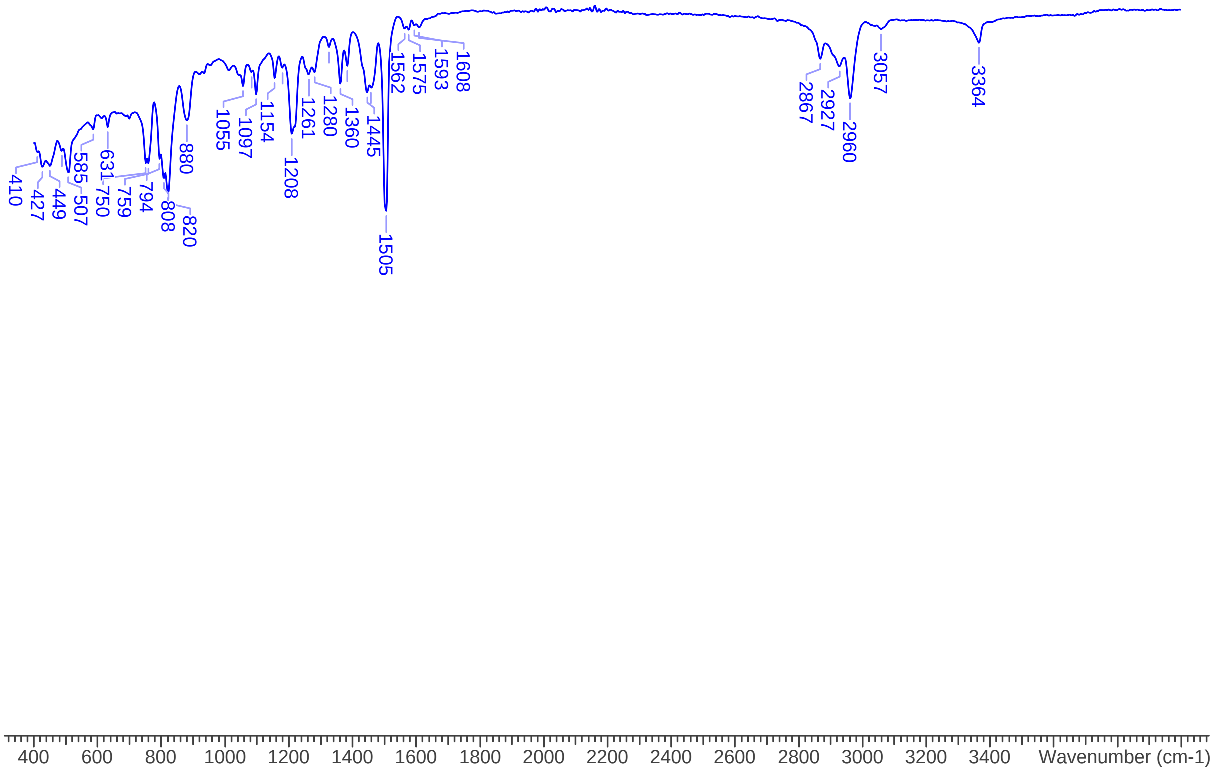

Figure S70: LIFDI mass spectrum of Fehler! Verweisquelle konnte nicht gefunden werden.**P** (toluene).

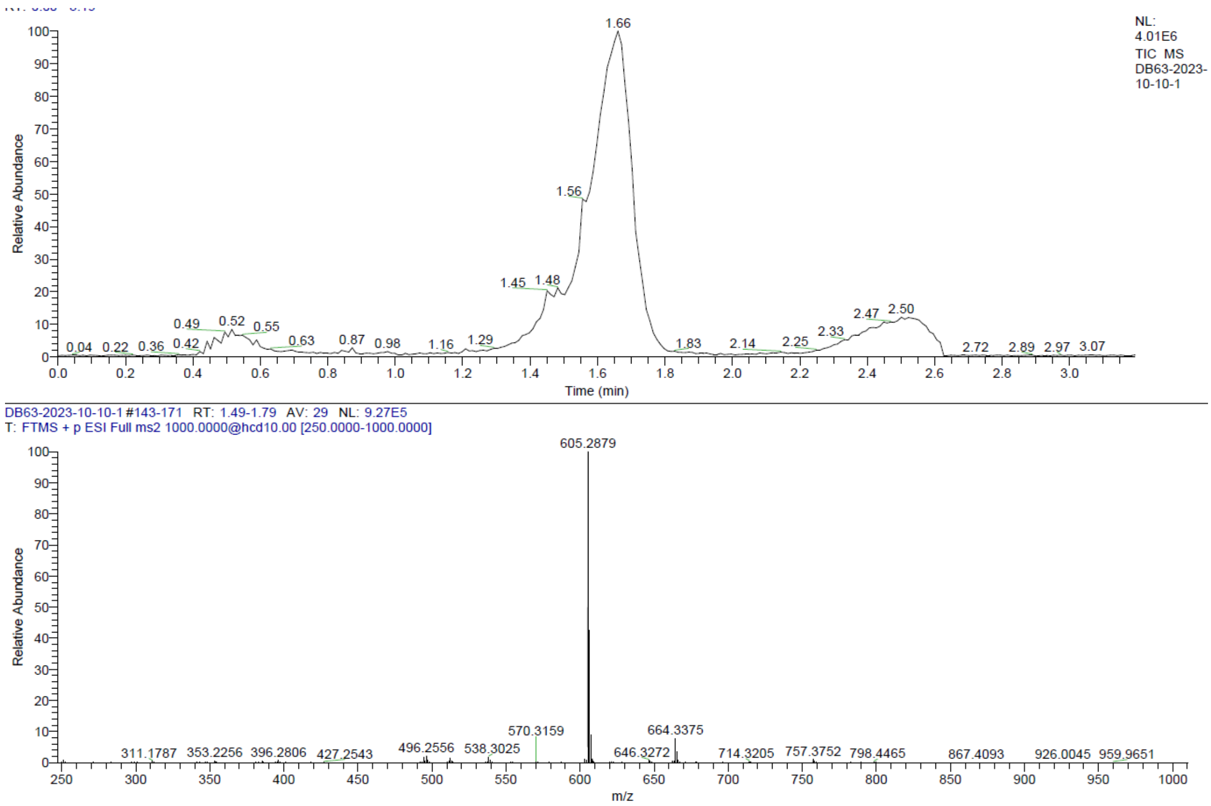

4.7 Compound 2P by reaction of ^{Dipp}TerPAlCp* with 2-azido-1,3-dibromo-5-methylbenzene

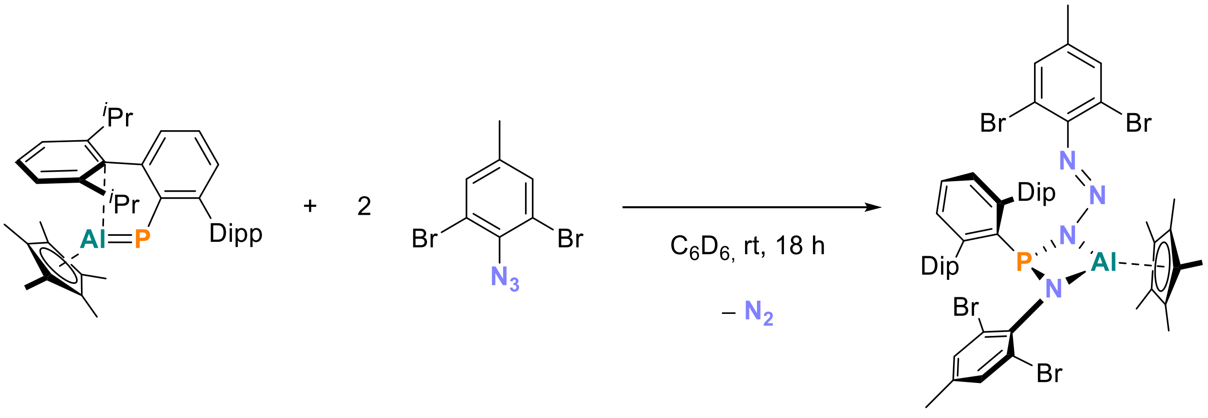

2P (62%)

^{Dipp}TerPAlCp* (20.0 mg, 33.9 µmol, 1.00 eq.) was dissolved in C_6D_6 (0.4 mL) in a *J.-Young* NMR tube and 2-azido-1,3-dibromo-5-methylbenzene (19.6 mg, 67.5 µmol, 2.00 eq.) was added at ambient temperature. Upon addition, the colour of the reaction mixture instantly changed from deep purple to orange. After 30 min, full conversion of **^{Dipp}TerPAlCp*** to **2P** was confirmed via $^{31}P\{^1H\}$ NMR spectroscopy. All volatile components were removed *in vacuo* (~1×10^{-3} mbar). In a glovebox, the resulting pale-orange oil was re-dissolved in *n*-pentane (0.4 mL) and filtered using a pipette equipped with a glass microfiber paper. The *n*-pentane solution was placed in a small vial and the solvent was slowly removed by placing the small vial into a closed, bigger vial that contained silicon grease. The solution was stored at ambient temperature for 48 h, resulting in the crystallization of **2P**. The remaining supernatant was removed with a glass pipette and the solid was carefully washed with cold (–30 °C) *n*-pentane (1 × 0.1 mL) and dried by evaporation of the remaining solvent at ambient temperature in the glovebox.

Yield of 2P: 23.4 mg (20.9 µmol, 62%) of a colourless, crystalline solid.

Suitable crystals for single crystal X-ray diffraction were obtained by slow evaporation of a saturated solution of **2P** in *n*-pentane at ambient temperature in a glovebox with silicon grease as solvent absorbent.

Note: Heating of compound **2P** for 6 h at 80 °C in C_6D_6 led to full conversion of **2P** and was confirmed via $^{31}P\{^1H\}$ NMR spectroscopy to two unknown products with $^{31}P\{^1H\}$ NMR shifts of 101.0 and 11.6 ppm with a ratio of 0.65 to 0.35 . Attempts of lyophilisation and washing of the resulting deep red solid, by analogy to reprocessing of **1P** and **1P_F** or crystallization in benzene or *n*-pentane were not successful and led to a red oily compound.

CHN calc. (found) in %: C 56.56 (51.55), H 5.63 (5.01), N 4.89 (3.61).[xiv] **^{1}H NMR** (500.1 MHz, C_6D_6, 298 K): δ = 0.79 (d, $^3J_{(H-H)}$ = 6.7 Hz, 3 H, C(H)(C*H*$_3$)(CH$_3$) (group A[xv])), 0.97 (d, $^3J_{(H-H)}$ = 6.7 Hz, 3 H, C(H)(C*H*$_3$)(CH$_3$) (group B)), 1.25 (d, $^3J_{(H-H)}$ = 6.7 Hz, 3 H, C(H)(C*H*$_3$)(CH$_3$) (group C)), 1.28 (d, $^3J_{(H-H)}$ = 6.7 Hz, 3 H, C(H)(CH$_3$)(C*H*$_3$) (group B)), 1.29 (d, $^3J_{(H-H)}$ = 6.7 Hz, 3 H, C(H)(C*H*$_3$)(CH$_3$) (group D)), 1.33 (d, $^3J_{(H-H)}$ = 6.7 Hz, 3 H, C(H)(CH$_3$)(C*H*$_3$) (group D)), 1.38 (d, $^3J_{(H-H)}$ = 6.7 Hz, 3 H, C(H)(CH$_3$)(C*H*$_3$) (group C)), 1.54 (d, $^3J_{(H-H)}$ = 6.7 Hz, 3 H, C(H)(CH$_3$)(C*H*$_3$) (group A)), 1.70 (s, 3 H, N$_3$-*i*-C-*o*-(CBr)$_2$-*m*-(CH)$_2$-*p*-C-C*H*$_3$ (group E))[xvi], 1.76 (s, 15 H, C$_5$(C*H*$_3$)$_5$), 1.78 (s, 3 H, N-*i*-C-*o*-(CBr)$_2$-*m*-(CH)$_2$-*p*-C-C*H*$_3$ (group F))[xvi], 2.78 (ψ-hept, $^3J_{(H-H)}$ = 6.7 Hz, 1 H, C(*H*)(CH$_3$)(CH$_3$) (group B)), 3.36 (ψ-hept, $^3J_{(H-H)}$ = 6.7 Hz, 1 H, C(*H*)(CH$_3$)(CH$_3$) (group C)), 3.65 (ψ-hept, $^3J_{(H-H)}$ = 6.7 Hz, 1 H, C(*H*)(CH$_3$)(CH$_3$) (group A)), 3.67 (ψ-hept, $^3J_{(H-H)}$ = 6.7 Hz 1 H, C(*H*)(CH$_3$)(CH$_3$) (group D)), 6.84 (dd, $^3J_{(H-H)}$ = 7.7 Hz, $^4J_{(H-H)}$ = 1.0 Hz, 1 H, *m-H* (Dipp) (group H)), 6.88 (s, 1 H, N-*i*-C-*o*-(CBr)$_2$-*m*-(C*H*)$_2$-*p*-C-CH$_3$ (group F1)), 6.89 (m,1 H, *m-* or *p-H* (^{Dipp}Ter) (group G)), 6.92 (dd, $^3J_{(H-H)}$ = 7.7 Hz, $^4J_{(H-H)}$ = 1.7 Hz, 1 H, *m-* or *p-H* (^{Dipp}Ter) (group G), 7.11 (t, $^3J_{(H-H)}$ = 7.7 Hz, 1 H, *p-H* (Dipp) (group H)), 7.14 (m, 1 H, *m-* or *p-H* (^{Dipp}Ter) (group G))[xvii],

[xiv] Deviations likely due to incomplete combustion, repeated measurements did not give better results.

[xv] *Note*: The different substitution on the nitrogen atoms of the four-membered ring in the product lead to a low symmetry (C_1) in solution. As a result, there are 8 resonances for methyl groups (C(H)(C*H*$_3$)(C*H*$_3$)) and four resonances for methanetriyl groups (C(*H*)(CH$_3$)(CH$_3$)) in the aliphatic region of the ^{1}H NMR spectrum for the ^{Dipp}Ter substituent in **2P**. To assign all resonances that belong to one *i*Pr group the labels A, B, C, D were used. The labels E and F were used to assign all resonances that belong to one dibromotolyl group. Additionally, the two *meta* resonances of the dibromotolyl group F were chemically inequivalent, therefore the labels F1 and F2 were used. A similar differentiation appears for the resonances of the ^{Dipp}Ter ligand G, H, I substituent in the aromatic region.

[xvi] Assigned with ^{1}H/^{1}H ROESY NMR spectrum.

[xvii] Appears as dd in ^{1}H{^{31}P} NMR spectrum.

7.16 (s, 2 H, N_3-*i*-C-*o*-$(CBr)_2$-*m*-$(CH)_2$-*p*-C-CH_3 (<u>group E</u>))[xviii], 7.18 (d, $^4J_{(H-H)}$ = 2.0 Hz, 1H, N-*i*-C-*o*-$(CBr)_2$-*m*-$(CH)_2$-*p*-C-CH_3 (<u>group F2</u>)), 7.21 (dd, $^3J_{(H-H)}$ = 7.7 Hz, $^4J_{(H-H)}$ = 1.1 Hz, 1H, *m-H* (Dipp) (<u>group H</u>)), 7.30 – 7.36 (m, 3H, *m-* and *p-H* (Dipp) (<u>group I</u>)) ppm. **^{13}C{^{1}H} NMR** (125.7 MHz, C_6D_6, 298 K): δ = 10.6 (s, $C_5(CH_3)_5$), 19.6 (s, N-*i*-C-*o*-$(CBr)_2$-*m*-$(CH)_2$-*p*-C-CH_3 (<u>group F</u>)), 19.8 (s, N_3-*i*-C-*o*-$(CBr)_2$-*m*-$(CH)_2$-*p*-C-CH_3 (<u>group E</u>)), 23.3 (d, $J_{(C-P)}$ = 3.1 Hz, C(H)(CH_3)(CH_3) (<u>group B</u>) or C(H)(CH_3)(CH_3) (<u>group D</u>)), 23.3 (s C(H)(CH_3)(CH_3) (<u>group B</u>) or C(H)(CH_3)(CH_3) (<u>group D</u>)), 24.0 (s, C(H)(CH_3)(CH_3) (<u>group C</u>), 24.9 (s, C(H)(CH_3)(CH_3) (<u>group A</u>)), 25.8 (s, C(H)(CH_3)(CH_3) (<u>group A</u>)), 26.4 (s, C(H)(CH_3)(CH_3) (<u>group B</u>) and C(H)(CH_3)(CH_3) (<u>group D</u>)), 28.0 (s, C(H)(CH_3)(CH_3) (<u>group C</u>)), 30.2 (s, *C*(H)(CH_3)(CH_3) (<u>group B</u>)), 30.6 (s, *C*(H)(CH_3)(CH_3) (<u>group C</u>)), 30.8 (s, *C*(H)(CH_3)(CH_3) (<u>group A</u>)), 31.6 (d, $J_{(C-P)}$ = 6.0 Hz, *C*(H)(CH_3)(CH_3) (<u>group D</u>)), 115.3 (s, $C_5(CH_3)_5$), 121.1 (s, *m*-CH (Dipp) (<u>group H</u>)), 122.3 (s, *m*- or *p*-CH (Dipp) (<u>group I</u>), 122.9 (s, *m*-CH (Dipp) (<u>group H</u>)), 124.6 (d, $J_{(C-P)}$ = 4.7 Hz, N-*i*-C-*o*-$(CBr)_2$-*m*-$(CH)_2$-*p*-C-CH_3 (<u>group F2</u>)), 124.85 (s, *m*- or *p*-CH (Dipp) (<u>group I</u>)), 125.6 (d, $J_{(C-P)}$ = 7.4 Hz, N-*i*-C-*o*-$(CBr)_2$-*m*-$(CH)_2$-*p*-C-CH_3 (<u>group F1</u>)), 127.0 (s, *p*- or *m*-CH (^{Dipp}Ter) (<u>group G</u>)), 127.6 (s, *p*-CH (Dipp) (<u>group H</u>))[xix], 128.4 (s, *m*-CH (Dipp) (<u>group I</u>))[xix], 132.1 (d, $J_{(C-P)}$ = 7.3 Hz, *p*-CH (^{Dipp}Ter) (<u>group G</u>)), 132.8 (s, N-*i*-C-*o*-$(CBr)_2$-*m*-$(CH)_2$-*p*-C-CH_3 (<u>group F2</u>)), 133.6 (s, N-*i*-C-*o*-$(CBr)_2$-*m*-$(CH)_2$-*p*-C-CH_3 (<u>group F1</u>)), 134.1 (bs, N_3-*i*-C-*o*-$(CBr)_2$-*m*-$(CH)_2$-*p*-C-CH_3 (<u>group E</u>))[xx], 135.0 (d, $J_{(C-P)}$ = 1.9 Hz, N_3-*i*-C-*o*-$(CBr)_2$-*m*-$(CH)_2$-*p*-*C*-CH_3 (<u>group F</u>), 135.4 (s, *m*-CH (^{Dipp}Ter) (<u>group G</u>)), 136.9 (s, N_3-*i*-C-*o*-$(CBr)_2$-*m*-$(CH)_2$-*p*-*C*-CH_3 (<u>group E</u>)), 141.0 (d, $^3J_{(C-P)}$ = 30.1 Hz, *i-C* (Dipp) (<u>group H</u>)), 141.3 (d, $^1J_{(C-P)}$ = 69.7 Hz, *i-C* (^{Dipp}Ter) (<u>group G</u>)). 142.7 (d, $J_{(C-P)}$ = 3.1 Hz, *o-C* (^{Dipp}Ter) (<u>group G</u>)). 143.4 (s, *i-C* (Dipp) (<u>group I</u>)), 145.0 (d, $J_{(C-P)}$ = 18.8 Hz, N-*i*-*C*-*o*-$(CBr)_2$-*m*-$(H)_2$-*p*-C-CH_3 (<u>group F</u>)), 145.3 (s, N_3-*i*-*C*-*o*-$(CBr)_2$-*m*-$(CH)_2$-*p*-C-CH_3 (<u>group E</u>)), 146.8 (d, $J_{(C-P)}$ = 2.4 Hz, *o-C* (Dipp) (<u>group H</u>)), 147.2 (s, *o-C* (Dipp) (<u>group I</u>)), 148.8 (d, $J_{(C-P)}$ = 4.4 Hz, *o-C* (Dipp) (<u>group H</u>)), 149.0 (s, *o-C* (Dipp) (<u>group I</u>)), 150.9 (d, $^1J_{C-P)}$ = 57.8 Hz, *o-C* (^{Dipp}Ter) (<u>group G</u>)) ppm. **^{31}P{^{1}H} NMR** (202.5 MHz, C_6D_6, 298 K): δ = 77.9 (s) ppm.

[xviii] Superimposed with C_6D_6 resonance, assigned with ^{1}H/^{1}H COSY NMR spectrum.

[xix] Superimposed with solvent signal assigned with DEPT-90 ^{13}C NMR, DEPT-135 ^{13}C NMR and ^{1}H/^{13}C HSQC NMR.

[xx] Assigned with ^{1}H/^{13}C HSQC NMR spectrum.

IR (ATR, 32 scans, cm^{-1}): $\tilde{\nu}$ = 3354 (w), 3304 (w), 3057 (w), 2960 (m), 2923 (m), 2865 (m), 2807 (w), 1616 (w), 1589 (w), 1575 (w), 1542 (w), 1455 (vs), 1381 (m), 1360 (m), 1327 (w), 1290 (w), 1259 (m), 1214 (w), 1202 (w), 1177 (m), 1113 (m), 1103 (m), 1078 (m), 1057 (m), 1039 (m), 1000 (m), 954 (m), 934 (m), 905 (m), 851 (vs), 822 (s), 806 (s), 794 (s), 750 (vs), 734 (vs), 705 (s), 690 (s), 658 (s), 643 (s), 625 (s), 583 (s), 561 (vs), 536 (vs), 499 (s), 491 (s), 464 (vs), 456 (vs), 435 (vs), 427 (vs). **LIFDI-MS** (toluene): m/z calc 1144.1230 [$C_{54}H_{62}AlBr_4N_4P$]; m/z found: 1101.0647 [M−*i*-Pr]$^+$, 912.9532 [M−*i*-Pr−2×Me−AlCp*+4H]$^+$, 718.8246 [M−Dipp−2×*i*-Pr−Me−AlCp*+2H]$^+$.

Figure S71: ^{1}H NMR spectrum of **2P** (500.1 MHz, C_6D_6, 298 K).

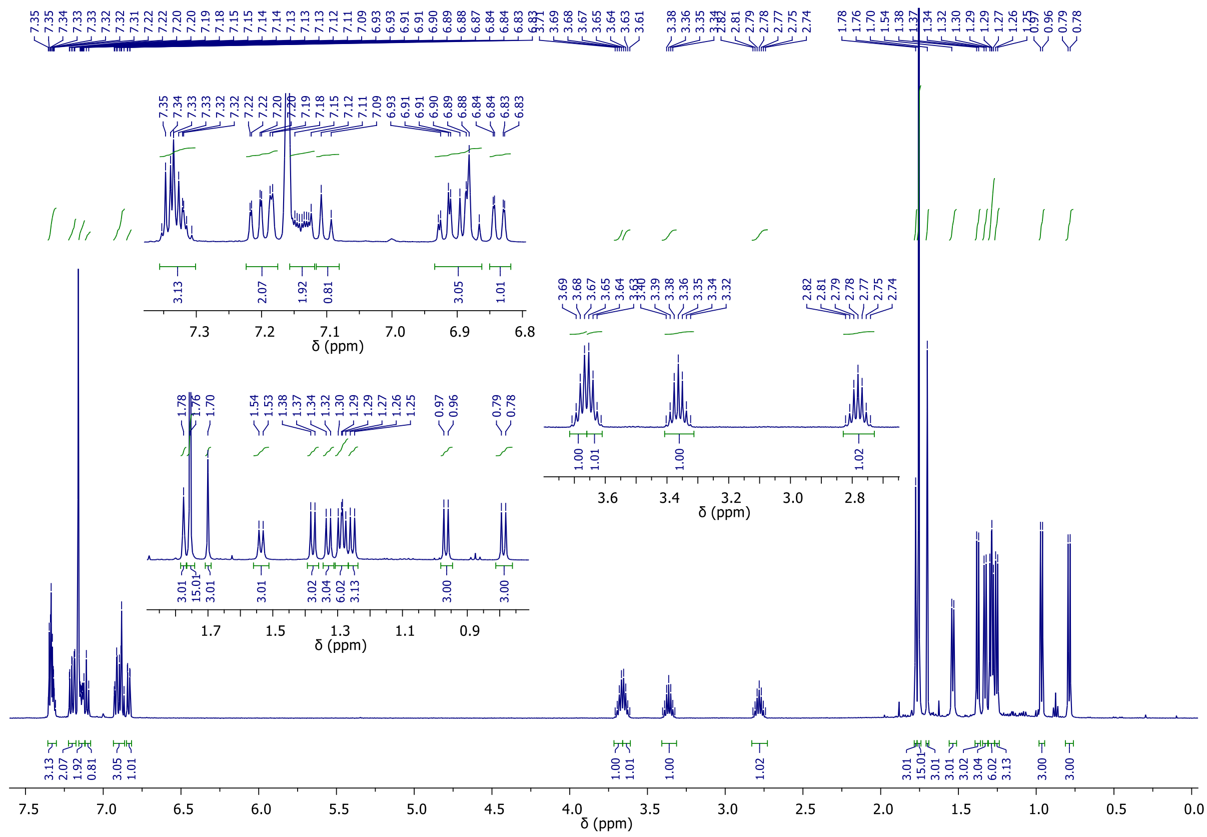

Figure S72: $^{13}C\{^{1}H\}$ NMR spectrum of **2P** (125.7 MHz, C_6D_6, 298 K).

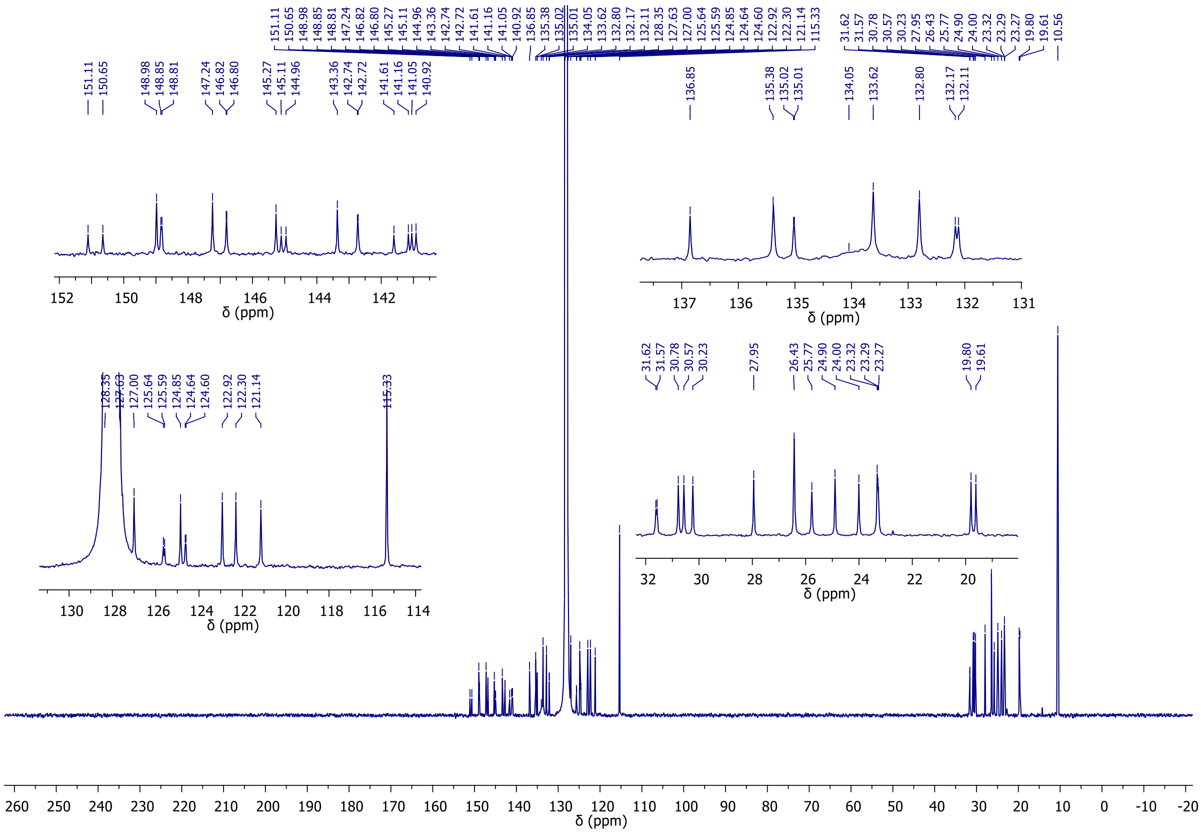

Figure S73: $^{31}P\{^{1}H\}$ NMR spectrum of **2P** (202.5 MHz, C_6D_6, 298 K).

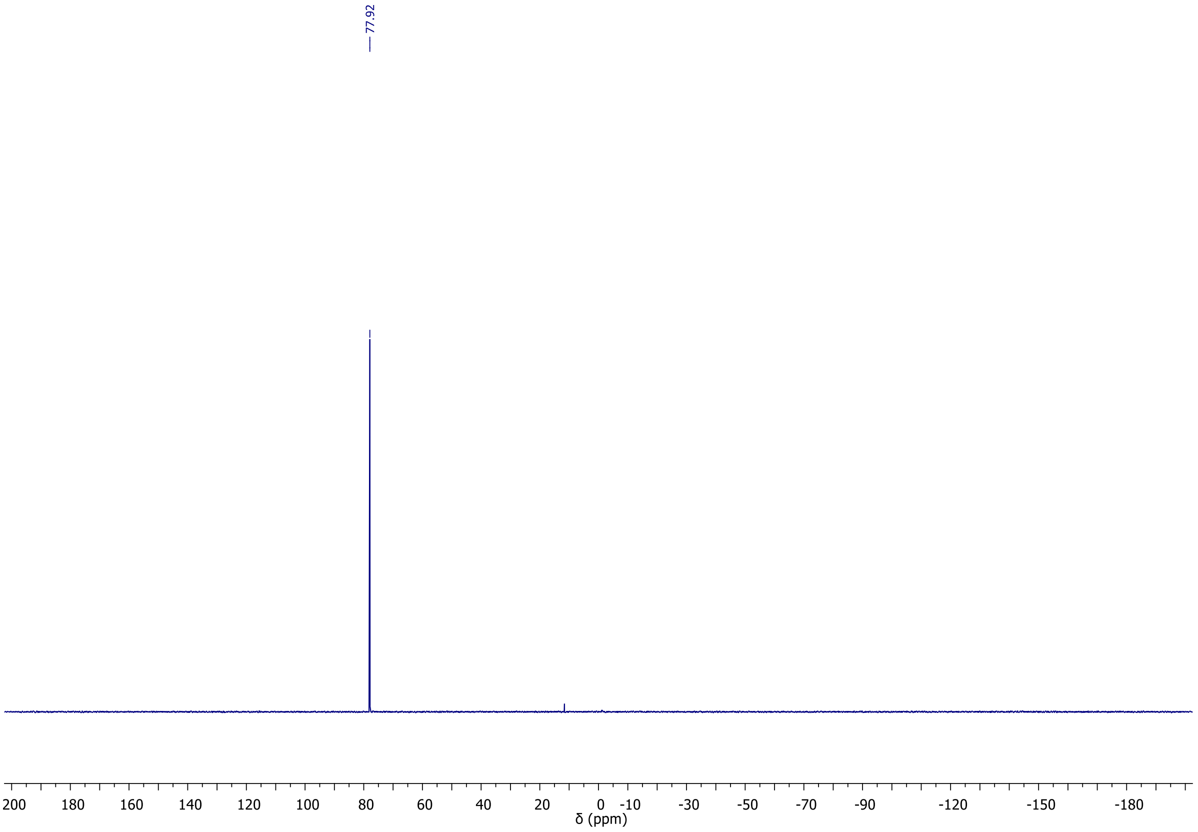

Figure S74: IR spectrum of **2P** (ATR, 32 scans, cm^{-1}, powder; ; modes at >3300 cm^{-1} might arise from partial hydrolysis during measurement).

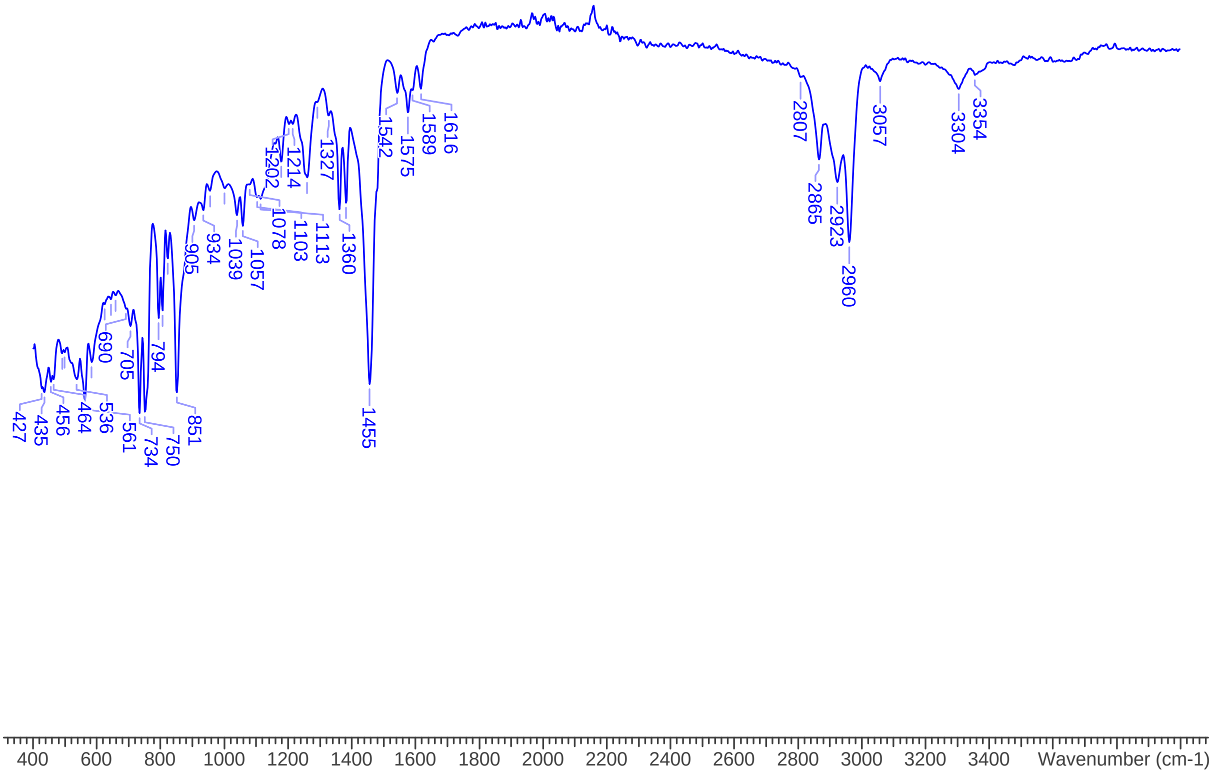

Figure S75: LIFDI mass spectrum of **2P** (toluene).

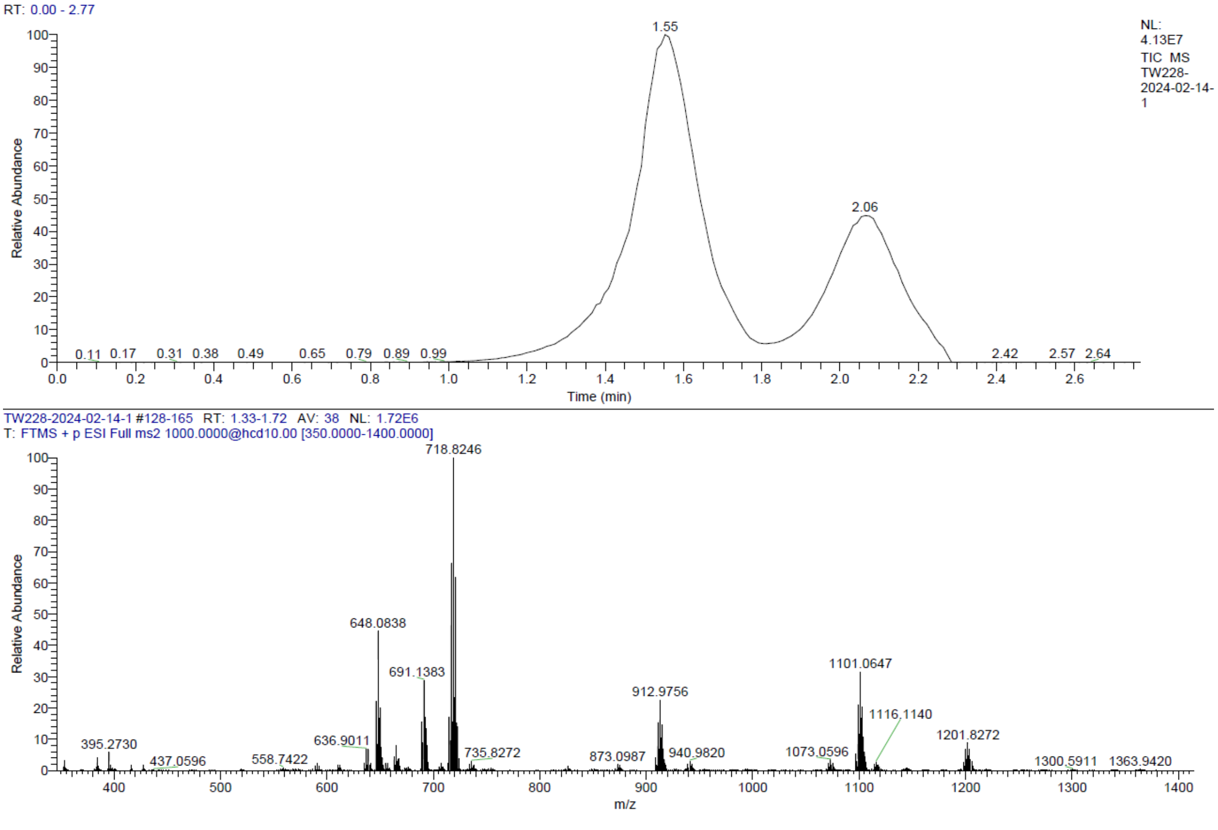

Figure S76: $^{31}P\{^1H\}$ NMR spectrum of the reaction mixture after heating **2P** at 80 °C for 6 h in C_6D_6 (162.0 MHz, C_6D_6, 298 K).

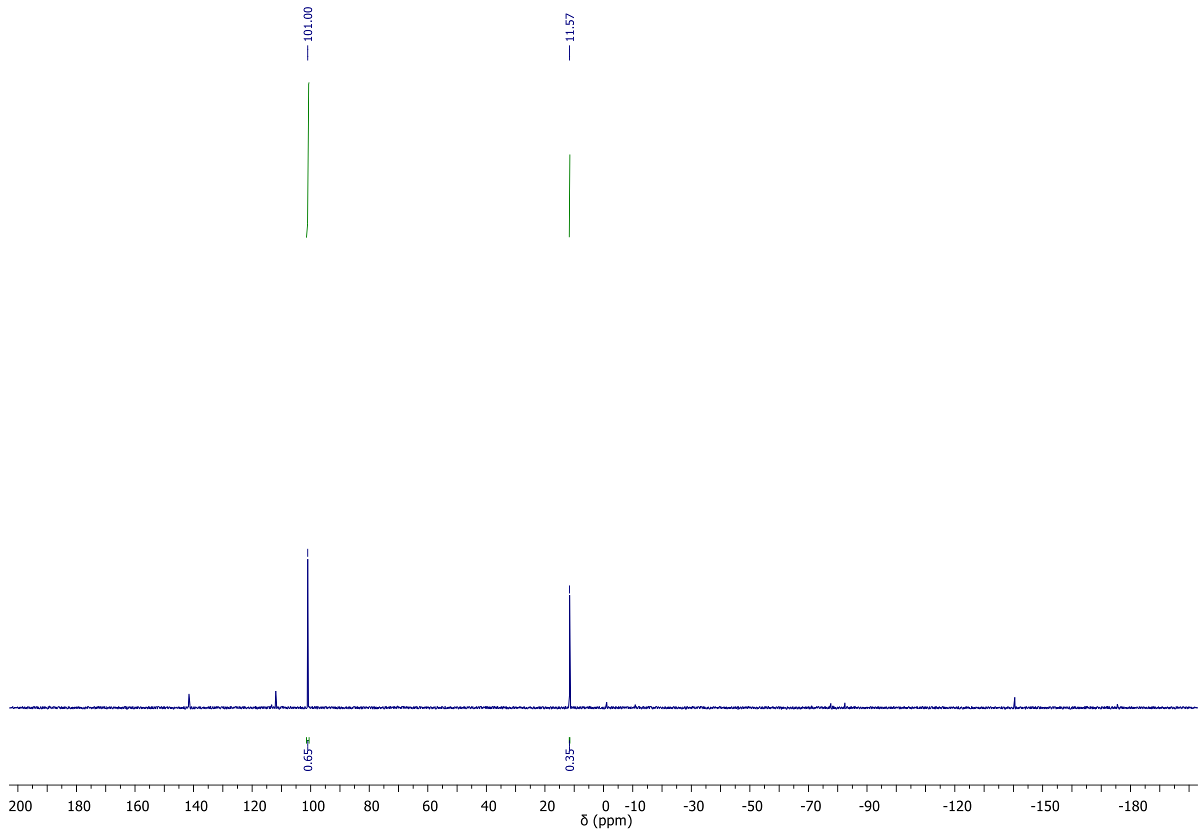

4.8 Compound 2As by reaction of ^{Dipp}TerAsAlCp* with 2-azido-1,3-dibromo-5-methylbenzene

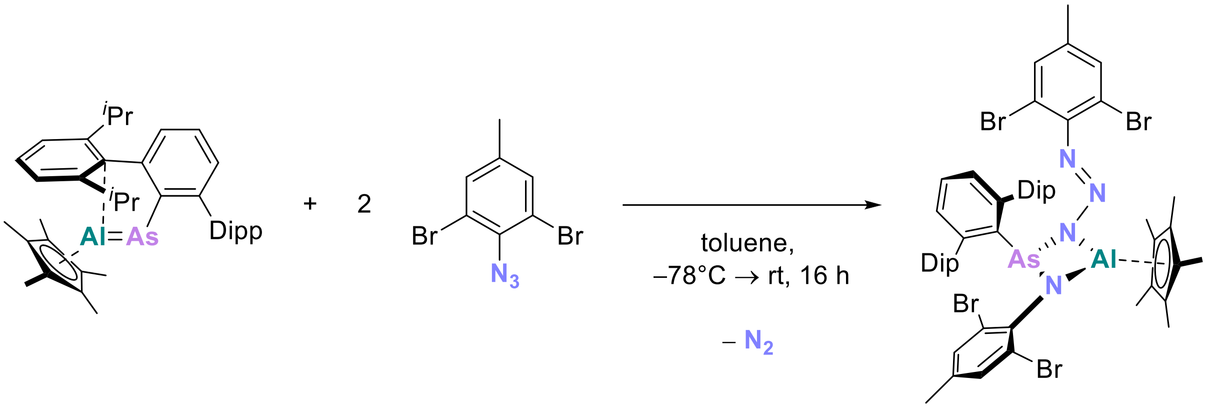

^{Dipp}TerAsAlCp* (50.0 mg, 78.8 μmol) was dissolved in toluene (2.5 mL) at 45 °C (water bath). The solution was cooled to −78 °C (CO_2/EtOH cooling bath) and a solution of 2-azido-1,3-dibromo-5-methylbenzene (46.0 mg, 160.0 μmol) in toluene (2.5 mL) was added over a period of 10 min in the dark, whereby a colour change from blue to yellow occurred. The reaction mixture was allowed to slowly warm to ambient temperature by keeping it in the cooling bath overnight. The solvent was removed *in vacuo* and the residual solid was dried for 30 min at ambient temperature at 1×10^{-3} mbar. The residue was then extracted with *n*-hexane (10 mL). The insoluble material was removed by filtration through a plug of celite. The filtrate was concentrated rapidly to ~0.5 mL *in vacuo* and was placed in a bowl of warm water (approx. 45 °C) in a fridge (5 °C) to get colourless crystals overnight. The supernatant was removed by syringe and the crystals were washed with cold (−78 °C) *n*-hexane (1 ml). Supernatant and washing solution were combined to get a second fraction of product analogous to the procedure described above. The isolated crystalline fractions were dried for 1 h at ambient temperature at 1×10^{-3} mbar.

Yield of **2As:** 46.0 mg (39.0 μmol, 49%) of a colourless, crystalline solid.

Single crystals suitable for SCXRD were grown as described in the synthesis procedure.

CHN calc. (found) in %: C 54.57 (53.18), H 5.26 (5.43), N 4.71 (2.89); deviations probably due to incomplete combustion, repeated measurements with and without adding V_2O_5 as additional oxidation reagent did not result in better agreement. **^{1}H NMR** (300.2 MHz, C_6D_6,): δ = 0.84 (d, $^3J_{(H-H)}$ = 6.7 Hz, 3 H, C(H)(CH_3)(CH_3) (<u>group A</u>)[xxi]), 0.94 (d, $^3J_{(H-H)}$ = 6.7 Hz, 3 H, C(H)(CH_3)(CH_3) (<u>group B</u>)), 1.16 (d, $^3J_{(H-H)}$ = 6.9 Hz, 3 H, C(H)(CH_3)(CH_3) (<u>group B</u>)), 1.23 (d, $^3J_{(H-H)}$ = 6.7 Hz, 3 H, C(H)(CH_3)(CH_3) (<u>group C</u>)), 1.29-1.38 (superimposed signals, 9 H, C(H)(CH_3)(CH_3) (<u>group C</u> (1×), <u>group D</u> (2×))), 1.50 (d, $^3J_{(H-H)}$ = 6.5 Hz, 3 H, C(H)(CH_3)(CH_3) (<u>group A</u>)), 1.70 (m, 3 H, ($C_6H_2Br_2$)-CH_3 (<u>group E</u>)), 1.75 (m, 3 H, ($C_6H_2Br_2$)-CH_3 (<u>group F</u>)), 1.81 (s, 15 H, C_5(CH_3)$_5$), 2.84 (ψ-hept, $J_{(H-H)}$ = 6.7 Hz, 1 H, C(H)(CH_3)(CH_3) (<u>group B</u>)), 3.32 (ψ-hept, $^3J_{(H-H)}$ = 6.5 Hz, 1 H, C(H)(CH_3)(CH_3) (<u>group A</u>)), 3.40 (ψ-hept, $J_{(H-H)}$ = 6.5 Hz, 1 H, C(H)(CH_3)(CH_3) (<u>group D</u>)), 3.61 (ψ-hept, $^3J_{(H-H)}$ = 6.7 Hz, 1 H, C(H)(CH_3)(CH_3) (<u>group C</u>)), 6.85-7.37 (superimposed signals, 9 H, aromatic CH (^{Dipp}Ter)), 6.89 (m, 1 H, CH *(dibromotolyl, <u>group F</u>)*, 7.15 (m, 2 H, CH *(dibromotolyl, <u>group E</u>)*, 7.20 (m, 1 H, CH *(dibromotolyl, <u>group F</u>)*. **^{13}C{^{1}H} NMR** (C_6D_6, 75.5 MHz): δ = 10.7 (s, C_5(CH_3)$_5$), 14.4 (*n*-hexane (impurity)), 19.6 (s, ($C_6H_2Br_2$)-CH_3), 19.8 (s, ($C_6H_2Br_2$)-CH_3), 23.1 (*n*-hexane (impurity)), 23.1 (s, C(H)(CH_3)(CH_3)), 23.4 (s, C(H)(CH_3)(CH_3)), 23.9 (s, C(H)(CH_3)(CH_3)), 25.2 (s, C(H)(CH_3)(CH_3)), 25.4 (s, C(H)(CH_3)(CH_3)), 25.8 (s, C(H)(CH_3)(CH_3)), 26.2 (s, C(H)(CH_3)(CH_3)), 27.9 (s, C(H)(CH_3)(CH_3)), 30.4 (s, C(H)(CH_3)(CH_3)), 30.7 (s superimposed signals, C(H)(CH_3)(CH_3)), 31.7 (s, C(H)(CH_3)(CH_3)), 32.0 (*n*-hexane (impurity)), 177.7 (s, aromatic $C_{quart.}$), 115.2 (s, C_5(CH_3)$_5$), 122.1 (s, aromatic CH), 122.8 (s, aromatic CH), 123.1 (s, aromatic CH), 124.1 (s, aromatic CH), 125.3 (s, aromatic CH), 126.5 (s, aromatic $C_{quart.}$), 127.0 (s, aromatic CH), 132.0 (s, aromatic CH), 132.8 (s, aromatic CH), 133.1 (s, aromatic CH), 133.7 (s, aromatic CH), 134.3 (s, aromatic CH), 135.1 (s, aromatic CH), 136.7 (s, aromatic CH), 144.3 (aromatic $C_{quart.}$), 145.5 (aromatic $C_{quart.}$), 147.0 (aromatic $C_{quart.}$), 147.2 (aromatic

[xxi] *Note*: The different substitution on the nitrogen atoms of the four-membered ring and the hindered rotation of the ^{Dipp}Ter substituent in the product lead to a low symmetry (C_1) in solution. As a result, there are 8 signals for methyl groups (C(H)(CH_3)(CH_3)) and four signals for methanetriyl groups (C(H)Me_2) in the aliphatic region of the ^{1}H NMR spectrum for the ^{Dipp}Ter substituent in **2As**. To assign all signals that belong to one *i*Pr group the labels **<u>A</u>**, **<u>B</u>**, **<u>C</u>**, **<u>D</u>** were used. The labels **<u>E</u>** and **<u>F</u>** were used to assign all signals that belong to one dibromotolyl group. A similar differentiation is expected for the Signals of the ^{Dipp}Ter substituent in the aromatic region. However, as there is a strong overlap between these signals, no assignments could be made here.

$C_{quart.}$), 147.4 (aromatic $C_{quart.}$), 147.6 (aromatic $C_{quart.}$), 148.4 (aromatic $C_{quart.}$), 148.7 (aromatic $C_{quart.}$). Some of the $C_{quart.}$ could not be found due to the low signal to noise ratio. **IR** (ATR, 32 scans, cm^{-1}): $\tilde{\nu}$ = 3053 (w), 2960 (m), 2921 (m), 2863 (m), 1589 (w), 1575 (w), 1556 (w), 1531 (w), 1455 (m), 1437 (s), 1379 (m), 1358 (s), 1261 (m), 1245 (s), 1204 (m), 1171 (s), 1057 (m), 1041 (m), 977 (m), 911 (m), 884 (m), 847 (s), 818 (m), 804 (m), 794 (m), 759 (vs), 742 (s), 734 (s), 728 (s), 717 (s), 641 (s), 614 (m), 600 (s), 561 (vs), 536 (vs), 524 (s), 493 (m), 468 (s), 439 (m), 420 (m), 406 (s). **MS** (CI, pos., isobutene, m/z): 472 [^{Dipp}TerAs]$^+$, 396 [^{Dipp}Ter − H]$^+$.

Figure S77: ^{1}H NMR spectrum **2As** (300.2 MHz, C_6D_6, 298 K).

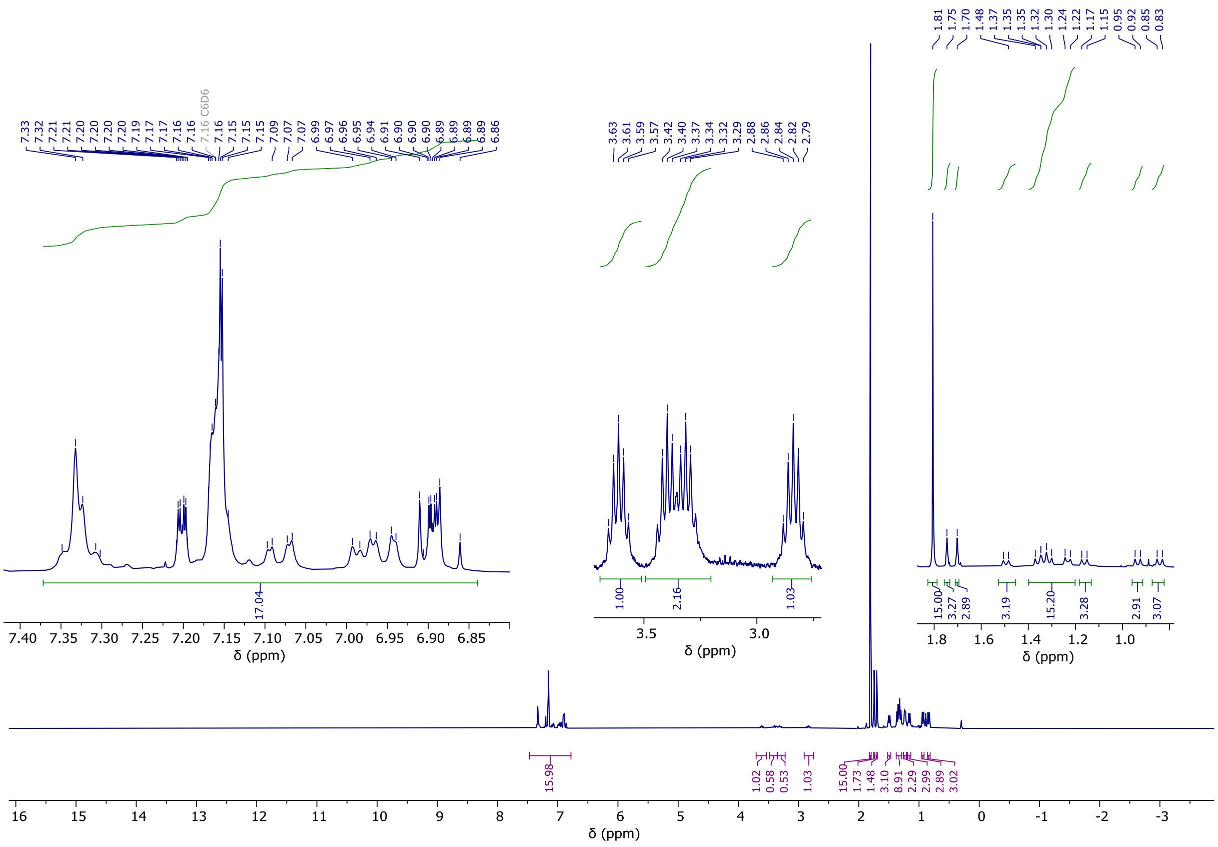

Figure S78: $^{13}C\{^1H\}$ NMR spectrum of **2As** (75.5 MHz, C_6D_6, 298 K).

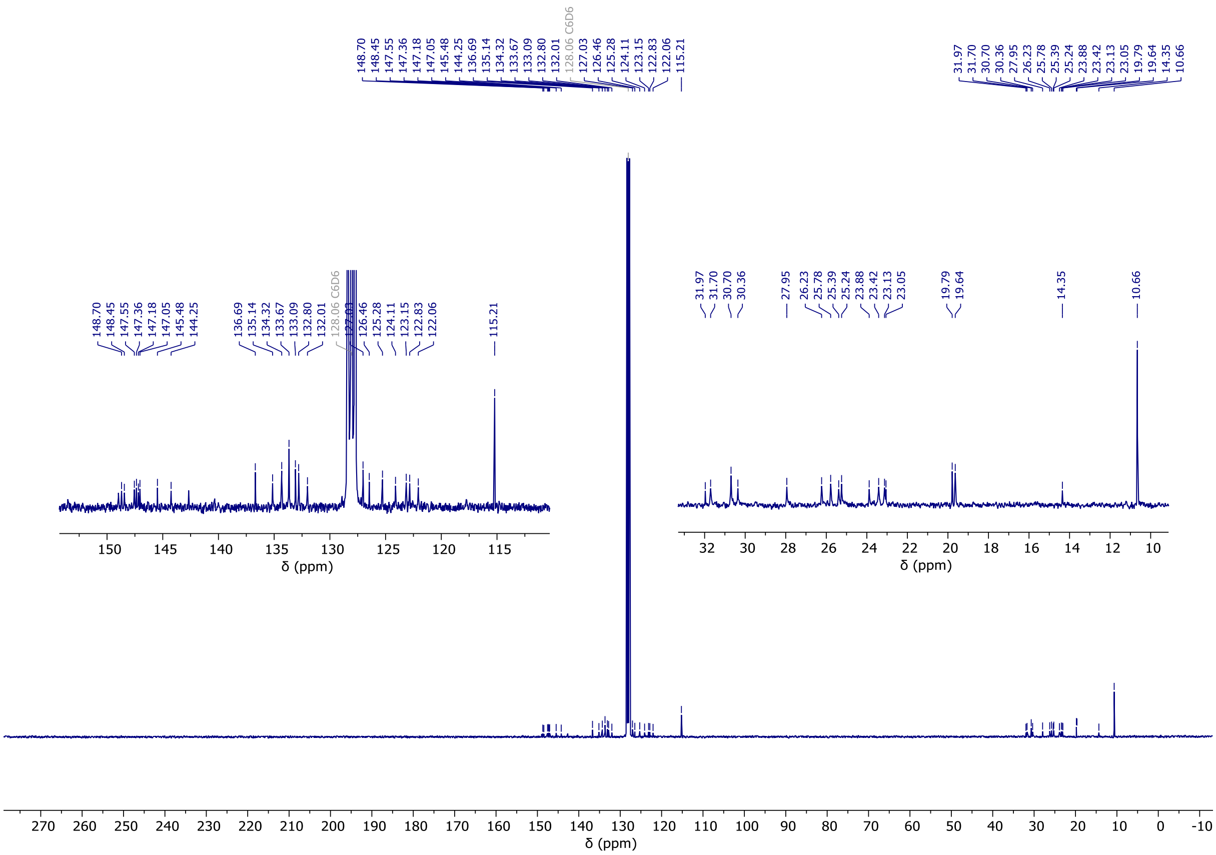

Figure S79: IR spectrum of **2As** (ATR, 32 scans, cm^{-1}, powder).

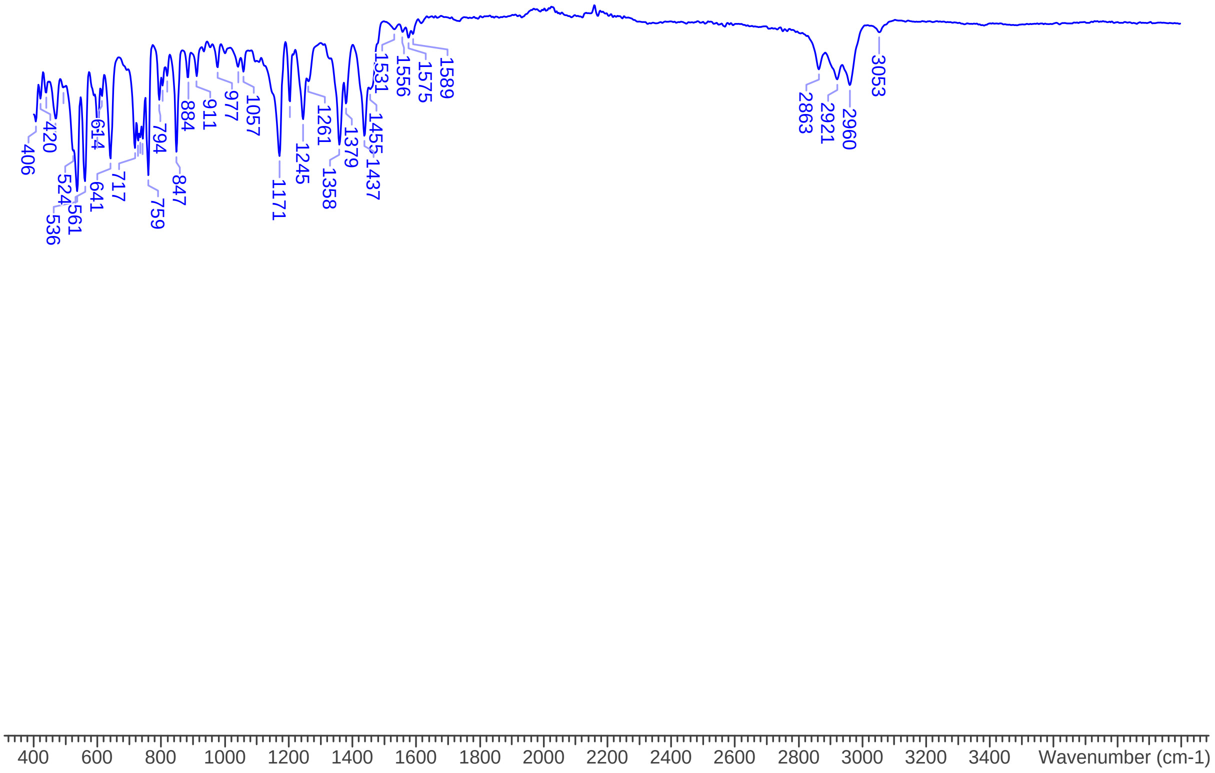

4.9 Compound 3P by reaction of ^{Dipp}TerPAlCp* with 1-azido-2,6-diisopropylbenzene

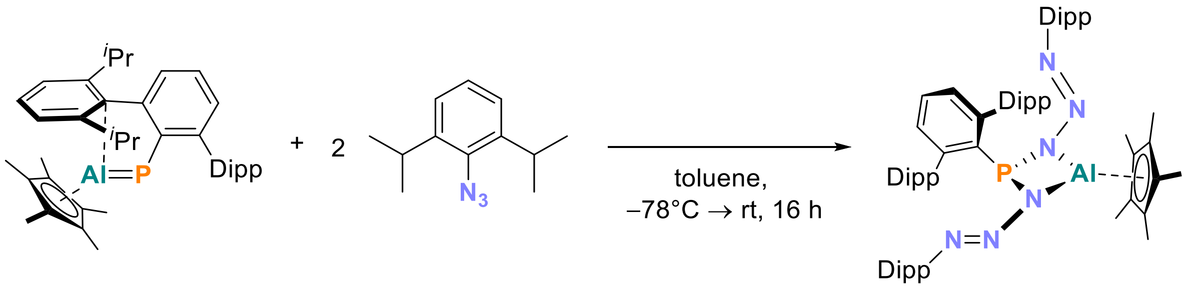

3P (37%)

^{Dipp}TerPAlCp* (20.0 mg, 33.9 μmol, 1.00 eq.) was dissolved in toluene (0.3 mL) in a *J.-Young* NMR tube and was cooled to –78 °C (N_2(l)/*i*-PrOH cooling bath). 1-Azido-2,6-diisopropylbenzene (16.0 mg, 78.8 μmol, 2.30 eq.) was dissolved in toluene (0.3 mL) in a second *J.-Young* NMR tube and cooled to –78 °C (N_2(l)/*i*-PrOH cooling bath). Both *J.-Young* NMR tubes were connected to the Schlenk line via NMR tube adapter and the slightly yellow azide solution was rapidly (within 5 sec.) cannulated with a PTFE cannula (approx. length 60 cm long and diameter 1.5 mm) to the purple solution of **^{Dipp}TerPAlCp***. After closing the *J.-Young* NMR tube at –78 °C (N_2(l)/*i*-PrOH cooling bath) in a argon counterflow with a *J.-Young* NMR tube cap in the NMR tube adapter, the reaction mixture was shaken for 3 s. The reaction solution turned yellow immediately upon mixing it. Then, the reaction mixture was slowly warmed up to ambient temperature overnight (16 h). All volatile components were removed at ambient temperature *in vacuo* (~1×10^{-3} mbar). In a glovebox, the obtained yellow oil was re-dissolved in a mixture of *n*-hexane (0.6 mL) and HMDSO (0.05 mL) and filtered using a pipette equipped with a glass microfiber paper. The *n*-hexane (0.6 mL) / HMDSO (0.05 mL) mixture was placed in a small vial and the solvent was slowly removed by placing the small vial in a closed, bigger vial containing silicon grease. The solution was stored at –30 °C for 24 h until **3P** crystallized. The remaining supernatant was removed with a glass pipette and the colourless crystals were washed with cold *n*-pentane (–30 °C) (2 × 0.1 mL), then dried by evaporation of the remaining solvent at ambient temperature in the glovebox.

Yield of 3P: 12.5 mg (12.5 μmol, 37%), as colourless, crystalline solid.

Suitable crystals for single crystal X-ray diffraction were obtained by slow evaporation of a saturated solution of **3P** in *n*-hexane (0.6 mL) and HMDSO (0.05 mL) at −30 °C in a glovebox with silicon grease as solvent absorbent.

Note: At ambient temperature, compound **3P** slowly decomposes in solution (benzene). Heating of compound **3P** for 6 h at 80 °C in C_6D_6 led to the selective and full conversion of **3P** and was confirmed via $^{31}P\{^1H\}$ NMR spectroscopy to an unknown product with a $^{31}P\{^1H\}$ NMR shift of 102.1 ppm. Attempts of lyophilisation and washing of the resulting yellow solid, analogue to reprocessing of **1P** and **1P_F** or crystallization in benzene or *n*-pentane were not successful and led to a yellow/orange oily compound.

^{1}H NMR (500.1 MHz, C_6D_6, 298 K): δ = 0.96 (d, $^3J_{(H-H)}$ = 6.8 Hz, 6 H, CH(C*H*$_3$)(CH$_3$), (group A)[xxii]), 0.98 (d, $^3J_{(H-H)}$ = 6.8 Hz, 6 H, CH(C*H*$_3$)(CH$_3$), (group B)), 1.04 (d, $^3J_{(H-H)}$ = 6.8 Hz, 6 H, CH(C*H*$_3$)(CH$_3$), (group C)), 1.07 (d, $^3J_{(H-H)}$ = 6.8 Hz, 6 H, CH(CH$_3$)(C*H*$_3$), (group B)), 1.20 (d, $^3J_{(H-H)}$ = 6.8 Hz, 6 H, CH(CH$_3$)(C*H*$_3$), (group C)), 1.23 (d, $^3J_{(H-H)}$ = 6.8 Hz, 6 H, CH(C*H*$_3$)(CH$_3$), (group D)), 1.29 (d, $^3J_{(H-H)}$ = 6.8 Hz, 6 H, CH(CH$_3$)(C*H*$_3$), (group D)), 1.35 (d, $^3J_{(H-H)}$ = 6.8 Hz, 6 H, CH(CH$_3$)(C*H*$_3$), (group A)), 1.78 (s, 15 H, C(C*H*$_3$)$_5$), 2.81 (superimposed ψ-hept, $^3J_{(H-H)}$ = 6.8 Hz, 4 H, C*H*(CH$_3$)(CH$_3$), (group C + B)), 2.97 (ψ-hept, $^3J_{(H-H)}$ = 6.8 Hz, 2 H C*H*(CH$_3$)(CH$_3$), (group D)), 3.05 (ψ-hept, $^3J_{(H-H)}$ = 6.8 Hz, 2 H, C*H*(CH$_3$)(CH$_3$), (group A)), 6.82 (dd, $^3J_{(H-H)}$ = 7.3 Hz, $^4J_{(H-H)}$ = 1.6 Hz, 1 H, *m-H* (^{Dipp}Ter) (group E), 6.97 (d, $^3J_{(H-H)}$ = 7.7 Hz, 2 H, *m-H* (Dipp) (group F)), 6.98 (dd, $^3J_{(H-H)}$ = 7.3 Hz, $J_{(H-P)}$ = 0.7 Hz, 1 H *m-H* (^{Dipp}Ter) (group E)[xxiii], 7.00 (ddd, $^3J_{(H-H)}$ = 7.3 Hz, $J_{(H-P)}$ = 3.5 Hz, $^4J_{(H-H)}$ = 1.6 Hz, 1 H, *p-H* (^{Dipp}Ter) (group E)[xxiii], 7.13 – 7.15

[xxii] *Note*: Due to the same substituents, the differentiation between the Dipp groups were challenging and to assign all resonances that belong to one *i*Pr group the labels A, B, C, D were used. A similar differentiation appears for the resonances of the ^{Dipp}Ter ligand and Dipp groups **E**, **F**, G substituent in the aromatic region.

[xxiii] Appears as dd in $^1H\{^{31}P\}$ NMR spectrum.

(m, 5 H, 2 *m-H* (Dipp) (group G) + 2 *p-H* (Dipp) (group G), *p-H* (Dipp) (group F))[xxiv], 7.19 (m, 2 H, *m-H* (Dipp) (group G)), 7.27 – 7.24 (m, 2 H, *m-H* (Dipp) (group F)), 7.31 (m 1 H, *p-H* (Dipp) (group F)) ppm. **^{13}C{^{1}H} NMR** (125.7 MHz, C_6D_6, 298 K): δ = 10.4 (s, $C_5(CH_3)_5$), 23.1 (s, $CH(CH_3)(CH_3)$, (group C)), 23.7 (s, $CH(CH_3)(CH_3)$, (group A)), 23.8 (s, $CH(CH_3)(CH_3)$, (group D)), 24.1 (d, $J_{(C\text{-}P)}$ = 2.8 Hz, $CH(CH_3)(CH_3)$, (group B)), 26.0 (s, $CH(CH_3)(CH_3)$, (group A)), 26.3 (s, $CH(CH_3)(CH_3)$, (group C)), 26.7 (d, $J_{(C\text{-}P)}$ = 2.4 Hz, $CH(CH_3)(CH_3)$, (group C)), 27.0 (s, $CH(CH_3)(CH_3)$, (group D)), 27.0 (s, $CH(CH_3)(CH_3)$, (group B)), 27.4 (s, $CH(CH_3)(CH_3)$, (group D)), 30.7 (s, $CH(CH_3)(CH_3)$, (group A)), 30.8 (s, $CH(CH_3)(CH_3)$, (group B)), 115.7 (s, $C_5(CH_3)_5$), 122.7 (s, *m*-CH (Dipp) (group F)), 123.2 (s, *m*-CH (Dipp) (group G)), 123.3 (s, *m*-CH (Dipp) (group F)), 123.7 (s, *m*-CH (Dipp) (group G)), 126.1 (s, *p*-CH (Dipp) (group G), 127.7 (s, *m*-CH (^{Dipp}Ter) (group E)), 128.4 (s, *p*-CH (Dipp) (group F))[xxv], 128.7 (s, *p*-CH (Dipp) (group F))[xxv], 131.5 (d, $J_{(C\text{-}P)}$ = 7.8 Hz, *p*-CH (^{Dipp}Ter) (group E)), 132.7 (s, *m*-CH (^{Dipp}Ter) (group E)), 138.9 (d, $J_{(C\text{-}P)}$ = 9.0 Hz, *o-C* (Dipp) (group F)), 140.6 (s, *o-C* (Dipp) (group F)), 140.8 (s), *o-C* (^{Dipp}Ter) (group E)), 141.0 (s, *o-C* (Dipp) (group G)), 141.3 (s, *o-C* (Dipp) (group G)), 142.5 (d, $J_{(C\text{-}P)}$ = 2.8 Hz, *o-C* (^{Dipp}Ter) (group E)), 146.9 (s, *i-C* (Dipp) (group G)), 148.0 (s, *i-C* (Dipp) (group F)), 149.6 (d, $^{1}J_{(C\text{-}P)}$ = 47.6 Hz, *i-C* (^{Dipp}Ter) (group E)) ppm. **^{31}P{^{1}H} NMR** (202.4 MHz, C_6D_6, 298 K) δ = 57.1 (s) ppm. **IR** (ATR, 32 scans, cm^{-1}): $\tilde{\nu}$= 3354 (vw), 3255 (vw), 3059 (w), 2960 (m), 2925 (m), 2865 (w), 1577 (w), 1459 (m), 1439 (m), 1402 (w), 1381 (m), 1360 (m), 1344 (w), 1307 (m), 1235 (vs), 1179 (s), 1123 (s), 1082 (s), 1057 (m), 1043 (m), 981 (s), 936 (m), 886 (m), 868 (m), 802 (s), 794 (s), 775 (m), 759 (s), 750 (s), 705 (m), 695 (m), 660 (m), 637 (s), 606 (m), 587 (m), 563 (m), 550 (m), 534 (m), 513 (m), 488 (m), 462 (m), 447 (m), 437 (m), 429 (m). **LIFDI-MS**: (m/z) calc: 996.6466 [$C_{64}H_{86}AlN_6P$]; (m/z) found: 737.4946 [M−(Dipp-N_2)−(Dipp)+(toluene)]$^+$, 619.4168 [M−(Dipp-N_2)−(Dipp-N_2)]$^+$, 604.4060 [M−(Dipp-N_3)−(Dipp-N_2)]$^+$. **CHN** calc. (found) in %: C 77.07 (70.79), H 8.69 (8.08), N 8.43 (7.09).

[xxiv] Superimposed with C_6D_6 resonance, assigned with ^{1}H/^{1}H COSY and ^{1}H/^{13}C HSQC NMR spectra.
[xxv] Superimposed with C_6D_6 signal, assigned with ^{1}H/^{13}C HSQC and ^{1}H/^{13}C HMBC NMR spectra.

Figure S80: ^{1}H NMR spectrum of **3P** (500.1 MHz, C_6D_6, 298 K); 0.12 ppm: 3% HMDSO.

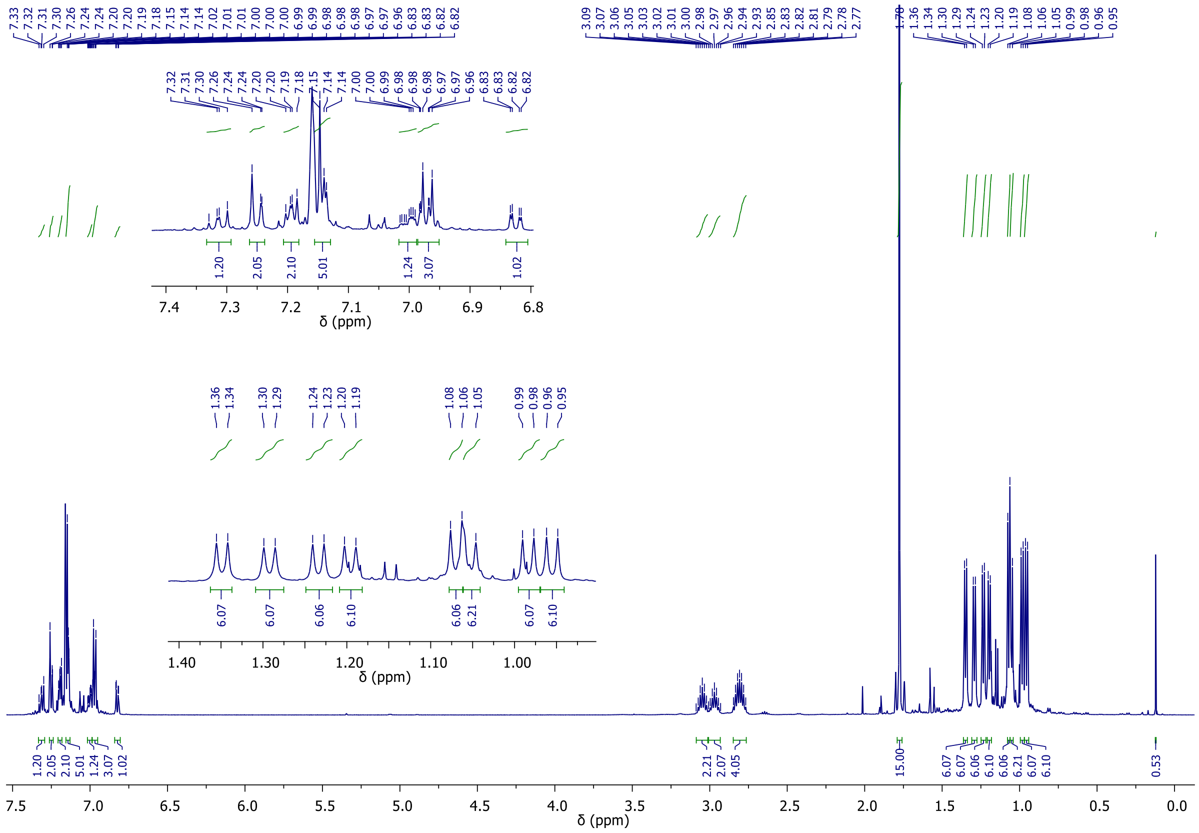

Figure S81: $^{13}C\{^{1}H\}$ NMR spectrum of **3P** (125.7 MHz, C_6D_6, 298 K) 2.1 ppm: 3% HMDSO.

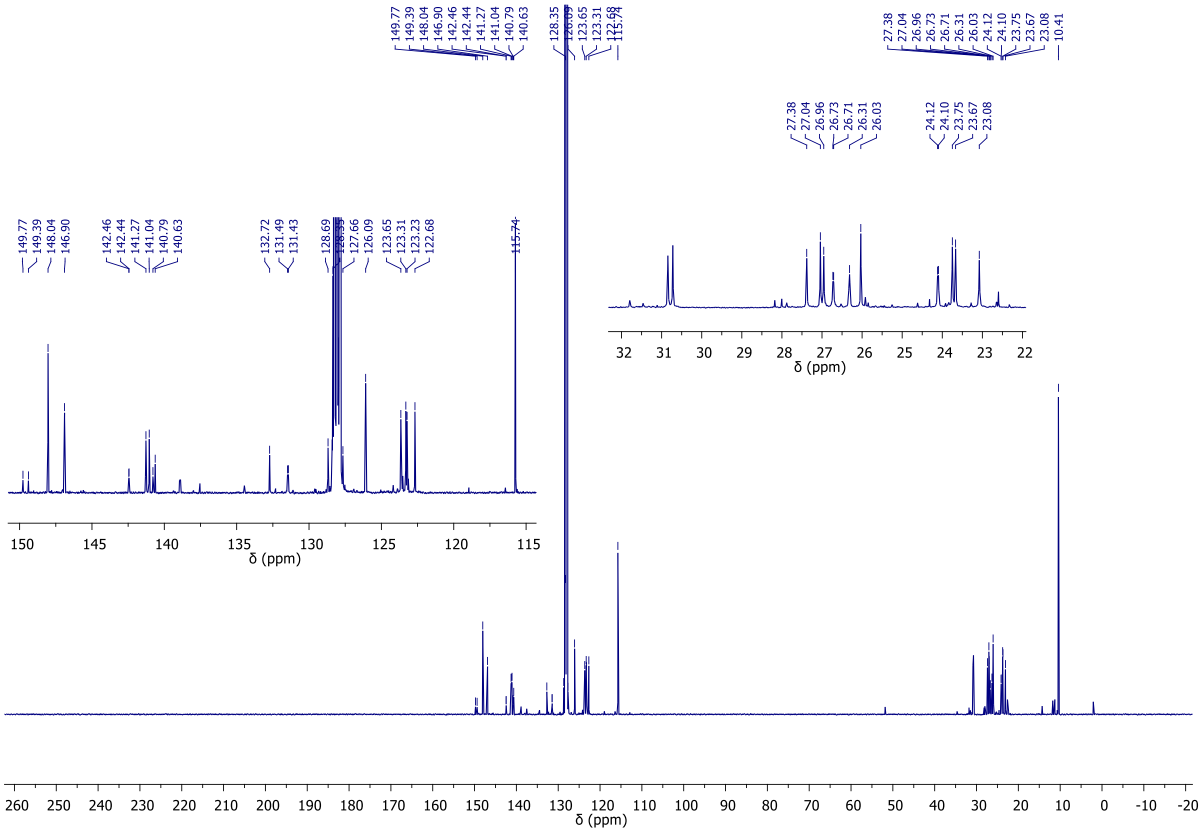

Figure S82: $^{31}P\{^{1}H\}$ NMR spectrum of **3P** (202.5 MHz, C_6D_6, 298 K).

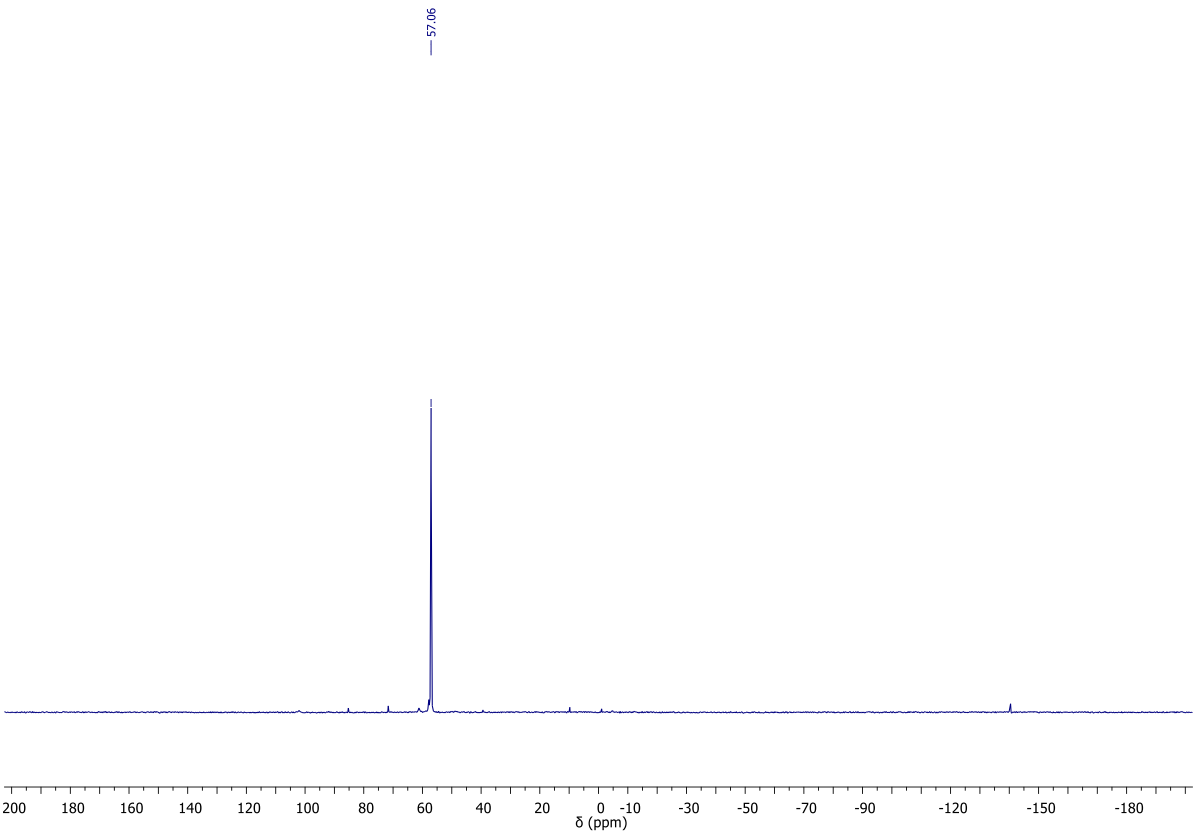

Figure S83: LIFDI mass spectrum of **3P** (toluene).

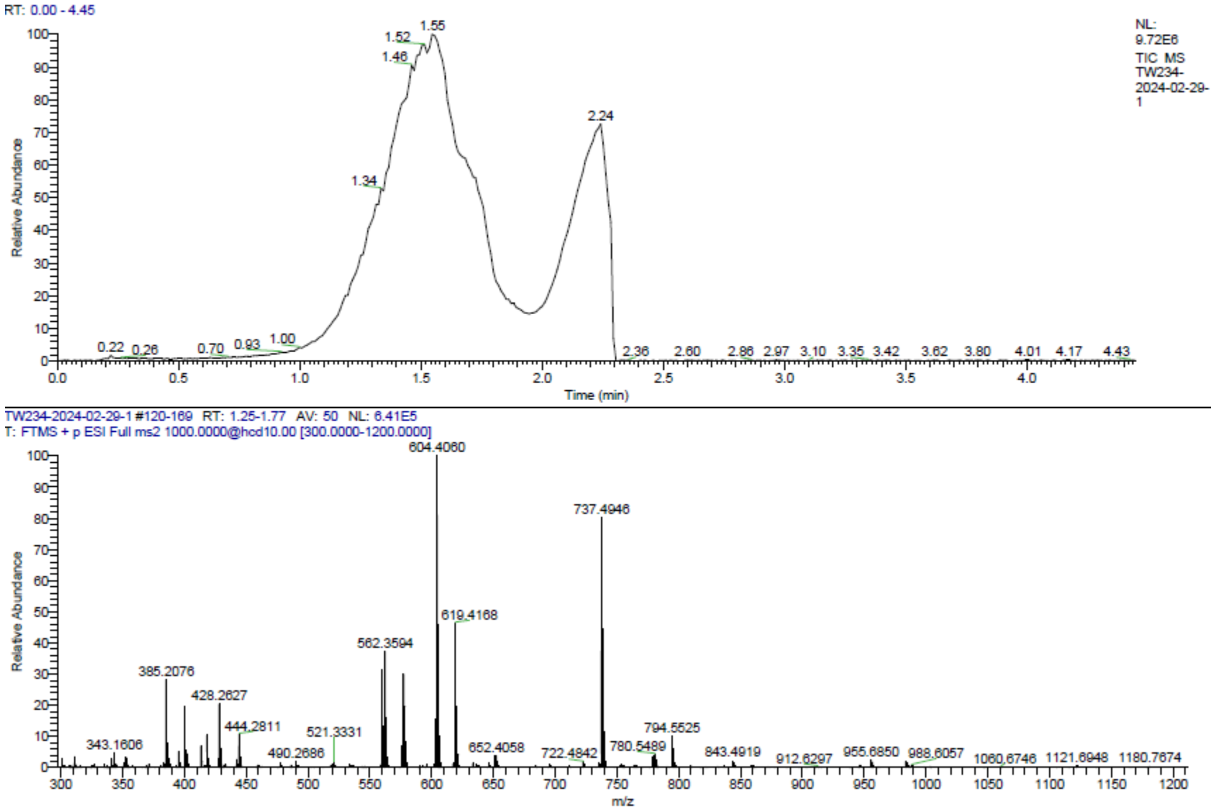

Figure S84: IR spectrum of **3P** (ATR, 32 scans, cm^{-1}, powder).

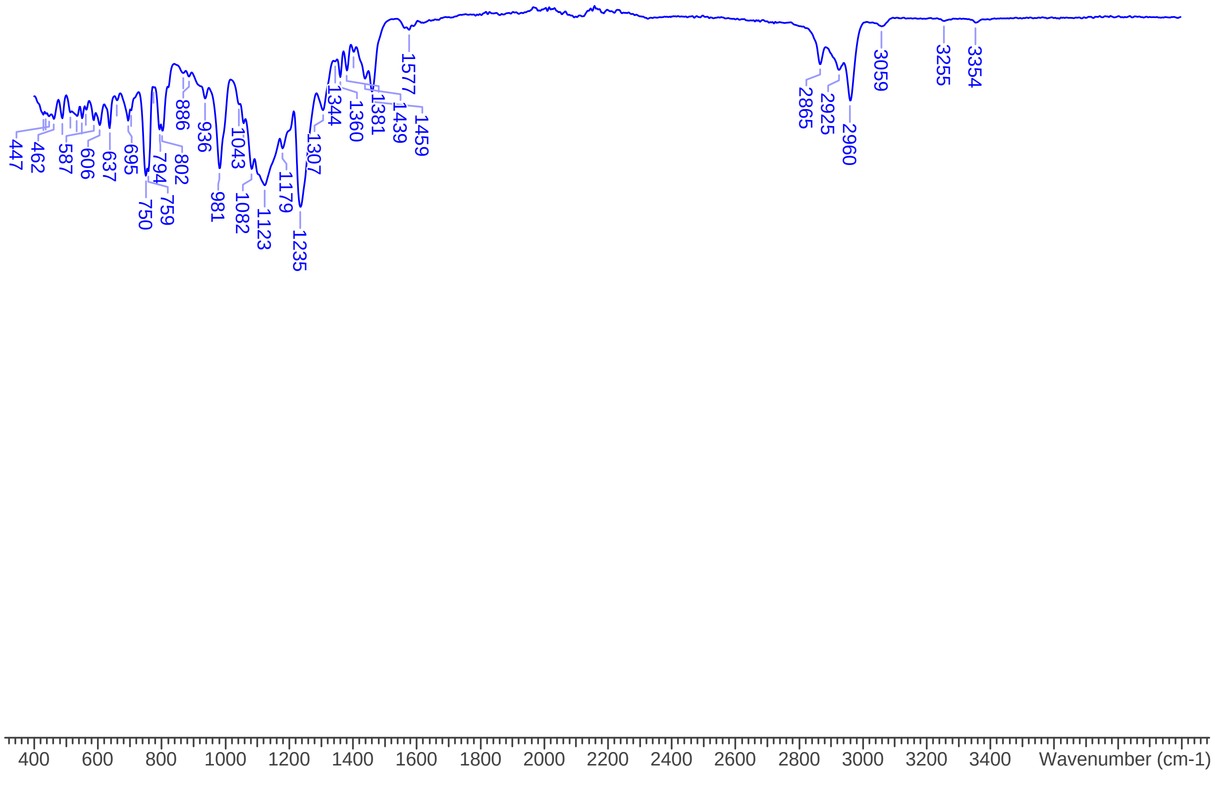

Figure S85: $^{31}P\{^{1}H\}$ NMR spectrum of the reaction mixture after heating **3P** at 80 °C for 6 h in C_6D_6 (162.0 MHz, C_6D_6, 298 K).

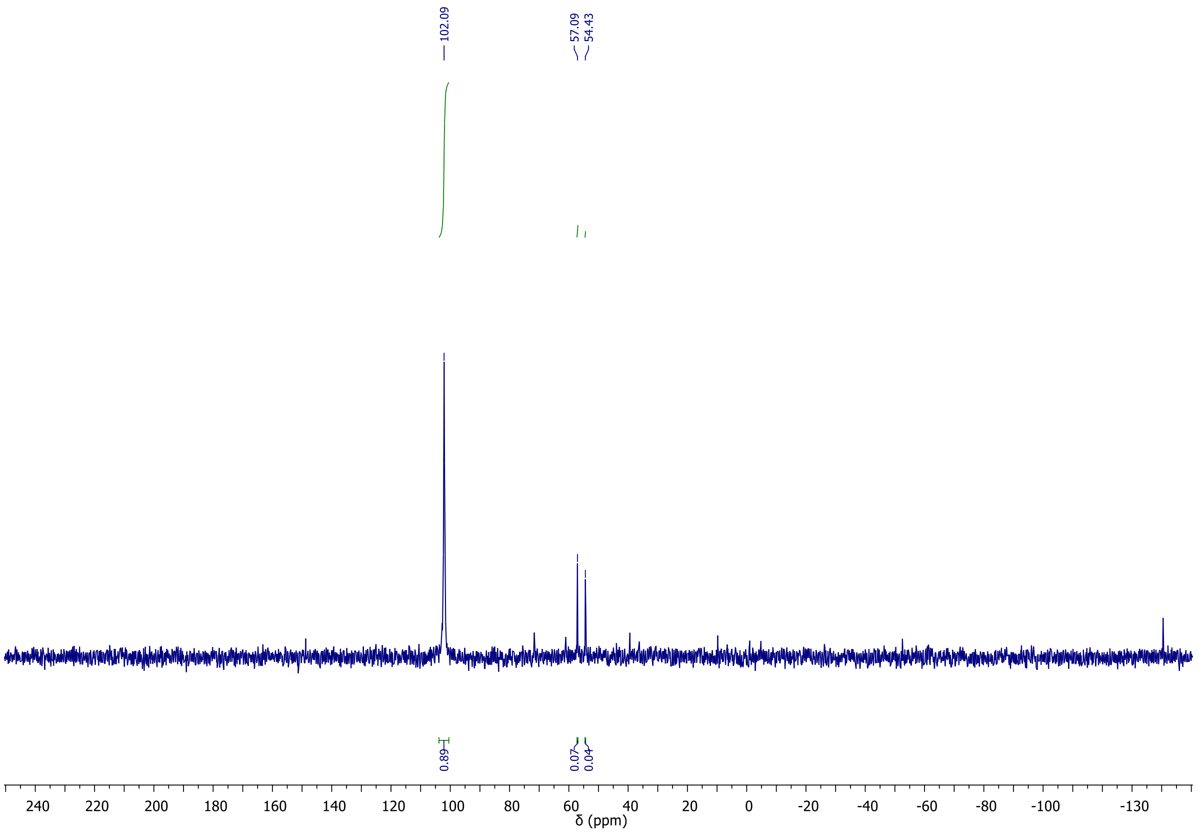

4.10 Reaction of ^{Dipp}TerPAlCp* with a 1:1 mixture of $DippN_3$ and 2-azido-1,3-dibromo-5-methylbenzene

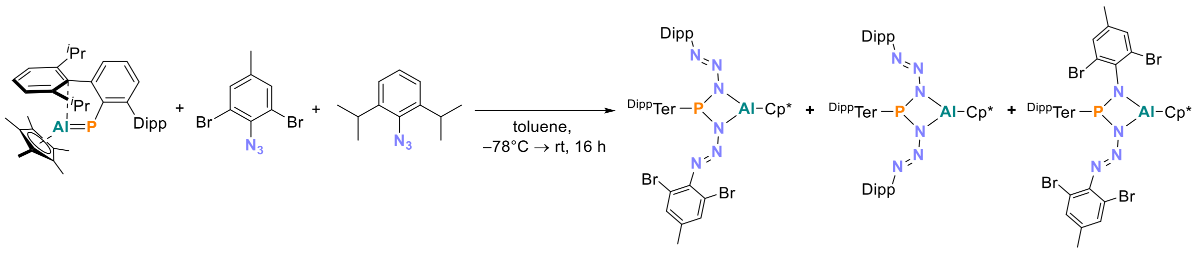

^{Dipp}TerPAlCp* (30.0 mg, 50.8 µmol, 1.00 eq.) was dissolved in toluene (0.3 mL) in a *J.-Young* NMR tube and was cooled to –78 °C (N_2(l)/*i*-PrOH cooling bath). 1-Azido-2,6-diisopropylbenzene (10.3 mg, 50.8 µmol, 1.00 eq.) and 2-azido-1,3-dibromo-5-methylbenzene (14.7 mg, 50.8 µmol, 1.00 eq.) were dissolved in toluene (0.3 mL) in a second *J.-Young* NMR tube and cooled to –78 °C (N_2(l)/*i*-PrOH cooling bath). Both *J.-Young* NMR tubes were connected to the Schlenk line via NMR tube adapter and the slightly yellow azide solution was rapidly (within 5 sec.) cannulated with a PTFE cannula (approx. length 60 cm long and diameter 1.5 mm) to the purple solution of **^{Dipp}TerPAlCp***. After closing the *J.-Young* NMR tube at –78 °C (N_2(l)/*i*-PrOH cooling bath) in an argon counterflow with a *J.-Young* NMR tube cap in the NMR tube adapter, the reaction mixture was shaken for 3 s. The reaction solution turned yellow immediately upon mixing it. Then, the reaction mixture was slowly warmed up to ambient temperature overnight (16 h). All volatile components were removed at ambient temperature *in vacuo* (~1×10^{-3} mbar). In a glovebox, the obtained yellow oil was re-dissolved in *n*-hexane (0.6 mL) and filtered using a pipette equipped with a glass microfiber paper. The *n*-hexane (0.6 mL) solution was placed in a small vial and the solvent was slowly removed by placing the small vial in a closed, bigger vial containing silicon grease. The solution was stored at ambient temperature for 24 h until **3P_mixed** crystallized. It was not possible to isolate **3P_mixed** as pure compound due to co-crystallization with **2P** and **3P**.

^{31}P{^{1}H} NMR (202.4 MHz, C_6D_6, 298 K): δ = 60.8 (s) ppm.

Figure S86: $^{31}P\{^{1}H\}$ NMR spectrum of the crude reaction mixture containing **3P_mixed** (162.2 MHz, Tol-d_8, 298 K).

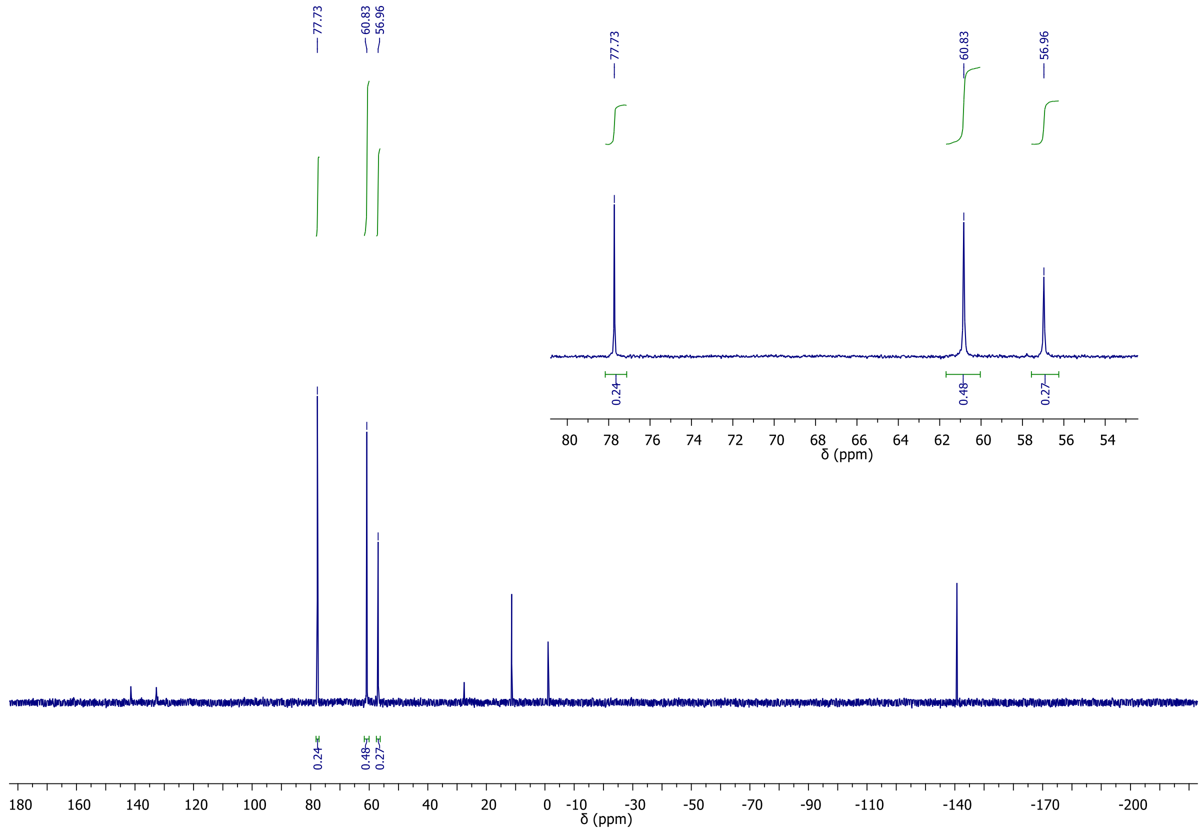

4.11 Compound 3As by reaction of ^{Dipp}TerAsAlCp* with 1-azido-2,6-diisopropylbenzene

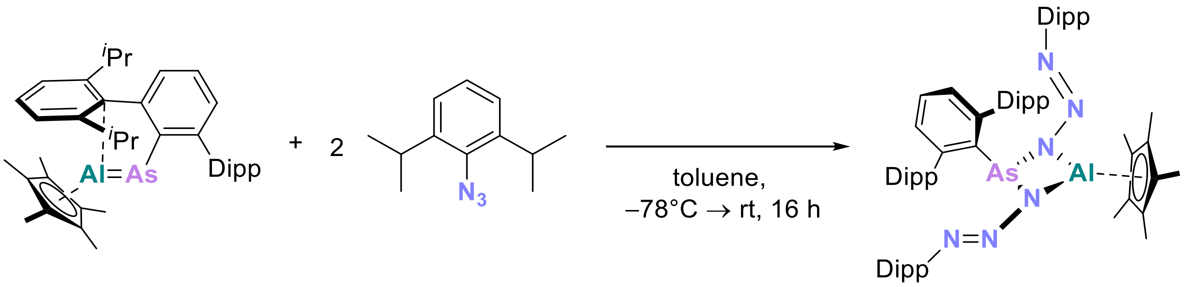

3As (40%)

^{Dipp}TerAsAlCp* (50.0 mg, 78.8 μmol) was dissolved in toluene (2.5 mL) at 45 °C (water bath). The solution was cooled to −78 °C (CO_2/EtOH cooling bath) and a solution of $DippN_3$ (32.0 mg, 160 μmol) in toluene (2.5 mL) was added over a period of 10 min in the dark, whereby a colour change from blue to yellow occurred. The reaction mixture was allowed to slowly warm to ambient temperature by keeping it in the cooling bath overnight. The solvent was removed *in vacuo* and the residual solid was dried for 15 min at ambient temperature at 1×10^{-3} mbar. The residue was then extracted with *n*-hexane (15 mL). The insoluble material was removed by filtration through a plug of celite. The filtrate was concentrated rapidly to ~2 mL *in vacuo* and was placed in a bowl of warm water (approx. 45 °C) and was then transferred to a fridge (5 °C) to afford colourless crystals overnight. The supernatant was removed by syringe and the crystals were washed twice with cold (−78 °C) *n*-hexane (1 ml each). Supernatant and washing solution were combined to get a second fraction of product analogous to the procedure described above. The isolated crystalline fractions were dried for 1 h at ambient temperature at 1×10^{-3} mbar.

Yield of 3As: 33.0 mg (32.0 μmol, 40%) of a colourless, crystalline solid.

Single crystals suitable for X-ray diffraction were grown as described in the synthesis procedure.

CHN calc. (found) in %: C 73.82 (73.47), H 8.32 (7.57), N 8.07 (5.53); deviations probably due to incomplete combustion, repeated measurements with and without adding V_2O_5 as additional oxidation reagent did not result in better agreement. **^{1}H NMR** (C_6D_6, 300.2 MHz): δ = 0.95 − 1.00 (superimposed signals, 12 H, C(H)(C*H*$_3$)(CH$_3$) (2x)), 1.03 – 1.09 (superimposed signals, 12 H, C(H)(C*H*$_3$)(CH$_3$) (2x)), 1.14 − 1.22 (superimposed signals, 12 H, C(H)(C*H*$_3$)(CH$_3$) (2x)), 1.34 (d, $^3J_{(H-H)}$ = 6.8 Hz, 6 H, C(H)(C*H*$_3$)(CH$_3$)), 1.38 (d, $^3J_{(H-H)}$ = 6.7 Hz, 6 H, C(H)(C*H*$_3$)(CH$_3$)), 1.79 (s, 15 H, C_5(C*H*$_3$)$_5$), 2.73 – 2.94 (superimposed signals, 4 H, C(*H*)(CH$_3$)(CH$_3$) (2x)), 3.00 − 3.18 (superimposed signals, 4 H, C(*H*)(CH$_3$)(CH$_3$) (2x)), 6.85 − 7.36 (superimposed signals, 15 H, aromatic C*H*). **^{13}C{^{1}H} NMR** (C_6D_6, 75.5 MHz): δ = 10.4 (s, C_5(CH$_3$)$_5$), 23.0 (s, C(H)(CH$_3$)(CH$_3$)), 23.7 (s, C(H)(CH$_3$)(CH$_3$)), 23.8 (s, C(H)(CH$_3$)(CH$_3$)), 23.9 (s, C(H)(CH$_3$)(CH$_3$)), 25.7 (s, C(H)(CH$_3$)(CH$_3$)), 25.9 (s, C(H)(CH$_3$)(CH$_3$)), 26.3 (s, C(H)(CH$_3$)(CH$_3$)), 26.9 (s, C(H)(CH$_3$)(CH$_3$)), 27.1 (s, *C*(H)(CH$_3$)(CH$_3$)), 27.4 (s, *C*(H)(CH$_3$)(CH$_3$)), 30.7 (s, *C*(H)(CH$_3$)(CH$_3$)), 30.9 (s, *C*(H)(CH$_3$)(CH$_3$)), 115.5 (s, *C*$_5$(CH$_3$)$_5$), 122.7 (s, aromatic *C*H), 123.1 (s, aromatic *C*H), 123.2 (s, aromatic *C*H), 123.7 (s, aromatic *C*H), 125.9 (s, aromatic *C*H), 127.5 (s, aromatic *C*H), 131.0 (s, aromatic *C*H), 133.2 (s, aromatic *C*H), 140.3 (aromatic $C_{quart.}$), 141.2 (aromatic $C_{quart.}$), 141.3 (aromatic $C_{quart.}$), 143.2 (aromatic $C_{quart.}$), 146.0 (aromatic $C_{quart.}$), 146.8 (aromatic $C_{quart.}$), 147.9 (aromatic $C_{quart.}$), 148.4 (aromatic $C_{quart.}$). **IR** (ATR, 32 scans, cm^{-1}): $\tilde{\nu}$ = 3057 (w), 3026 (vw), 2964 (s), 2956 (s), 2925 (m), 2867 (m), 1591 (vw), 1577 (w), 1554 (vw), 1461 (m), 1435 (m), 1408 (s), 1393 (s), 1379 (s), 1358 (m), 1321 (m), 1249 (m), 1212 (m), 1165 (m), 1117 (m), 1099 (m), 1086 (s), 1055 (m), 987 (m), 973 (m), 936 (m), 884 (w), 802 (m), 789 (m), 759 (vs), 744 (s), 678 (s), 608 (s), 596 (s), 585 (s), 536 (m), 517 (s), 478 (s), 456 (m), 433 (m), 420 (m). **MS** (CI, pos., isobutene, m/z): 1043 [M+2 H]$^+$, 178 [Dipp-NH$_3$]$^+$.

Figure S87: ^{1}H NMR spectrum of **3As** (300.2 MHz, C_6D_6, 298 K).

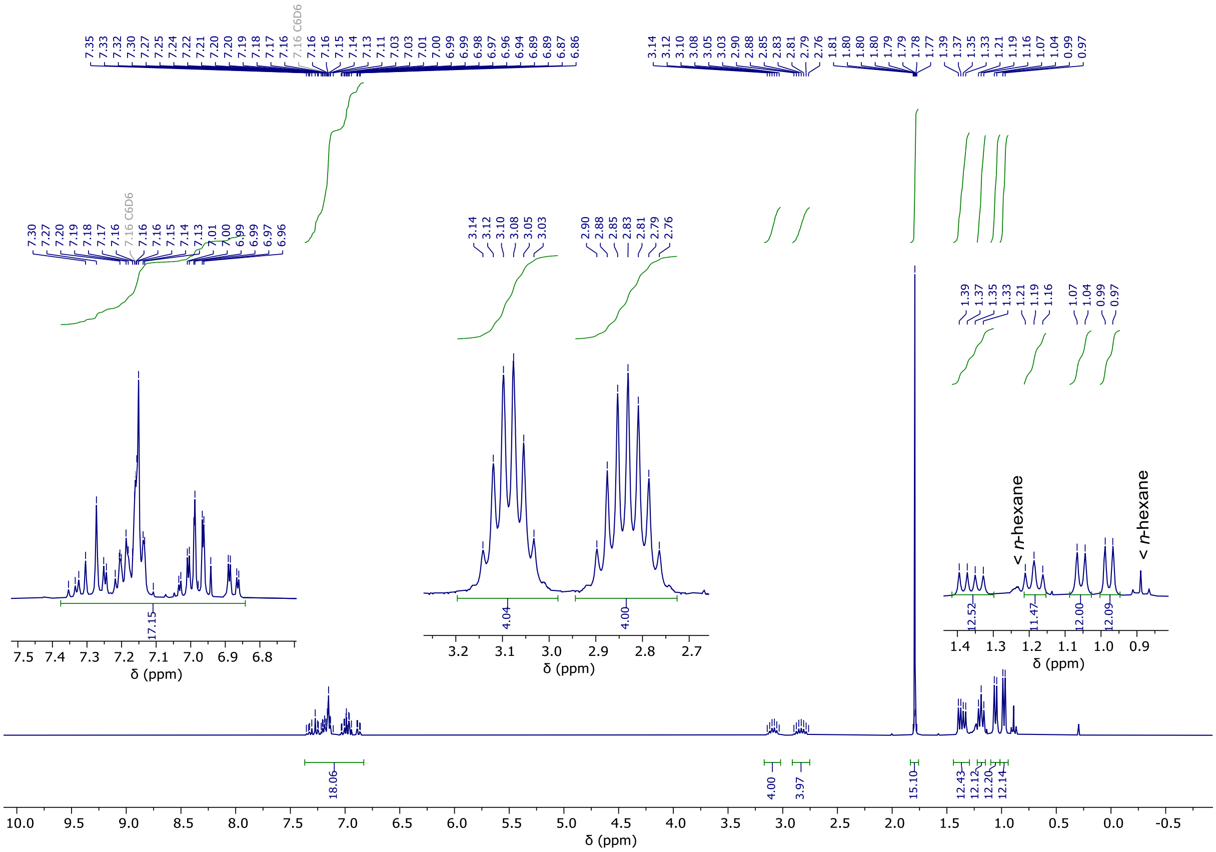

Figure S88: Part of the ^{1}H-^{1}H COSY NMR spectrum of **3As** (300.2 MHz, C_6D_6, 298 K), that shows the correlation between C*H*$(CH_3)(CH_3)$ and CH(C*H*$_3$)(CH_3) as well as $CH(CH_3)$(C*H*$_3$) protons within the Dipp groups. The fact that eight correlation peaks can be seen indicates that there are four groups of chemical inequivalent C*H*$(CH_3)(CH_3)$ protons (f1 domain). Two of these signals overlap with each other. The two methyl groups in one $CH(CH_3)(CH_3)$ group are chemically inequivalent to each other so that the resonances of four chemical inequivalent CH(C*H*$_3$)(CH_3) and four chemical inequivalent $CH(CH_3)$(C*H*$_3$) groups can be seen in the f2 domain. However, there is an overlap between some of them.

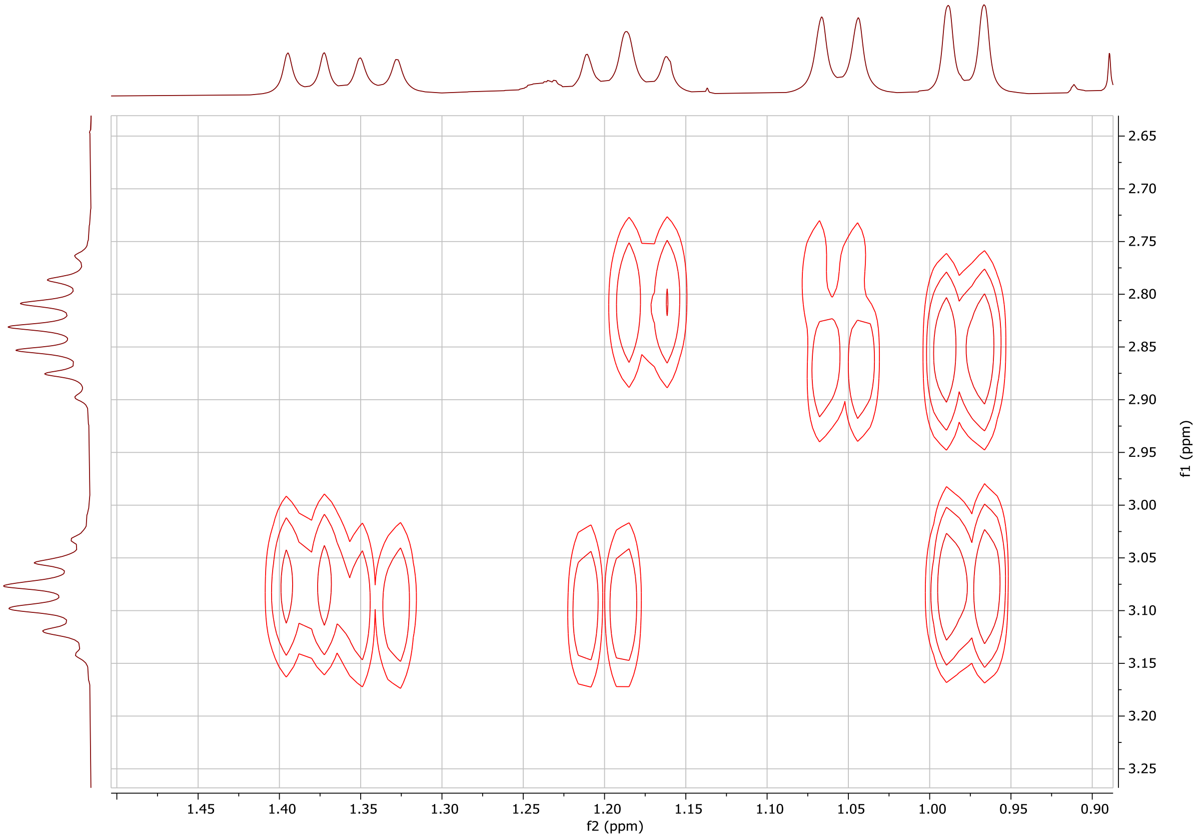

Figure S89: $^{13}C\{^{1}H\}$ NMR spectrum of **3As** (75.5 MHz, C_6D_6, 298 K). The signals at 14.4, 23.1 and 32.0 ppm correspond to *n*-hexane.

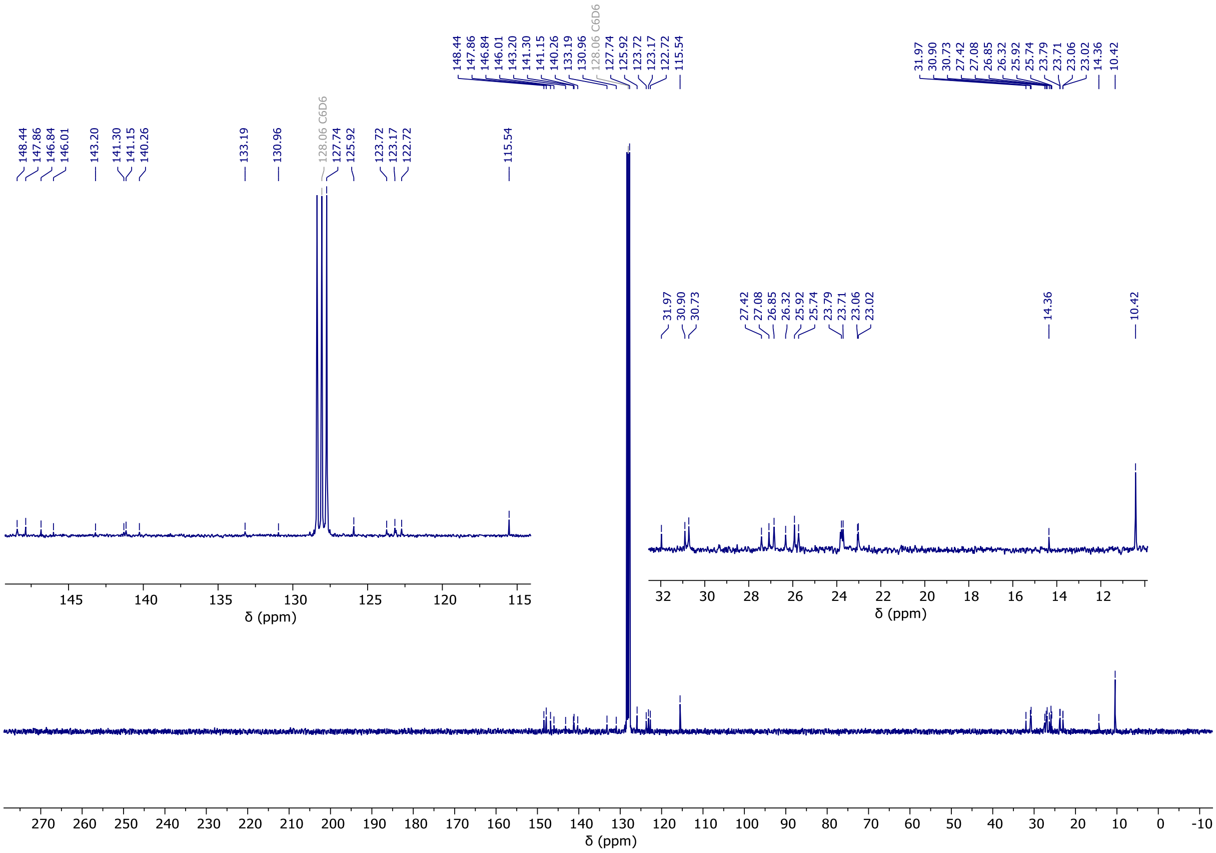

Figure S90: IR spectrum of **3As** (ATR, 32 scans, cm^{-1}, powder).

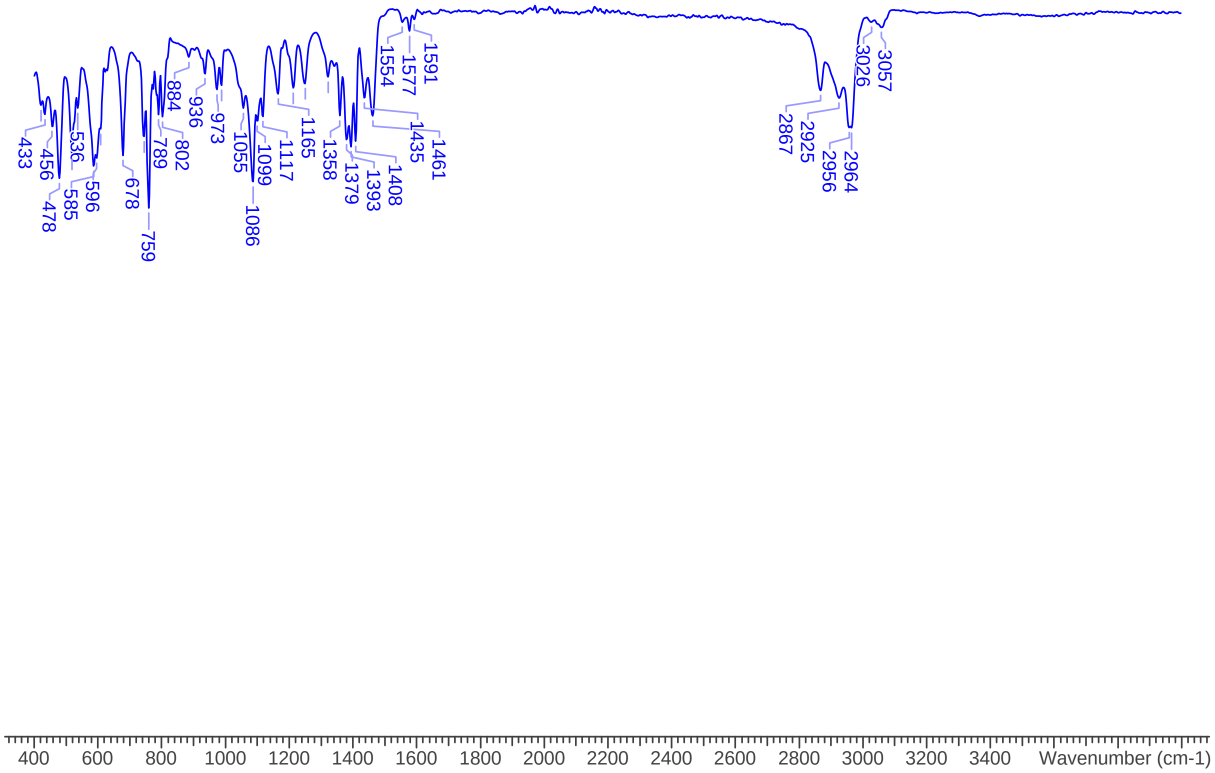

4.12 Compound 4P by reaction of ^{Dipp}TerPAlCp* with (trimethylsilyl)-diazomethane

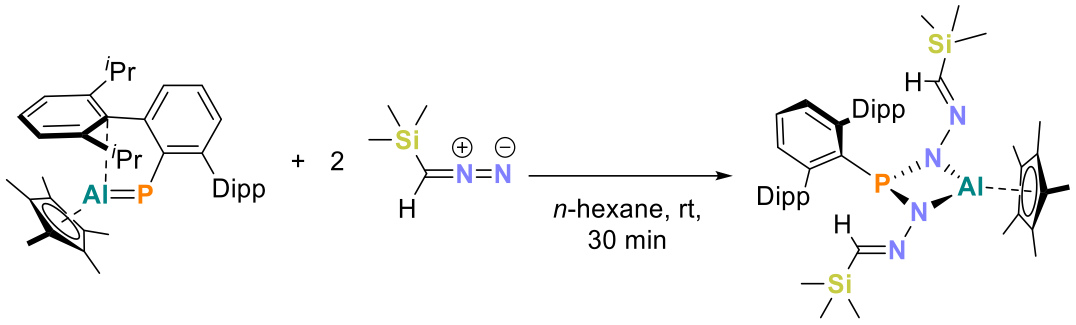

4P (40%)

^{Dipp}TerPAlCp* (20.0 mg, 33.9 μmol, 1.00 eq.) was dissolved in *n*-hexane (0.5 mL) in a *J.-Young* NMR tube. At ambient temperature, a solution of (trimethylsilyl)diazomethane (0.6 M in *n*-hexane, 0.12 mL, 72.0 μmol, 2.10 eq.) was added. The colour of the reaction mixture instantly changed from deep purple to pale-yellow. After 30 min, full conversion of **^{Dipp}TerPAlCp*** to **4P** was confirmed via ^{31}P{^{1}H} NMR spectroscopy. In a glovebox, the pale-yellow solution was filtered using a pipette equipped with a microfiber paper. The *n*-hexane solution was placed in a small vial and the solvent was slowly removed by placing the small vial into a closed, bigger vial that contained silicon grease. The solution was stored at –30 °C for 2 days until crystallization of **4P**. The remaining supernatant was removed with a glass pipette and the solid was carefully washed with cold (–30 °C) *n*-pentane (1 × 0.1 mL) and dried by evaporation of the remaining solvent at ambient temperature in the glovebox.

Yield of 4P: 11.1 mg (13.5 μmol, 40%) of a colourless, crystalline solid.

Suitable crystals for single crystal X-ray diffraction were obtained by slow evaporation of a saturated solution of **4P** in *n*-hexane at ambient temperature in a glovebox with silicon grease as solvent absorbent.

At ambient temperature, the slow decomposition of **4P** in solution was observed over the course of a few hours by a colour change from colourless to yellow (*n*-pentane, *n*-hexane) or orange (benzene-d_6, toluene-d_8) accompanied by the formation of new resonances in the ^{31}P NMR spectra.

CHN calc. (found) in %: C 70.37 (66.51), H 8.86 (8.31), N 6.84 (6.35). **^{1}H NMR** (600.2 MHz, Tol-d_8, 213 K): δ = 0.28 (s, 18 H, $N_2CHSi(CH_3)_3$), 1.04 (d, $^3J_{(H\text{-}H)}$ = 6.6 Hz, 6 H, C(H)(C*H*$_3$)(CH$_3$) (<u>group A</u>)[xxvi]), 1.14 (d, $^3J_{(H\text{-}H)}$ = 6.6 Hz, 6 H, C(H)(C*H*$_3$)(CH$_3$) (<u>group B</u>)), 1.34 (d, $^3J_{(H\text{-}H)}$ = 6.6 Hz, 6 H, C(H)(CH$_3$)(C*H*$_3$) (<u>group B</u>)), 1.42 (d, $^3J_{(H\text{-}H)}$ = 6.6 Hz, 6 H, C(H)(CH$_3$)(C*H*$_3$) (<u>group A</u>)), 1.84 (s, 15 H, $C_5(CH_3)_5$), 2.74 (bs, 2 H, C(*H*)(CH$_3$)(CH$_3$) (<u>group B</u>)), 2.85 (ψ-hept, $^3J_{(H\text{-}H)}$ = 6.6 Hz, 2 H, C(*H*)(CH$_3$)(CH$_3$) (<u>group A</u>)), 6.79 (dd, $^3J_{(H\text{-}H)}$ = 7.5 Hz, $^4J_{(H\text{-}P)}$ = 0.9 Hz, 1 H, *m-H* (^{Dipp}Ter) (1))[xxvii], 6.85 (t, $^3J_{(H\text{-}H)}$ = 7.5 Hz, 1 H, *p-H* (^{Dipp}Ter)), 6.88 (d, $J_{(H\text{-}P)}$ = 4.2 Hz, 2 H, N$_2$C*H*Si(CH$_3$)$_3$)), 7.03 (d, $^3J_{(H\text{-}H)}$ = 7.5 Hz, 1 H, *m-H* (^{Dipp}Ter) (2)), 7.10 (d, $^3J_{(H\text{-}H)}$ = 7.6 Hz, 2H, *m-H* (Dipp) (<u>group C</u>)), 7.12 (d, $^3J_{(H\text{-}H)}$ = 7.8 Hz, 2 H, *m-H* (Dipp) (<u>group D</u>)), 7.17 (t, $^3J_{(H\text{-}H)}$ = 7.6 Hz, 1 H, *p-H* (Dipp) (<u>group C</u>)), 7.36 (t, $^3J_{(H\text{-}H)}$ = 7.8 Hz, 1H, *p-H* (Dipp) (group D)) ppm. **^{13}C{^{1}H} NMR** (150.9 MHz, Tol-d_8, 213 K): δ = −0.7 (s, $N_2CHSi(CH_3)_3$). 10.3 (s, $C_5(CH_3)_5$), 23.0 (s, C(H)(CH$_3$)(CH$_3$) (<u>group B</u>)), 23.6 (s, C(H)(CH$_3$)(CH$_3$) (<u>group A</u>)), 26.1 (s, C(H)(CH$_3$)(CH$_3$) (<u>group A</u>)), 26.6 (s, C(H)(CH$_3$)(CH$_3$) (<u>group B</u>)), 30.6 (s, *C*(H)(CH$_3$)(CH$_3$) (<u>group A</u>)), 31.5 (s, *C*(H)(CH$_3$)(CH$_3$) (<u>group B</u>)), 114.2 (s, *C*$_5$(CH$_3$)$_5$), 122.2 (s, *m*-CH (Dipp) (<u>group C</u>)), 123.1 (s, *m*-CH (Dipp) (<u>group D</u>)), 127.7 (s, *p*-CH (^{Dipp}Ter))[xxviii], 128.2 (s, *p*-CH (Dipp) (<u>group D</u>))[xxviii], 128.5 (s, *p*-CH (Dipp) (<u>group C</u>))[xxviii], 130.1 (d, $J_{(C\text{-}P)}$ = 5.2 Hz, *m*-CH (^{Dipp}Ter) (2)), 132.5 (s, *m*-CH (^{Dipp}Ter) (1)), 139.2 (d, $J_{(C\text{-}P)}$ = 7.0 Hz, *i-C* (Dipp) (<u>group D</u>)), 140.2 (s, *i-C* (Dipp) (<u>group C</u>)), 141.1 (d, $^1J_{(C\text{-}P)}$ = 66.1 Hz, *i-C* (^{Dipp}Ter), 143.2 (s, *o-C* (^{Dipp}Ter) (1)), 144.7 (d, $J_{(C\text{-}P)}$ = 33.7 Hz, $N_2CHSi(CH_3)_3$)), 147.0 (s, *o-C* (Dipp) (<u>group C</u>)), 147.1 (s, *o-C* (Dipp)

[xxvi] *Note*: Due to the rotational hindrance of the bulky TMS and Dipp substituents, the NMR experiment was conducted at low temperature (213 K) to prevent signal broadening due to wobbeling. To assign all resonances that belong to one *i*Pr group the labels <u>A</u> and <u>B</u> and a similar differentiation for the resonances of the ^{Dipp}Ter ligand <u>C</u> and <u>D</u> substituent in the aromatic region were used. The labels <u>1</u> and <u>2</u> were used to differentiate the *meta* Protons at the ^{Dipp}Ter ligand.

[xxvii] Appears as d in the ^{1}H{^{31}P} NMR spectrum.

[xxviii] Superimposed with solvent signal, assigned with ^{1}H/^{13}C HSQC, DEPT-90 and DEPT-135 NMR spectra.

(group D)), 147.5 (d, $J_{(C\text{-}P)}$ = 42.4 Hz, *o*-*C* (^{Dipp}Ter) (2)) ppm. **^{29}Si{^{1}H} NMR** (119.2 MHz, Tol-d$_8$, 213 K): δ = −9.9 (s) ppm. **^{31}P{^{1}H} NMR** (242.9 MHz, Tol-d$_8$, 213 K): δ = 46.8 (s) ppm. **^{31}P{^{1}H} NMR** (162.1 MHz, C_6D_6, 298 K): δ = 49.1 (s) ppm. **IR** (ATR, 32 scans, cm^{-1}): $\tilde{\nu}$ = 3053 (vw), 2960 (m), 2925 (w), 2904 (w), 2867 (w), 1575 (vw), 1554 (vw), 1501 (m), 1459 (w), 1445 (w), 1426 (w), 1381 (w), 1360 (w), 1321 (w), 1296 (w), 1243 (m), 1142 (m), 1053 (m), 1043 (m), 936 (w), 897 (m), 874 (s), 860 (m), 833 (vs), 804 (s), 794 (m), 761 (m), 746 (s), 713 (s), 686 (m), 616 (s), 592 (m), 530 (s), 505 (m), 445 (m). **LIFDI-MS** (m/z) calc: 818.4848 (100%), 819.4882 (51.9%), 820.4915 (13.2%), [$C_{48}H_{72}AlN_4PSi_2$]; found: 818.4848, 819.4877, 820.4910 [$C_{48}H_{72}AlN_4PSi_2$].

Figure S91: ^{1}H NMR spectrum of **4P** (600.2 MHz, Tol-d$_8$, 213 K); 0.94 ppm, 1.26 ppm: *n*-hexane.

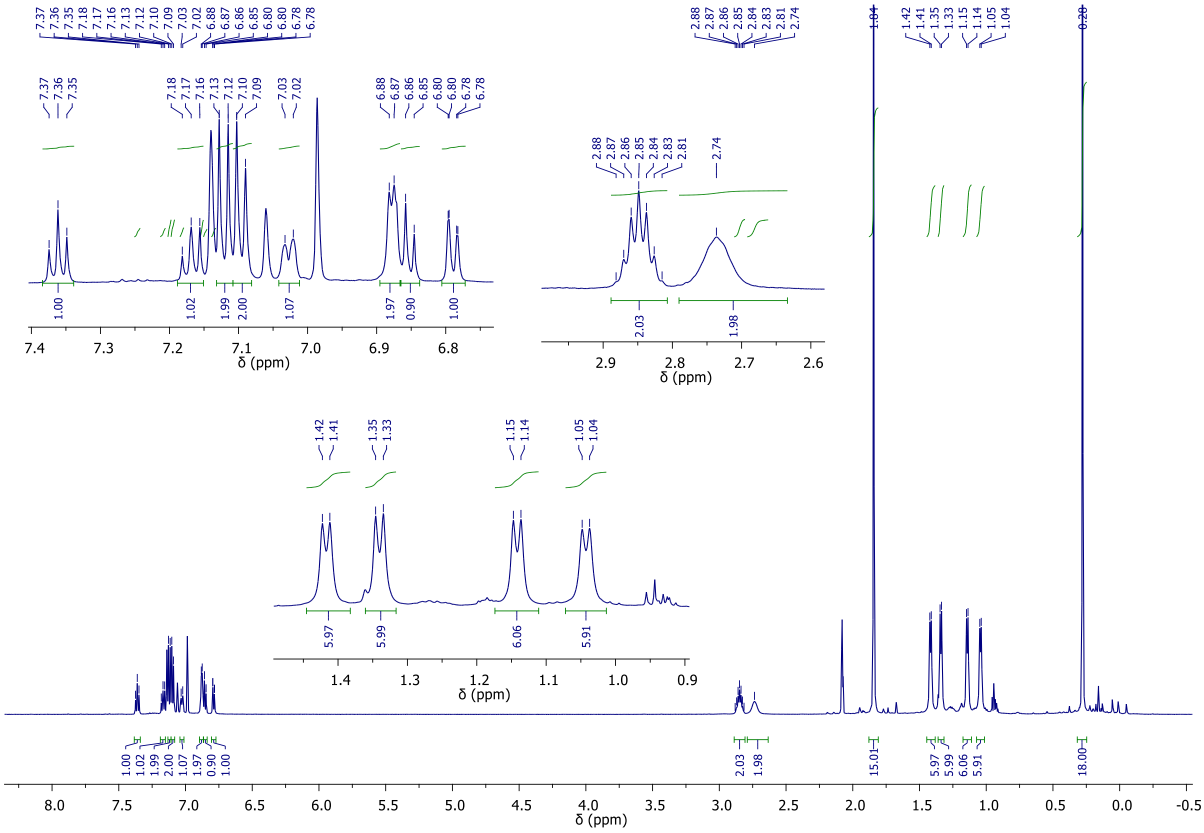

Figure S92: $^{13}C\{^1H\}$ NMR spectrum of 4**P** (150.9 MHz, Tol-d_8, 213 K); 14.7 ppm, 23.3 ppm, 32.3 ppm *n*-hexane.

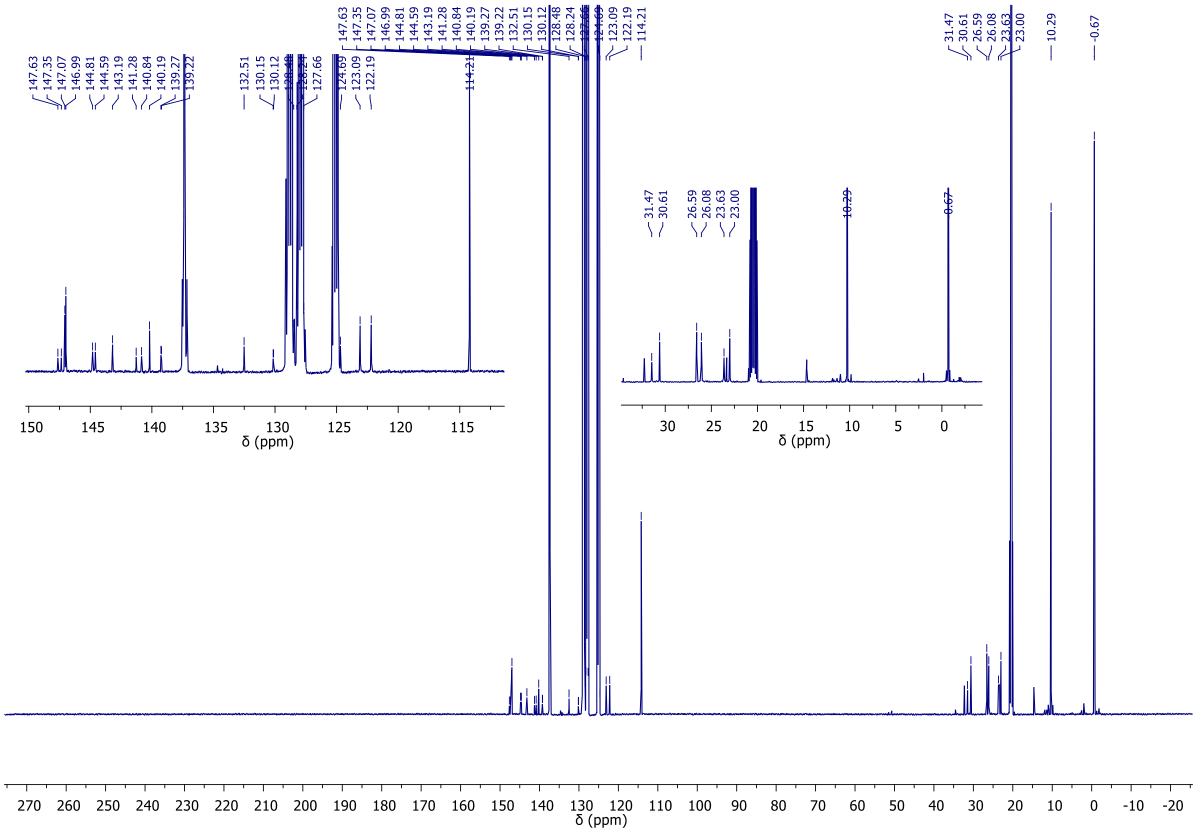

Figure S93: $^{29}Si\{^1H\}$ NMR spectrum of **4P** (119.2 MHz, Tol-d$_8$, 213 K).

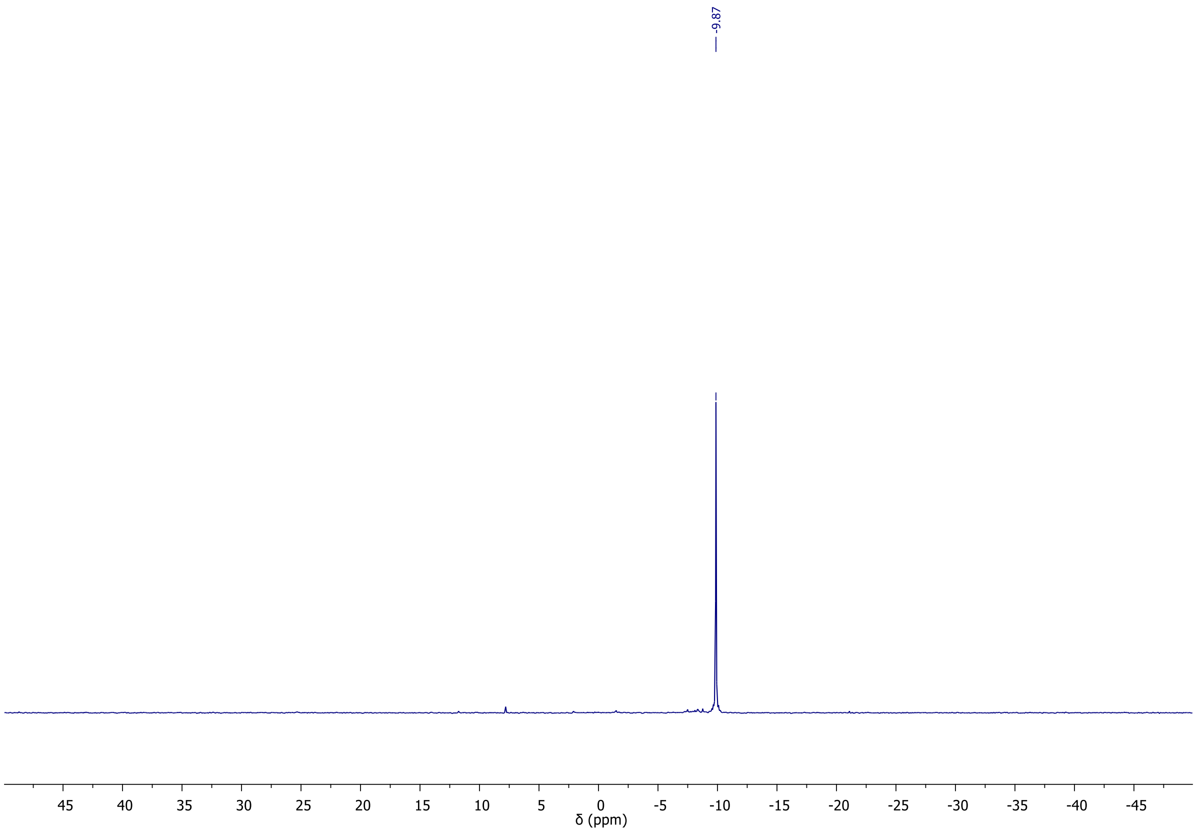

Figure S94: $^{31}P\{^1H\}$ NMR spectrum of **4P** (242.9 MHz, Tol-d$_8$, 213 K).

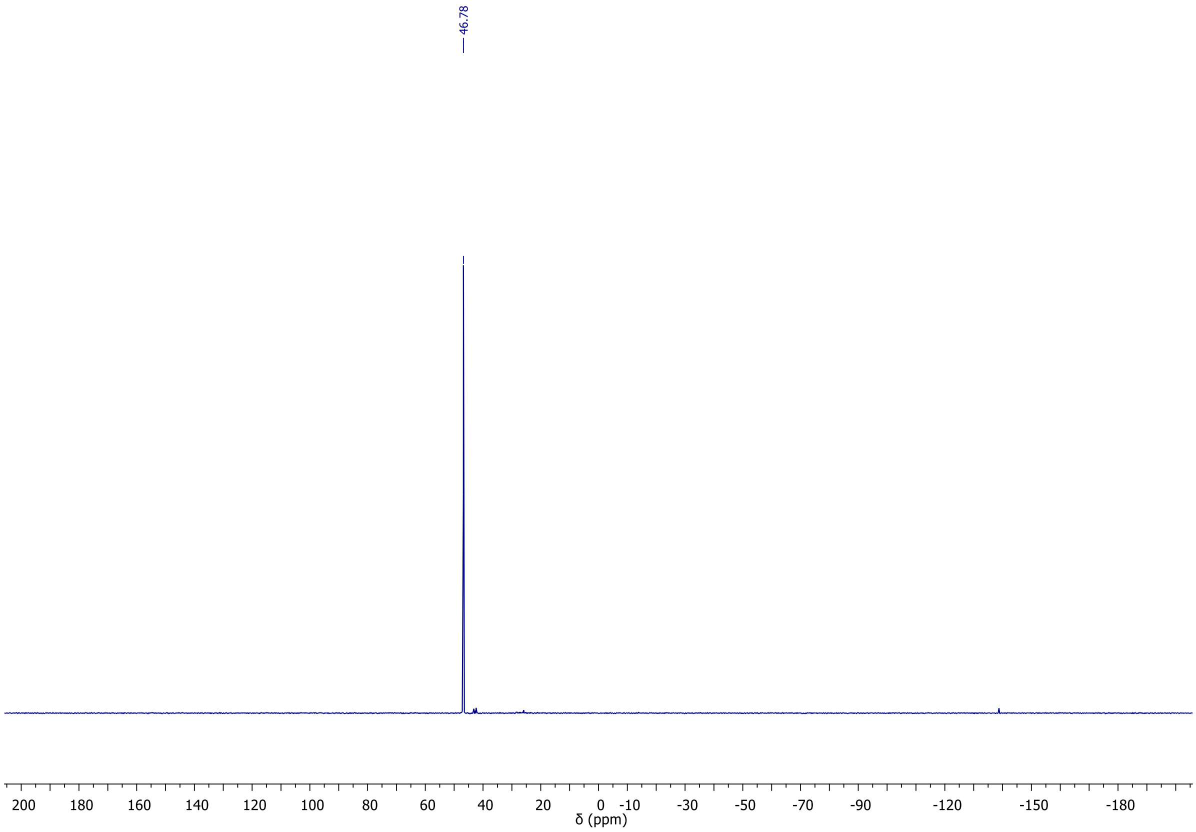

Figure S95: LIFDI mass spectrum of **4P** (toluene).

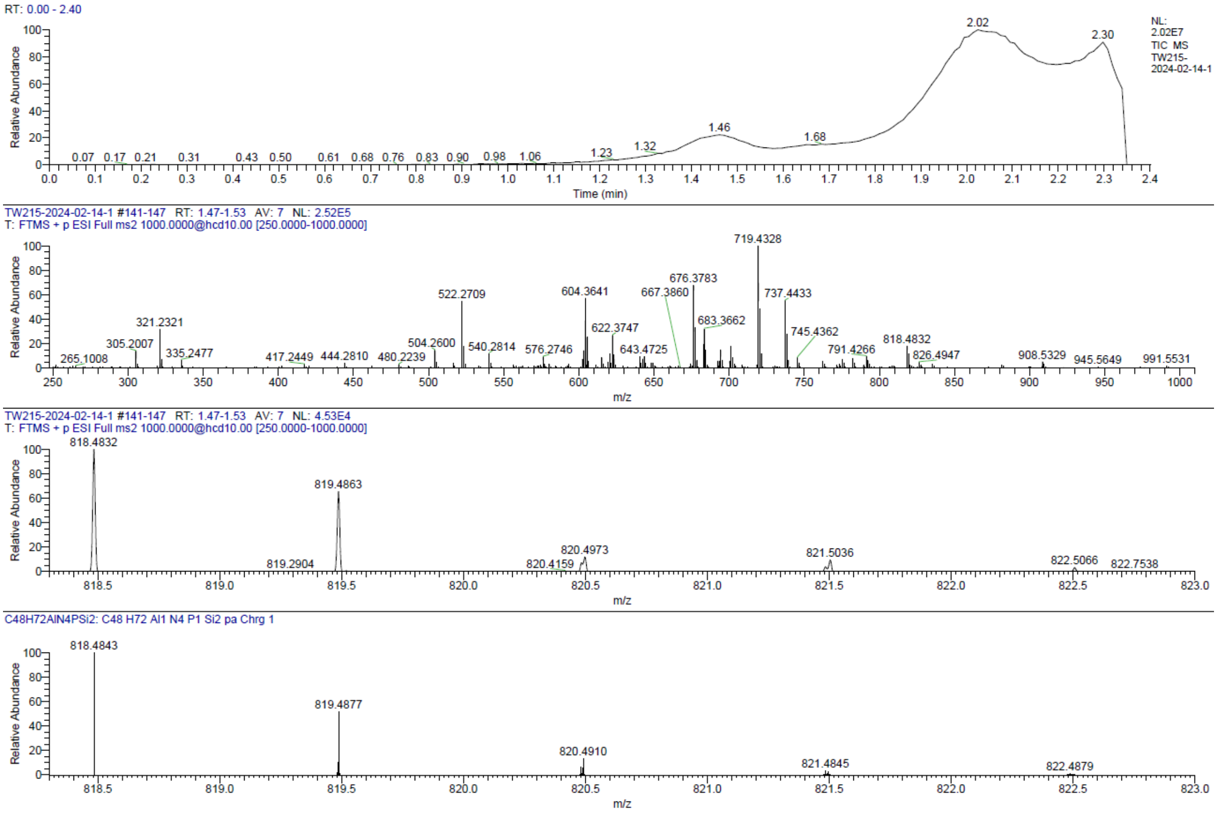

4.13 Compound 4As by reaction of ^{Dipp}TerAsAlCp* with (trimethylsilyl)diazomethane

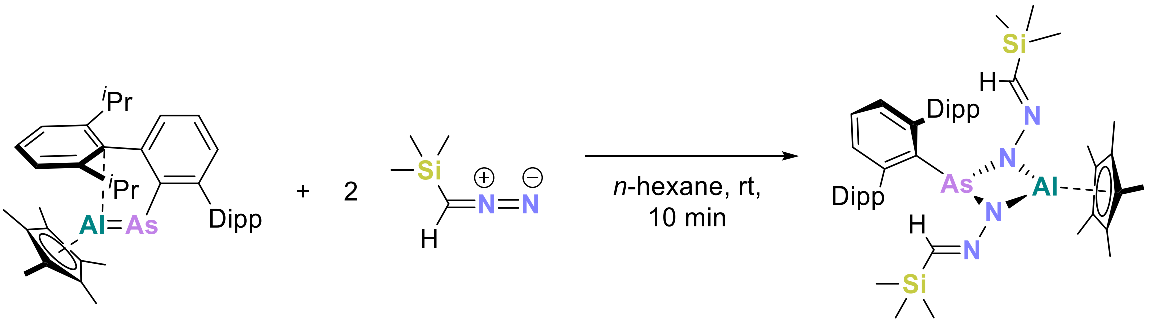

4As (59%)

^{Dipp}TerAsAlCp* (50.0 mg, 78.8 μmol, 1.00 eq.) was suspended in *n*-hexane (2.5 mL) in a Schlenk flask. A solution of (trimethylsilyl)diazomethane (0.6 M in *n*-hexane, 0.29 mL, 174.0 μmol, 2.21 eq.) was added over a period of 1 min at ambient temperature. The reaction mixture was stirred for 10 min until a colour change from blue to yellow occurred. The clear yellow solution was concentrated to ~0.5 mL *in vacuo* and was allowed to cool down from 45 °C to ambient temperature (in an oil bath) over night to get colourless crystals. The supernatant was removed by syringe and the crystals were washed with cold (−78 °C) *n*-hexane (0.3 ml). Supernatant and washing solution were combined to get a second fraction of product analogous to the procedure described above. The isolated crystalline fractions were dried for 30 min at ambient temperature at 1×10^{-3} mbar.

Yield of 4As: 40.1 mg (46.3 μmol, 59%) of a colourless, crystalline solid.

Single crystals suitable for X-ray diffraction were grown as described in the synthetic procedure.

^{1}H NMR (600.2 MHz, Tol-d_8, 213 K): δ = 0.30 (s, 18 H, $N_2CHSi(C\mathit{H}_3)_3$), 1.05 (d, $^3J_{(H\text{-}H)}$ = 6.7 Hz, 6 H, $CH(C\mathit{H}_3)(CH_3)$ (group A)[xxix]), 1.12 (d, $^3J_{(H\text{-}H)}$ = 6.6 Hz, 6 H, $CH(C\mathit{H}_3)(CH_3)$ (group B)), 1.32 (d, $^3J_{(H\text{-}H)}$ = 6.8 Hz, 6 H, $CH(CH_3)(C\mathit{H}_3)$ (group B)), 1.40 (d, $^3J_{(H\text{-}H)}$ = 6.5 Hz, 6 H, $CH(CH_3)(C\mathit{H}_3)$ (group A)), 1.87 (s, 15 H, $C_5(C\mathit{H}_3)_5$), 2.77 (ψ-hept, $^3J_{(H\text{-}H)}$ = 6.7 Hz, 2 H, $C\mathit{H}(CH_3)(CH_3)$ (group B)), 2.82 (ψ-hept, $^3J_{(H\text{-}H)}$ = 6.6 Hz, 2 H, $C\mathit{H}(CH_3)(CH_3)$ (group A)), 6.68 (s, 2 H, $N_2C\mathit{H}Si(CH_3)_3$), 6.86 (dd, $^3J_{(H\text{-}H)}$ = 7.6 Hz, $^4J_{(H\text{-}H)}$ = 1.1 Hz, 1 H, *m-H* (^{Dipp}Ter)), 6.91 (t, $^3J_{(H\text{-}H)}$ = 7.6 Hz, 1 H, *p-H* (^{Dipp}Ter)[xxx]), 7.06 (dd, $^3J_{(H\text{-}H)}$ = 7.6 Hz, $^4J_{(H\text{-}H)}$ = 1.1 Hz, 1 H, *m-H* (^{Dipp}Ter))[xxxi], 7.09 (d, $^3J_{(H\text{-}H)}$ = 7.7 Hz, 2 H, *m-H* (Dipp) (group C)), 7.10 (d, $^3J_{(H\text{-}H)}$ = 7.6 Hz, 2 H, *m-H* (Dipp) (group D)), 7.18 (t, $^3J_{(H\text{-}H)}$ = 7.6 Hz, 1 H, *p-H* (Dipp) (group D)), 7.32 (t, $^3J_{(H\text{-}H)}$ = 7.7 Hz, 1 H, *p-H* (Dipp) (group C)) ppm. **^{13}C{^{1}H} NMR** (150.9 MHz, Tol-d_8, 213 K): δ = –0.6 (s, $Si(\mathit{C}H_3)_3$), 10.2 (s, $C_5(\mathit{C}H_3)_5$)), 22.9 (s, $CH(CH_3)(\mathit{C}H_3)$ (group B)), 23.7 (s, $CH(CH_3)(\mathit{C}H_3)$ (group A)), 26.0 (s, $CH(\mathit{C}H_3)(CH_3)$ (group A)), 26.8 (s, $CH(\mathit{C}H_3)(CH_3)$ (group B)), 30.4 (s, $\mathit{C}H(CH_3)(CH_3)$ (group A)), 31.4 (s, $\mathit{C}H(CH_3)(CH_3)$ (group B)), 114.0 (s, $\mathit{C}_5(CH_3)_5$)), 121.7 (s, *m*-CH (Dipp) (group D)), 122.9 (s, *m*-CH (Dipp) (group C)), 127.6 (s, *p-H* (^{Dipp}Ter)), 128.4 (s, *p*-CH (Dipp) (group C)), 128.6 (s, *p*-CH (Dipp) (group D))[xxxii], 129.5 (s, *m*-CH (^{Dipp}Ter)), 133.0 (s, *m*-CH (^{Dipp}Ter)), 138.5 (s, *i*-C (Dipp) (group D)), 139.4 (s, *i*-C (Dipp) (group C)), 139.5 (s, $N_2CHSi(CH_3)_3$), 143.9 (s, *o-C* (^{Dipp}Ter)), 144.5 (s, *i-C* (^{Dipp}Ter)), 146.40 (s, *o-C* (^{Dipp}Ter)), 147.0 (s, *o*-C (Dipp) (group C)), 147.2 (s, *o*-C (Dipp) (group D)) ppm. **^{29}Si{^{1}H} NMR** (119.2 MHz, Tol-d_8, 213 K): δ = −9.9 (s) ppm. **IR** (ATR, 32 scans, cm^{-1}): $\tilde{\nu}$ = 3265 (vw), 3057 (vw), 2958 (m), 2927 (w), 2906 (w), 2867 (w), 1663 (vw), 1548 (w), 1525 (w), 1501 (w), 1459 (w), 1443 (w), 1381 (m), 1362 (w), 1325 (w), 1294 (w), 1276 (w), 1245 (m), 1177 (w), 1160 (w), 1134 (w), 1101 (w), 1055 (m), 1043 (m), 985 (w), 954 (w), 934 (w), 868 (s), 835 (vs), 806 (s), 791 (s), 759 (s), 746 (s), 690 (s), 658 (s), 616 (m), 585 (m), 557 (m), 501 (s), 480 (m), 464 (m), 406 (m). **LIFDI-MS**: (m/z) calc: 862.4326 [$C_{48}H_{72}AlAsN_4Si_2$]; (m/z)

[xxix] *Note*: Due to the rotational hindrance of the bulky TMS and Dipp substituents, the NMR experiment was conducted at low temperature (213 K) to prevent signal broadening due to wobbeling. To assign all resonances that belong to one *i*Pr group the labels A and B and a similar differentiation for the resonances of the ^{Dipp}Ter ligand C and D substituent in the aromatic region were used.

[xxx] Superimposed resonances are a double duplet (dd), which appears as triplet (t).

[xxxi] Superimposed with Tol-d_8 resonance. Assigned with ^{1}H/^{1}H COSY NMR and ^{1}H/^{13}C HSQC NMR spectra.

[xxxii] Superimposed with Tol-d_8 resonance. Assigned with ^{1}H/^{13}C HSQC NMR spectrum.

found: 602.2897 (M^+–(AlCp*)–(TMSC(H)N)]). **CHN** calc. (found) in %: C 66.79 (64.72), H 8.41 (8.16), N 6.49 (6.30).[xxxiii]

Figure S96: ^{1}H NMR spectrum of **4As** (600.2 MHz, Tol-d_8, 213 K).

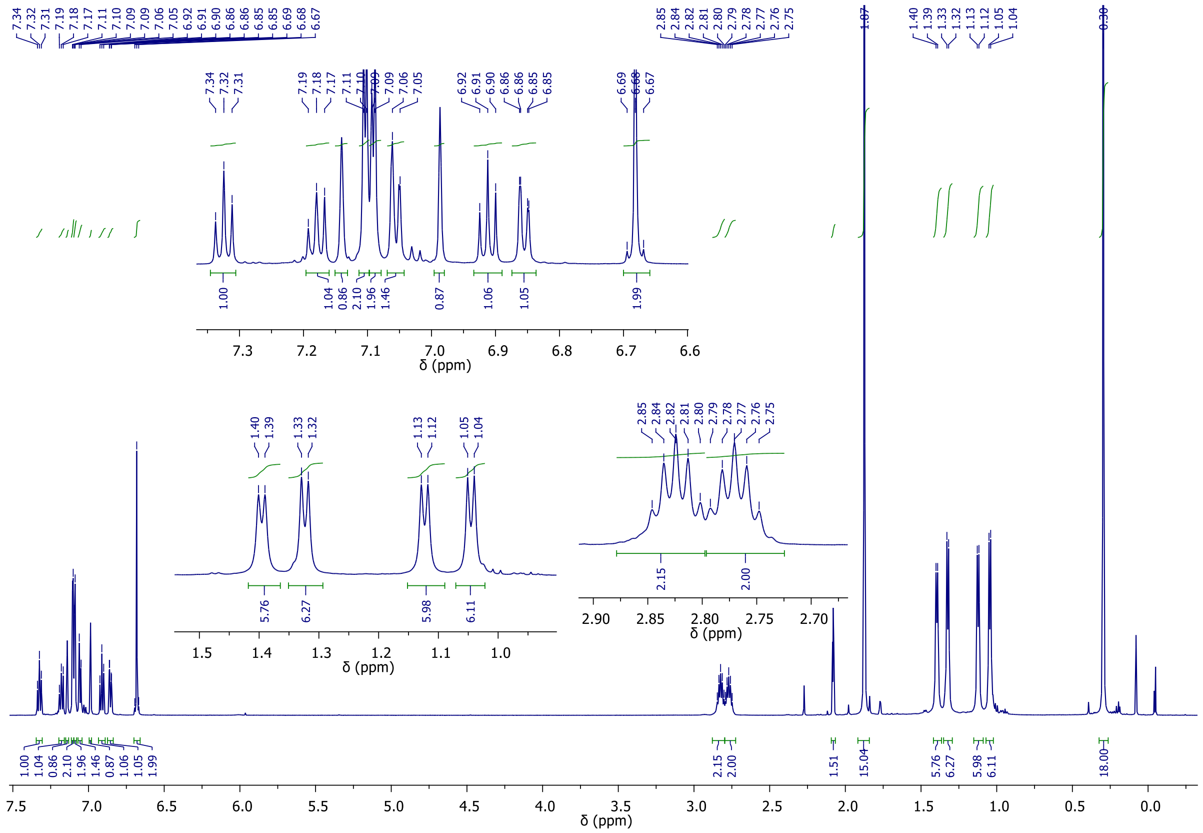

[xxxiii] Deviations likely due to incomplete combustion, repeated measurements without oxidizing agents did not give better results.

Figure S97: $^{13}C\{^{1}H\}$ NMR spectrum of **4As** (150.9 MHz, Tol-d$_8$, 213 K).

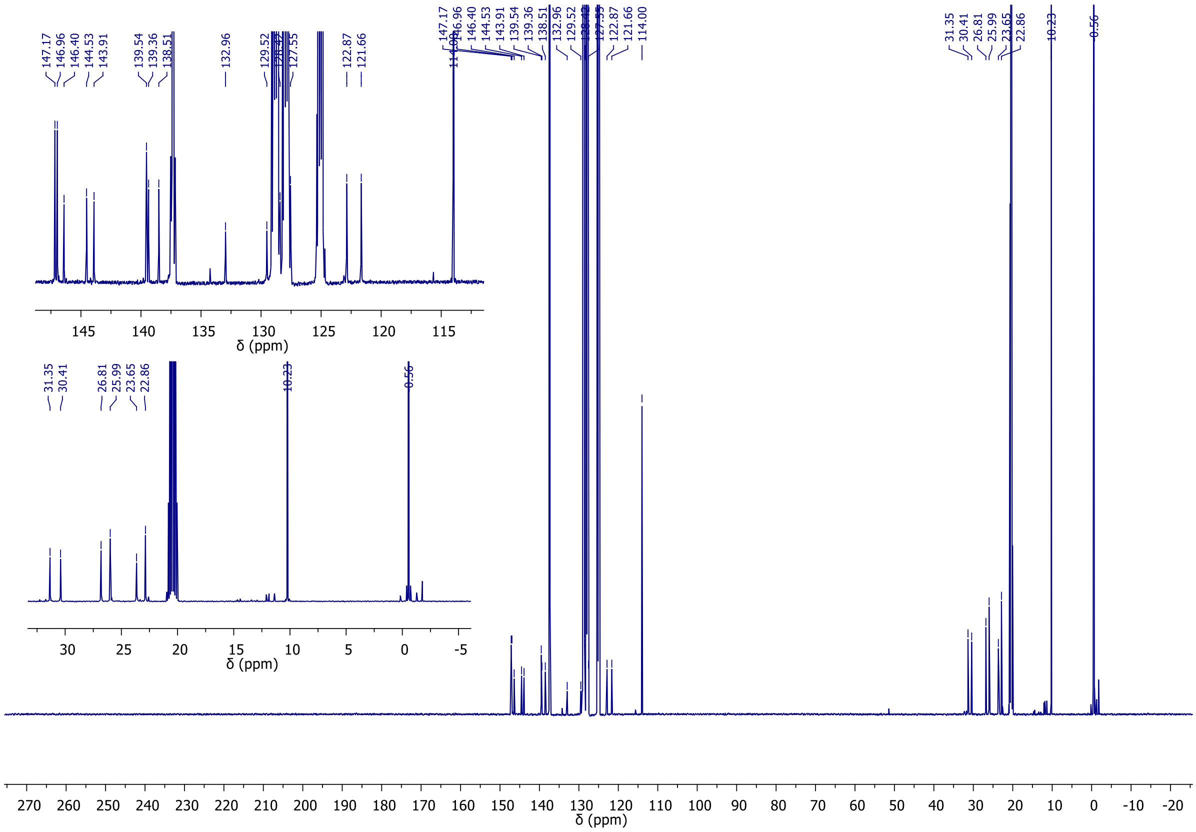

Figure S98: ^{29}Si NMR spectrum of **4As** (119.2 MHz, Tol-d$_8$, 213 K).

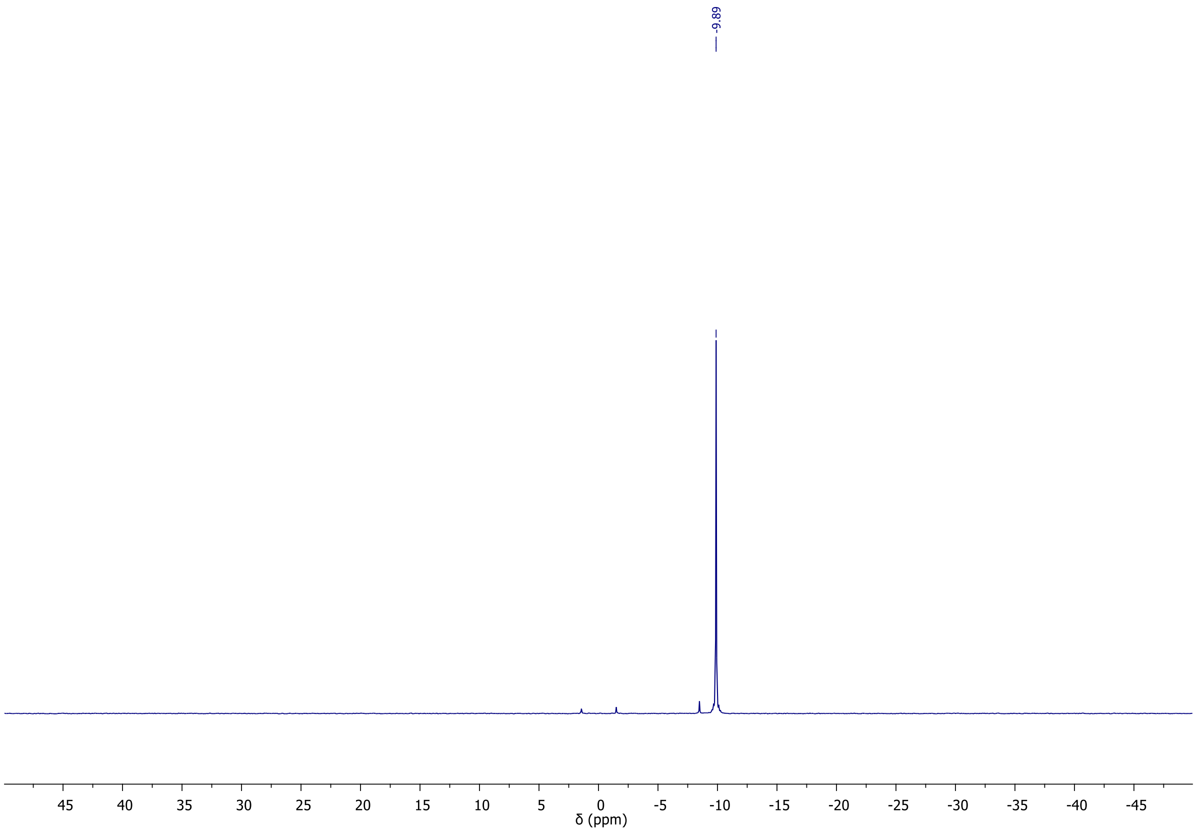

Figure S99: IR spectrum of **4As** (ATR, 32 scans, cm^{-1}, powder).

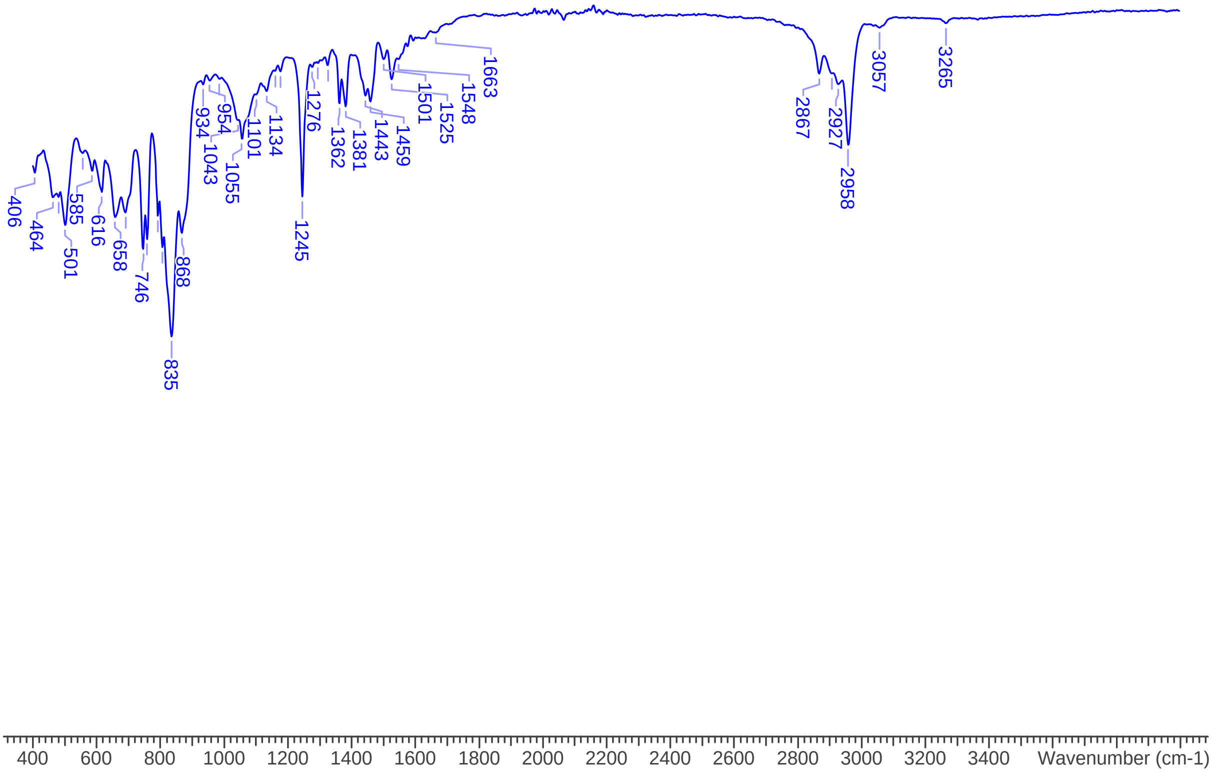

Figure S100: LIFDI mass spectrum of **4As** (toluene).

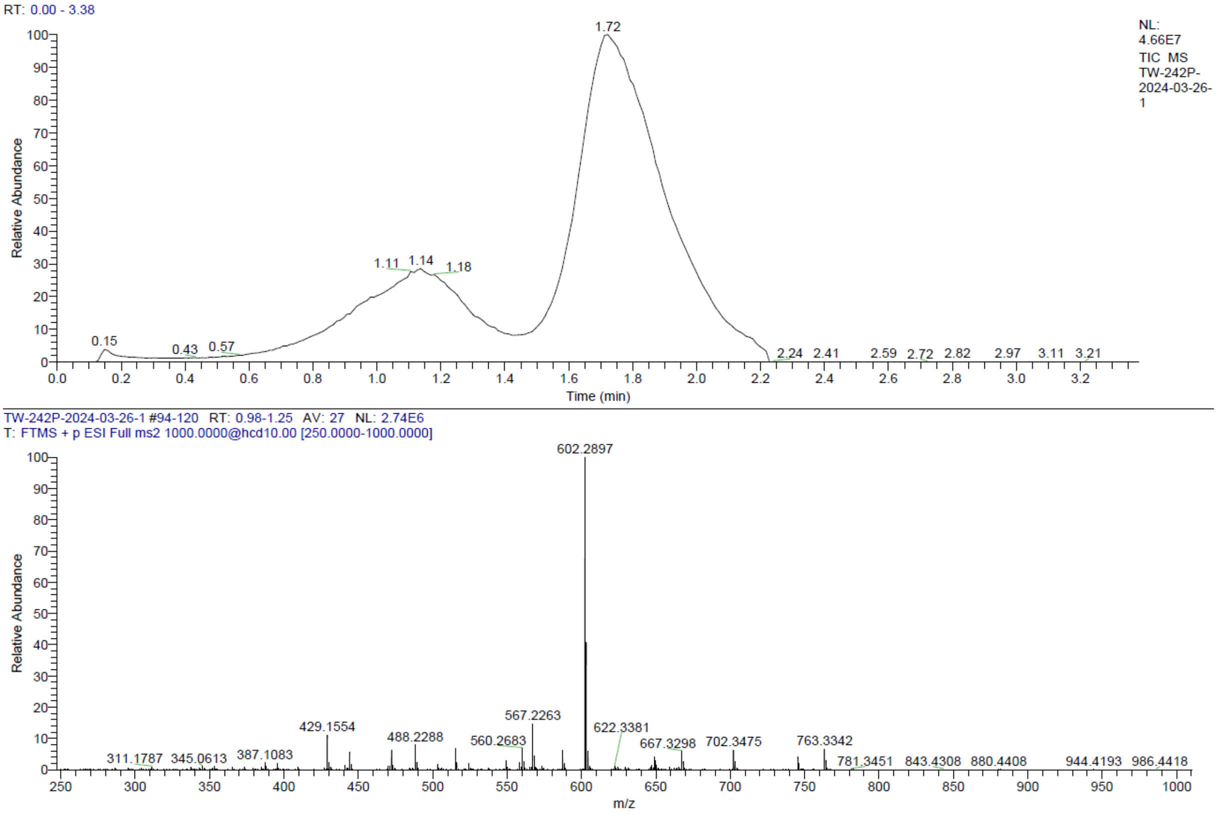

4.14 Thermal / Photochemical rearrangement of compound 4P to 5P

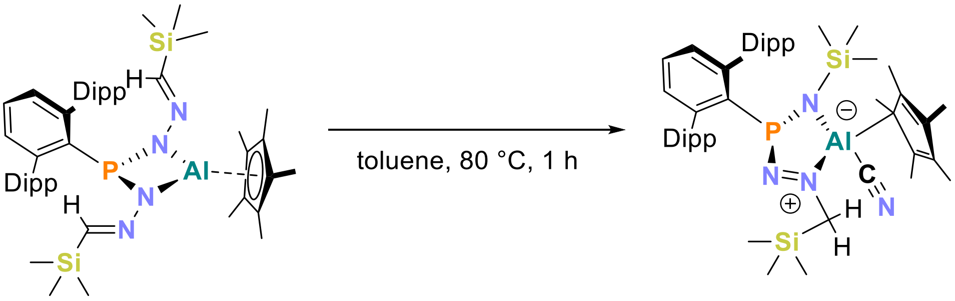

5P (41%)

<u>Method 1</u>

Compound **4P** (11.0 mg, 13.4 µmol, 1.00 eq.) was dissolved in toluene-d8 (0.4 mL) in a *J.-Young* NMR tube. The pale-yellow solution was heated for 1.5 h at 80 °C (heating block) and during heating, a colour change to bright red was observed. The solvent was removed *in vacuo* (~1×10^{-3} mbar) and the oily residue was re-dissolved in *n*-pentane (0.4 mL). Immediately upon addition of *n*-pentane to the oil, crystallization of **5P** in form of red blocks took place. No yield was determined for this method.

Suitable crystals for single crystal X-ray diffraction were obtained from a saturated *n*-pentane solution of **5P** ambient temperature.

<u>Method 2</u>

Compound **4P** (15.7 mg, 19.2 µmol, 1.00 eq.) was dissolved in toluene-d8 (0.4 mL) in a *J.-Young* NMR tube. The pale-yellow solution was irradiated by a Hg/Xe UV lamp (I = 19 A, U = 26 V, P = 500 W, λ = 210–600 nm) for 3 h at ambient temperature (distance between lamp and *J.-Young* NMR tube is 25 cm to minimalize thermal contributions). After 3 h, the solution had turned bright red and partial conversion of **4P** to compound **5P** was confirmed via $^{31}P\{^1H\}$ NMR spectroscopy. No yield was determined for this method.

Method 3

^{Dipp}TerPAlCp* (40.0 mg, 67.8 μmol, 1.00 eq.) was dissolved in *n*-hexane (0.5 mL) in a *J.-Young* NMR tube and a solution of (trimethylsilyl)diazomethane (0.6 M in *n*-hexane, 0.24 ml, 144 μmol, 2.10 eq.) was added at ambient temperature. Upon addition, the colour of the reaction mixture instantly changed from deep purple to pale-yellow. All volatile components were removed *in vacuo* ($\sim 1\times10^{-3}$ mbar) at ambient temperature and the pale-yellow oil was re-dissolved in toluene (0.5 mL). For the thermal rearrangement, the pale-yellow solution of **4P** was heated at 80 °C (heating block) for 1.5 h and the colour of the reaction mixture changed to bright red. Full conversion of **4P** to compound **5P** was confirmed via $^{31}P\{^{1}H\}$ NMR spectroscopy. All volatile components were removed *in vacuo* ($\sim 1\times10^{-3}$ mbar) at ambient temperature. The red oil was re-dissolved in a mixture of *n*-pentane (0.5 mL) and HMDSO (0.05 mL). The *n*-pentane (0.5 mL) / HMDSO (0.05 mL) mixture was placed in a small vial and the solvent was slowly removed by placing the small vial into a closed, bigger vial containing silicon grease. The solution was stored at −30 °C for 48 h until crystallization of **5P**. The remaining supernatant was removed with a glass pipette and the solid was carefully washed with cold (–30 °C) *n*-pentane (2 × 0.1 mL) and dried by evaporation of the remaining solvent at ambient temperature in a glovebox.

Yield of 5P: 23.1 mg (28.1 μmol, 41%) of a red crystalline solid.[xxxiv]

[xxxiv] *Note*: Several attempts to isolate crystals of **5P** always yielded a mixture of two species. This was indicated by two $^{31}P\{^{1}H\}$ NMR resonances at 137.5 ppm and 136.8 ppm (298 K, toluene-d_8), with a ratio of approx. 80:20. Also, in the ^{1}H NMR spectrum (298 K, toluene-d_8) two distinct resonance sets were observed with the same ratio. A ^{1}H DOSY NMR experiment was performed to rule out a co-crystallized impurity and confirm that the second species is the isocyanide form of **5P** (**5P_NC**). For the major species (80%), an average diffusions coefficient of 7.086×10^{-10} m^2/s and for the minor species, an average diffusion coefficient of 7.161×10^{-10} m^2/s was found, indicating the same molecular weight for both species. Thus, the resonance sets could likely belong to an isomerisation of the CN/NC substituent at the Al atom. Additionally, it was observed, that due to the temperature-related shift of the resonances in the $^{31}P\{^{1}H\}$ NMR and prevented rotation of the substituents the minor species is more difficult to identify.

^{1}H NMR (600.2 MHz, Tol-d$_8$, 213 K): δ = 0.08 (s, 9 H, $N(CH_2)Si((CH_3)_3)$), 0.31 (s, 9 H, $NSi((CH_3)_3)$), 0.73 (d, $^{3}J_{(H\text{-}H)}$ = 6.5 Hz, 3 H, $CH(CH_3)(CH_3)$ (group A)), 0.96 (d, $^{3}J_{(H\text{-}H)}$ = 6.5 Hz, 3 H, $CH(CH_3)(CH_3)$ (group B)), 1.14 (d, $^{3}J_{(H\text{-}H)}$ = 6.7 Hz, 3 H, $CH(CH_3)(CH_3)$ (group C)), 1.15 (d, $^{3}J_{(H\text{-}H)}$ = 6.6 Hz, 3 H, $CH(CH_3)(CH_3)$ (group D)), 1.19 (d, $^{3}J_{(H\text{-}H)}$ = 6.5 Hz, 3 H, $CH(CH_3)(CH_3)$ (group A)), 1.31 (d, $^{3}J_{(H\text{-}H)}$ = 6.6 Hz, 3 H, $CH(CH_3)(CH_3)$ (group D)), 1.38 (d, $^{3}J_{(H\text{-}H)}$ = 6.7 Hz, 3 H, $CH(CH_3)(CH_3)$ (group C)), 1.42 (d, $^{3}J_{(H\text{-}H)}$ = 6.5 Hz, 3 H, $CH(CH_3)(CH_3)$ (group B)), 1.84 (s, 15 H, $C_5(CH_3)_5$), 2.86 (hept, $^{3}J_{(H\text{-}H)}$ = 6.5 Hz, 1 H, $CH(CH_3)(CH_3)$ (group A)), 2.91 (hept, $^{3}J_{(H\text{-}H)}$ = 6.5 Hz, 1H, $CH(CH_3)(CH_3)$ (group B)), 3.05 (overlap hept, 2 H, $CH(CH_3)(CH_3)$ (group D + C)), 3.83 (dd, $J_{(H\text{-}P)}$ = 28.4 Hz, $^{1}J_{(H\text{-}H)}$ = 11.5 Hz, 2 H, N_2CH)[xxxv], 4.45 (dd, $J_{(H\text{-}P)}$ = 20.2 Hz, $^{1}J_{(H\text{-}H)}$ = 11.5 Hz, 1H, N_2CH),‡ 6.92 (d, $^{3}J_{(H\text{-}H)}$ = 7.7 Hz, 1 H, *m-H* (^{Dipp}Ter)), 6.95 (superimposed signals, 2 H, *m-H* (Dipp) (group E)+ *m-H* (^{Dipp}Ter)), 6.97 – 7.00 (m, 1 H, *m-H* (Dipp) (group E))[xxxvi], 7.04 – 7.09 (superimposed signals, 2 H, *m-H* (Dipp) (group F) and *p-H* (^{Dipp}Ter))[xxxvi], 7.12 – 7.16 (m, 1 H, *m-H* (Dipp) (group F))[xxxvi], 7.27 (t, $^{3}J_{(H\text{-}H)}$ = 7.7 Hz, 1 H, *p-H* (Dipp) (group F)), 7.30 – 7.34 (m, 1H, *p-H* (Dipp) (group E))[xxxv] ppm. **^{13}C{^{1}H} NMR** (150.9 MHz, Tol-d$_8$, 213 K): δ = −0.4 (s, $N(CH_2)Si((CH_3)_3)$), 3.35 (d, $J_{(C\text{-}P)}$ = 6.4 Hz, $NSi((CH_3)_3)$), 12.9 (s, $C_5(CH_3)_5$, 22.1 (s, $CH(CH_3)(CH_3)$ (group A)), 22.1 (s, $CH(CH_3)(CH_3)$ (group B)), 22.9 (s, $CH(CH_3)(CH_3)$ (group D)), 24.6 (s, $CH(CH_3)(CH_3)$ (group C)), 25.2 (s, $CH(CH_3)(CH_3)$ (group A)), 26.1 (s, $CH(CH_3)(CH_3)$ (group B)), 26.7 (s, $CH(CH_3)(CH_3)$ (group C)), 27.3 (s, $CH(CH_3)(CH_3)$ (group D)), 30.1 (s, $CH(CH_3)(CH_3)$ (group A)), 30.8 (s, $CH(CH_3)(CH_3)$ (group B)), 31.3 (s, $CH(CH_3)(CH_3)$ (group C)), 31.5 (s, $CH(CH_3)(CH_3)$ (group D)), 60.6 (d, $J_{(C\text{-}P)}$ = 8.4 Hz, N_2CH), 119.2 (s, $C_5(CH_3)_5$), 122.4 (s, *C*H), 123.0 (s, *C*H), 123.9 (s, *C*H), 125.0 (s, *C*H)[xxxvii], 128.9 (s, *C*H)[xxxvii], 129.0 (s, *C*H),129.3 (s, *C*H), 131.15 (d, $J_{(C\text{-}P)}$ = 10.0 Hz, *C*H),134.0 (s, *C*H), 135.2 (s, C_q), 135.6 (s, C_q), 138.9 (d, $J_{(C\text{-}P)}$ = 11.2 Hz, C_q), 139.4 (s, C_q), 143.7 (s, C_q), 144.9 (s, C_q), 145.4 (s, C_q), 145.7 (s, C_q), 147.00 (bs, C_q), 148.6 (s, C_q), 148.9 (d, $J_{(C\text{-}P)}$ = 52.5 Hz, *i-C* (^{Dipp}Ter)) ppm. **^{29}Si{^{1}H} NMR** (119.2 MHz, Tol-d$_8$, 213 K): δ = 8.29 (d, $J_{(Si\text{-}P)}$ = 14.6 Hz, $NSi((CH_3)_3)$), 14.13 (d, $J_{(Si\text{-}P)}$ = 39.6 Hz, $N(CH_2)Si((CH_3)_3)$) ppm. **^{31}P{^{1}H} NMR** (243.0 MHz, Tol-d$_8$, 213 K): δ = 135.67 (s) ppm. **^{31}P{^{1}H} NMR** (243.0 MHz, Tol-d$_8$, 298 K): δ = 137.5 (s, major species), 136.8 (s, minor species) ppm. **^{31}P{^{1}H} NMR**

(162.0 MHz, C_6D_6, 298 K): δ = 140.2 (s, major species), 139.5 (s, minor species) ppm. **IR** (ATR, 32 scans, cm^{-1}): $\tilde{\nu}$ = 3065 (vw), 2960 (m), 2925 (w), 2908 (w), 2865 (w), 2261 (w, CN group), 1606 (vw), 1591 (vw), 1575 (w), 1560 (w), 1494 (vw), 1459 (w), 1445 (w), 1428 (w), 1381 (w), 1360 (w), 1303 (w), 1251 (m), 1177 (w), 1160 (w), 1146 (w), 1121 (w), 1111 (w), 1099 (w), 1078 (w), 1057 (w), 1035 (w), 1012 (w), 936 (w), 884 (s), 837 (vs), 806 (m), 796 (s), 750 (s), 728 (m), 693 (m), 658 (m), 602 (w), 585 (m), 554 (m), 532 (m), 493 (vs), 456 (s), 423 (m). **LIFDI-MS** (m/z) calc: 818.4848 (100%), 819.4882 (51.9%), 820.4915 (13.2%), [$C_{48}H_{72}AlN_4PSi_2$]; found: 818.4848, 819.4885, 820.4927 [$C_{48}H_{72}AlN_4PSi_2$]. **UV-Vis** (benzene, ambient temperature): λ = 479 nm (ε = 2405 L × mol^{-1} × cm^{-1}).

Figure S101: $^{31}P\{^1H\}$ NMR spectrum of **5P** (243.0 MHz, Tol-d_8, 298 K).

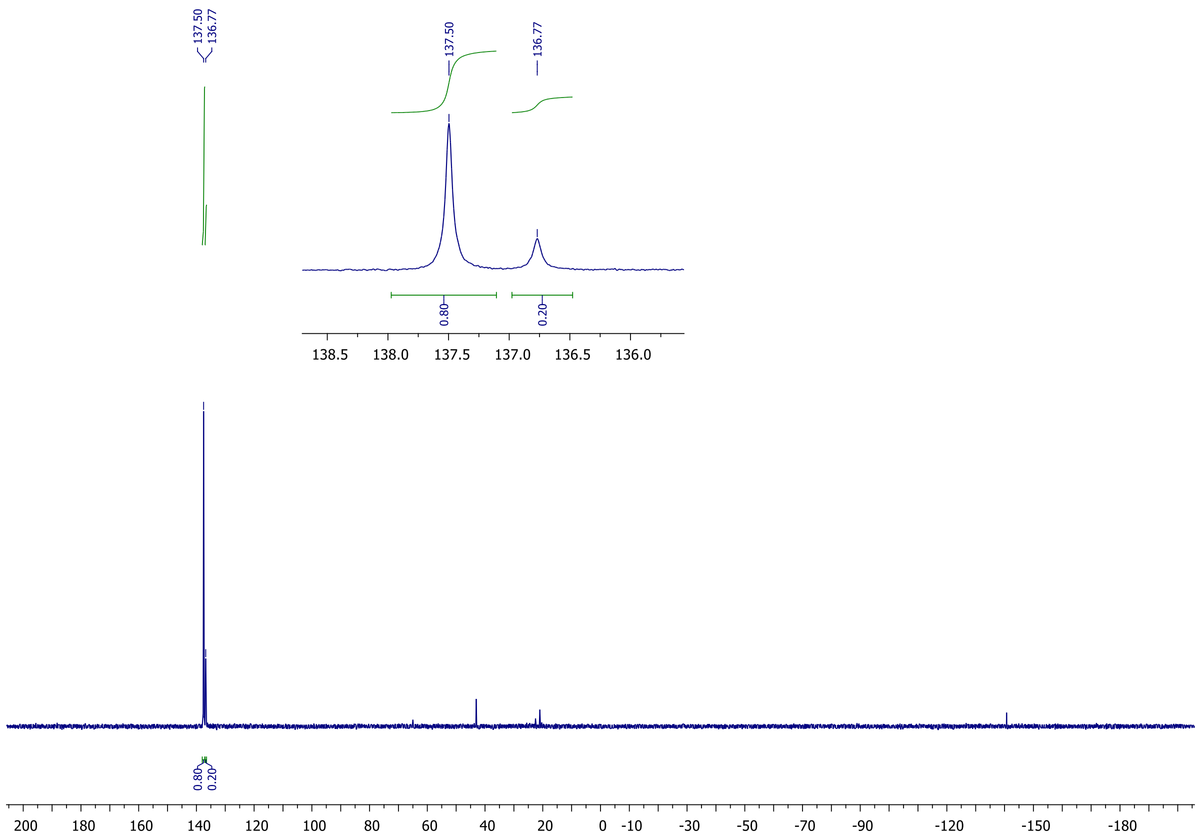

---

xxxv Appear as doublet (d) in $^1H\{^{31}P\}$ NMR spectrum.

xxxvi Superimposed with Tol-d_8 resonance. Assigned with $^1H/^1H$ COSY NMR spectrum.

xxxvii Superimposed with Tol-d_8 resonance. Assigned with $^1H/^{13}C$ HSQC NMR spectrum.

Figure S102: ^{1}H NMR spectrum of **5P** (600.2 MHz, Tol-d$_8$, 213 K).

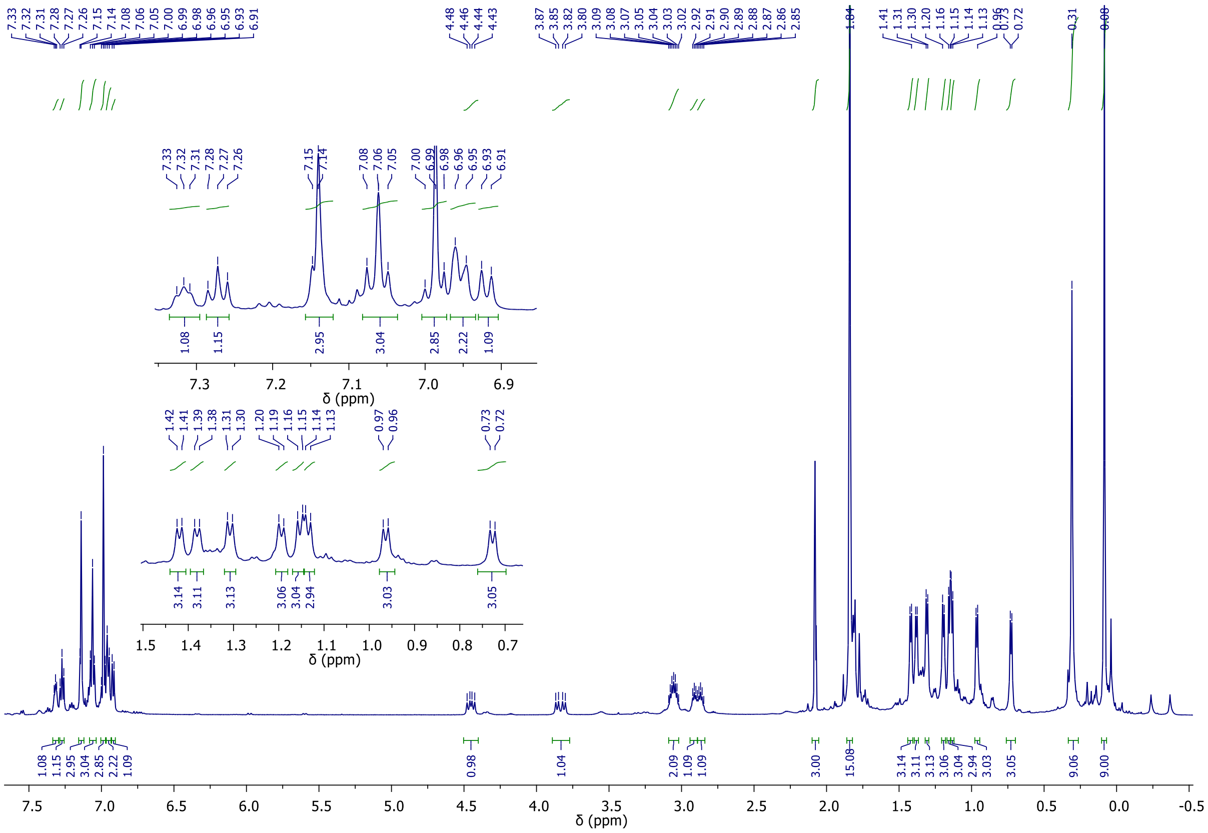

Figure S103: ^{13}C{^{1}H} NMR spectrum of **5P** (150.9 MHz, Tol-d$_8$, 213 K).

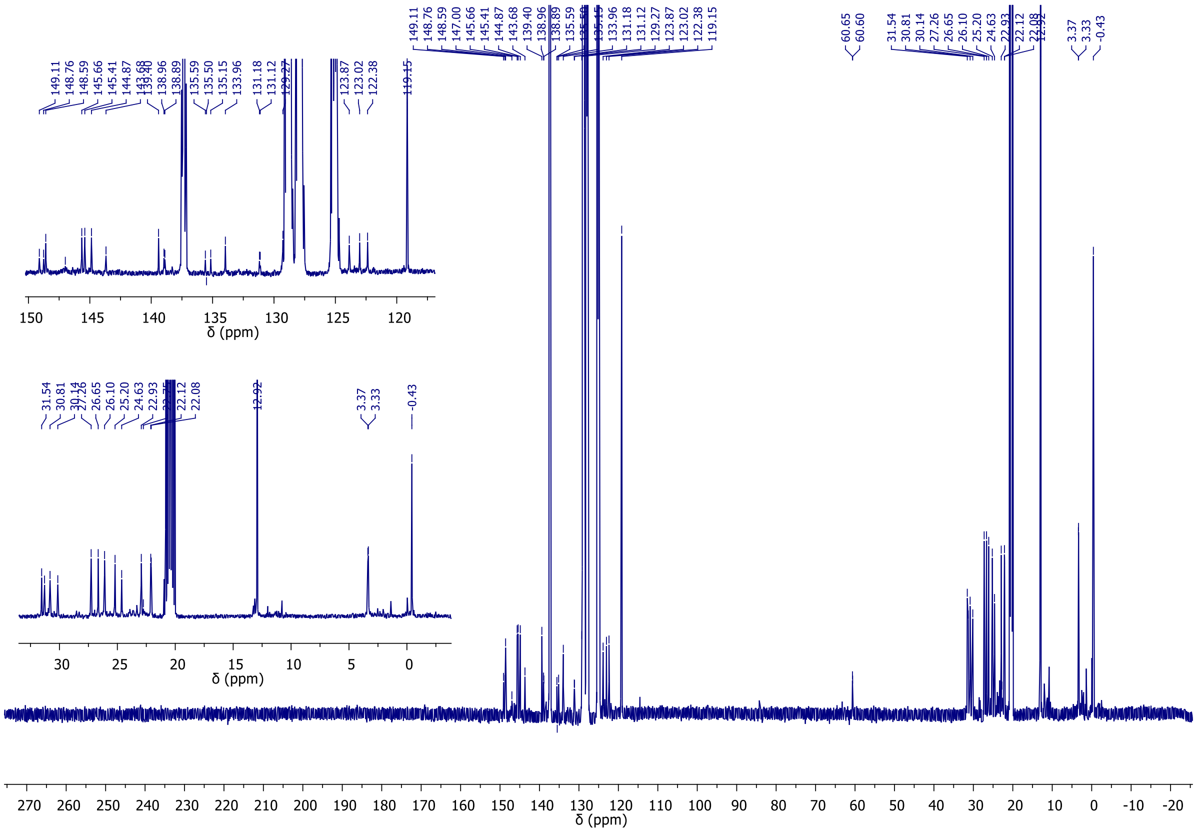

Figure S104: $^{29}Si\{^{1}H\}$ NMR spectrum of **5P** (119.2 MHz, Tol-d$_8$, 213 K).

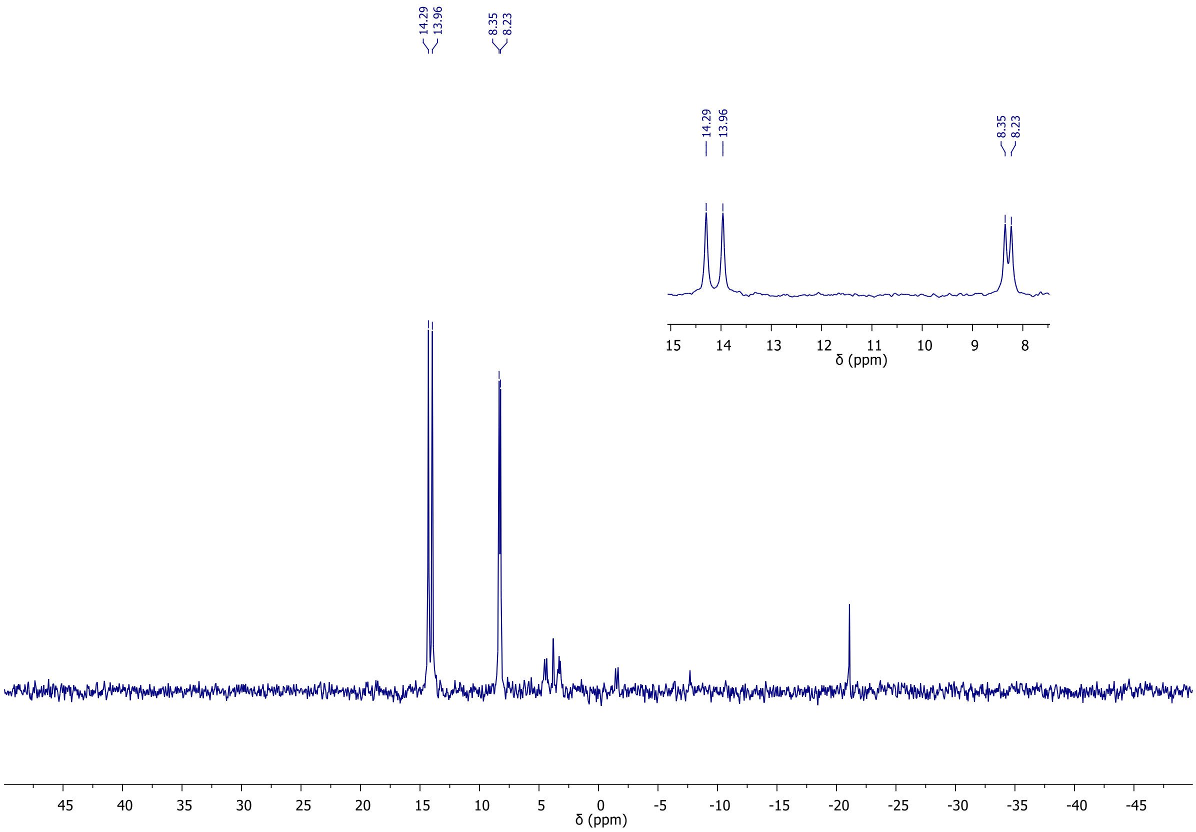

Figure S105: $^{31}P\{^{1}H\}$ NMR spectrum of **5P** (243.0 MHz, Tol-d$_8$, 213 K).

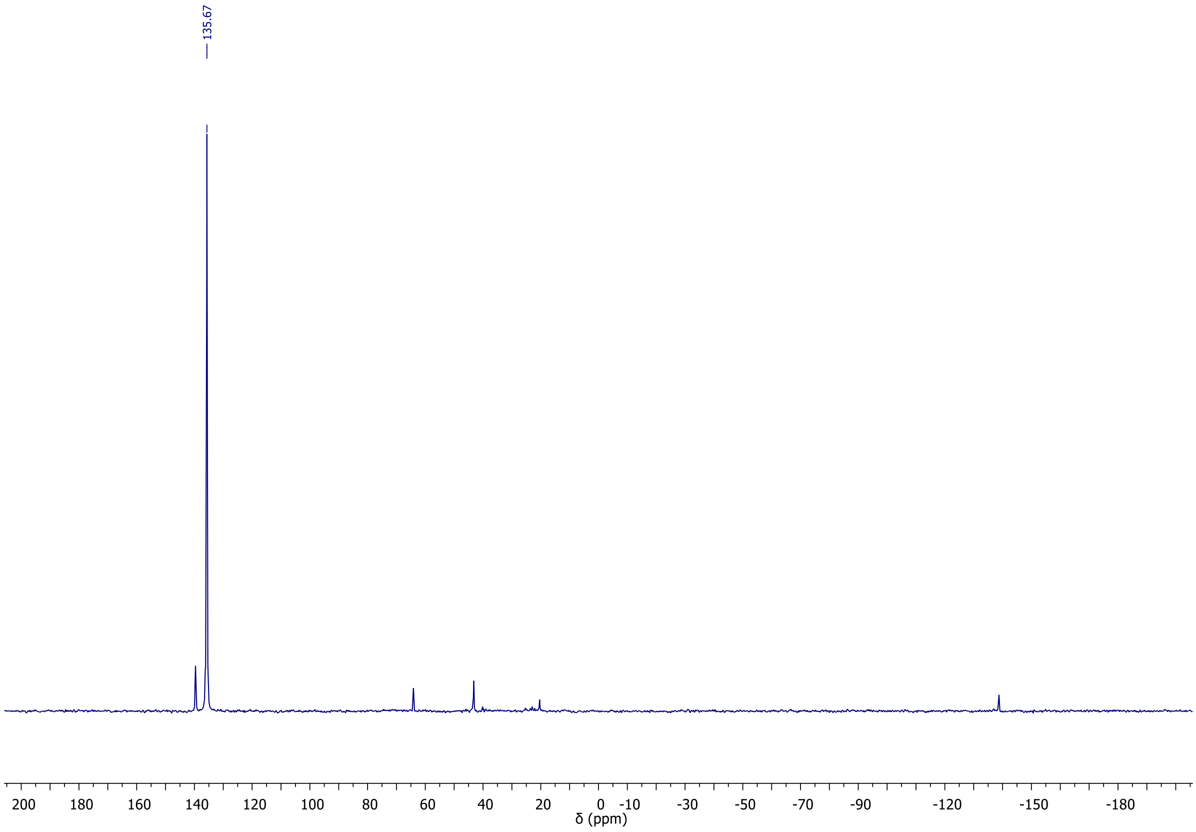

Figure S106: IR spectrum of **5P** (ATR, 32 scans, cm^{-1}, powder).

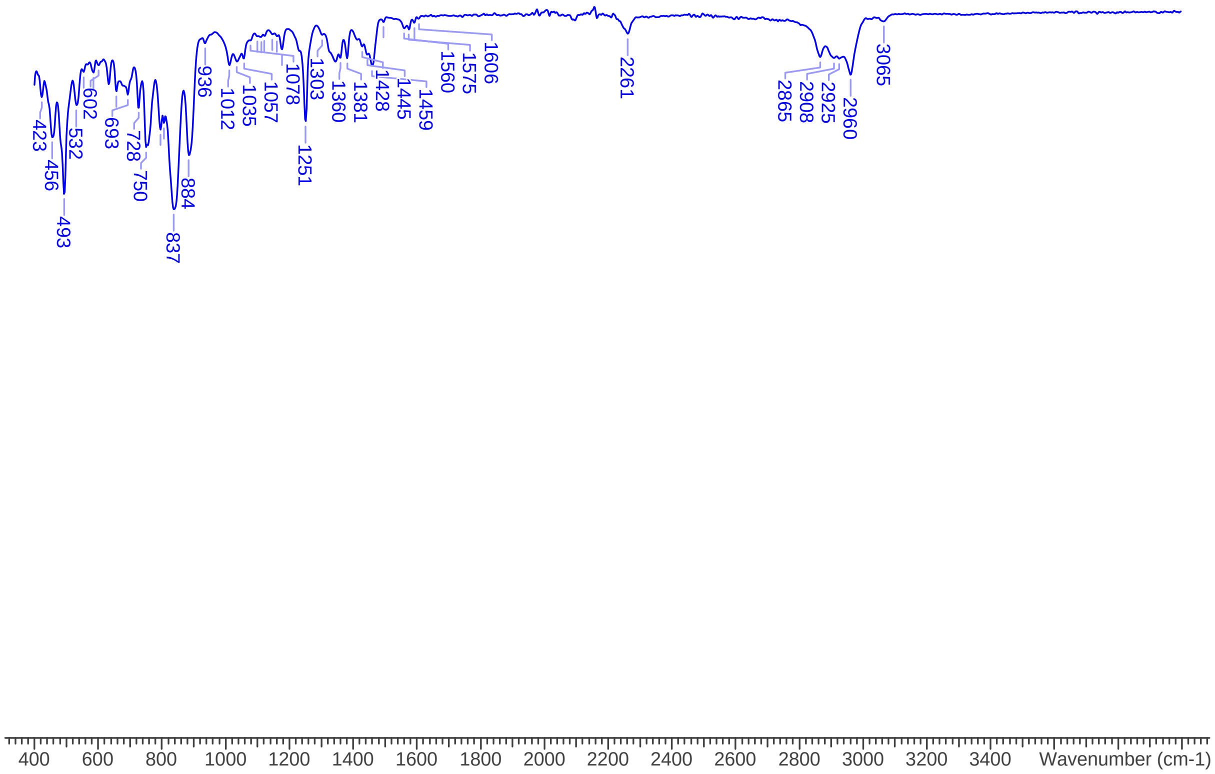

Figure S107: LIFDI mass spectrum of **5P** (toluene),

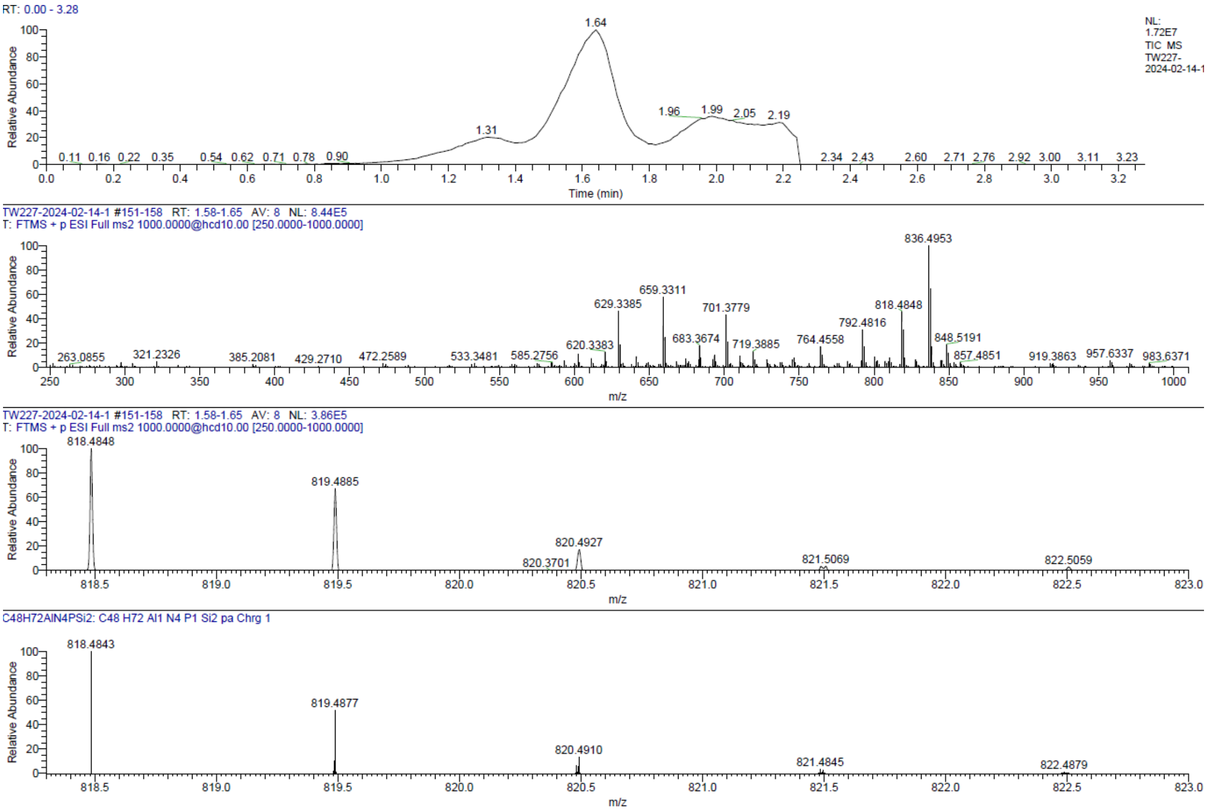

Figure S108: UV-vis absorption spectrum of **5P** (c = 1.34 × 10^{-4} mol/L, orange) in benzene at ambient temperature. Absorption maximum: λ = 479 nm.

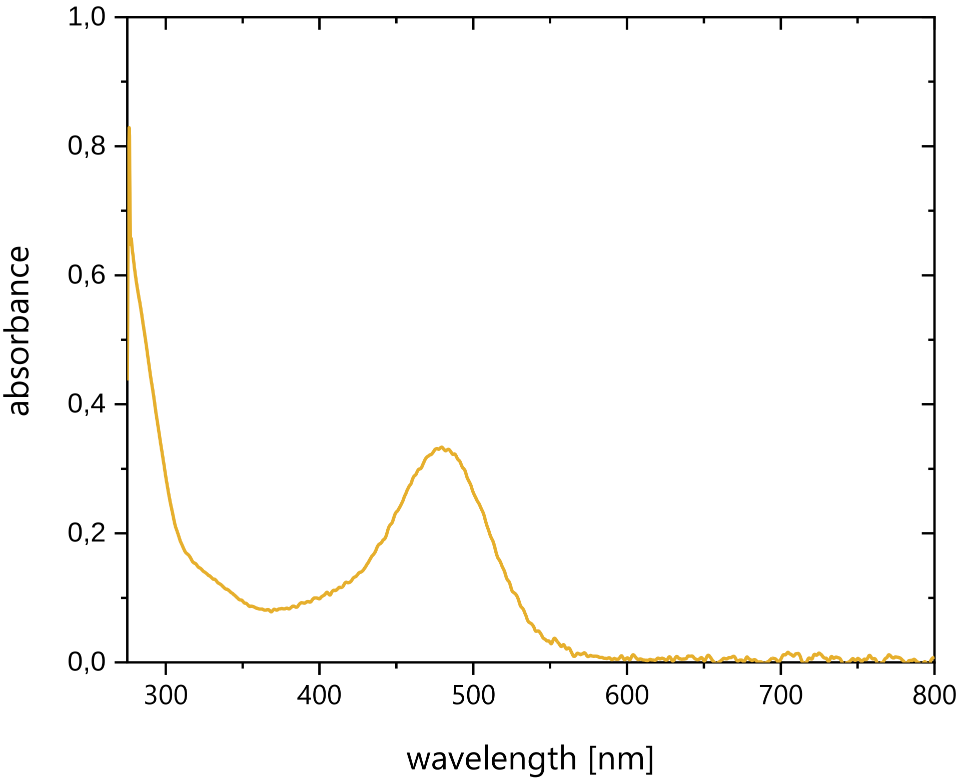

4.15 Thermal rearrangement of compound 5P to 6P

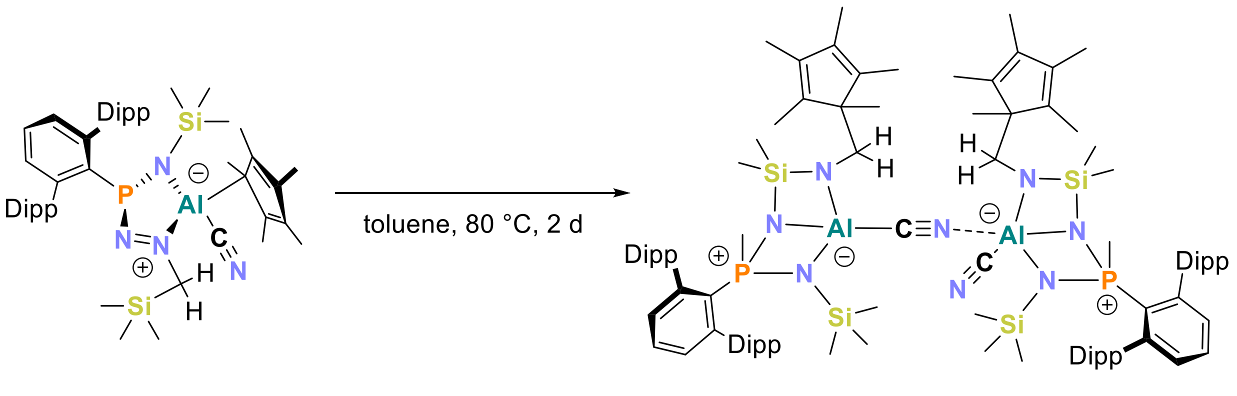

5P **6P** (29%)

^{Dipp}TerPAlCp* (40.0 mg, 67.8 *μ*mol, 1.00 eq.) was dissolved in *n*-hexane (0.5 mL) in a *J.-Young* NMR tube and a solution of (trimethylsilyl)diazomethane (0.6 M in *n*-hexane, 0.24 ml, 144 *μ*mol, 2.10 eq.) was added at ambient temperature. Upon addition, the colour of the reaction mixture instantly changed from deep purple to pale-yellow and after 2 h at −30 °C **4P** precipitated as colourless crystalline solid. The remaining supernatant was removed with a glass pipette and the solid was carefully washed with cold (–30 °C) *n*-pentane (2 × 0.1 mL). The isolated crystalline fraction was dried for 30 min at ambient temperature at 1×10^{-3} mbar. The colourless crystalline solid of **4P** was re-dissolved in toluene-d_8 (0.5 mL). For the thermal rearrangement, the pale-yellow solution of **4P** was heated at 80 °C (heating block) for 2 days and the colour of the reaction mixture changed to yellow. Full conversion of **4P** to compound **6P** was confirmed via $^{31}P\{^1H\}$ NMR spectroscopy. All volatile components were removed by evaporation at ambient temperature in a glovebox. The orange oil was re-dissolved in a mixture of *n*-hexane (0.5 mL) and HMDSO (0.05 mL). The *n*-hexane (0.5 mL) / HMDSO (0.05 mL) mixture was placed −30 °C for 72 h in a small vial and the solvent was slowly removed by evaporating until crystallization of **6P**.

Suitable crystals for single crystal X-ray diffraction were obtained from a *n*-hexane (0.5 mL) and HMDSO (0.05 mL) solution of **6P** at −30 °C for 72 h.

Note: **6P** shows partial decomposition *in vacuo* (~1×10^{-3} mbar) and poor crystallization behaviour after removal of the solvent *in vacuo* (~1×10^{-3} mbar). Only the formal HCN-addition product **6P^{H}** (*vide supra*) with protonation of a nitrogen atom and cyanide transfer to Al could be crystallised in this way.

Yield of 6P: 16.2 mg (9.9 μmol, 29%) of a colourless crystalline solid

^{1}H NMR (500.1 MHz, Tol-d$_8$, 298 K): δ = −0.09 (s, 3 H, Si(C*H*$_3$)(CH$_3$)), 0.12 (s, 9 H, NSi((C*H*$_3$)$_3$)), 0.14 (s, 3 H, Si(CH$_3$)(C*H*$_3$)), 0.75 (s, 3 H, C$_5$(C*H*$_3$)$_5$), 0.89 (d, $^{3}J_{(H-H)}$ = 6.8 Hz, 6 H, C(H)(C*H*$_3$)(CH$_3$) (group A)), 0.95 (d, $^{3}J_{(H-H)}$ = 6.6 Hz, 6 H, C(H)(C*H*$_3$)(CH$_3$) (group B)), 1.12 (d, $^{3}J_{(H-P)}$ = 13.7 Hz, 3H, P-C*H*$_3$)[xxxviii], 1.31 (d, $^{3}J_{(H-H)}$ = 6.8 Hz, 6 H, C(H)(CH$_3$)(C*H*$_3$) (group A)), 1.48 (d, $^{3}J_{(H-H)}$ = 6.6 Hz, 6 H, C(H)(CH$_3$)(C*H*$_3$) (group B)), 1.65 (s, 6 H, C$_5$(C*H*$_3$)$_5$), 1.70 (d, *J* = 0.9 Hz, 3 H, C$_5$(C*H*$_3$)$_5$), 1.83 (s, 3 H, C$_5$(C*H*$_3$)$_5$), 2.76 – 2.87 (superimposed signal, 5 H, C(*H*)(CH$_3$)(CH$_3$) and NC*H*$_2$Cp*), 3.13 (d, $^{2}J_{(H-H)}$ = 13.3 Hz, 1 H, NC*H*$_2$Cp*), 6.91 – 6.98 (m, 3H, *m*- and *p-H* (^{Dipp}Ter)), 7.09 (d, $^{3}J_{(H-H)}$ = 7.7 Hz, 1 H, *m-H* (Dipp), 7.10 (d, $^{3}J_{(H-H)}$ = 7.7 Hz, 1 H, *m-H* (Dipp), 7.25 – 7.30 (m, 2H, *m-H* (Dipp), 7.33 (t, *J* = 7.7 Hz, 2H, *p-H* (Dipp), ppm. **^{13}C{^{1}H} NMR** (125.8 MHz, Tol-d$_8$, 298 K): δ = 3.4 (d, $J_{(C-P)}$ = 3.3 Hz, NSi((CH$_3$)$_3$)), 3.5 (d, $J_{(C-P)}$ = 6.3 Hz, Si(C*H*$_3$)(CH$_3$)), 6.1 (s, Si(CH$_3$)(CH$_3$)), 10.6 (s, C$_5$(CH$_3$)$_5$), 11.31 (s, C$_5$(CH$_3$)$_5$), 11.5 (s, C$_5$(CH$_3$)$_5$), 11.6 (s, C$_5$(CH$_3$)$_5$), 20.1 (s, C$_5$(CH$_3$)$_5$), 23.6 (s, C(H)(CH$_3$)(*C*H$_3$) (group A)), 23.9 (s, C(H)(CH$_3$)(*C*H$_3$) (group B)), 25.4 (d, $^{1}J_{(C-P)}$ = 60.8 Hz), 25.8 (s, C(H)(CH$_3$)(CH$_3$) (group A)), 26.1 (s, C(H)(CH$_3$)(CH$_3$) (group B)), 31.2 (s, *C*(H)(CH$_3$)(CH$_3$) (group B)), 31.3 (s, *C*(H)(CH$_3$)(CH$_3$) (group A)), 48.9 (s, NCH$_2$Cp*), 59.3 (s, *C*$_5$(CH$_3$)$_5$), 123.4 (s, *m*-CH (Dipp)), 124.8 (s, *m*-CH (Dipp)), 129.0 (s, *m*-CH (^{Dipp}Ter))[xxxix], 129.9 (s, *p*-CH (Dipp), 132.8 (s, *p*-CH (Dipp), 133.6 (s, *C*), 133.7 (s, *C*$_5$(CH$_3$)$_5$), 134.6 (s, CH), 134.8 (s, *C*$_5$(CH$_3$)$_5$), 139.6 (s, *C*$_5$(CH$_3$)$_5$), 140.3 (s, *i-C* (^{Dipp}Ter)), 140.7 (s, C$_5$(CH$_3$)$_5$), 144.6 (s, *i-C* (Dipp)), 145.8 (s, *o-C* (Dipp)), 146.8 (s, *o-C* (Dipp)) ppm. **^{29}Si{^{1}H} NMR** (99.4 MHz, Tol-d$_8$, 298 K): δ = −3.04 (s, N*Si*((CH$_3$)$_3$)), −3.22 (s, *Si*(CH$_3$)(CH$_3$)) ppm.

[xxxviii] Appears as a singlet resonance in the ^{1}H{^{31}P} NMR spectrum.
[xxxix] Superimposed with solvent signal, assigned with ^{1}H/^{13}C HSQC spectrum.

$^{31}P\{^1H\}$ NMR (202.5 MHz, Tol-d$_8$, 298 K): δ = 38.95 (s) ppm. **IR** (ATR, 128 scans, cm^{-1})[xl]: $\tilde{\nu}$ = 3059 (vw), 2960 (w), 2924 (s), 2864 (vw), 2151 (vw, CN group), 2089 (vw, CN group), 1659 (vw), 1566 (vw), 1460 (w), 1443 (vw), 1426 (vw), 1383 (vw), 1362 (vw), 1324 (vw), 1248 (s), 1183 (vw), 1137 (w), 1125 (m), 1074 (m), 1023 (s), 932 (vw), 899 (vw), 837 (vs), 817 (vs), 795 (s), 753 (vs), 720 (w), 682 (w), 626 (w), 602 (w), 575 (w), 533 (w), 514 (m), 457 (w). **LIFDI-MS** (m/z) calc: 1637.9724 (100%) [$C_{96}H_{144}Al_2N_8P_2Si_4$]; found: 819.4921, 820.4955, 821.4988, 822.4923 [$C_{48}H_{72}AlN_4PSi_2$+(H^+)].

[xl] For this IR spectrum, 128 scans were used to achieve a better signal-to-noise ratio for a better resolution of the cyanide region.

Figure S109: ^{1}H NMR spectrum of **6P** (500.1 MHz, Tol-d$_8$, 298 K).

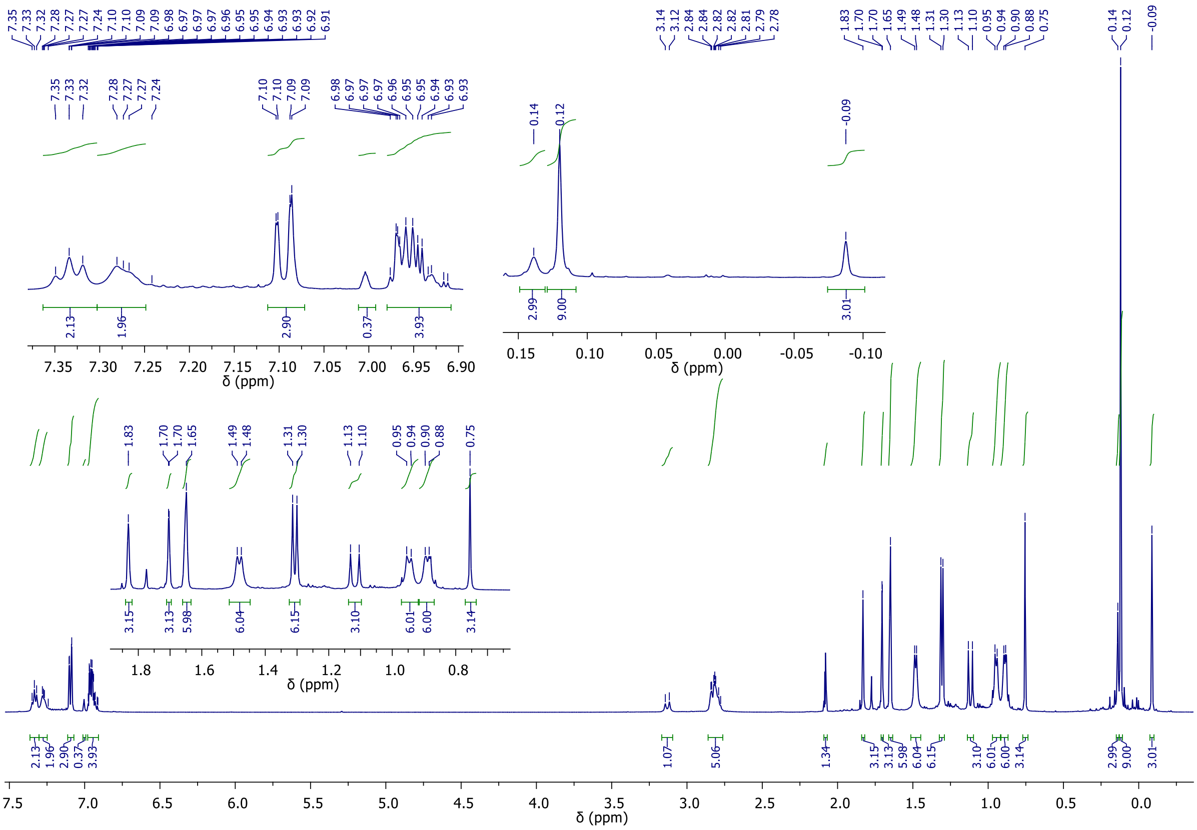

Figure S110: ^{13}C{^{1}H} NMR spectrum of **6P** (125.8 MHz, Tol-d$_8$, 298 K).

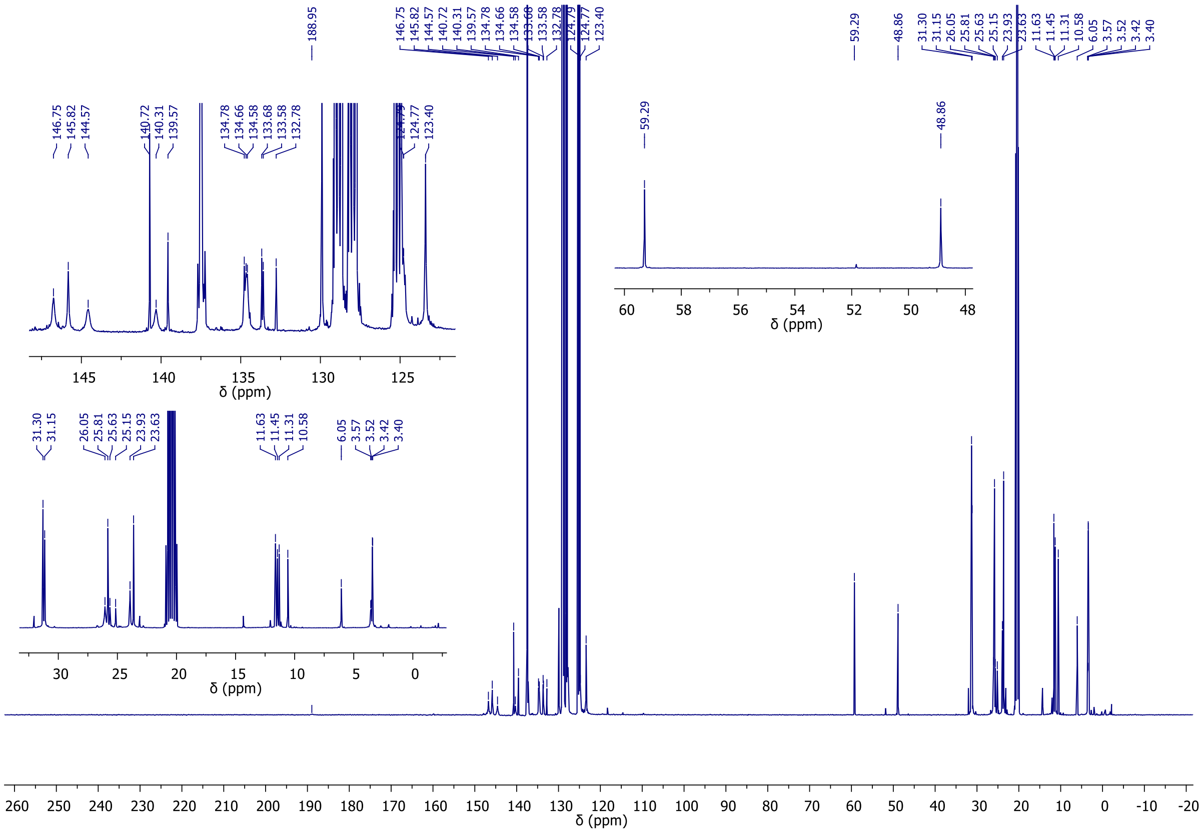

Figure S111: $^{29}Si\{^{1}H\}$ NMR spectrum of **6P** (99.4 MHz, Tol-d_8, 298 K).

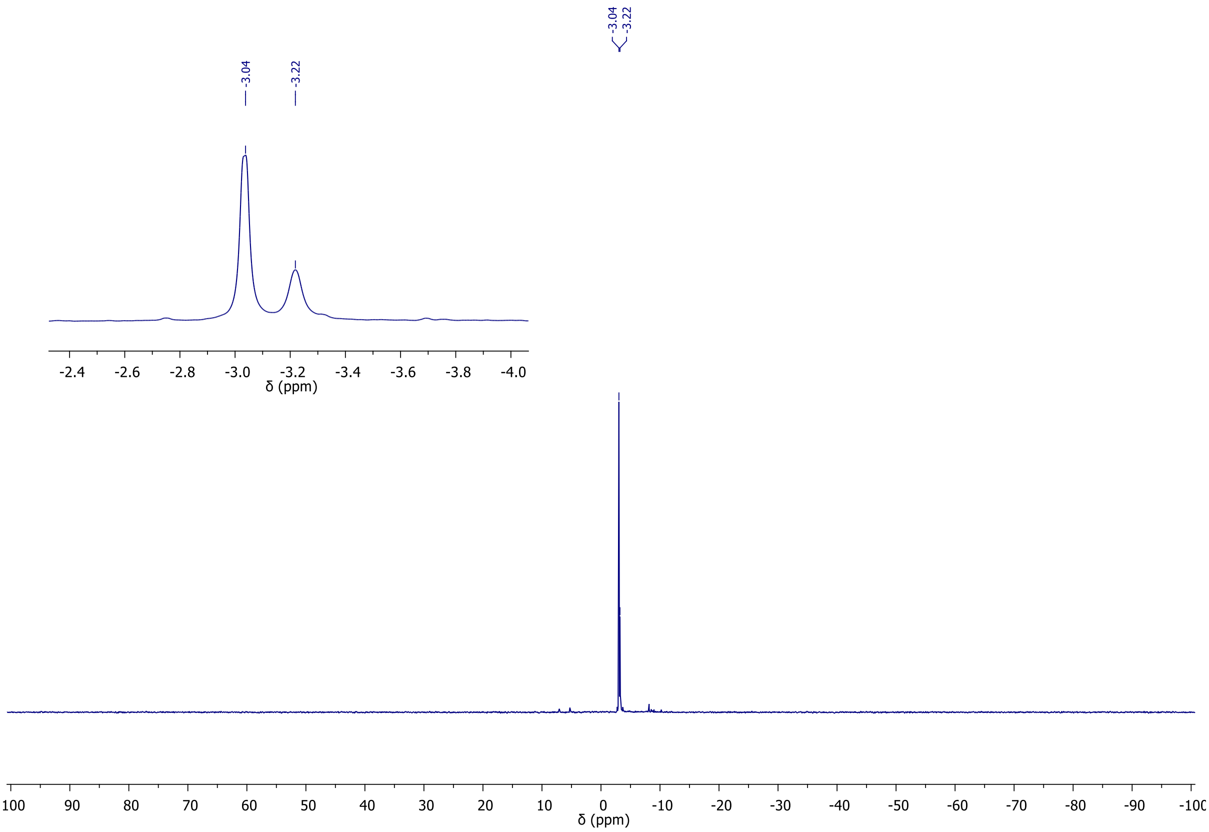

Figure S112: $^{31}P\{^{1}H\}$ NMR spectrum of **6P** (202.5 MHz, Tol-d_8, 298 K).

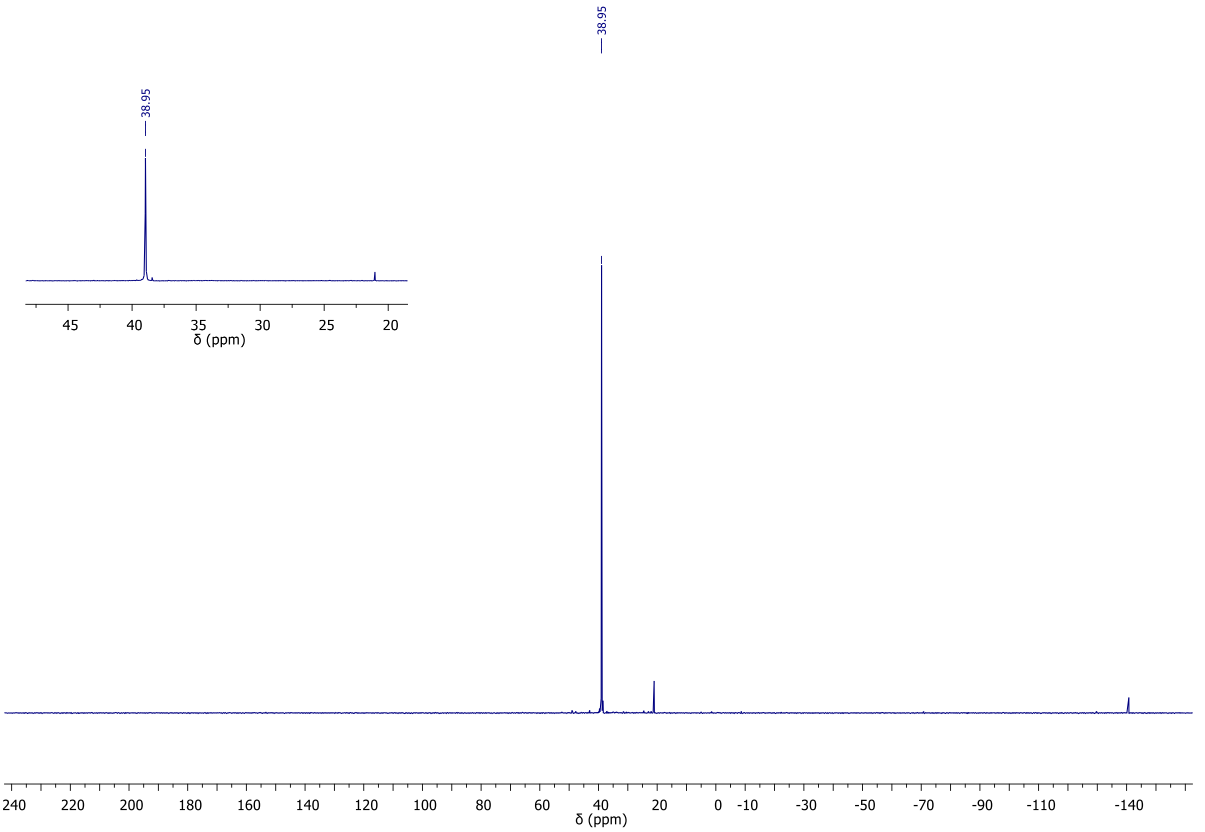

Figure S113: IR spectrum of **6P** (ATR, 128 scans, cm^{-1}, powder).[xli]

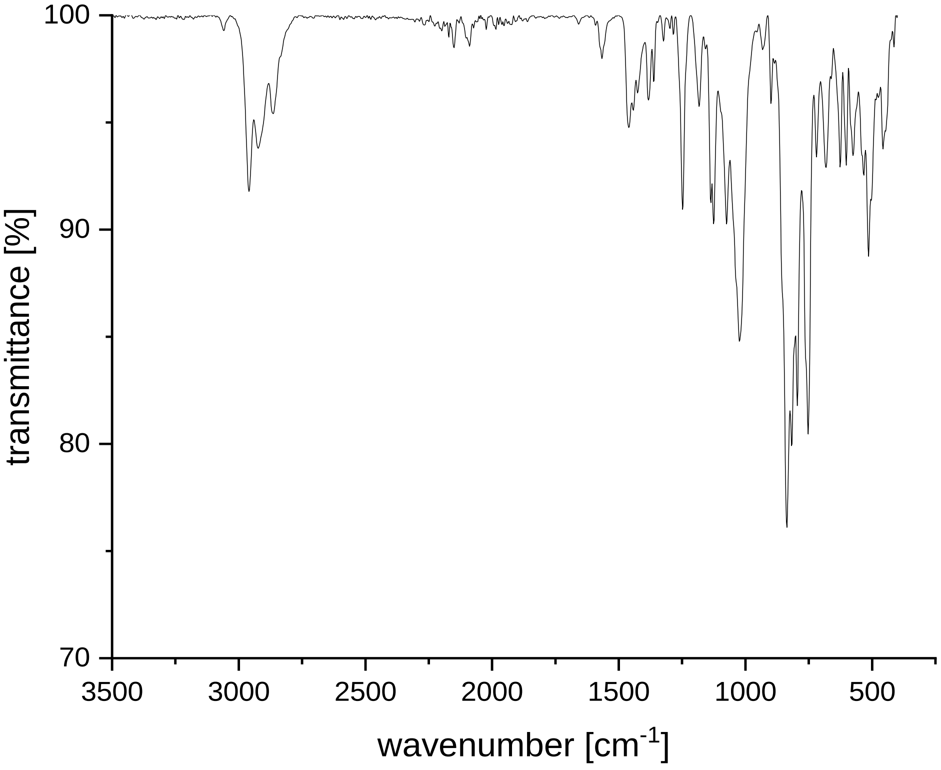

[xli] For this IR spectrum, 128 scans were used to achieve a better signal-to-noise ratio and for a better resolution of the cyanide region.

Figure S114: LIFDI mass spectrum of **6P** (toluene).

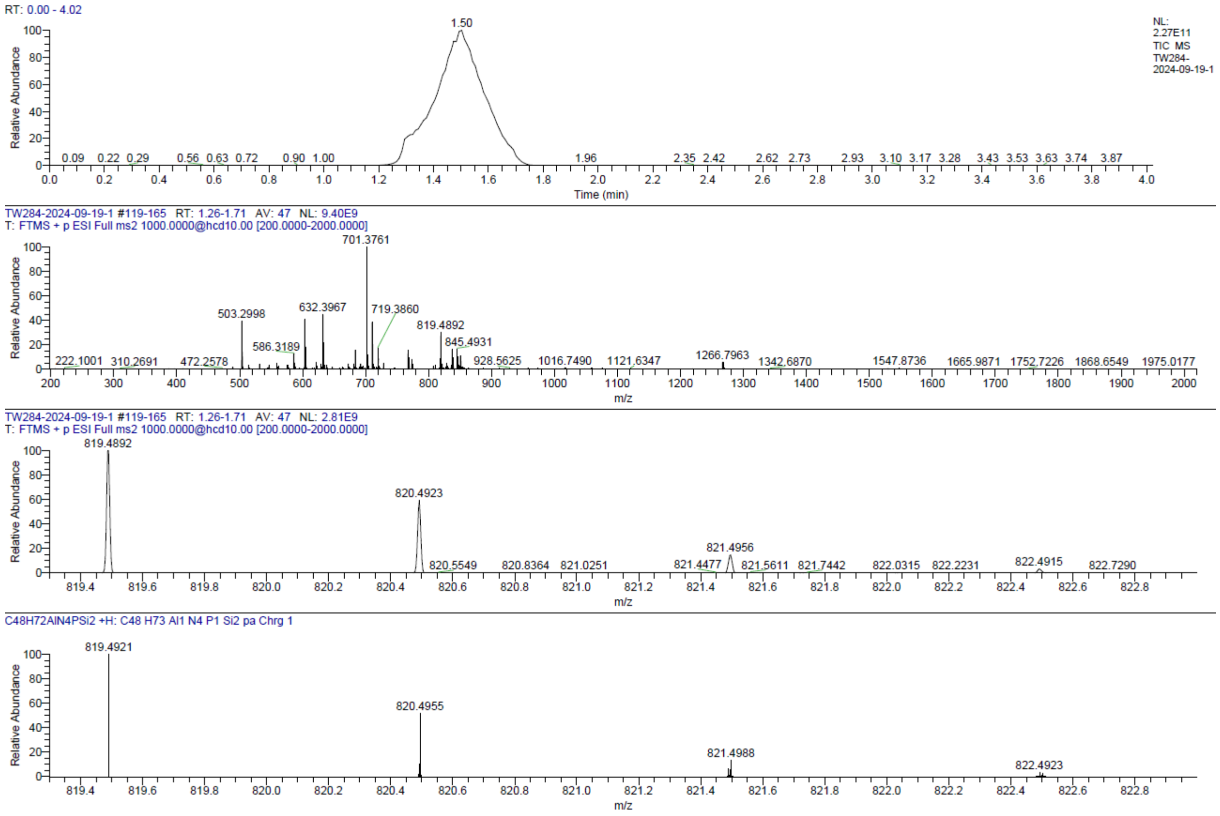

4.16 Substrate scope of additionally tested azides

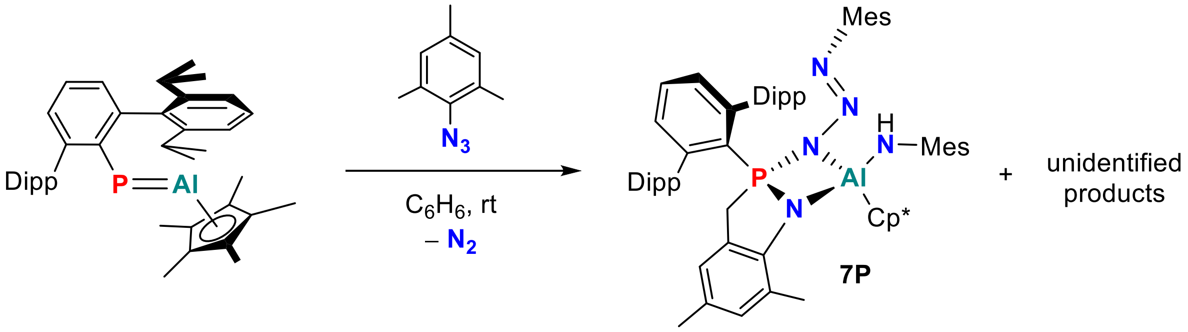

A variety of other azides were tested in the reaction with **DippTerPAlCp***. Only in the case of $MesN_3$ X-Ray quality crystals were obtained. Similar to **2P**, simultaneous MesN and $MesN_3$ insertion is observed, with an additional MesNH-unit on the Al atom, along with an η^1-Cp* group. The NH group arises from intramolecular C-H activation of one *o*-Me groups of the μ-NMes unit. This resulted in P–C bond formation and a tetra-coordinate P atom. DFT calculations using the GIAO-methid on the PBE0-D3/def2TZVP//PBE0-D3/def2SVP level of theory, revealed a gas phase ^{31}P NMR shift of 93.4 ppm and thus it can be concluded that two of the resonances in the $^{31}P\{^1H\}$ NMR spectrum at 78.0 and 73.3 ppm correspond to different isomers of **7P**.

Table S7 shows the substrate scope of additionally tested azides and diazomethane derivatives for the reaction with **DippTerPAlCp***, including reaction conditions, $^{31}P\{^1H\}$ NMR data and our suggestion of a likely reaction product. Overall, purification and crystallization issues due to the compound’s excellent solubility in all available solvents (benzene, *o*-DFB, *n*-pentane, *n*-hexane, HMDSO, THF, Et_2O, DME) limits the experimental data to $^{31}P\{^1H\}$ NMR data.

Table S7. Substrate scope of tested azides, reaction conditions, $^{31}P\{H\}$ NMR data and suggested reaction products.

**Organoazide**	**Reaction conditions**	**Observed via $^{31}P\{^1H\}$ NMR spectroscopy**	**Suggested product**
2′-Azido-2,2′′,4,4′′,6,6′′-hexamethyl-1,1′:3′,1′′-terphenyl ($^{Mes}TerN_3$)			
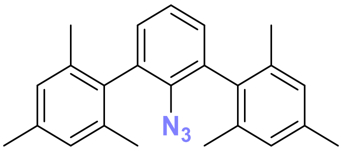	1.00 eq., C_6D_6,<br>ambient temp.	−1.1 ppm<br>Phosphafluorene<br>(6-(2,6-di*iso*propylphenyl)-<br>1,5-di*iso*propyl-5*H*-<br>benzo[b]phosphindole)	^{Mes}TerN=AlCp*
2-Azido-1,3,5-trimethylbenzene ($MesN_3$)			
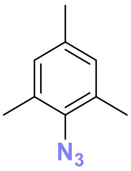	exc., C_6D_6,<br>ambient temp. /<br>3.00 eq., C_6D_6,<br>ambient temp. /<br>3.00 eq., C_6D_6,<br>60 °C to 80 °C /<br>1.00 eq., C_6D_6, h*ν*,<br>ambient temp., /<br>1.90 eq., C_6D_6,<br>ambient temp. /<br>1.90 eq., o-DFB,<br>−30 °C	unselective,<br>major resonances:<br>78.0 ppm, 73.3 ppm,<br>12.8 ppm, 11.6 ppm,<br>118.1 ppm	few single<br>crystals of **7P**<br>obtained
4-Azido-*N*,*N*-dimethylaniline ($^{NMe2}PhN_3$)			
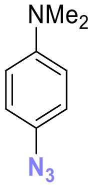	1.00 eq., C_6D_6,<br>ambient temp. /<br>1.00 eq., dilution,<br>C_6D_6, ambient<br>temp. /<br>2.00 eq., C_6D_6,<br>ambient temp. /<br>2.00 eq., *o*-DFB<br>−30 °C	unselective,<br>major resonances:<br>87.7 ppm<br>(Diazaphosphaalumetidine),<br>9.0 ppm, 63.2 ppm,<br>101.5 ppm, 130.7 ppm	-
1-Azido-4-methoxybenzene ($^{OMe}PhN_3$)			
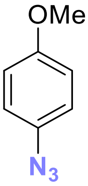	1.00 eq., C_6D_6,<br>ambient temp. /<br>2.00 eq., C_6D_6,<br>ambient temp.	unselective<br>major resonances:<br>87.6 ppm<br>(Diazaphosphaalumetidine)<br>63.2 ppm, 7.7 ppm	-
Trimethylsilyl azide ($TMSN_3$)			
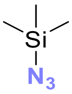	exc., C_6D_6,<br>ambient temp.	selective<br>37.9 ppm<br>(unidentified product),<br>−140.5 ppm ($^{Dipp}TerPH_2$),	-

Organoazide	Reaction conditions	Observed via $^{31}P\{^1H\}$ NMR spectroscopy	Suggested product
Sodium azide (NaN_3)			
NaN_3	exc., C_6D_6, ambient temp. / exc., C_6D_6, sonification / exc., C_6D_6, 60 °C to 80 °C	no reaction	-
2-Azido-1,3,2-benzodioxaborole (CatB-N_3)			
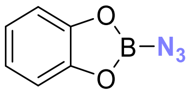	1.00 eq., C_6D_6, ambient temp. / 1.00 eq., C_6D_6, hν, ambient temp.	unselective / decomposition, major resonances: −70.4 ppm, −34.1 ppm, −50 ppm −1.1 ppm (Phosphafluorene)	-
1-Azidooctane (*n*OcN_3)			
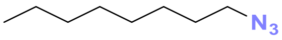	1.00 eq., C_6D_6, ambient temp. /	selective 1.5 ppm (unidentified product)	-
Adamantyl azide (AdN_3)			
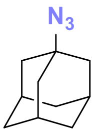	1.00 eq., C_6D_6, ambient temp. / 1.00 eq., C_6D_6, 60 °C to 105 °C / 1.00 eq., C_6D_6, hν, ambient temp.	no reaction, decomposition −140.5 ppm ($^{Dipp}TerPH_2$)	-
4,4'-(Diazomethylene)bis(methylbenzene)			
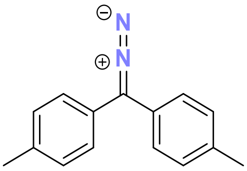	1.00 eq., C_6D_6, ambient temp. / 2.00 eq., C_6D_6, ambient temp. /	unselective major resonances: 133.7 ppm, 22.6 ppm, 22.1 ppm	-
1,5-Diazidonaphthalene (Naph$(N_3)_2$)			
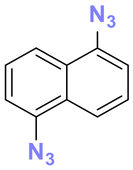	2.00 eq., C_6D_6, ambient temp.	precipitation of an amorphous insoluble solid after 30 min	-

Organoazide	Reaction conditions	Observed via ${}^{31}P\{{}^{1}H\}$ NMR spectroscopy	Suggested product
1-Azido-2,3,4,5,6-pentafluorobenzene (${}^{F}PhN_3$)			
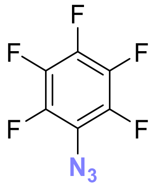	2.00 eq., C_6D_6,<br>ambient temp.	−1.1 ppm<br>(Phosphafluorene)<br>114.5 ppm	-
1-Azidotrimethylstannane<br>(Me_3SnN_3)			
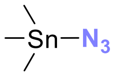	1.00 eq., ambient temp., C_6D_6,<br>1.00 eq., 60 to 80 °C, C_6D_6<br>1.00 eq., UV, ambient temp., C_6D_6	unselective<br>major resonances:<br>−1.1 ppm<br>(Phosphafluorene)<br>−163.1 ppm<br>minor resonances:<br>−124.0 ppm, −152.7 ppm, 79.7 ppm, 78.0 ppm, 73.3 ppm, 55.0 ppm, 50.2 ppm.	P-Sn species

5. Computational details

General remarks

Computations were carried out using Gaussian16,[95] ORCA 4.2.1[96] and the standalone version of NBO 6.0[73-77] in conjunction with Gaussian09.[97] ELF and AIM calculations were performed with Multiwfn 3.8(dev).[77]

Structure optimizations employed the hybrid DFT functional PBE0[61-63] in conjunction with Grimme's dispersion correction D3(BJ)[64,65] and the def2-SVP basis set[66](notation e.g. PBE0-D3/def2-SVP). All structures were fully optimized and confirmed as minima by frequency analyses.

Chemical shifts were derived by the GIAO method.[67-71] The calculated absolute shifts ($\sigma_{calc,X}$) were referenced to the experimental absolute shift of 85 % H_3PO_4 in the gas phase ($\sigma_{ref,1}$ = 328.35 ppm),[72] using PH_3 ($\sigma_{ref,2}$ = 594.45 ppm) as a secondary standard:

$$\begin{aligned} \delta_{calc,X} &= (\sigma_{ref,1} - \sigma_{ref,2}) - (\sigma_{calc,X} - \sigma_{calc,PH_3}) \\ &= \sigma_{calc,PH_3} - \sigma_{calc,X} - 266.1 \text{ ppm} \end{aligned}$$

At the PBE0-D3/def2-TZVP level of theory, σ_{calc,PH_3} amounts to +567.77 ppm.

The rearrangement of **4P** towards **5P_CN** and onwards to **6P** was investigated with respect to the thermodynamics of the process. More accurate estimates of the electronic energy were obtained by single-point DLPNO-CCSD(T)/def2-TZVP[98,99,100] computations (notation DLPNO-CCSD(T)/def2-TZVP//PBE-D3/def2-SVP). The T_1 diagnostic was evaluated in each case to ensure reliable results. (Empirically, CCSD(T) results with T_1 values smaller than 0.02 are considered reliable.)[101] Solvation energies were added as the difference between E_{tot} and $E_{tot,solv}$ obtained through single point calculations employing Truhlar's continuum solvation model SMD for benzene (scrf=smd,benzene(or toluene)).[102]

Optimized Structures. In addition to the electronic supporting information we provide a multi-structure xyz-file including all calculated molecules. For a better understanding and a more intuitive view of the calculated 3D structures, we strongly recommend using this file e.g. with the free program MERCURY.

Please note that most of the computations were carried out for single, isolated molecules in the gas phase (ideal gas approximation). There may well be significant differences between gas phase and condensed phase.

5.1.1 Summary of calculated data

Table S8. Summary of calculated data.

Compd.	PG	N_{imag}	E_{tot}[a]	U_0[a]	U_{298}[a]	H_{298}[a]	G_{298}[a]
**1P**	C_1	0	−2708.0263	−2707.0129	−2706.9555	−2706.9546	−2707.1021
**1As**	C_1	0	−4602.0255	−4601.0163	−4600.9574	−4600.9565	−4601.1104
**1P·PMe$_3$**	C_1	0	−3168.6969	−3167.5695	−3167.5041	−3167.5031	−3167.6661
**1As·PMe$_3$**	C_1	0	−5062.8981	−5061.7728	−5061.7066	−5061.7056	−5061.8723
**2P**	C_1	0	−13187.5783	−13186.5411	−13186.4723	−13186.4714	−13186.6472
**2As**	C_1	0	−15081.7846	−15080.7492	−15080.6796	−15080.6787	−15080.8569
**3P**	C_1	0	−3397.4494	−3396.0766	−3395.9991	−3395.9981	−3396.1854
**3As**	C_1	0	−5291.6587	−5290.28786	−5290.20955	−5290.2086	−5290.3990
**4P**	C_1	0	−3249.7465	−3248.65167	−3248.5835	−3248.5826	−3248.7528
**4As**	PG	0	−5143.9528	−5142.85979	−5142.7910	−5142.7900	−5142.9625
**5P_CN**	C_1	0	−3249.7990	−3248.7040	−3248.6358	−3248.6349	−3248.8027
**5P_NC**	C_1	0	−3249.7968	−3248.7023	−3248.6339	−3248.6330	−3248.8014
**6P**	C_1	0	−3249.8868	−3248.7871	−3248.7206	−3248.7197	−3248.8860
**1P_Dipp**	C_1	0	−3178.8719	−3177.51627	−3177.4442	−3177.4433	−3177.6133
**1P_Br$_2$Tol**	C_1	0	−13078.3067	−13077.2798	−13077.2130	−13077.2121	−13077.3806

[a] energy in a.u.

Table S9: Comparison of calculated vs. experimentally determined Pn−N and N−Al distances. Calculated chemical shifts ($\delta_{calc,X}$) as obtained from a GIAO calculation at the PBE0-D3/def2TZVP level of theory in the gas phase using 85% H_3PO_4 and PH_3 as standards.

	$d_{exp.}$(Pn−N)	$d_{calc.}$(Pn−N)	$d_{exp.}$(Al−N)	$d_{calc.}$(Al−N)	$\delta_{exp.}$(^{31}P)	$\delta_{calc.}$(^{31}P)
**1P**	1.717(1)<br>1.7218(12)	1.741<br>1.738	1.839(1)<br>1.851(1)	1.850<br>1.858	84.2	80.0
**1P·PMe$_3$**	1.727(1)<br>1.737(1)	1.746<br>1.740	1.856(1)<br>1.873(1)	1.878<br>1.884	108.5<br>−47.6	108.9<br>−43.6
**2P**	1.754(2)<br>1.760(2)	1.777<br>1.767	1.848(2)<br>1.853(2)	1.857<br>1.868	77.9	70.6
**3P**	1.769(2)<br>1.776(2)	1.794<br>1.787	1.847(2)<br>1.855(2)	1.856<br>1.865	57.1	58.3
**4P**	1.753(1)<br>1.754(1)	1.776<br>1.771	1.849(1)<br>1.833(1)	1.856<br>1.848	46.8	45.3
**5P_CN**	1.673(1)<br>1.678(1)	1.686<br>1.699	1.896(1)<br>1.981(1)	1.905<br>2.025	137.5	147.8
**5P_NC**	-	1.686<br>1.670	-	1.904<br>2.020	136.8	148.3
**6P**	-	-	-	-	39.0	43.6
**7P**	-	-	-	-		93.6
**1P_Dipp**					102.1	128.0
**1P_Br$_2$Tol**					101.0	96.6

Table S10: Calculated absolute shifts ($\sigma_{calc,X}$) as obtained from a GIAO calculation at the PBE0-D3/def2TZVP/SCRF=(solvent=benzene) level of theory in the gas phase and chemical shift using 85% H_3PO_4 and PH_3 as standards.

Compound	$\sigma_{calc,X}$	$\delta_{calc,X}$ [ppm]
**1P**	221.6	80.0
**1P_F**	220.3	81.4
**1P•PMe$_3$**	192.8<br>345.3	108.9<br>−43.6
**2P**	231.1	70.6
**3P**	243.4	58.3
**4P**	256.3	45.3
**5P_CN**	153.9	147.8
**5P_NC**	153.3	148.3
**6P**	258.0	43.6
**7P**	208.2	93.4
**^{Dipp}TerP(μ-NDipp)$_2$AlCp***	173.7	128.0
**^{Dipp}TerP(μ-NBrTol)$_2$AlCp***	205.0	96.6

5.1.2 Steric demand of different azides

The steric accessibility of the N_α and N_γ atoms in Ph-, Br_2Tol- and Dipp-N_3 was investigated using the ray-tracing tool AtomAccess.[103] This clearly showed, that the N_α atom of Ph-N_3 is most accessible (16.9 %), while N_α in Dipp-N_3 is more shielded, with only 12.7% of the space available. No difference can be seen in the access to N_γ, which amounts to 50% in all cases. The results are summarized below. The steric accessibility of the N_α atom is expected to greatly influence the tendency to eliminate N_2 through a four-membered transition state (cf. depiction of KS-orbitals).

	Ph-N_3	Br_2Tol-N_3	Dipp-N_3
N_α	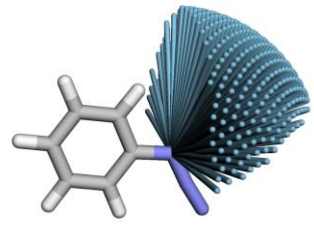 16.9 %	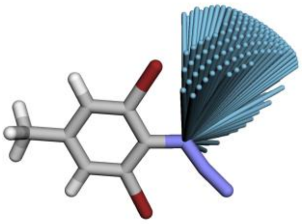 13.4 %	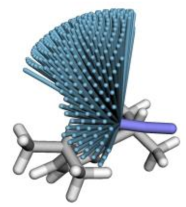 12.6 %
N_γ	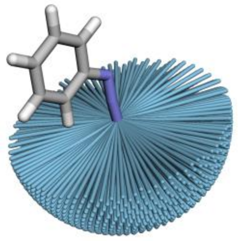 50.1 %	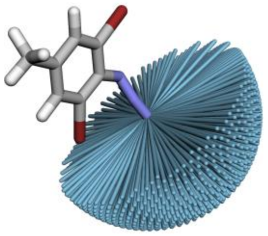 50.0 %	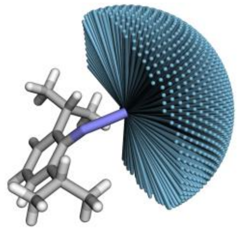 48.4 %

5.1.3 Thermodynamic considerations

Calculations at the DLPNO-CCSD(T)/def2-TZVP//PBE-D3/def2-SVP(smd=solvent) were carried out to determine whether the rearrangement of **4P** to **5P** and eventually to **6P** (when **5P** is heated to 80 °C) is thermodynamically feasible. This revealed that the formation of **5P_CN** is strongly exergonic ($\Delta_R G°_{298K,C6H6}$ = −211.8 kJ·mol^{-1}, $\Delta_R G°_{298K,Toluene}$ = −212.2 kJ·mol^{-1}). The P−CN derivative **5P_CN** is thermodynamically less stable than **5P_NC**, albeit a miniscule difference between both isomers of ca. 1.3 kJ·mol^{-1} is calculated. Considering the size of the molecule this energy difference should is in the range of conformational changes in the molecular periphery and should therefore not be over-interpreted. Consequently, a differentiation between **5P_CN** and **5P_NC** is difficult based on these calculations. It is however clear that **5P_CN** is prone to further evolve in solution, as the rearrangement to **6P**, with a terminal Al−CN unit is strongly exergonic ($\Delta_R G°_{298K,Toluene}$ = −295.2 kJ·mol^{-1}).

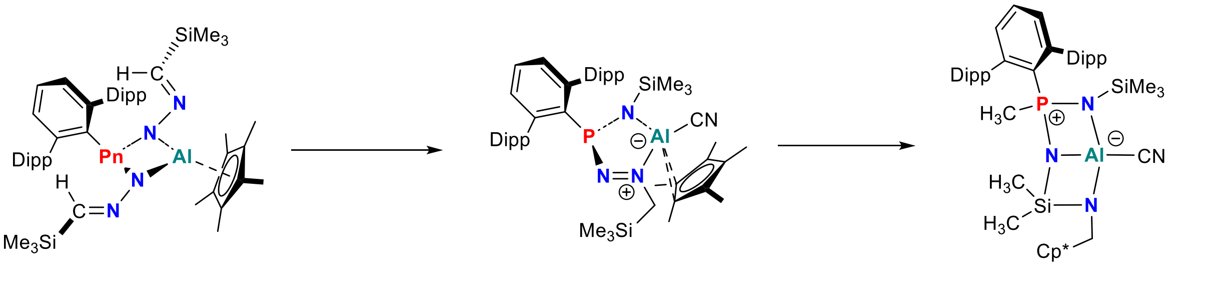

4P **5P** **6P**

$\Delta_R G°_{298K,C6H6}$ = −211.8 kJ·mol^{-1}
$\Delta_R G°_{298K,Toluene}$ = −212.2 kJ·mol^{-1}

$\Delta_R G°_{298K,Toluene}$ = −295.2 kJ·mol^{-1}

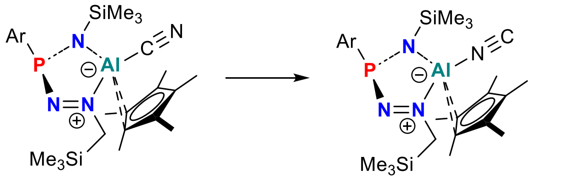

5P_CN **5P_NC**

$\Delta_R G°_{298K,C6H6}$ = −1.31 kJ·mol^{-1}
$\Delta_R G°_{298K,Toluene}$ = −1.32 kJ·mol^{-1}

5.1.4 NBO Analyses

NBO analyses were carried out using the structures optimized at the PBE0-D3/def2-SVP level of theory. The densities were obtained at the PBE0-D3/def2-TZVP level of theory.

First then natural charges were evaluated, these were derived from a Natural Population Analysis (NPA). This clearly showed accumulation of positive charge on the aluminium centers (ca. +2 e^-), while negative charge is accumulated on the N atoms (Table S11). It needs to be noted that the negative charge on the N atoms in derivatives **2Pn**, **3Pn** and **4Pn** is delocalized into the respective π-systems of the intact N_3R or N_2CR_2 units. NBO analyses revealed two lone pairs of electrons on each of the N atoms. One of the LPs has nearly ideal sp^2 character, while the second LP has mainly pure p-character. The sp^2-type LPs are directed towards the Al atom and can be interpreted as the highly polarized Al−N bonds, which have a high ionic character. The Pn−N bonds are likewise strongly polarized towards the N atoms, with an additional LP on the Pn atom, which has a high s-character and interacts weakly with a lone valence on aluminium according to second order perturbation analysis. Consequently, all four-membered species are best described by three major resonance forms (**A**, **B**, **B'**) and a non-bonding form in **C**.

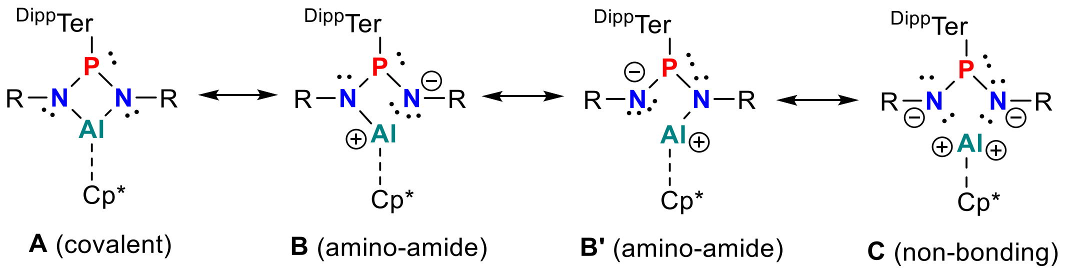

Table S11: Summary of natural charges (in e^-) and Wiberg Bond Indexes for compounds **1Pn-4Pn**.

	q(Pn)	*q(Al)*	*q(N1)*	*q(N2)*	**$WBI_{avg.}$ (P–N)**	**$WBI_{avg.}$ (Al–N)**
**1P**	1.22	2.02	−1.21	−1.21	0.80	0.37
**1P·PMe$_3$**	1.21	1.88	−1.22	−1.21	0.81	0.26
**1As**	1.31	2.01	−1.23	−1.23	0.74	0.39
**1As·PMe$_3$**	1.29	1.89	−1.23	−1.23	0.75	0.27
**2P**	1.19	2.01	−1.24	−0.92	0.74	0.34
**2As**	1.26	2.01	−1.27	−0.92	0.67	0.34
**3P**	1.13	2.01	−0.95	−0.96	0.73	0.35
**3As**	1.19	2.01	−0.96	−0.96	0.66	0.37
**4P**	1.07	2.03	−0.97	−0.97	0.78	0.35
**4As**	1.12	2.03	−0.97	−0.97	0.73	0.37

Figure S115: Selected NLMOs of **1P** at the PBE0-D3/def2-TZVP//PBE0-D3/def2-SVP level of theory.

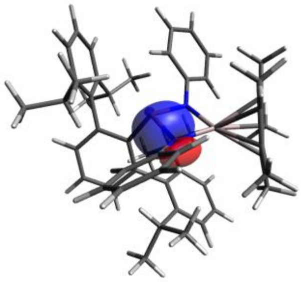

σ(P68-N69)
−18.1 eV
P: 25.7%
N: 71.7%

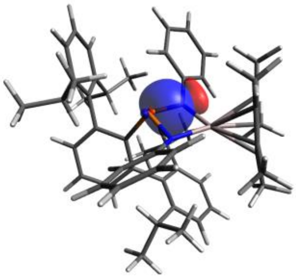

σ(P68-N71)
−18.1 eV
P: 26.4%
Ga: 71.5%

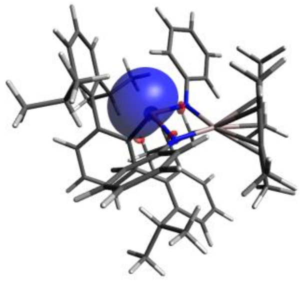

LP(P68)
−11.5 eV
s: 55.5 %
p: 44.5 %
Al: 2.1 %

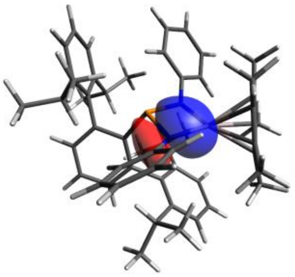

LP1(N69)
−12.0 eV
s: 13.9 %
p: 86.1 %
Al: 5.7 %
P: 1.4 %

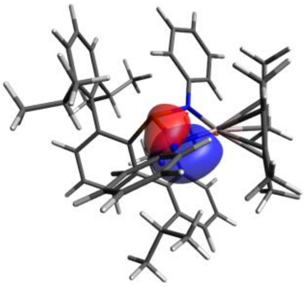

LP2(N69)
−11.5 eV
s: 18.4 %
p: 81.5 %
Al: 5.3 %

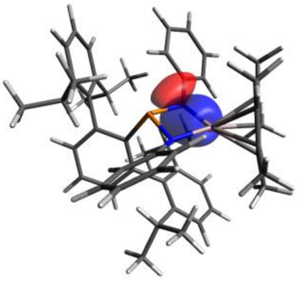

LP1(71)
−12.0 eV
s: 24.1 %
p: 75.8 %
Al: 6.6 %

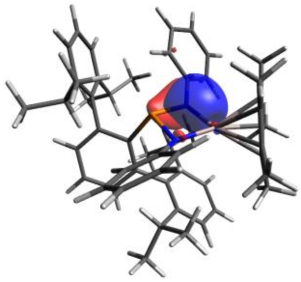

LP2(N71)
−11.0 eV
s: 9.0 %
p: 91.0 %
Al: 4.1 %

Figure S116: Selected NLMOs of **2P** at the PBE0-D3/def2-TZVP//PBE0-D3/def2-SVP level of theory.

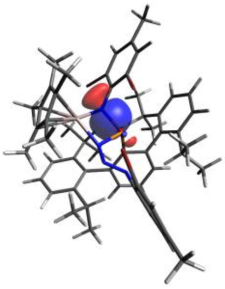

σ(P1-N3)
−17.8 eV
P: 24.8%
N: 72.2%

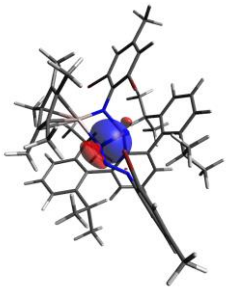

σ(P68-N71)
−18.0 eV
P: 25.5%
N: 72.2%

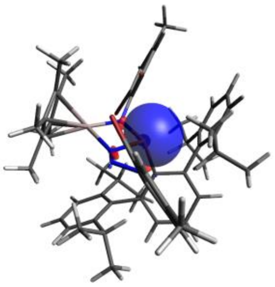

LP(P1)
−11.7 eV
s: 58.4 %
p: 41.6 %
Al: 1.7 %

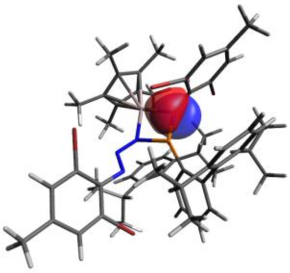

LP1(N3)
−9.8 eV
s: 13.9 %
p: 85.9 %
Al: 2.8 %
P: 1.2 %

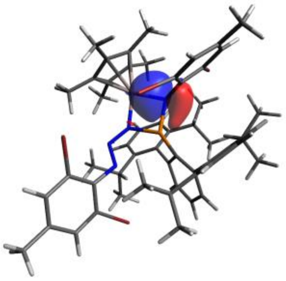

LP2(N3)
−14.2 eV
s: 22.2 %
p: 77.6 %
Al: 6.7 %

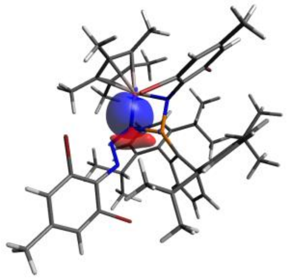

LP1(N4)
−16.0 eV
s: 36.7 %
p: 63.2 %
Al: 8.9 %

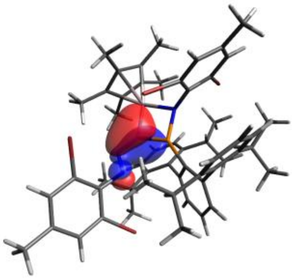

LP2(N4)
−11.0 eV
s: 1.1 %
p: 87.9 %
Al: 1.3 %
N5: 9.8 %
N6: 5.8 %

Figure S117: Selected NLMOs of **3P** at the PBE0-D3/def2-TZVP//PBE0-D3/def2-SVP level of theory.

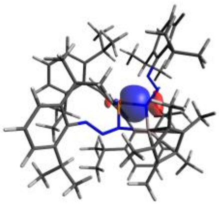

σ(P1-N3)
−17.9 eV
P: 25.7%
N: 72.0%

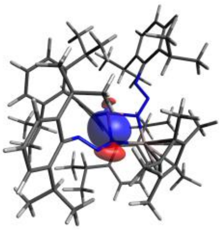

σ(P1-N6)
−17.7 eV
P: 26.3%
N: 71.2%

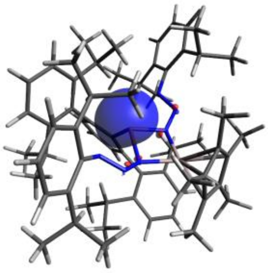

LP(P1)
−12.0 eV
s: 58.6 %
p: 41.3 %
P: 94.3 %
Al: 1.6 %

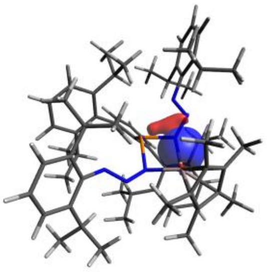

LP1(N3)
−9.8 eV
s: 36.6 %
p: 63.2 %
Al: 9.3 %
N3: 89.0 %

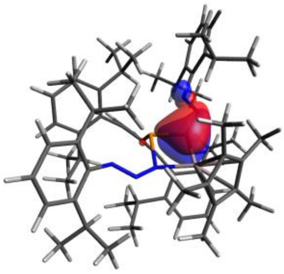

LP2(N3)
−14.2 eV
s: 1.0 %
p: 99.0 %
Al: 1.5 %
N4: 8.2
N3: 81.5 %

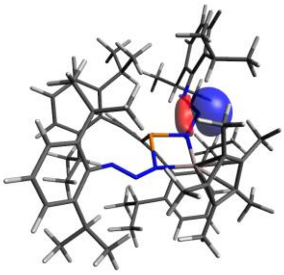

LP1(N4)
−12.5 eV
s: 39.1 %
p: 60.7 %
N4: 95.5 %

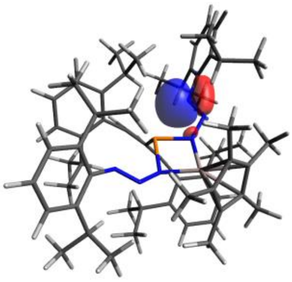

LP1(N5)
−11.8 eV
s: 37.4 %
p: 62.4 %
N5: 93.2 %

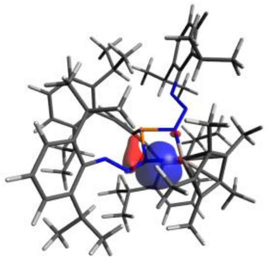

LP1(N6)
−16.0 eV
s: 39.5 %
p: 60.3 %
Al: 8.5 %
N6: 89.4 %

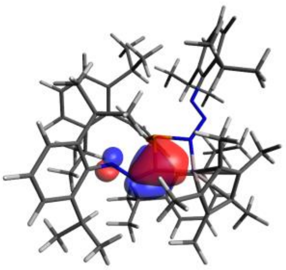

LP2(N6)
−9.3 eV
s: 1.2 %
p: 98.7 %
Al: 1.3 %
N7: 7.5
N3: 82.7 %

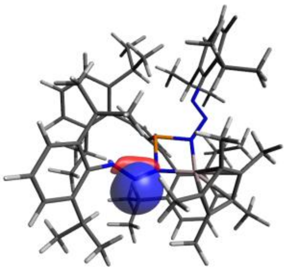

LP1(N7)
−12.5 eV
s: 39.1 %
p: 60.8 %
N4: 95.4 %

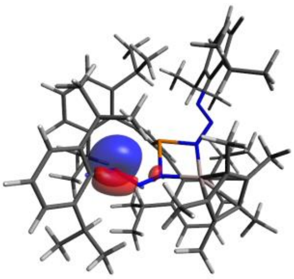

LP1(N8)
−11.9 eV
s: 38.2 %
p: 61.7 %
N8: 93.7 %

Figure S118: Selected NLMOs of **4P** at the PBE0-D3/def2-TZVP//PBE0-D3/def2-SVP level of theory.

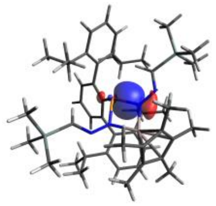

σ(P2-N3)
−17.5 eV
P: 28.3 %
N: 69.4 %

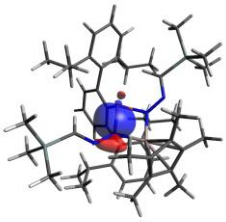

σ(P2-N4)
−17.7 eV
P: 28.0 %
N: 70.0 %

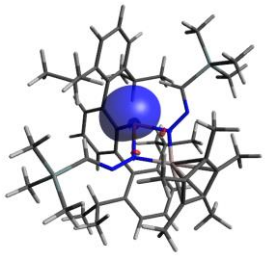

LP(P1)
−11.8 eV
s: 58.6 %
p: 41.3 %
P: 94.3 %
Al: 1.6 %

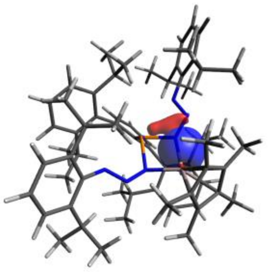

LP1(N3)
−16.0 eV
s: 38.3 %
p: 61.6 %
Al: 9.4 %
N3: 88.7 %

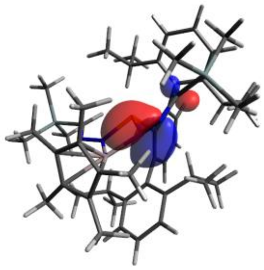

LP2(N3)
−8.7 eV
s: 0.9 %
p: 99.0 %
Al: 1.4 %
N21: 5.0
N3: 85.3 %

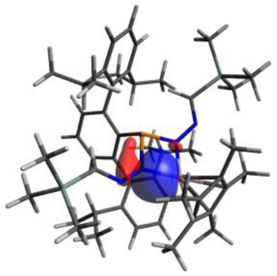

LP1(N4)
−15.6 eV
s: 36.5 %
p: 63.4 %
N4: 89.2 %
Al: 8.4

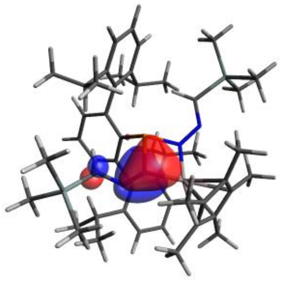

LP2(N4)
−8.5 eV
s: 37.4 %
p: 62.4 %
Al: 1.7 %
N5: 5.3 %
N4: 84.5 %

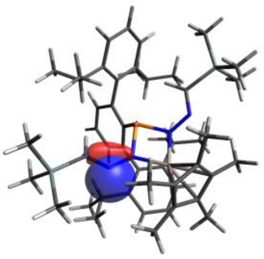

LP1(N5)
−10.8 eV
s: 28.9 %
p: 70.9 %
P: 1.9 %
N6: 93.8 %

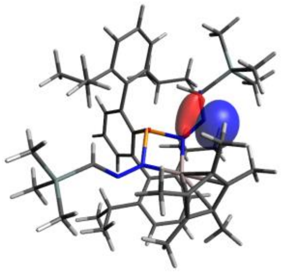

LP(N21)
−10.7 eV
s: 28.2 %
p: 71.7 %
P: 2.0 %
N21: 93.6 %

Figure S119: Selected NLMOs of **5P_CN** at the PBE0-D3/def2-TZVP//PBE0-D3/def2-SVP level of theory.

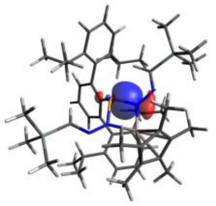

σ(P2-N3)
−17.5 eV
P: 28.3 %
N: 69.4 %

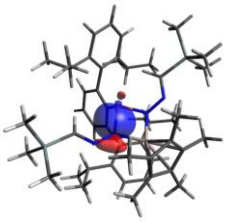

σ(P2-N4)
−17.7 eV
P: 28.0 %
N: 70.0 %

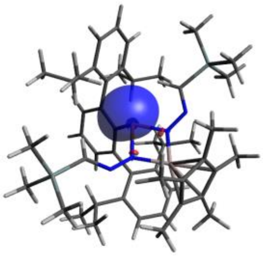

LP(P1)
−11.8 eV
s: 58.6 %
p: 41.3 %
P: 94.3 %
Al: 1.6 %

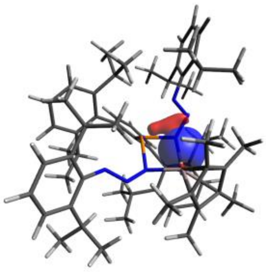

LP1(N3)
−16.0 eV
s: 38.3 %
p: 61.6 %
Al: 9.4 %
N3: 88.7 %

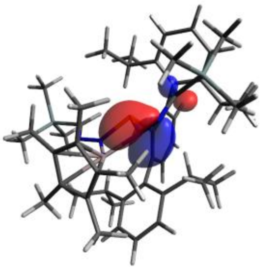

LP2(N3)
−8.7 eV
s: 0.9 %
p: 99.0 %
Al: 1.4 %
N21: 5.0
N3: 85.3 %

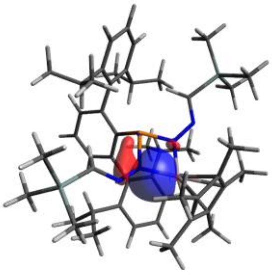

LP1(N4)
−15.6 eV
s: 36.5 %
p: 63.4 %
N4: 89.2 %
Al: 8.4

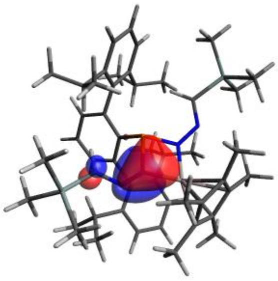

LP2(N4)
−8.5 eV
s: 37.4 %
p: 62.4 %
Al: 1.7 %
N5: 5.3 %
N4: 84.5 %

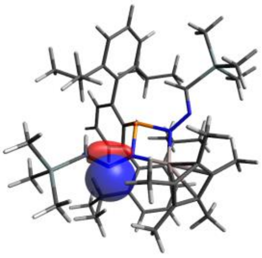

LP1(N5)
−10.8 eV
s: 28.9 %
p: 70.9 %
P: 1.9 %
N6: 93.8 %

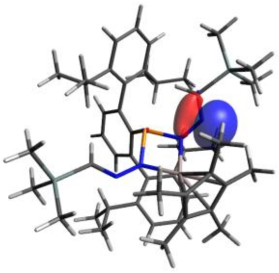

LP(N21)
−10.7 eV
s: 28.2 %
p: 71.7 %
P: 2.0 %
N21: 93.6 %

5.1.5 Kohn-Sham Orbitals

In the following selected frontier molecular orbitals at the PBE0-D3/def2-TZVP//PBE0-D3/def2-SVP level of theory for 1P-4P are shown. Note: The orbitals for the corresponding arsenic species are similar with only minimally different energy levels, while the order is the same. Therefore only the P-congeners are shown.

Figure S120: Selected Kohn-Sham orbitals of **1P** at the PBE0-D3/def2-TZVP//PBE0-D3/def2-SVP level of theory.

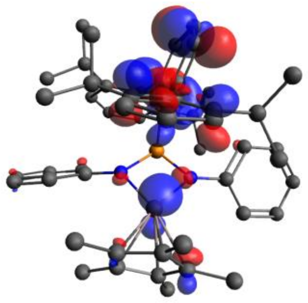

LUMO+1
−0.43 eV

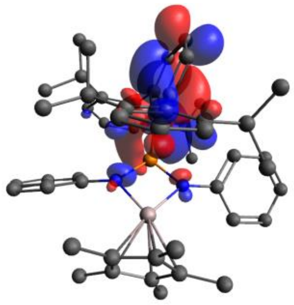

LUMO
−1.11 eV

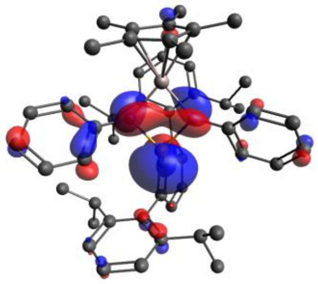

HOMO
−5.27 eV
s: 58.6 %
p: 41.3 %
P: 94.3 %
Al: 1.6 %

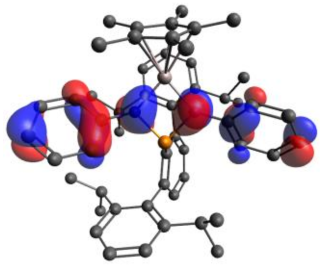

HOMO-1
−5.61 eV
s: 38.3 %
p: 61.6 %
Al: 9.4 %
N3: 88.7 %

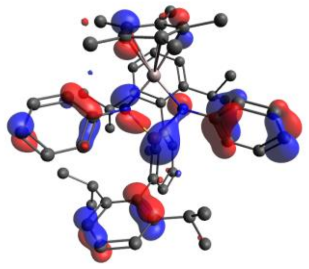

HOMO-2
−6.35 eV
s: 0.9 %
p: 99.0 %
Al: 1.4 %
N21: 5.0
N3: 85.3 %

Figure S121: Selected Kohn-Sham orbitals of **2P** at the PBE0-D3/def2-TZVP//PBE0-D3/def2-SVP level of theory.

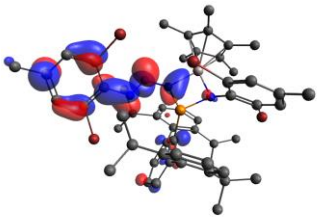

LUMO
−1.24 eV

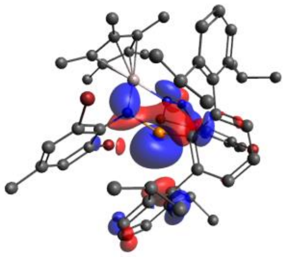

HOMO
−5.49 eV

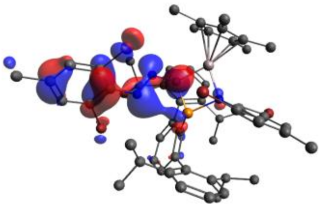

HOMO-1
−5.96 eV

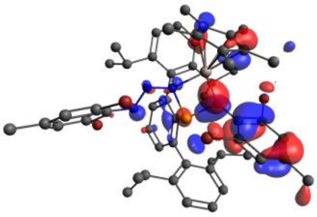

HOMO-2
−6.34 eV

Figure S122: Selected Kohn-Sham orbitals of **3P** at the PBE0-D3/def2-TZVP//PBE0-D3/def2-SVP level of theory.

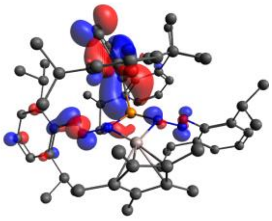

LUMO
−1.19 eV

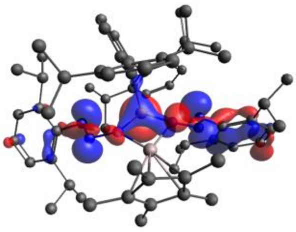

HOMO
−5.68 eV

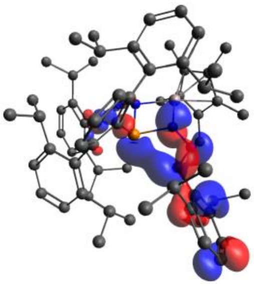

HOMO-1
−5.87 eV

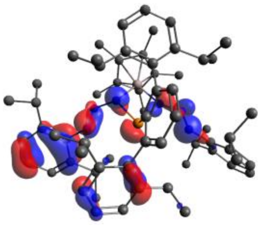

HOMO-2
−6.28 eV

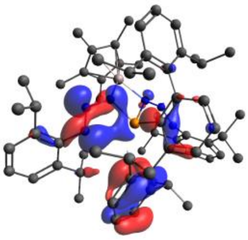

HOMO-3
−6.33 eV

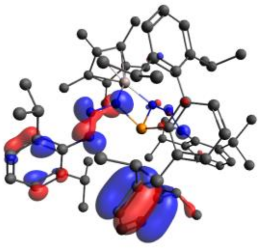

HOMO-4
−6.47 eV

Figure S123: Selected Kohn-Sham orbitals of **4P** at the PBE0-D3/def2-TZVP//PBE0-D3/def2-SVP level of theory.

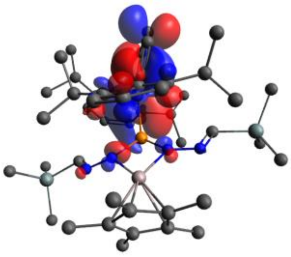

LUMO
−1.14 eV

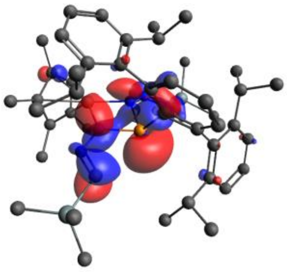

HOMO
−5.59 eV

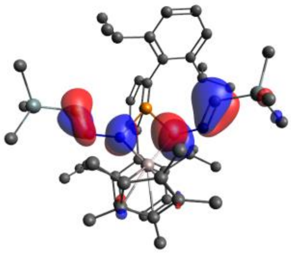

HOMO-1
−5.76 eV

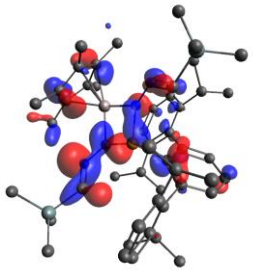

HOMO-2
−6.27 eV

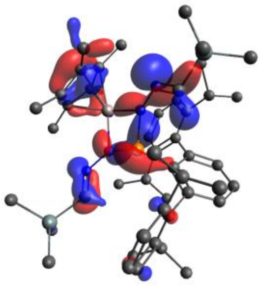

HOMO-3
−6.40 eV

6. References

These references are also cited in the manuscript. References that only appear in the ESI start at reference 78.

[37] M. Fischer, S. Nees, T. Kupfer, J. T. Goettel, H. Braunschweig, C. Hering-Junghans, *J. Am. Chem. Soc.* **2021**, *143*, 4106–4111.

[39] S. Nees, T. Wellnitz, F. Dankert, M. Härterich, S. Dotzauer, M. Feldt, H. Braunschweig, C. Hering-Junghans, *Angew. Chem. Int. Ed.* **2023**, *62*, e202215838.

[61] J. P. Perdew, K. Burke, M. Ernzerhof, *Phys. Rev. Lett.* **1996**, *77*, 3865–3868.

[62] J. P. Perdew, K. Burke, M. Ernzerhof, *Phys. Rev. Lett.* **1997**, *78*, 1396–1396.

[63] C. Adamo, V. Barone, *J. Chem. Phys.* **1999**, *110*, 6158–6170.

[64] S. Grimme, J. Antony, S. Ehrlich, H. Krieg, *J. Chem. Phys.* **2010**, *132*, 154104.

[65] S. Grimme, S. Ehrlich, L. Goerigk, *J. Comput. Chem.* **2011**, *32*, 1456–1465.

[66] F. Weigend, R. Ahlrichs, *Phys. Chem. Chem. Phys.* **2005**, *7*, 3297–305.

[67] F. London, *Journal de Physique et le Radium* **1937**, *8*, 397–409.

[68] R. McWeeny, *Phys. Rev.* **1962**, *126*, 1028–1034.

[69] R. Ditchfield, *Mol. Phys.* **1974**, *27*, 789–807.

[70] K. Wolinski, J. F. Hinton, P. Pulay, *J. Am. Chem. Soc.* **1990**, *112*, 8251.

[71] J. R. Cheeseman, G. W. Trucks, T. a Keith, M. J. Frisch, *J. Chem. Phys.* **1996**, *104*, 5497–5509.

[72] C. J. Jameson, A. De Dios, A. Keith Jameson, *Chem. Phys. Lett.* **1990**, *167*, 575–582.

[73] E. D. Glendening, J. K. Badenhoop, A. E. Reed, J. E. Carpenter, J. A. Bohmann, C. M. Morales, C. R. Landis, F. Weinhold, *NBO 6.0*, Theoretical Chemistry Institute, University of Wisconsin, Madison, **2013**.

[74] J. E. Carpenter, F. Weinhold, *J. Mol. Struct.: THEOCHEM* **1988**, *169*, 41–62.

[75] F. Weinhold, J. E. Carpenter, in *The Structure of Small Molecules and Ions* (Eds.: R. Naaman, Z. Vager), Springer, Boston, MA, **1988**, pp. 227–236.

[76] F. Weinhold, C. R. Landis, *Valency and Bonding. A Natural Bond Orbital Donor-Acceptor Perspective*, Cambridge University Press, **2005**.

[77] T. Lu, F. Chen, *J. Comput. Chem.* **2012**, *33*, 580–592.

---

[78] S. K. Pandey, A. Steiner, H. W. Roesky, S. Kamepalli, A. H. Cowley, in *Inorganic Syntheses, Vol. 31*, **1997**, pp. 148-150.

[79] P. Awolade, N. Cele, N. Kerru, P. Singh, *Mol. Diversity* **2021**, *25*, 2201-2218.

[80] S. W. Kwok, J. R. Fotsing, R. J. Fraser, V. O. Rodionov, V. V. Fokin, *Organic Lett.* **2010**, *12*, 4217-4219.

[81] H. Braunschweig, F. Hupp, I. Krummenacher, L. Mailänder, F. Rauch, *Chem. Eur. J.* **2015**, *21*, 17844-17849.

[82] F. X. Kohl, P. Jutzi, *J. Organomet. Chem.* **1983**, *243*, 119-121.

[83] F. Meloni, W. D. G. Brittain, L. Male, C. S. Le Duff, B. R. Buckley, A. G. Leach, J. S. Fossey, *Tetrahedron Chem.* **2022**, *1*, 100004.

[84] M. L. Luetkens Jr., A. P. Sattelberger, H. H. Murray, J. D. Basil, J. P. Fackler Jr., R. A. Jones, D. E. Heaton, in *Inorg. Syntheses*, **1989**, pp. 7-12.

[85] G. M. Sheldrick, *Acta Cryst. A* **2015**, *71*, 3–8.

[86] G. M. Sheldrick, *Acta Crystallogr. Sect. C: Struct. Chem.* **2015**, *71*, 3–8.

[87] G. M. Sheldrick, *SADABS Version 2*, University of Göttingen, Germany, **2004**.

[88] A. M. Arif, B. L. Benac, A. H. Cowley, R. A. Jones, K. B. Kidd, C. M. Nunn, A. Kuckowski, S. Schulz, P. R. Harris, D. A. Atwood, C. Knapp, C. Carmalt, J. Weßing, K. Freitag, G. Chelladurai, R. A. Fischer, J. Arnold, H. W. Roesky, K. Freitag, G. S. Hair, D. Barisic, R. Anwander, **2018**. Synthetic routes to phosphido and arsenide derivatives of the group 13 metals aluminum, gallium, and indium, tris(tert-butyl)gallium and its reactions with ammonia, and the aluminum(I) species pentamethylcyclopentadienyl aluminum tetramer. In *Inorganic Syntheses*, P.P. Power (Ed.). https://doi.org/10.1002/9781119477822.ch7

[89] C. Ganesamoorthy, S. Loerke, C. Gemel, P. Jerabek, M. Winter, G. Frenking, R. A. Fischer, *Chem. Commun.* **2013**, *49*, 2858-2860.

[90] J.-E. Siewert, A. Schumann, C. Hering-Junghans, *Dalton Trans.* **2021**, *50*, 15111-15117.

[91] B. R. Barnett, C. C. Mokhtarzadeh, J. S. Figueroa, P. Lummis, S. Wang, J. D. Queen, J. Gavenonis, N. Schüwer, T. D. Tilley, J. N. Boynton, P. P. Power, T. B. Ditri, N. Weidemann, B. R. Barnett, D. W. Agnew, P. W. Smith, T. B. Ditri, A. E. Carpenter, D. W. Agnew, P. W. Smith, J. K. Pratt, N. D. Mendelson, J. D. Queen, **2018**. Terphenyl Ligands and complexes. In *Inorganic Syntheses*, P.P. Power (Ed.). https://doi.org/10.1002/9781119477822.ch55

[92] E. Zander, J. Bresien, V. V. Zhivonitko, J. Fessler, A. Villinger, D. Michalik, A. Schulz, *J. Am. Chem. Soc.* **2023**, 145, 26, 14484–14497.

[93] B. Buster, A. A. Diaz, T. Graham, R. Khan, M. A. Khan, D. R. Powell, R. J. Wehmschulte, *Inorg. Chim. Acta* **2009**, *362*, 3465-3474.

[94] P. Gupta, J.-E. Siewert, T. Wellnitz, M. Fischer, W. Baumann, T. Beweries, C. Hering-Junghans, *Dalton Trans.* **2021**, *50*, 1838-1844.

[95] Gaussian 16, Revision C.01, M. J. Frisch, G. W. Trucks, H. B. Schlegel, G. E. Scuseria, M. A. Robb, J. R. Cheeseman, G. Scalmani, V. Barone, G. A. Petersson, H. Nakatsuji, X. Li, M. Caricato, A. V. Marenich, J. Bloino, B. G. Janesko, R. Gomperts, B. Mennucci, H. P. Hratchian, J. V. Ortiz, A. F. Izmaylov, J. L. Sonnenberg, D. Williams-Young, F. Ding, F. Lipparini, F. Egidi, J. Goings, B. Peng, A. Petrone, T. Henderson, D. Ranasinghe, V. G. Zakrzewski, J. Gao, N. Rega, G. Zheng, W. Liang, M. Hada, M. Ehara, K. Toyota, R. Fukuda, J. Hasegawa, M. Ishida, T. Nakajima, Y. Honda, O. Kitao, H. Nakai, T. Vreven, K. Throssell, J. A. Montgomery, Jr., J. E. Peralta, F. Ogliaro, M. J. Bearpark, J. J. Heyd, E. N. Brothers, K. N. Kudin, V. N. Staroverov, T. A. Keith, R. Kobayashi, J. Normand, K. Raghavachari, A. P. Rendell, J. C. Burant, S. S. Iyengar, J. Tomasi, M. Cossi, J. M. Millam, M. Klene, C. Adamo, R. Cammi, J. W. Ochterski, R. L. Martin, K. Morokuma, O. Farkas, J. B. Foresman, and D. J. Fox, Gaussian, Inc., Wallingford CT, **2016**.

[96] F. Neese, *WIREs Comput. Mol. Sci.* **2018**, *8*, e1327.

[97] Gaussian 09, Revision A.02, M. J. Frisch, G. W. Trucks, H. B. Schlegel, G. E. Scuseria, M. A. Robb, J. R. Cheeseman, G. Scalmani, V. Barone, G. A. Petersson, H. Nakatsuji, X. Li, M. Caricato, A. Marenich, J. Bloino, B. G. Janesko, R. Gomperts, B. Mennucci, H. P. Hratchian, J. V. Ortiz, A. F. Izmaylov, J. L. Sonnenberg, D. Williams-Young, F. Ding, F. Lipparini, F. Egidi, J. Goings, B. Peng, A. Petrone, T. Henderson, D. Ranasinghe, V. G. Zakrzewski, J. Gao, N. Rega, G. Zheng, W. Liang, M. Hada, M. Ehara, K. Toyota, R. Fukuda, J. Hasegawa, M. Ishida, T. Nakajima, Y. Honda, O. Kitao, H. Nakai, T. Vreven, K. Throssell, J. A. Montgomery, Jr., J. E. Peralta, F. Ogliaro, M. Bearpark, J. J. Heyd, E. Brothers, K. N. Kudin, V. N. Staroverov, T. Keith, R. Kobayashi, J. Normand, K. Raghavachari, A. Rendell, J. C. Burant, S. S. Iyengar,

J. Tomasi, M. Cossi, J. M. Millam, M. Klene, C. Adamo, R. Cammi, J. W. Ochterski, R. L. Martin, K. Morokuma, O. Farkas, J. B. Foresman, and D. J. Fox, Gaussian, Inc., Wallingford CT, **2016**.

[98] C. Riplinger, F. Neese, *J. Chem. Phys.* **2013**, *138*, 034106.

[99] D. G. Liakos, M. Sparta, M. K. Kesharwani, J. M. L. Martin, F. Neese, *J. Chem. Theory Comput.* **2015**, *11*, 1525–1539.

[100] C. Riplinger, P. Pinski, U. Becker, E. F. Valeev, F. Neese, *J. Chem. Phys.* **2016**, *144*, 024109.

[101] C. J. Cramer, *Essentials of Computational Chemistry: Theories and Models*, John Wiley & Sons, Ltd, Chichester, UK, **2004**.

[102] A. V. Marenich, C. J. Cramer, D. G. Truhlar, *J. Phys. Chem. B* **2009**, *113*, 6378–6396.

[103] G. K. Gransbury, S. C. Corner, J. G. C. Kragskow, P. Evans, H. M. Yeung, W. J. A. Blackmore, G. F. S. Whitehead, I. J. Vitorica-Yrezabal, N. F. Chilton and D. P. Mills, *ChemRxiv* 2023, DOI: 10.26434/chemrxiv-2023-28z84.
